# A tumor microenvironment-specific gene expression signature predicts chemotherapy resistance in colorectal cancer patients

**DOI:** 10.1038/s41698-021-00142-x

**Published:** 2021-02-12

**Authors:** Xiaoqiang Zhu, Xianglong Tian, Linhua Ji, Xinyu Zhang, Yingying Cao, Chaoqin Shen, Ye Hu, Jason W. H. Wong, Jing-Yuan Fang, Jie Hong, Haoyan Chen

**Affiliations:** 1 State Key Laboratory for Oncogenes and Related Genes, Division of Gastroenterology and Hepatology, Key Laboratory of Gastroenterology and Hepatology, Ministry of Health, Shanghai Institute of Digestive Disease, Renji Hospital, School of Medicine, Shanghai Jiao Tong University, Shanghai, China; 2 School of Biomedical Sciences, Li Ka Shing Faculty of Medicine, The University of Hong Kong, Pokfulam, Hong Kong SAR China; 3 Department of Gastroenterology, Tongren Hospital, Shanghai Jiao Tong University School of Medicine, Shanghai, China; 4 Department of Gastrointestinal Surgery, Renji Hospital, School of Medicine, Shanghai Jiao Tong University, Shanghai, China; 5 Department of Gastroenterology, Xinhua Hospital, Shanghai Jiao Tong University School of Medicine, Shanghai, China; 6 Women’s Cancer Program at the Samuel Oschin Comprehensive Cancer Institute, Cedars-Sinai Medical Center, Los Angeles, CA USA

**Keywords:** Colorectal cancer, Cancer microenvironment

## Abstract

Studies have shown that tumor microenvironment (TME) might affect drug sensitivity and the classification of colorectal cancer (CRC). Using TME-specific gene signature to identify CRC subtypes with distinctive clinical relevance has not yet been tested. A total of 18 “bulk” RNA-seq datasets (total *n* = 2269) and four single-cell RNA-seq datasets were included in this study. We constructed a “Signature associated with FOLFIRI resistant and Microenvironment” (SFM) that could discriminate both TME and drug sensitivity. Further, SFM subtypes were identified using *K*-means clustering and verified in three independent cohorts. Nearest template prediction algorithm was used to predict drug response. TME estimation was performed by CIBERSORT and microenvironment cell populations-counter (MCP-counter) methods. We identified six SFM subtypes based on SFM signature that discriminated both TME and drug sensitivity. The SFM subtypes were associated with distinct clinicopathological, molecular and phenotypic characteristics, specific enrichments of gene signatures, signaling pathways, prognosis, gut microbiome patterns, and tumor lymphocytes infiltration. Among them, SFM-C and -F were immune suppressive. SFM-F had higher stromal fraction with epithelial-to-mesenchymal transition phenotype, while SFM-C was characterized as microsatellite instability phenotype which was responsive to immunotherapy. SFM-D, -E, and -F were sensitive to FOLFIRI and FOLFOX, while SFM-A, -B, and -C were responsive to EGFR inhibitors. Finally, SFM subtypes had strong prognostic value in which SFM-E and -F had worse survival than other subtypes. SFM subtypes enable the stratification of CRC with potential chemotherapy response thereby providing more precise therapeutic options for these patients.

## Introduction

Colorectal cancer (CRC) is a disease with great heterogeneity characterized as distinctive molecular pathogenesis, histogenesis, and drug sensitivity^1,2^. The heterogeneity of CRC has been revealed by using whole-genome sequencing (WGS), epigenetic analysis, and gene expression profiles. For instance, at the genetic level, some DNA markers have been recognized including microsatellite instability (MSI), CpG island methylator phenotype (CIMP), chromosomal instability (CIN), BRAF, and KRAS mutations. Further, tumor microenvironment (TME) components consist of distinctive and interacting cell populations, including tumor epithelial cells, immune cells, and cancer-associated fibroblasts (CAFs)^3,4^. The diversity of TME has made it possible to perform immune classification of cancers regarding prognosis^5^, chemotherapy^6^, and immunotherapy^7^ response prediction. For example, MSI tumor displays higher densities of type 1 T helper, effector memory T cells^8^, and has good prognosis. It has also shown significant benefit from immune checkpoint blockade therapy, anti-PD1/PDL1 (refs. ^9,10^). Besides, increasing evidence indicated that the dysbiosis of gut microbiota can lead to the development and progression of CRC by inducting chronic inflammatory state and immune response, regulating stem cell dynamics, producing toxic and genotoxic metabolites, and affecting the host metabolism^11^. Microbial communities also varied in different parts of gut, including distal colon and proximal ileum, both of which admittedly have distinctive prognosis and treatment strategies^12^.

Gene expression-based classifying has shown high efficiency for cancer classification. Several molecular classifiers in CRC have been built using global gene expression analysis, including colon cancer subtypes (CCS)^13^, the Colorectal Cancer Assigner^14^, colon cancer molecular subtype systems, and colorectal cancer subtyping consortium (CRCSC)^15^. Most of them were constructed directly based on global expression profiles using unsupervised consensus-based clustering algorithms. However, these transcriptome signals derived from both cancer cells and noncancerous components. And these approaches can’t distinguish these signals automatically when applied to classification. Recent studies have consistently suggested that TME components played important roles in defining CRC with poor prognosis and immune escape^16–18^. Given that TME also makes great contribution to chemotherapy and immunotherapy sensitivity^7,19,20^, and FOLFIRI (combination of folinic acid, fluorouracil, and irinotecan) and FOLFOX (combination of 5-fluorououracil, leukovorin, and oxaliplatin) are two of the most common first-line treatment strategies for metastatic CRC (mCRC), we proposed that using gene expression profiles that could discriminate both TME and drug sensitivity might be an effective way to define CRC molecular subtypes.

To test this hypothesis, a total of 2269 gene expression profiles from 18 datasets and another four single-cell RNA-sequencing (scRNA-seq) datasets were analyzed in this study to (i) construct a gene signature consisted of genes that could discriminate both TME and drug sensitivity; (ii) further identify a robust molecular classification using this gene signature; and (iii) evaluate the associations between CRC subtypes and clinicopathological factors, common oncogenic mutations, genetic changes, signaling pathways, drug sensitivity, immune infiltration, prognosis, and gut microbiota patterns.

## Results

### A chemotherapy resistant gene signature associated with TME

**Fig. 1:**
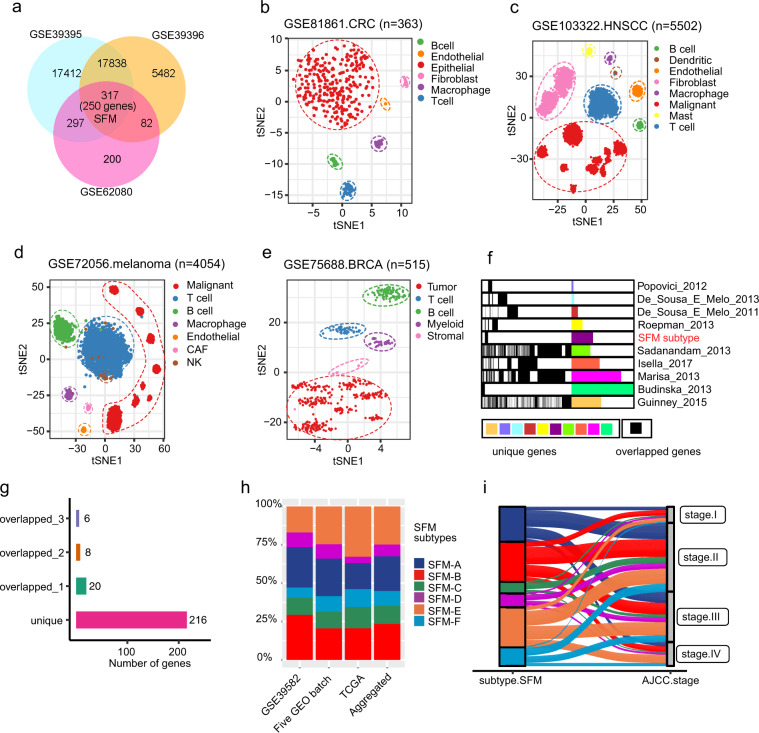
Construction of SFM signature and subtypes. **a** Venn diagram of differentially expressed probe sets in three datasets showing intersection of 317 probes (250 unique genes). **b**–**e** *t*-SNE plot of SFM gene expression profiles from four datasets (**b**, CRC, *n* = 363; **c**, HNSCC, *n* = 5502; **d**, melanoma, *n* = 4054; **e**, BRCA, *n* = 515). **f**–**g** Genes overlapped between SFM and published gene signatures. Each column indicates each gene signature and each row indicate each gene. Shared genes are indicated with black lines (**f**). Graph depicts the quantification of overlap across these signatures (**g**). **h** Proportion of SFM subtypes in individual and aggregated datasets. **i** Sankey diagram showing how each SFM subtype contributes to AJCC stage classification.

A total of 2269 bulk gene expression profiles of CRC patients were included in this study (Supplementary Table. 1). Supplementary Figure 1a summarized the schematic workflow of this study. We identified 896 probe sets that were involved with FOLFIRI response. To further select genes that differed from TME, we further identified genes with significantly discriminative expression amongst TME components, such as tumor epithelial cells, immune cells, and stromal cells (see “Methods” section for details). After overlapping the differential probes, we acquired a list of 317 probes which corresponded to 250 unique genes (Fig. 1a, Supplementary Fig. 1b, and Supplementary Table 2) and referred to this gene signature as the “Signature associated with FOLFIRI resistant and Microenvironment” (SFM). To confirm that SFM signature could discriminate TME, we applied SFM signature to four scRNA-seq datasets of human CRC, head and neck squamous cell carcinoma (HNSCC), melanoma and breast cancer (BRCA). Each data contained malignant and nonmalignant cells derived from human tumors, thus enabling to validate the ability of SFM to define the heterogeneity of TME. We explored the global structure of SFM expression in these four scRNA-seq datasets using *t*-distributed stochastic neighbor embedding (*t*-SNE). *t*-SNE plot indicated that SFM formed distinct clusters corresponding to different cells types in all the four scRNA-seq datasets, implying the universal ability of SFM to discriminate TME (Fig. 1b–e). SFM expression profiles and SFM gene signature scores further confirmed our observation (Supplementary Fig. 1c–g). Moreover, we found that malignant cells from different origins (i.e., from which tumor patients) could also form distinct clusters, while nonmalignant cells could cluster together regardless of the origins indicating that SFM expression of normal cells had no strong interpatient heterogeneity. Next, we evaluated overlaps among nine published gene signatures with SFM and found large overlaps among some of these gene signatures (Fig. 1f and Supplementary Table. 3). Intriguingly, the SFM displayed very limited overlaps with other gene signatures. A total of 216 out of the SFM genes were unique; 20, 8, and 6 genes were shared with other gene signatures for one, two, and three times, respectively (Fig. 1g).

### *K*-means clustering CRC subtypes

To test if SFM could classify CRC subtypes, we used *k*-means clustering algorithm to the classification using the SFM in discovery dataset (GSE39582). By doing so, six subtypes were identified and referred as CRC SFM subtypes from SFM-A to SFM-F (Supplementary Fig. 1h and Supplementary Table. 4). The robustness of SFM classification was further validated in two large cohorts and our Renji cohort (Supplementary Fig. 1i–k and Supplementary Table. 4). In Renji cohort, only four SFM subtypes were classified mainly because of small number of sample size. Overall, the proportion of each subtype was similar in three large datasets (Fig. 1h). Given that stromal signal strongly affects the transcriptional classification^21^ and SFM consists of genes predominately expressed in stomal content, we additionally applied SFM to a patient-derived xenografts CRC dataset (GSE76402, *n* = 515), in which the stromal components from the original tumors have been substituted by murine counterparts^21^. As expected, the SFM classification was not perfect since the SFM genes were not clearly discriminative amongst resulted clusters (Supplementary Fig. 1l). This implied that the SFM classification was also depended on original TME components. After combining the subtype information, we saw that SFM-A (23%), SFM-B (23%), and SFM-E (25%) accounted for larger proportions followed by SFM-C (12%), SFM-F (10%), and SFM-D (8%). We then compared the overlap of SFM subtypes with published CRC classifiers. For each classifier, we combined all used samples that were annotated with individual subtype information. As expected, we saw that SFM subtypes had strong overlaps with other classifiers (Supplementary Fig. 1m). For instance, compared to the CMS subtypes developed by Guinney et al.^15^, 61% of SFM-A and 80% of SFM-B were CMS2; 81% of SFM-C were CMS1; 33% of SFM-1 were SFM-C; half of SFM-D and E, and 92% of SFM-F were CMS4. As for CCS classification developed by De. Sousa et al.^13^, almost SFM-A (96%) and SFM-B (75%) belonged to CCS1; 86% of SFM-C belonged to CCS2; and all of SFM-F were CCS3. Some overlaps could also be observed for the remaining subtypes including The Cancer Genome Atlas (TCGA) CRC molecular subtypes^22^, C1–C6 clusters developed by Marisa et al.^23^, CRISA-CRISE clusters by Isella et al.^21^, and five molecular subtypes by Sadanandam et al.^14^.

### Leading peculiarities of SFM subtypes

Further analysis implied that SFM subtypes were associated with distinct clinicopathological, molecular and phenotypic characteristics, and specific enrichments of gene signatures and signaling pathways. Firstly, we found higher proportion of stage II and III in each SFM cluster compared to stage I and IV (Fig. 1i). Higher proportion of stage IV could be observed in SFM-E (18%) and SFM-F (17%). Secondly, 74% of SFM-C belonged to MSI tumors and 60% MSI tumors were assigned to SFM-C (*P* < 0.0001, Supplementary Fig. 1n). Furthermore, SFM-C and SFM-F were mainly endowed with hypermutation genotype (*P* < 0.0001, Supplementary Fig. 1n), proximal colon tumors (*P* < 0.0001, Supplementary Fig. 1n). On the contrary, tumors classified as SFM-A-B-D-E showed CIN+ (*P* < 0.0001, Supplementary Fig. 1n), MSS, non-hypermutant features. In addition to SFM-C, SFM-F was the second subtype enriched with BRAF mutation (*P* < 0.0001, Supplementary Fig. 1n). And TP53 was more frequently mutant in SFM-B and SFM-D (*P* = 0.0034, Supplementary Fig. 1n), while KRAS mutations were more frequently occurred in SFM-A (*P* = 0.0001, Supplementary Fig. 1n). As for oncogenic mutation, we further focused on 95 driver mutations of CRC in TCGA dataset^24^. Among these 95 drivers, SFM-C (33%, 26 in 80) and SFM-D (21%, 5 in 24) had higher proportion of samples that had more than seven mutant driver genes (Supplementary Fig. 1o). In addition, 53 of 95 gene mutation status had significant differences among SFM subtypes (*P* < 0.05, Supplementary Fig. 1p).

We used previously reported gene signatures to identify the cell and precursor origins of SFM subtypes based on the nearest template prediction (NTP) algorithm^25^ (Supplementary Fig. 1q, and Supplementary Tables 5 and 6). We applied an intestinal stem cell signature and a colon crypt signature to the four gene expression profiles. SFM-E and SFM-F were found remarkably enriched for “stem-like” phenotype (*P* < 0.0001) and SFM-B-E-F had hallmarks of colon base crypt (*P* < 0.0001). As epithelial-to-mesenchymal transition (EMT) has been regarded as a critical process in CRC progression^26^, we applied an EMT signature to our data similarly. The results indicated that SFM-D-E-F were enriched for EMT phenotype (*P* < 0.0001). Besides, serrated CRC is morphologically distinctive compared with conventional CRC and has been suggested to be involved with serrated neoplasia procedure^27^. We found that SFM-C-E-F were characterized as “serrated CRC” phenotype, whereas SFM-A-B-D displayed a “conventional CRC” phenotype (*P* < 0.0001).

We also analyzed dysregulated signaling pathways in each SFM subtype. Two thousand top up- and down-expressed genes in each SFM subtype were subjected to perform analysis (Supplementary Table. 7). Most of SFM subtypes were enriched in specific activated pathways (Supplementary Fig. 1r). Specifically, glucose metabolism was activated in SFM-A, while most signaling pathways were downregulated in this subtype. SFM-B showed upregulation of DNA replication/damage associated pathways. Upregulated interleukin-6/8 (IL-6/8) and downregulated EMT and fibroblast growth factor receptor associated pathways were found in SFM-C. SFM-D-E-F all displayed upregulation of cell focal adhesion, collagen formation, and integrin pathways. In SFM-D, IL-2 and IL-3 associated pathways were activated, while IL-5 mediated pathway was upregulated in SFM-E. SFM-E and SFM-F displayed significantly upregulated immune system and EMT pathways.

### SFM subtype predicted chemotherapy response

**Fig. 2:**
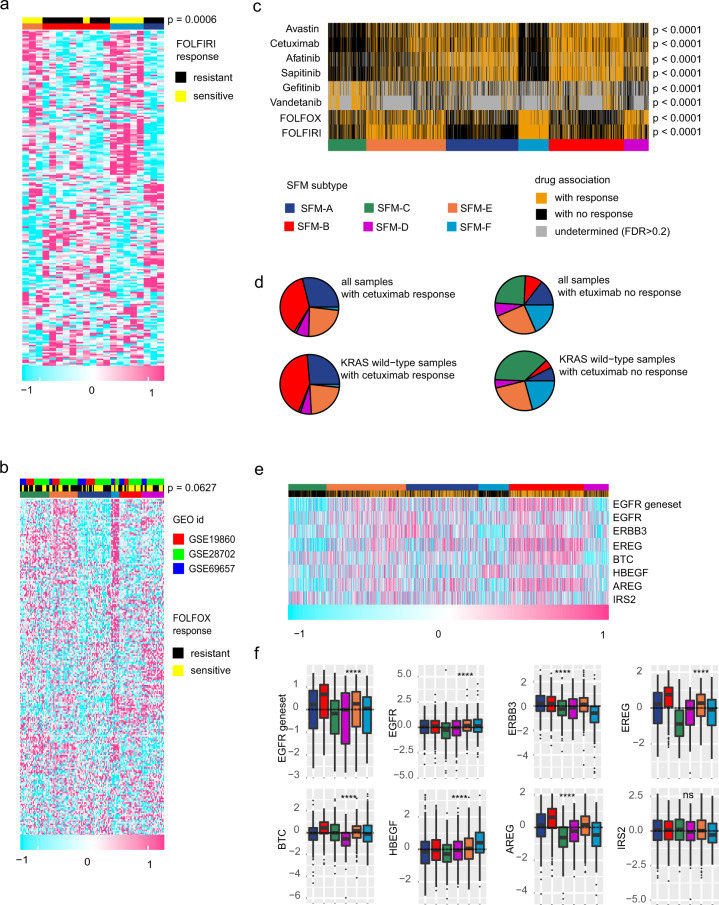
Distinct sensitivity among SFM subtypes to FOLFIRI and FOLFOX chemotherapy regimens and RGFR inhibitors. **a** Heatmap showing individual response of patients to FOLFIRI and their association with subtypes in GSE62080 dataset (*n* = 21). *P* value was calculated using Chi-squared test. **b** Heatmap showing individual responses of patients to FOLFOX and their association with subtypes in combined three GEO datasets (GSE19860, GSE28702, and GSE69657, *n* = 142). *P* value was calculated using Chi-squared test. **c** Heatmap showing association of individual CRC patient’s response to FOLFIRI, FOLFOX, and EGFR inhibitors. In these analyses, samples with FDR < 0.2 were regarded as significant. **d** Prevalence of SFM subtypes regarded as sensitive or resistant to cetuximab in all CRC patients (*n* = 1752) or only in KRAS wild-type patients (*n* = 637). **e** Heatmap showing response to cetuximab, quantified expression of gene set of the EGFR pathway activity by applying GSVA and expression of individual genes of the EGFR gene set among SFM subtypes. **f** Box plots of the EGFR gene set and individual genes of the EGFR gene set among SFM subtypes. Box hinges represent first and third quartiles, and middle represents the median. The upper and lower whiskers extend from hinges up and down indicate the most extreme values that are within 1.5 × IQR (interquartile range) of the respective hinge.

Since SFM signature was correlated with FOLFIRI sensitivity derived from GSE62080, we first performed *K*-means clustering using the SFM signature in GSE62080 dataset to examine whether SFM subtypes were associated with drug response. Our results showed that 21 cases in GSE62080 could be classified into four of the SFM subtypes (Fig. 2a, Supplementary Fig. 2a, and Supplementary Table. 8). All of the SFM-E and SFM-F were responsive to FOLFIRI, and eight out of nine FOLFIRI responsive CRCs were defined as SFM-E and SFM-F (*P* = 0.0006). On the contrary, SFM-A and SFM-B were resistant to FOLFIRI. Similarly, we also tested another chemotherapy regimen FOLFOX response in combined datasets (*n* = 142, Fig. 2b, Supplementary Fig. 2b, and Supplementary Table. 8). Interestingly, we found that 75% of SFM-F (*n* = 6), as well as 36% of SFM-E (*n* = 10), 55% of SFM-D (*n* = 12) and 64% of SFM-B (*n* = 14) responded to FOLFOX (*P* = 0.0627). In addition, we included another two datasets (GSE72970 and GSE104645) in which samples were treated with FOLFIRI or FOLFOX, and the survival information was available. A total of 158 samples were classified into five main SFM subtypes (Supplementary Fig. 2c). Although there was no significant difference among the subtypes in terms of the response (*P* = 0.1003, Supplementary Fig. 2d) with more than half of SFM-A/B resistant, but 75% of SFM-C responsive to FOLFIRI or FOLFOX. Given that there were no significant differences in survival between responder and nonresponder samples in these two datasets, respectively, as indicated by He et al.^28^, we compared the survival differences among SFM subtypes. Interestingly, our results demonstrated that SFM-A/B had shorter OS and PFS, while SFM-C and -E had better survival, and SFM-D and -F were intermediate (log-rank *P* < 0.000 for OS, *P* < 0.0001 for PFS, Supplementary Fig. 2e, f). Hence, these results to some extent suggested that at least SFM-A/B subtype could not benefit from FOLFIRI or FOLFOX treatment. To comprehensively compare the drug-response differences among SFM subtypes, we applied previous drug gene signatures to gene expression profiles using NTP algorithm, including FOLFIRI, FOLFOX, and vascular endothelial growth factor (VEGF) or epidermal growth factor receptor (EGFR) inhibitors (Supplementary Table. 5). Overall, the drug sensitivity among SFM subtypes were distinctive (Fig. 2c, Supplementary Fig. 2g, and Supplementary Table. 8). Specifically, the FOLFIRI response signature was significantly (false discovery rate, FDR < 0.2) associated with 99% (*n* = 166) of SFM-F, 80% (*n* = 328) of SFM-E, and 67% (*n* = 84) of SFM-D subtype samples, as compared to only 10% (*n* = 37) of SFM-A, 30% (*n* = 108) of SFM-B, and 28% (*n* = 50) of SFM-C subtype (*P* < 0.0001). Similar results could also be found for FOLFOX response (*P* < 0.0001). We also applied another FOLFOX response signature of five gene pairs which has shown good performance to do the prediction by NTP or the way used in corresponding study^28^. Similar results were obtained in these two ways (Supplementary Fig. 2h, i), but not consistent with what we found that SFM-D/E/F were prone to response to FOLFOX. Further analysis on these two FOLFOX gene signatures, five gene pairs signature from He et al.^28^ and 315 gene signature from Tong et al.^29^ suggested that there were no overlapped genes between these two gene signatures, implying the bias between them. Indeed, the 315 gene signatures were more robust because these genes were not only differentially expressed between responders and nonresponders in both pre-chemotherapy and post-chemotherapy samples, respectively, but also validated between parental and resistant cancer cells. In addition, we found that most of the SFM-A and SFM-B were significantly (FDR < 0.2) correlated with EGFR inhibitors (*P* < 0.0001, Fig. 2c). For instance, 68% (*n* = 237) of SFM-A, 80% (*n* = 305) of SFM-B, and 50% (*n* = 193) of SFM-E responded to cetuximab. Similar results could be observed for avastin, afatinib, and sapitinib. The results also indicated that SFM-C was responsive to EGFR tyrosine kinase inhibitors, including gefitinib and vandetanib. Ninety one percentage (*n* = 100) and 96% (*n* = 96) of SFM-C were strikingly associated with gefitinib and vandetanib response signature, respectively. As one of the EGFR-specific monoclonal antibody, cetuximab has been applied to treatment for mCRC harboring KRAS wild type. However, some studies also implied that cetuximab did not have obvious benefit in chemotherapy regimens regardless of KRAS status^30^. Thus, we only included KRAS wild-type samples to further validate cetuximab sensitivity across SFM subtypes. Again, we found that SFM-A-B-E could predict the cetuximab response (Fig. 2d). This result was more notable when combining these three SFM subtypes compared with the remaining SFM subtypes (Supplementary Fig. 2j, k). In view of this, several genes involved with EGFR pathway activity have been suggested to be associated with cetuximab response^31–33^. Consistently, this specific gene set showed higher expression in SFM-A-B-E subtypes (Fig. 2e, f and Supplementary Fig. 2l). It is also overt after combining SFM-A-B-E compared with the rest irrespective of KRAS phenotype (Supplementary Fig. 2m, n). To further confirm the association between EGFR inhibitors and SFM subtypes, we applied SFM signature to a combined dataset where samples were treated with cetuximab, GSE5851 (ref. ^32^) and PRJEB34338 (ref. ^34^; total *n* = 95). Considering the small sample size, three main clusters were identified including SFM-A/B, SFM-C, and SFM-D/E/F (Supplementary Fig. 2o). Overall, nearly half of SFM-A/B samples were responsive to cetuximab, but lowest fraction for SFM-C (20%, *P* = 0.0421, Supplementary Fig. 2p). Consistently, most of the EGFR pathway-associated genes were highly expressed in SFM-A/B, but not for SFM-C (Supplementary Fig. 2q, r). Together, these findings suggest that SFM subtypes have predictive value of cetuximab response regardless of KRAS phenotype.

### Distinctive TME among SFM subtypes

**Fig. 3:**
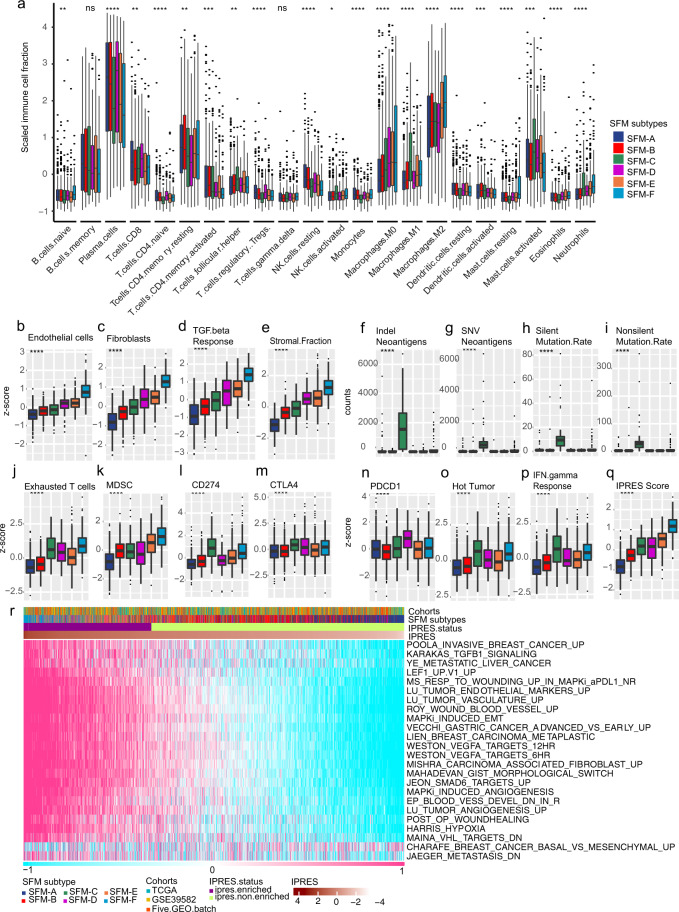
Immune and TME-associated differences across SFM subtypes. **a** Estimate immune cells filtration using CIBERSORT in aggregated dataset. The fraction of immune cells were plotted using box plot across SFM subtypes. Box plots of fractions of endothelial cells (**b**) and fibroblasts (**c**) by MCP-counter algorithm. TGF-β response score using GSVA (**d**), stromal fraction using ESTIMATE (**e**), neoantigen production (**f**, **g**), tumor mutation burden (**h**, **i**), proportion of exhausted cells (**j**), and MDSC (**k**) using GSVA, expression differences of checkpoint biomarkers, including CD274 (**l**), CTLA4 (**m**) and PDCD1 (**n**), hot tumor signature score (**o**), IFN-γresponse score (**p**), and IPRES score (**q**) using GSVA. **r** Estimate IPRES signature using GSVA. Cases were annotated as “IPRES enriched” when IPRES score were >0.35 unless were annotated as “IPRES non-enriched”. The statistical difference was examined usings the Kruskal–Wallis test. (ns, *P* > 0.05; *P < = 0.05; **P < = 0.01; ***P < = 0.001; ****P < = 0.0001). For **a**–**q**, box hinges represent first and third quartiles, and middle represents the median. The upper and lower whiskers extend from hinges up and down indicate the most extreme values that are within 1.5 × IQR (interquartile range) of the respective hinge.

Since the SFM signature could discriminate TME, we further compared TME component among SFM subtypes. Firstly, we explored cell fractions among SFM subtypes by CIBERSORT. Based on this algorithm, we found that SFM subtypes displayed different enrichment for immune cell populations (Fig. 3a, Supplementary Fig. 3a–c, and Supplementary Table. 9). SFM-A was enriched with memory B cells, memory CD4 T cells, regulatory T cells (Tregs), plasma cells, and rested dendritic cells and rested NK cells. SFM-B was characterized by increased memory CD4 T cells, activated dendritic cells, rested NK cells. SFM-C displayed high infiltration of activated NK cells, follicular helper T cells, M1 macrophage, activated mast cells, and neutrophils. SFM-D exhibited enrichment with activated naive CD4 T and B cells, plasma cells, CD8 T cells, and Tregs. SFM-E showed increases of follicular helper T cells, M0/1 macrophages, and neutrophils. SFM-F was enriched with naive B cells and macrophages, rested mast cells and neutrophils. In addition, SFM-D-E-F had displayed higher proportions of endothelial cells and fibroblasts (Fig. 3b, c). SFM-F had predominant stroma components with higher score of TGF-β response (Fig. 3d, e). As for clinical features of immunotherapy, we found that SFM-C displayed highest neoantigen production follow by SFM-F (Fig. 3f, g). Consistently, SFM-C-F exhibited higher mutation burden than other subtypes (Fig. 3h, i). Based on several gene signatures, we found that SFM-F was also enriched with exhausted T cells and myeloid-derived suppressor cells (MDSCs; Fig. 3j, k). Importantly, we found that checkpoint biomarkers were highly expressed in SFM-C-F, including CD274, PDCD1, and CTLA4 (Fig. 3l–n). These suggested that SFM-C-F were T cell suppressive. Further, both SFM-C and F were characterized as “hot” tumor and were responsive for IFN-γ response (Fig. 3o, p). However, SFM-F was enriched with innate anti-PD1 resistance (IPRES) gene signature (Fig. 3q, r) and displayed higher IPRES score, which meant that SFM-F had features of “nonresponder” of immunotherapy. Results above indicated that even though SFM-C and -F were T cell suppressive, they responded differently to immunotherapy. This might be explained by that SFM-C was enriched with MSI phenotype, in which the immune suppressive could be blocked by immune inhibitors, while SFM-F was enriched with stroma/EMT phenotype that could also lead to immune suppressive, but not reversed by immune inhibitors.

### SFM subtype was an independent predictor of CRC

**Fig. 4:**
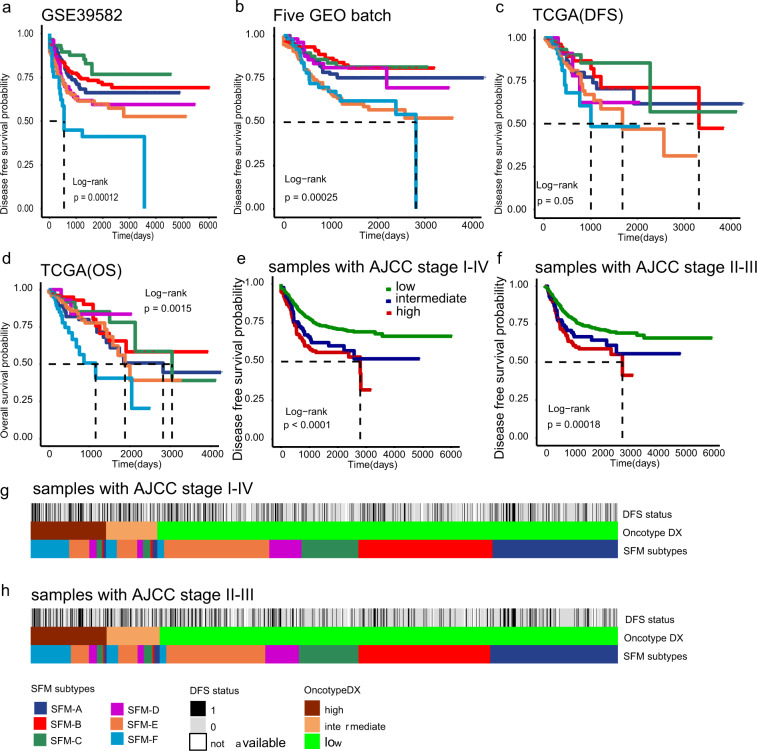
Survival differences across SFM subtypes. Kaplan–Meier plots of DFS and OS in training dataset (**a**, GSE39582) and validation dataset (**b**, five GEO batch; **c**, TCGA for DFS; and **d**, TCGA for OS). **e** Kaplan–Meier plots of Oncotype DX risk groups in stage I–IV cases. **f** Kaplan–Meier plots of Oncotype DX risk groups in stage II–III cases. The tick marks on the Kaplan–Meier curves indicated the censored patients. The differences between the curves were determined by the log-rank test. **g** Comparison of SFM subtypes and Oncotype DX classification in stage I–IV cases. **h** Comparison of SFM subtypes and Oncotype DX classification in stage II and III cases.

We examined SFM subtypes with survival to test its prognostic value. We first performed prognostic analysis in each dataset independently regardless of treatment (chemotherapy or radiotherapy) or AJCC stage. Interestingly, we saw significant association between SFM subtypes and DFS or OS (Fig. 4a–d), which was more remarkable after three datasets were combined (*P* < 0.0001, Supplementary Fig. 4a). Further, we assigned samples based on chemotherapy information. SFM subtypes still maintained significant association with DFS irrespective of chemotherapy (Supplementary Fig. 4b, c). The prognostic value of SFM subtypes were still significant in AJCC stage II and III patients, respectively, but not for stage I and IV patients probably because of the small sample size of stage I and IV patients (Supplementary Fig. 4d–g). Altogether, this suggested that SFM-E and SFM-F had worse prognosis likewise. We combined SFM-E and SFM-F as a single high-risk group versus the remaining four to confirm this. The binary classifier displayed strong prognostic value as expected (*P* < 0.0001, Supplementary Fig. 4h). In addition, we divided patents based on chemoradiotherapy information available. SFM subtypes showed significant prognostic value in non-chemoradiotherapy patients (*P* < 0.0001), but not in chemoradiotherapy patients (*P* = 0.11, Supplementary Fig. 4i, j). Then we performed similar analysis using the binary classifier. Interestingly, high-risk group had worse prognosis in non-chemoradiotherapy cases (*P* < 0.0001), but not for chemoradiotherapy cases (*P* = 0.095; Supplementary Fig. 4k, l). Although the result was not significant might because of small number of samples with chemoradiotherapy information, we saw a trend that chemoradiotherapy could improve DFS in SFM-E and SFM-F subtypes. As the Oncotype DX recurrence score has been regarded as a prognostic classifier in colon cancer^35,36^, we evaluated its prognostic value within combined datasets. This score did have prognostic value in all AJCC stage cases (*P* < 0.0001) or only stage II and III cases (*P* = 0.00018; Fig. 4e, f). Then we compared the proportions of subtypes between SFM subtypes and Oncotype DX classifier. We found that 77% of high-risk cases and 75% of intermediate-risk cases identified by Oncotype DX classifier could be classified into SFM high-risk group (SFM-E-F; Fig. 4g, h). This suggested that the SFM subtype had robust prognostic value. In addition, in univariate Cox regression analysis, most of the classifiers had at least one subtype that had significant difference (Supplementary Fig. 4m). However, when performed multivariate Cox regression analysis for each molecular subtype adjusted by age, gender, and AJCC stage, only SFM, CMS, CCS subtypes still had significant differences (Supplementary Fig. 4n). CCS3 subtype (*P* = 0.0002, HR = 11.32, 95% CI = 3.14–40.84) had strongest prognostic value followed by SFM-F (*P* = 0.0001, HR = 2.48, 95% CI = 1.51–3.96) and SFM-E (*P* = 0.0031, HR = 1.89, 95% CI = 1.24–2.89).

### Distinct gut microbiome patterns among SFM subtypes

**Fig. 5:**
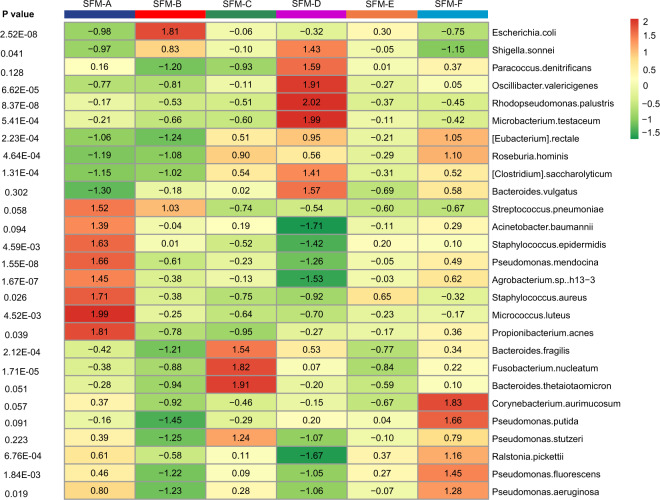
Heatmap showing the relative abundance of dominant bacterial across SFM subtypes. Kruskal–Wallis test was used to test the differences across SFM subtypes.

We performed the PathSeq algorithm in TCGA cohort. We acquired relative abundance value of 1093 microbes at the species level in 415 cases annotated with CRC subtypes and found that almost SFM subtypes harbored distinct bacterial communities (Fig. 5). Supplementary Table 10 displayed the top 15 highly enriched genera in each SFM subtype. The highest enriched bacterial species in SFM-A were *Micrococcus luteus* and *Propionibacterium acnes*. Bacterial species that highly enriched in SFM-A had lower enrichment in SFM-D, including *Staphylococcus aureus*, *Pseudomonas mendocina*, and *Acinetobacter baumannii*, etc. SFM-B was highly enriched with *Escherichia coli*. In addition, SFM-C had high enrichment for *Bacteroides thetaiotaomicron*, *Fusobacterium nucleatum*, and *Bacteroides fragilis*. SFM-D was enriched for *Microbacterium testaceum*, *Rhodopseudomonas palustris*, etc. And SFM-F displayed high enrichment for *Corynebacterium aurimucosum* and Pseudomonas putida. However, compared to other SFM subtypes, we did not see significantly enriched genera in SFM-E.

## Discussion

CRC is a disease with high heterogeneity just like other tumor types. The possible sources of heterogeneity of CRC derive from many aspects, such as genetic alterations, diversity of TME cell populations, and even the specific complex microbial community of gut. Increasing evidence indicated that TME has made great contribution to the development and progression of CRC. Gene expression profiles that can distinguish TME probably help to explain the heterogeneity of tumors. FOLFIRI and FOLFOX have been recommended as first-line backbone chemotherapy of mCRC by the Europe Society for Medical Oncology guidelines^37^. Although FOLFIRI or FOLFOX can significantly extend the median OS to >15 months, there are nearly 50% patients are not responsive^38^. Therefore, screening out those potentially responsive patients is also urgent. In this study, we took the advantages of comprehensive datasets to explore the heterogeneity of CRC, trying to identify CRC subtypes that are distinguished among TME and drug-response sensitivity, and helpfully, contributing to the precise treatment of CRC.

**Fig. 6:**
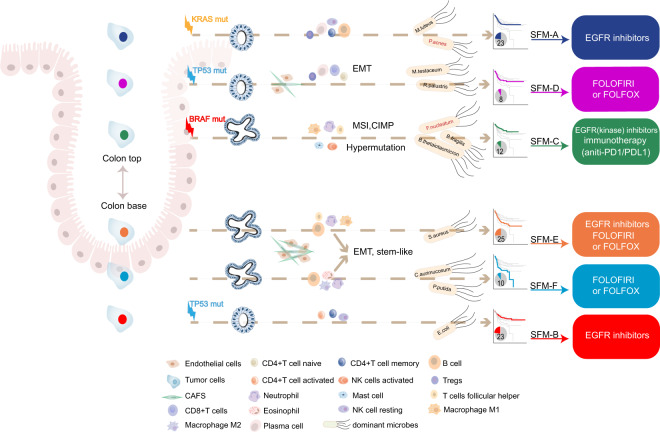
Summary of characteristics of SFM subtypes. These included pathological features, genomic markers, TME, epigenetic changes, gut microbes, prognostic value, and possible SFM subtypes-guided therapies for CRC.

We firstly built a SFM gene signature that not only could discriminate TME, but also was associated with FOLFIRI resistance. Although the importance of TME has been addressed, none published gene signatures for CRC classification focused on either TME-associated genes or combination of TME and cytotoxic treatment. We found that SFM signature had limited overlap with previous gene signatures, while SFM subtypes displayed large overlaps with these gene signature-derived subtypes. The potential reason might be that the SFM signature were derived from (i) transcription of sorted cell populations which were more discriminative for TME than those signatures; and (ii) transcription associated FOLFIRI response. This indicated that SFM was unique and specific which encouraged us to test if SFM could help to identify CRC subtypes. The large overlaps between SFM subtypes and previous reported molecular subtypes might because that since these molecular subtypes were focused on CRC using the same datasets, the main potential subtypes based on transcription pattern would not change too much, such as stromal predominate subtype. Indeed, the SFM subtype was well-captured of the transcription signals derived from TME which made it possibly comparable with other subtypes even though it was limitedly overlapped with other gene signatures. Future investigation is needed to confirm the specific biological functions of SFM genes. We chose *K*-means to perform the classification because it is a straightforward clustering algorithm, which can compute faster than hierarchical clustering when there are numbers of variables. In addition, *K*-means is easy to produce tighter clusters than hierarchical clustering^39^. Finally, we identified six SFM-based subtypes with distinctive molecular and clinical relevance (Fig. 6).

Specifically, we identified the SFM-C subtype that was highly enriched for MSI tumors with BRAF mutation, CIMP+, hypermutation, and proximal colon phenotypes. This was in line with previous report about the association among these characteristics^40^. On the contrary, tumors classified as SFM-A-B-D-E showed CIN+, MSS, and non-hypermutant features. We found mutation status of 53 CRC driver genes were significant different among SFM subtypes, which might reflect CRC intrinsic traits. Moreover, the association between previous existed gene signatures with SFM subtypes also uncovered underlying biological traits behind SFM subtypes. For instance, we observed significant association of serrated precursor neoplasia with SFM-C-E-F. This was consistent with previous report that sessile serrated polyps correlated with MSI tumors^41^. As SFM-E and SFM-F were not enriched for MSI tumors, we speculated that these two subtypes were associated with traditional serrated polyps^41^. Further pathological investigations about these correlations would be acquired. In terms of cell origins, our findings also indicated that SFM-B-E-F were derived from colon crypt base as these subtypes harbored hallmarks of colon crypt base cells. This means these subtypes might capture distinct colonic epithelial cell differentiation-associated pathways compared to other SFM subtypes. In terms of SFM-E and SFM-F, we saw significant enrichment for EMT and stem-like features. Our survival analysis indicated that these two subtypes had shorter DFS compared to the rest SFM subtypes. This was in line with previous investigations that stem/serrated/mesenchymal CRC subtype had poor prognosis^16,17^. In addition, large overlaps of high-risk cases between Oncotype DX and SFM subtypes also implied the prognostic value of SFM subtypes. To compare the prognostic value of previous CRC classifiers, we performed univariate and multivariate Cox regression analysis. Among these nine classifiers, including AJCC stage and SFM subtypes, seven of them had sub-classifiers that were significant in the univariate regression model. However, when adjusted by age, gender, and AJCC stage, only SFM, CMS, and CCS subtypes were still significant which meant that these three molecular classifiers had strong prognostic value in CRC.

The heterogeneity of CRC might also determine the sensitivity of chemotherapy. Sadanandam et al. have reported that stem-like subtype tumors responded to FOLFIRI^14^. This was consisted with our results that SFM-E and SFM-F were enriched for stem-like tumors and responsive to FOLFIRI. Given that we have shown that SFM-E and -F generally had shorter survival than the remaining subtypes, our analysis on combined datasets (GSE104645 and GSE72970) indicated that SFM-A/B had worse survival after treated with FOLFIRI or FOLFOX regimens, while SFM-D/E/F have improved their survival. This means that FOLFIRI or FOLFOX might be more suitable for SFM-D, -E, and -F subtypes. Moreover, although SFM-C displayed nonresponsive to FOLFIRI and FOLFOX, which was also consistent with previous studies suggesting MSI tumors barely responded to fluorouracil-based therapy^42^, it had better survival in this combined dataset probably because of the enrichment of MSI phenotype, which has been proofed to have relative better survival^43^. Regardless of SFM subtypes, the response rate of FOLFIRI and FOLFOX were 48% and 47%, respectively in the aggregated datasets (FDR < 0.2). This was consistent with the previous report that nearly half of CRC were responsive to these two treatments^38^. In addition, SFM-A and SFM-B displayed high sensitivity of VEGF/EGFR inhibitors. Importantly, as for cetuximab, our results suggested that these subtypes were enriched for overexpression of ERBB3, EREG, BTC, and AREG. These biomarkers have been proved to be involved in EGFR-associated pathways blockade^31–33^. Although the sensitivity of most samples for gefitinib and vandetanib could not be accurately evaluated (FDR < 0.2) across SFM subtypes, we saw SFM-C subtype was responsive to these two inhibitors. FOLFIRI or FOLFOX have been approved to be used as the standard chemotherapy treatments plus avastin or cetuximab, while our results indicated that not all CRCs are suitable for these combined regimens.

Moreover, increasing evidence suggested that MSI tumors were responsive to immunotherapy of anti-PD1/PDL1^9,10^. SFM-C displayed higher mutation burden with MSI phenotype. Our results also indicated that SFM-C and SFM-F were immune suppressive as these two subtypes had higher proportions of exhausted T cells and MDSCs and IFN-γresponse rate. Both of them were highly expressed of CD274 and CTLA4. Since SFM-F exhibited higher IPRES score, which meant that SFM-F had features of “nonresponder” of immunotherapy. These results indicated that both of MSI and high stroma/EMT microenvironment could lead to immune suppression, while MSI-induced immune suppression could easily be blocked by checkpoint inhibitors, but not for stroma/EMT-induced immune suppression. This might be reason why part of patients with high checkpoint markers did not response to checkpoint inhibitors.

So far, the association of CRC molecular subtypes with gut microbiome has not been clearly elucidated. The first time to describe the association of bacterial signatures with CRC molecular subtypes was reported by Burns et al.^44^. We found distinctive bacterial communities across SFM subtypes by mapping TCGA CRC nonhuman RNA-sequencing reads to bacterial reference sequences. Notably, SFM-C with MSI tumor showed high relative abundance of *F. nucleatum*, which has been proved to be associated with CRC development and progression^45,46^. *F. nucleatum* also has strong association with immune response of CRC, particularly by recruiting T cells. Our results were also in line with previous reports that *F. nucleatum* was associated with CIMP+, MSI CRC subtype^47^, both of which were features of SFM-C. *E. coli* was significantly enriched in SFM-B. *E. coli* has been regarded as commensal bacterial in gut microbiota, but some of its stains might also contribute to tumor development by inducing chronic inflammation or producing toxins^48^. *P. acnes*, enriched for SFM-A has shown association with prostate cancer^49^. *P. acnes* can induce prostatic inflammation in prostate cancer glands. As the biological functions of these distinctive gut microbes have not been clearly elucidated, the gap between these gut microbes and CRC should be bridged by performing more experimental studies and clinical trials.

The limitations should be acknowledged for this research. Firstly, this study is retrospective, the SFM subtypes classification should be further identified in large prospective clinical trials. Secondly, when performing multivariate Cox regression analysis, not all of the clinical factors were included, such as tumor grade and number of positive nodes as these information was not publicly available. Thus, SFM subtypes as independent prognostic factor needs to be further confirmed. Thirdly, we predicted drug response using previous gene signatures, since these information are not available in the included datasets. Therefore, drug sensitivity among SFM subtypes should be confirmed using more comprehensive data in the future. Fourthly, the SFM gene signature was initially correlated with FOLFIRI response, while we saw SFM subtypes can also predict EGFR inhibitors response, which could not be well interpreted based on our current analysis.

In conclusion, we build a new classifier of CRC into six molecular subtypes, using SFM signature that can discriminate TME and is associated with drug response. This gene signature can partially explain the heterogeneity of CRC. The SFM subtypes would help to improve precision treatment of CRC.

## Methods

### CRC data collection and candidate gene selection

The publicly available datasets used in this study were accessed from the Gene Expression Omnibus (GEO) and TCGA. Among these datasets, three datasets (GSE39395, GSE39396, and GSE62080) were used for SFM signature construction. Four datasets were used for CRC classification using SFM, including GSE39582, five GEO batch, TCGA, and Renji cohort. Five GEO batch consisted of five GEO datasets, including GSE14333, GSE17536, GSE17537, GSE33113, and GSE37892. Four scRNA-seq data were used for SFM validation, including GSE81861 for CRC, GSE103322 for HNSCC, GSE72056 for melanoma, and GSE75688 for BRCA. In addition, some aggregated datasets were used for drug-response exploration, including (i) combined GSE19860, GSE28702, and GSE69657 for FOLFOX response; (ii) GSE104645 and GSE72970 for FOLFOX or FOLFIRI response; and (iii) GSE5851 and PRJEB34338 for cetuximab response.

For GEO datasets, we first performed the robust multi-array average method within “affy” R package to normalize each dataset^50^. Three datasets named GSE39395, GSE39396, and GSE62080 were then used to do differential expression analysis to construct a SFM signature, in which genes could discriminate TME and were associated FOLFIRI sensitivity. In GSE39395 and GSE39396 (ref. ^51^), FACS was used to separate cell subpopulations from eight and six samples, respectively (CD45^+^Epcam^−^ for immune cells, CD45^−^Epcam^+^ for tumor epithelial cells and CD45^−^Epcam^−^ for stomal cells in GSE39395; CD45^+^EPCAM^−^CD31^−^FAP^−^ for immune cells, CD45^−^EPCAM^+^CD31^−^FAP^−^ tumor epithelial cells, CD45^−^EPCAM^−^CD31^+^FAP^−^ endothelial cells, and CD45^−^EPCAM^−^CD31^−^FAP^+^ for CAFs in GSE39396). GSE62080 contained transcriptomic data from nine FOLFIRI responders and 12 nonresponders^52^. We did differential expression analysis using limma R package^53^ between each two of the cell populations in GSE39395 and GSE39396, respectively. For instance, in GSE39395, there were three cell populations, including immune cells, tumor epithelial cells, and stomal cells, we thus did differential expression analysis for three times, including immune cells versus tumor epithelial cells, immune cells versus stromal cells, and tumor epithelial cells versus stromal cells. Similar strategies were applied to GSE39396. As for GSE62080, differential expression analysis was directly performed between FOLFIRI responders and nonresponders. Each time for differential expression analysis, candidate genes were identified as differentially expressed when *P* value was <0.05 and |logFC| ≥ 1. Further, we selected out differentially expressed genes within each of these three datasets. Finally, differential expressed genes among these three datasets were overlapped, and further termed as SFM gene signature, as shown in Fig. 1a.

GSE39582 consisted of 566 CRC samples was used as a discovery dataset^23^. To construct a large dataset for validation, we combined five GEO datasets as a unit (GSE14333, GSE17536, GSE17537, GSE33113, and GSE37892), referred to “five GEO batch” dataset^54–57^. Samples that overlapped between GSE14333 and GSE17536 were excluded from GSE14333. The ComBat method within “sva” R package was used for batch correction^58^. By doing so, the first validation dataset (five GEO batch) were composed of 609 CRC cases. In addition, we directly downloaded 577 TCGA CRC gene expression profiles from the synapse repository of CRC provided by the CRCSC^15^. This level 3 TCGA data was used as the second validation dataset. As for drug-associated datasets, we selected three GEO datasets where samples were treated with FOLFOX: GSE19860, GSE28702, and GSE69657, and combined these three datasets together after batch correction^59,60^. This constructed a gene expression profile derived from 78 nonresponders and 64 responders of FOLFOX. In addition, another two datasets in which samples were treated with FOLFIRI or FOLFOX: GSE104645 and GSE72970 were also combined after batch correction^61,62^. Finally, two datasets, GSE5851 and PRJEB34338 where samples were treated with EGFR inhibitor, cetuximab, were also combined after batch correction^32,34^. We also downloaded four scRNA-seq datasets for validation, including GSE81861 for CRC^63^, GSE103322 for HNSCC^64^, GSE72056 for melanoma^65^, and GSE75688 for BRCA^66^. The annotated cell types information are available from corresponding original papers. To account for the influences of technical noise, we firstly performed missing data imputation and data normalization to gene expression profiles. ScImpute algorithm was used to impute missing gene expression with default parameters and TPM, or raw counts value and gene lengths^67^. We used “scater” R packages to normalize imputed raw counts^68^. As for HNSCC and melanoma datasets which had far enough cells, tumors, and nonmalignant cells types containing <50 cells were excluded for further analysis.

Another total of 53 CRC samples were collected from Renji hospital. The study protocol was approved by the ethics committee of Shanghai Jiao Tong University School of Medicine. Written informed consent was obtained from all patients. All samples were sequenced on an Illumina HiSeq 4000 for 2 × 150-bp paired-end sequencing. Reads were mapped to the human genome (GRCh38) using HISAT2 v2.10 (https://ccb.jhu.edu/software/hisat2/), with the default options^69^. Count files of the aligned sequencing reads were generated by the featurecount using the Gencode version 22 gtf file (https://www.gencodegenes.org/human/)^70^. The read counts from each sequenced sample were combined into a count file, which was subsequently used for the downstream expression analysis.

Clinical data were directly downloaded from corresponding GEO website or supplementary materials from associated literatures. Clinical information for TCGA CRCs was downloaded from CRCSC in synapse database and immune associated features were downloaded from a recently public research^71^.

### Identification of SFM subtypes using *K*-means clustering algorithm

Based on the SFM, the identification of SFM subtypes was first performed in discovery dataset (GSE39582) by applying *K*-means clustering algorithm implemented in “factoextra” R package.

We identified the optimal number of clusters by gap statistics within the predetermined number of clusters (*k*) varying from 3 to 8. Among these clusters, *k* = 6 was selected with the best statistic in the discovery dataset. Then we evaluated the similarity and expression differences among the SFM subtypes with the cluster dendrogram and heatmap of the SFM, respectively. To validate the robustness of SFM subtypes, we further performed the same analysis in validation datasets (five GEO batch, TCGA, and Renji cohort).

We used *K*-means clustering algorithm to do the clustering since it is one of the simple and important clustering approach and statistically deterministic without specifying initial centers^72,73^. It is an easier way to classify dataset assuming *k* clusters. One of the advantages of *K*-means algorithm is its higher computational speed for large variable when the number of clusters is relative small. We applied *K*-means clustering implemented in “factoextra” R package to gene expression profiles based on SFM signature comprised of 250 unique genes. Several aspects were considered to determine to cluster assignment in each dataset: (i) gap statistics were reported for each cluster which compared the total within intra-cluster variation for different values of *k* with their expected values under null reference distribution of the data^74^. The estimate of the optimal clusters will be the value that maximize the gap statistics which means that the clustering structure is far away from the random uniform distribution of points. Given that *K*-means clustering requires to pre-specify the number of clusters, we set number of clusters varying from *k* = 3 to 8. Generally, the output of clustering can be visualized using “fvix_gap_stat” function in “factoextra” R package which can suggest the optimal number of clusters marked as vertical dashed line. (ii) For a dataset that the optimal number of clusters were not given by the function itself among *k* = 3 to *k* = 8, we visualized the dendrogram of the clustering and drew the heatmap showing the expression of SFM signature to facilitate the selection of number of clusters. We further selected out the number of clusters that (1) the height of dendrogram were good enough to discriminate amongst clusters as indicated by a red horizontal dashed line; (2) the gene expression profiles in the heatmap showed part of SFM genes that were discriminative amongst clusters, which might be subjective at this stage; and (3) additionally, as for a dataset with small number of sample size, we generally selected smaller number of clusters that also satisfied the principle above.

### Enriched functions and pathways of SFM subtypes

To find to dysregulated signaling pathways among the SFM subtypes, we first did differential expression analysis in each SFM subtype versus the rest in discovery cohort and selected 2000 top up- and down-expressed genes for further analysis in each SFM subtype^23^. These genes were then applied in ClueGO and CluePedia apps^75^. These two plug-ins of Cytoscape are open-source Java tools that can extract the non-redendant biological information for a set of genes. In this study, we performed Ontology/pathway analysis, including Gene Oncology (GO, BP, CC, MF, and immune system process) and Kyoto Encyclopedia of Genes and Genomes (KEGG) in Cytoscape 3.5.0 software.

### NTP implementation and signature adaptation

NTP-based classification^25^ was performed on GenePattern (https://www.genepattern.org/). NTP classification allows us to apply given signatures to individual cases wherever these gene signatures are derived from. Generally, these gene signatures consist of upregulated and downregulated genes to form a binary reference gene expression. NTP applies a nearest neighbor method to calculate the similarity of gene expression profile to a reference gene expression signature in each case. Then a null distribution of similarity coefficients would be assessed by randomly sub-sampling the gene space. Finally, a *P* value would be calculated when comparing the similarity coefficient derived from the given gene signature with the null distribution. The threshold selected for significance of each case was Benjamini–Hochberg-corrected FDR < 0.2 (ref. ^14^).

We evaluated the association of SFM subtypes with a set of gene signatures (Supplementary Table 5). The lists of gene signatures derived from previously published papers are as following: intestinal stem cell signature^76^, colon crypt signature^77^, serrated CRC signature^27^, EMT signature^78^, FOLFIRI response signature^79^, and FOLFOX^29^ response signature and VEFG/EGFR inhibitors signatures described by Schutte et al.^80^, including avastin, cetuximab, afatinib, sapitinib, gefitinib, and vandetanib.

### Cells infiltration estimation

We used CIBERSORT algorithm to estimate the immune cell infiltration in CRCs samples. This method used cell-specific gene signatures to discriminate a total of 22 immune cell populations as described by Newman et al.^81^. We additionally used microenvironment cell population (MCP)-counter algorithm to estimate the proportions of stroma and endothelial cells. This method can robustly quantify the abundance of various immune and stromal cell populations based on transcriptomic data for each sample^82^. The output of MCP-counter can be used to estimate the relative infiltration of endothelial cells, fibroblasts, and another eight immune cells populations. We performed MCP-counter analysis using “MCPcounter” R package. Stromal fraction was estimated using “estimate” R package.

### Survival analysis

DFS and OS were regarded as the end points upon the clinical information available in the datasets (RFS in GSE39582 and five GEO batch; RFS/OS in TCGA). Survival analysis was performed based on the Kaplan–Meier algorithm. The *P* value for the differences between SFM subtypes was calculated using log-rank test. Univariate and multivariate Cox models were constructed by cox proportion hazards regression. These analyses were implemented in “survival” and “survminer” R packages.

### Single sample gene set enrichment analysis

Gene set variation analysis (GSVA) is a nonparametric and unsupervised method that can be used to evaluate gene set enrichment based on gene expression profiles derived from microarrays or RNA-seq data^83^. GSVA can evaluate the given pathway activity variation by transforming the gene by sample matrix into a gene set by sample matrix. Therefore, it can easily assess a pathway enrichment for individual case. Importantly, the GSVA also provide a method called “single sample gene set enrichment analysis (ssGSEA)”, which can compute a gene set enrichment score per sample as the normalized difference in empirical cumulative distribution functions of gene expression ranks inside and outside a given gene set. Single sample gene set enrichment analysis (ssGSEA) was firstly described by Berbie et al.^84^. In this study, we performed ssGSEA implemented in “GSVA” R package and evaluated the EGFR gene set activity across the SFM subtypes in three datasets (GSE39582, five GEO batch and TCGA and Renji cohort). The EGFR gene set consisted of EGFR pathways-associated ligands or receptors, including EGFR, ERBB3, EREG, BTC, HBEGF, AREG, and IRS2 as previous papers reported^31–33^. Besides, we also performed similar analysis for TGF-beta response^85^, exhausted T cells^86^, MDSCs^86^, hot tumor^87^, IFN-gamma response^88^, IPRES signatures^89^, and SFM gene signature in four single-cell datasets.

### Oncotype DX

The 12-mRNA-based Oncotype DX colon cancer recurrence score assay was built based on transcriptomic data from 1851 cases with stage II and III colon cancer^90^. It has been recognized as an independent prognostic factor in CRC. To confirm the prognostic value of SFM subtypes, we proposed to associate the SFM subtypes, Oncotype DX with DFS in univariate and multivariate Cox regression models. To this end, we first reproduced the Oncotype DX calculation in three datasets as described by Clark-Langone et al.^36^. Cases with recurrence score (RS) < 30, 30 ≤ RS ≤ 41, or RS > 40 were regarded as low, intermediate or high risk of recurrence, respectively. The association of Oncotype DX with DFS was confirmed in all cases (*P* < 0.0001, Fig. 4e) or only stage II and III cases (*P* = 0.00018, Fig. 4f).

### Microbial detection using PathSeq algorithm

The PathSeq algorithm described by Kostic et al. can be used to identify microbes according to deep sequencing data from RNA sequencing and WGS in human tissue^91,92^. The human reads would be computationally subtracted by mapping reads to human genome database after low-quality, duplicate, and repetitive sequences were filtered. Then mapped reads would be removed and unmapped reads that belong to nonhuman, pathogen-derived reads would be subjected to further analysis. Followed by the assignment of the unmapped reads to the acknowledged sequenced whole bacterial reference genomes by a metagenomic analysis, these unmapped reads would be taxonomically classified into bacterial, viral, and fungal sequences. The relative abundance value for each organism would be then computed using the reads mapping with >90% sequence identity and >90% query coverage. Finally, the classification was analyzed at the domain, phylum, genus, and species level. Following PathSeq approach, we obtained the relative abundance of 1093 microbes in 429 CRC samples, 415 of which were annotated with SFM subtypes information and analyzed afterwards.

### Statistical analysis

We performed two-tailed Students’s *t* test, Fisher’s exact test, *χ*^2^ test, and Kruskal–Wallis test using R program (v.3.4.1). Cox regression hazard model and Kaplan–Meier analyses were conducted using “survival” and “survminer” R packages, respectively. In all these tests, statistical significance was set at 0.05. In the NTP algorithm, the results were regarded as significant if the Benjamini–Hochberg FDR was <0.2.

**Declarations**

## Supplementary information

supplementary figures and tables

reporting-summary

## Data Availability

The data generated and analyzed during this study are described in the following data record: 10.6084/m9.figshare.13027715^93^. The data analyzed during the study were downloaded from public databases, including Gene Expression Omnibus (GEO; https://www.ncbi.nlm.nih.gov/geo/) and The Cancer Genome Atlas (TCGA; TCGA CRC datasets available from the Synapse repository at: https://www.synapse.org/#!Synapse:syn2623706/files/). For a list of accession IDs for the analyzed data, see Supplementary Table S1. The Renji RNA-seq data is available from GEO: https://identifiers.org/geo:GSE158559 ^94^. All other output data are included in the figshare data record^93^.

## Supplementary information

**Supplementary information** The online version contains supplementary material available at 10.1038/s41698-021-00142-x.

## Code availability

All statistical analyses described in the manuscript were performed using custom software developed in R. Custom code for the analysis is available in at https://github.com/helianthuszhu/SFM.subtype.CRC.
