# Supplementary material for: A tumor microenvironment-specific gene expression signature predicts chemotherapy resistance in colorectal cancer patients
Source: NPJ Precis Oncol. 2021 Feb 12;5:7. doi: 10.1038/s41698-021-00142-x (PMC7881244; doi:10.1038/s41698-021-00142-x)
Supplement: Supplementary file 1 — supplementary figures and tables [file 41698_2021_142_MOESM1_ESM.pdf]

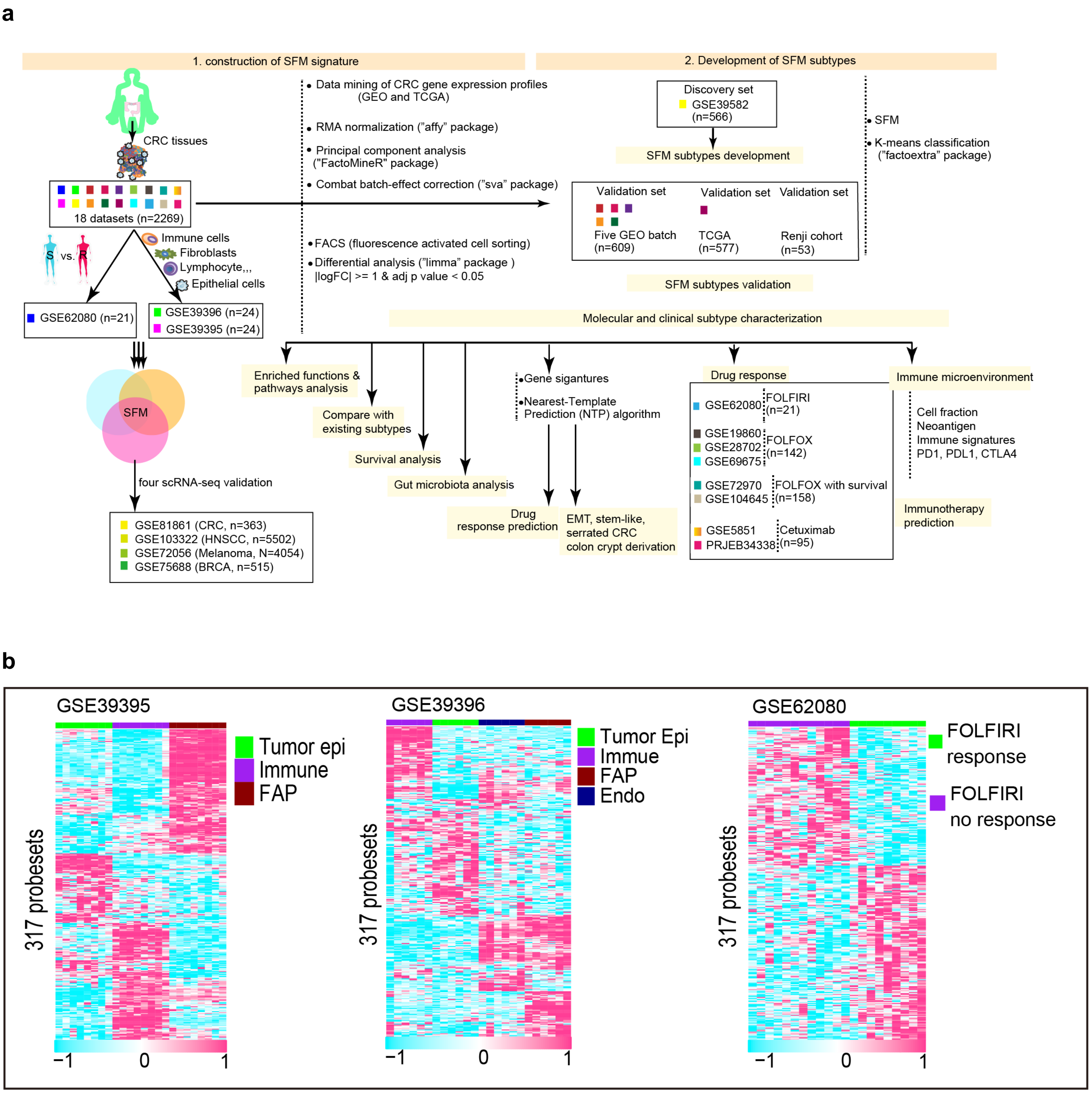
a
1. construction of SFM signature
2. Development of SFM subtypes
CRC tissues
18 datasets (n=2269)
S vs. R
Immune cells
Fibroblasts
Lymphocyte,,,
Epithelial cells
GSE62080 (n=21)
GSE39396 (n=24)
GSE39395 (n=24)
SFM
four scRNA-seq validation
GSE81861 (CRC, n=363)
GSE103322 (HNSCC, n=5502)
GSE72056 (Melanoma, N=4054)
GSE75688 (BRCA, n=515)
Data mining of CRC gene expression profiles (GEO and TCGA)
RMA normalization ("affy" package)
Principal component analysis ("FactoMineR" package)
Combat batch-effect correction ("sva" package)
FACS (fluorescence activated cell sorting)
Differential analysis ("limma" package) |logFC| >= 1 & adj p value < 0.05
Discovery set
GSE39582 (n=566)
SFM subtypes development
Validation set
Five GEO batch (n=609)
Validation set
TCGA (n=577)
Validation set
Renji cohort (n=53)
SFM subtypes validation
SFM
K-means classification ("factoextra" package)
Molecular and clinical subtype characterization
Enriched functions & pathways analysis
Compare with existing subtypes
Survival analysis
Gut microbiota analysis
Gene sigantures
Nearest-Template Prediction (NTP) algorithm
Drug response prediction
EMT, stem-like, serrated CRC colon crypt derivation
Drug response
GSE62080
FOLFIRI (n=21)
GSE19860
GSE28702
GSE69675
FOLFOX (n=142)
GSE72970
GSE104645
FOLFOX with survival (n=158)
GSE5851
PRJEB34338
Cetuximab (n=95)
Immune microenvironment
Cell fraction
Neoantigen
Immune signatures
PD1, PDL1, CTLA4
Immunotherapy prediction
b
GSE39395
Tumor epi
Immune
FAP
317 probesets
−1 0 1
GSE39396
Tumor Epi
Immue
FAP
Endo
317 probesets
−1 0 1
GSE62080
FOLFIRI response
FOLFIRI no response
317 probesets
−1 0 1

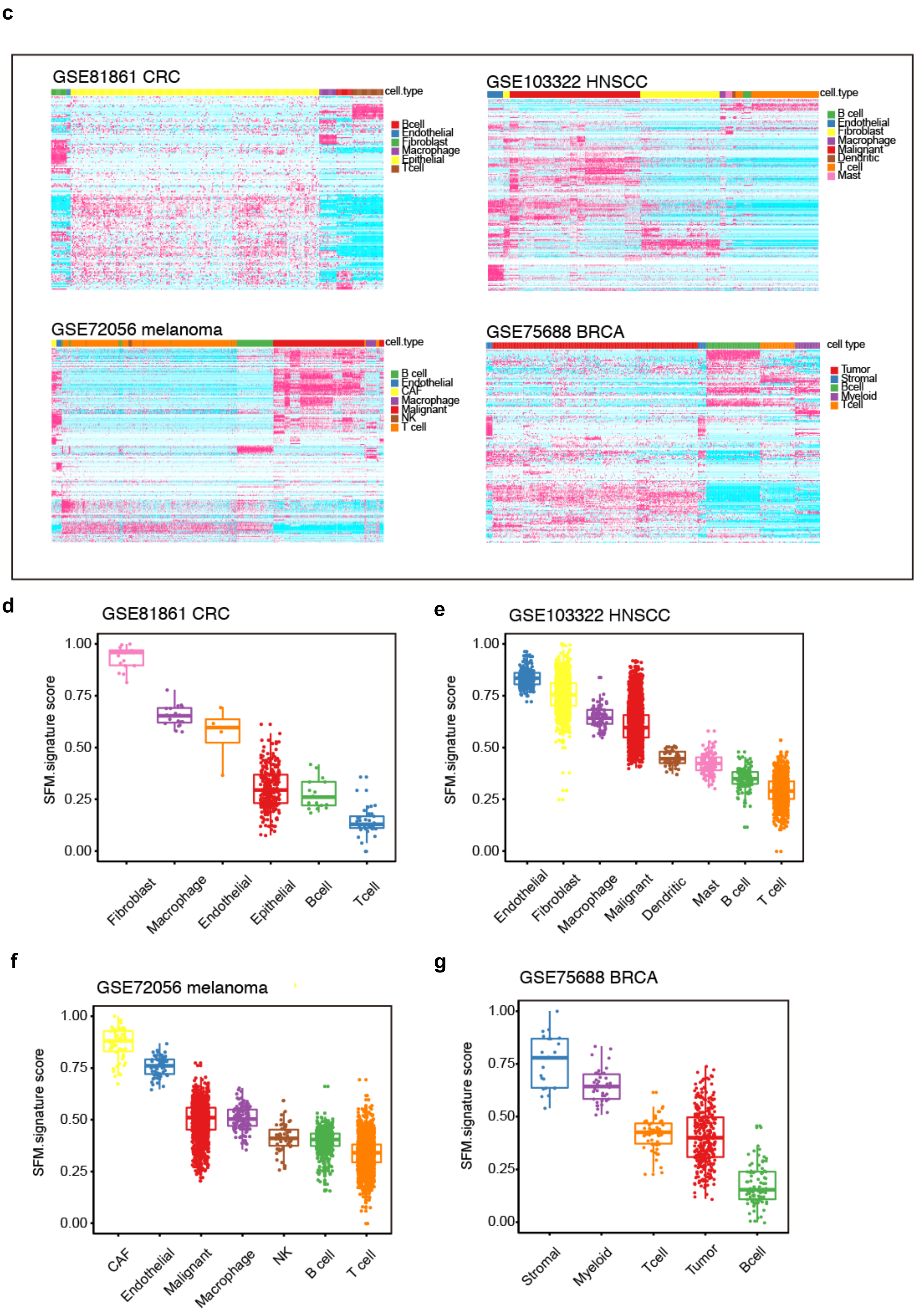
c
GSE81861 CRC
cell.type
Bcell
Endothelial
Fibroblast
Macrophage
Epithelial
Tcell
GSE103322 HNSCC
cell.type
B cell
Endothelial
Fibroblast
Macrophage
Malignant
Dendritic
T cell
Mast
GSE72056 melanoma
cell.type
B cell
Endothelial
CAF
Macrophage
Malignant
NK
T cell
GSE75688 BRCA
cell type
Tumor
Stromal
Bcell
Myeloid
Tcell
d
GSE81861 CRC
SFM.signature score
1.00
0.75
0.50
0.25
0.00
Fibroblast
Macrophage
Endothelial
Epithelial
Bcell
Tcell
e
GSE103322 HNSCC
SFM.signature score
1.00
0.75
0.50
0.25
0.00
Endothelial
Fibroblast
Macrophage
Malignant
Dendritic
Mast
B cell
T cell
f
GSE72056 melanoma
SFM.signature score
1.00
0.75
0.50
0.25
0.00
CAF
Endothelial
Malignant
Macrophage
NK
B cell
T cell
g
GSE75688 BRCA
SFM.signature score
1.00
0.75
0.50
0.25
0.00
Stromal
Myeloid
Tcell
Tumor
Bcell

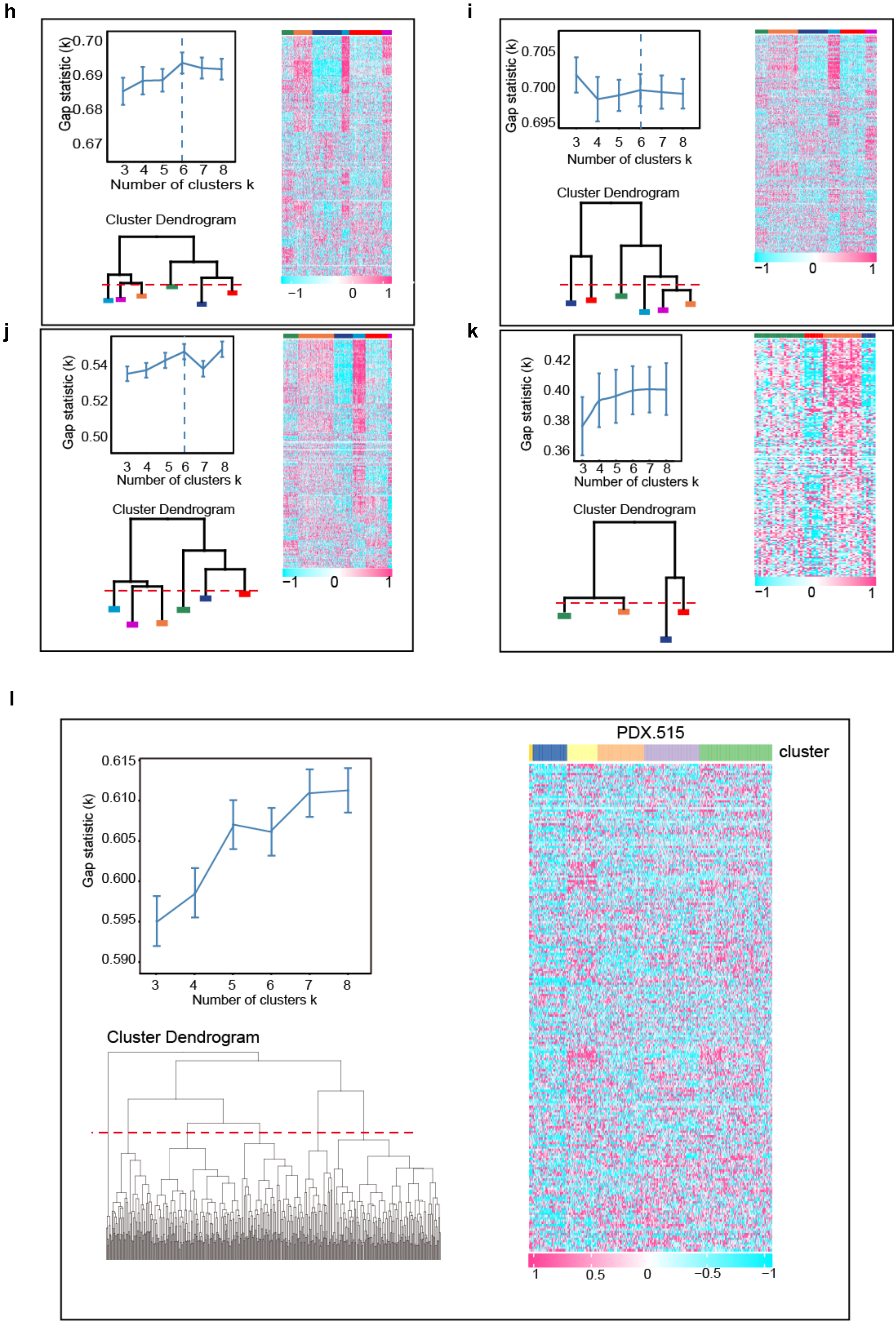

h
Gap statistic (k)
0.70
0.69
0.68
0.67
3 4 5 6 7 8
Number of clusters k
Cluster Dendrogram
−1 0 1
i
Gap statistic (k)
0.705
0.700
0.695
3 4 5 6 7 8
Number of clusters k
Cluster Dendrogram
−1 0 1
j
Gap statistic (k)
0.54
0.52
0.50
3 4 5 6 7 8
Number of clusters k
Cluster Dendrogram
−1 0 1
k
Gap statistic (k)
0.42
0.40
0.38
0.36
3 4 5 6 7 8
Number of clusters k
Cluster Dendrogram
−1 0 1
l
Gap statistic (k)
0.615
0.610
0.605
0.600
0.595
0.590
3 4 5 6 7 8
Number of clusters k
Cluster Dendrogram
PDX.515
cluster
1 0.5 0 −0.5 −1

m

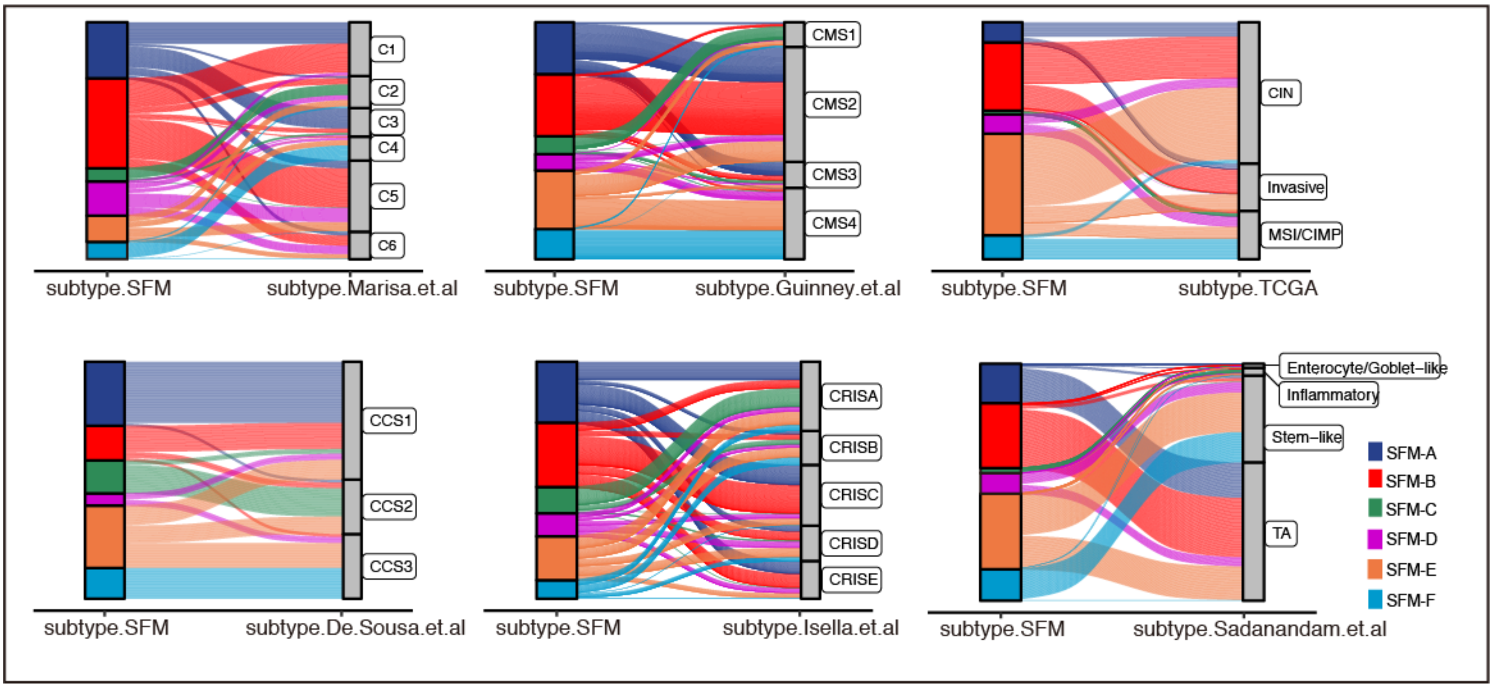

C1
C2
C3
C4
C5
C6
subtype.SFM
subtype.Marisa.et.al
CMS1
CMS2
CMS3
CMS4
subtype.Guinney.et.al
CIN
Invasive
MSI/CIMP
subtype.TCGA
CCS1
CCS2
CCS3
subtype.De.Sousa.et.al
CRISA
CRISB
CRISC
CRISD
CRISE
subtype.Isella.et.al
Enterocyte/Goblet–like
Inflammatory
Stem–like
TA
subtype.Sadanandam.et.al
SFM-A
SFM-B
SFM-C
SFM-D
SFM-E
SFM-F

n

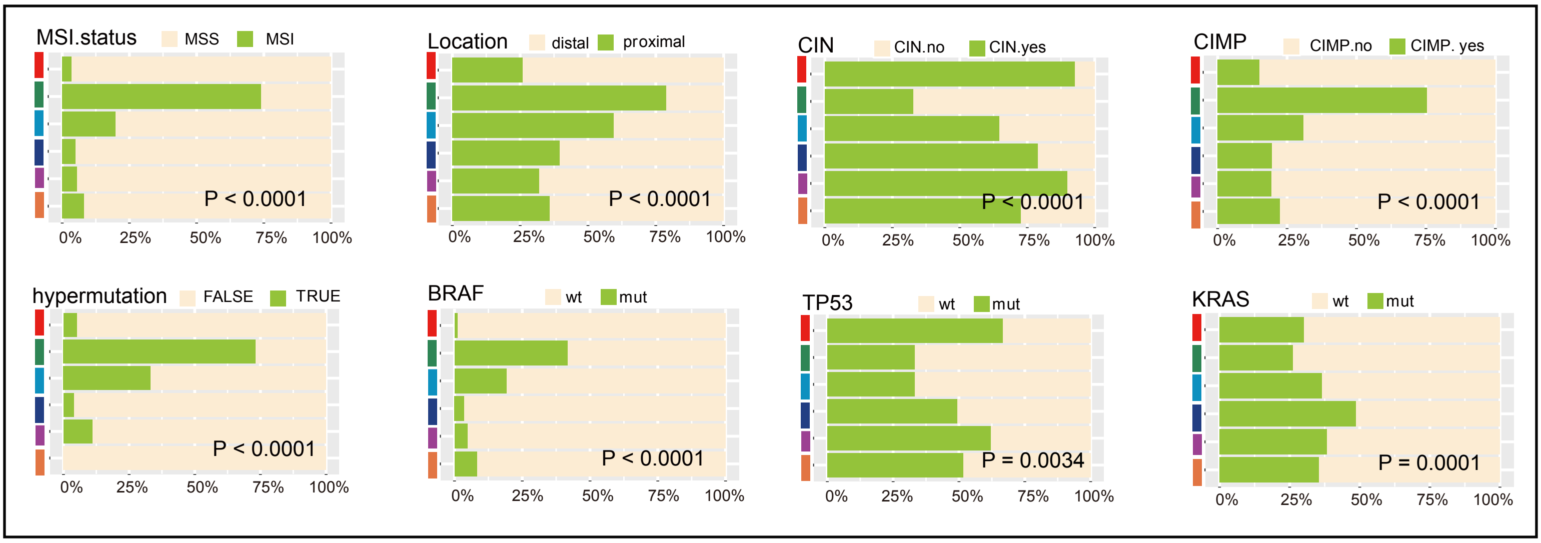

MSI.status MSS MSI
P < 0.0001
Location distal proximal
P < 0.0001
CIN CIN.no CIN.yes
P < 0.0001
CIMP CIMP.no CIMP. yes
P < 0.0001
hypermutation FALSE TRUE
P < 0.0001
BRAF wt mut
P < 0.0001
TP53 wt mut
P = 0.0034
KRAS wt mut
P = 0.0001
0% 25% 50% 75% 100%

o

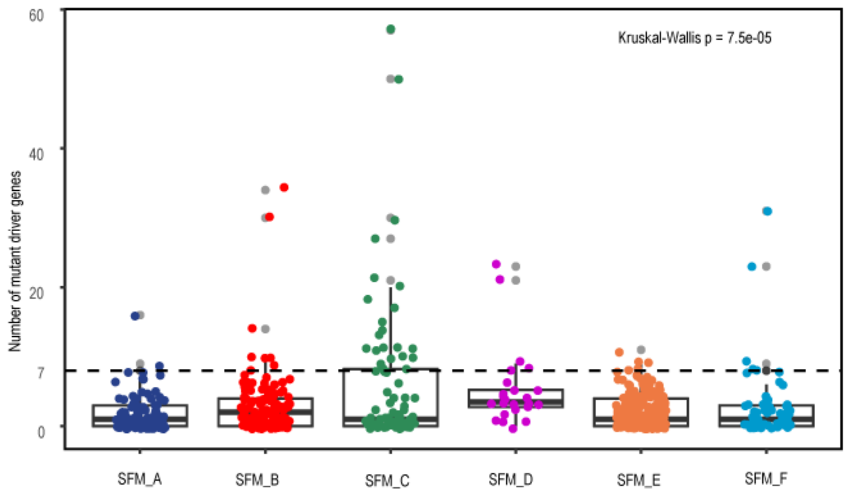

Kruskal-Wallis p = 7.5e-05
Number of mutant driver genes
60
40
20
7
0
SFM_A
SFM_B
SFM_C
SFM_D
SFM_E
SFM_F

p

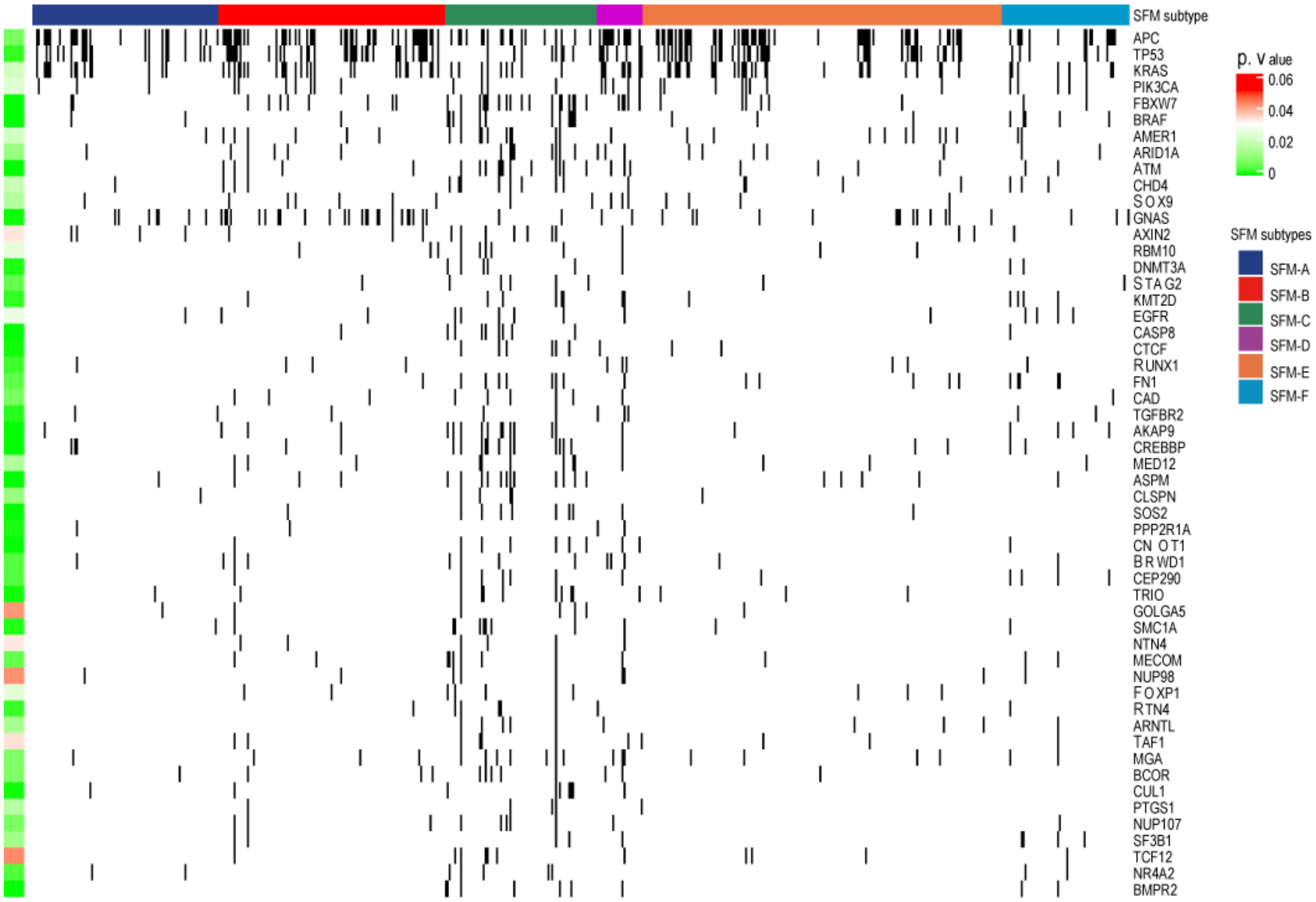

SFM subtype
APC
TP53
KRAS
PIK3CA
FBXW7
BRAF
AMER1
ARID1A
ATM
CHD4
SOX9
GNAS
AXIN2
RBM10
DNMT3A
STAG2
KMT2D
EGFR
CASP8
CTCF
RUNX1
FN1
CAD
TGFBR2
AKAP9
CREBBP
MED12
ASPM
CLSPN
SOS2
PPP2R1A
CNOT1
BRWD1
CEP290
TRIO
GOLGA5
SMC1A
NTN4
MECOM
NUP98
FOXP1
RTN4
ARNTL
TAF1
MGA
BCOR
CUL1
PTGS1
NUP107
SF3B1
TCF12
NR4A2
BMPR2
p. Value
0.06
0.04
0.02
0
SFM subtypes
SFM-A
SFM-B
SFM-C
SFM-D
SFM-E
SFM-F

q

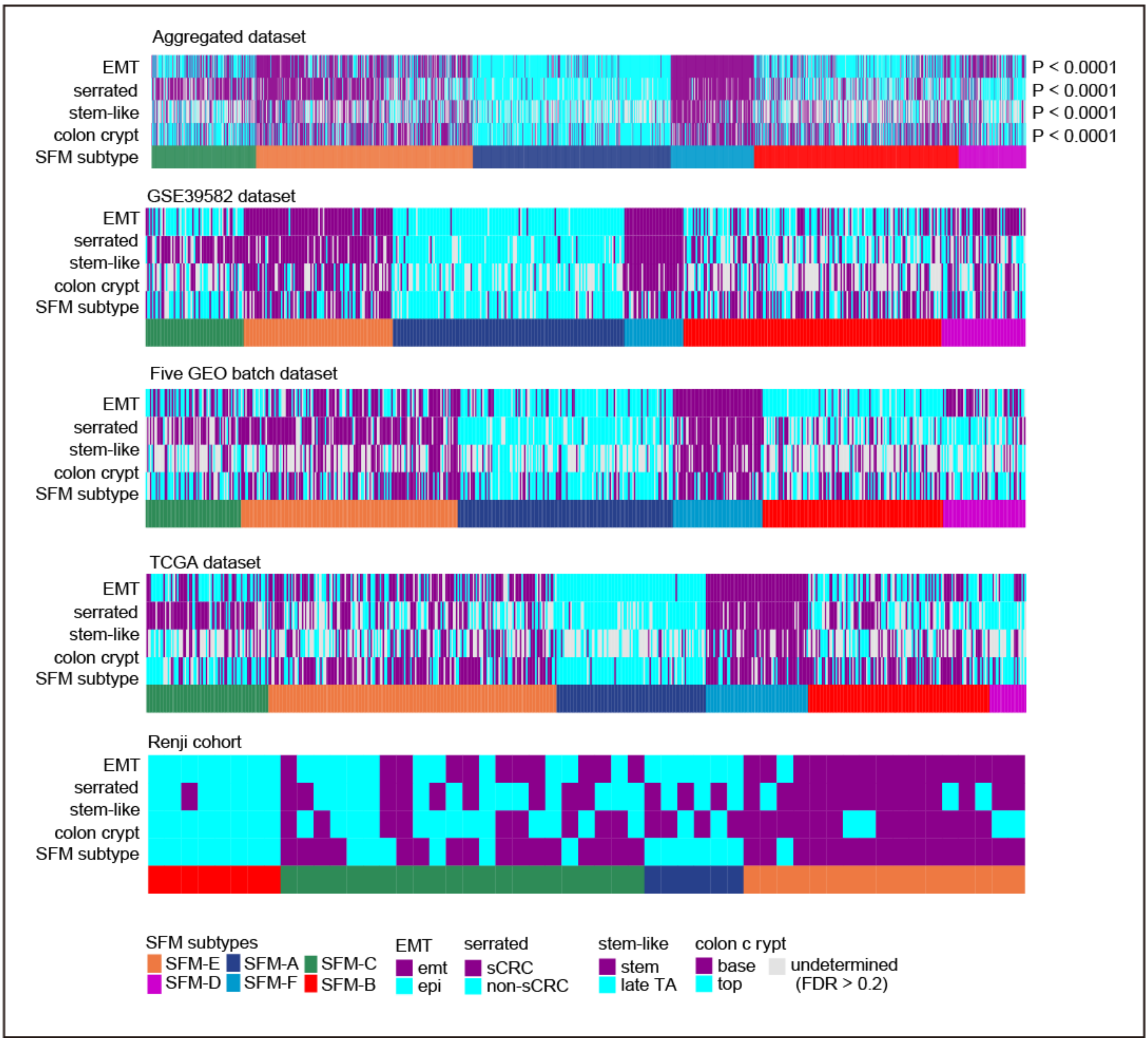
Aggregated dataset
EMT
serrated
stem-like
colon crypt
SFM subtype
P < 0.0001
P < 0.0001
P < 0.0001
P < 0.0001
GSE39582 dataset
Five GEO batch dataset
TCGA dataset
Renji cohort
SFM subtypes
SFM-E
SFM-A
SFM-C
SFM-D
SFM-F
SFM-B
EMT
emt
epi
serrated
sCRC
non-sCRC
stem-like
stem
late TA
colon c rypt
base
top
undetermined
(FDR > 0.2)

r

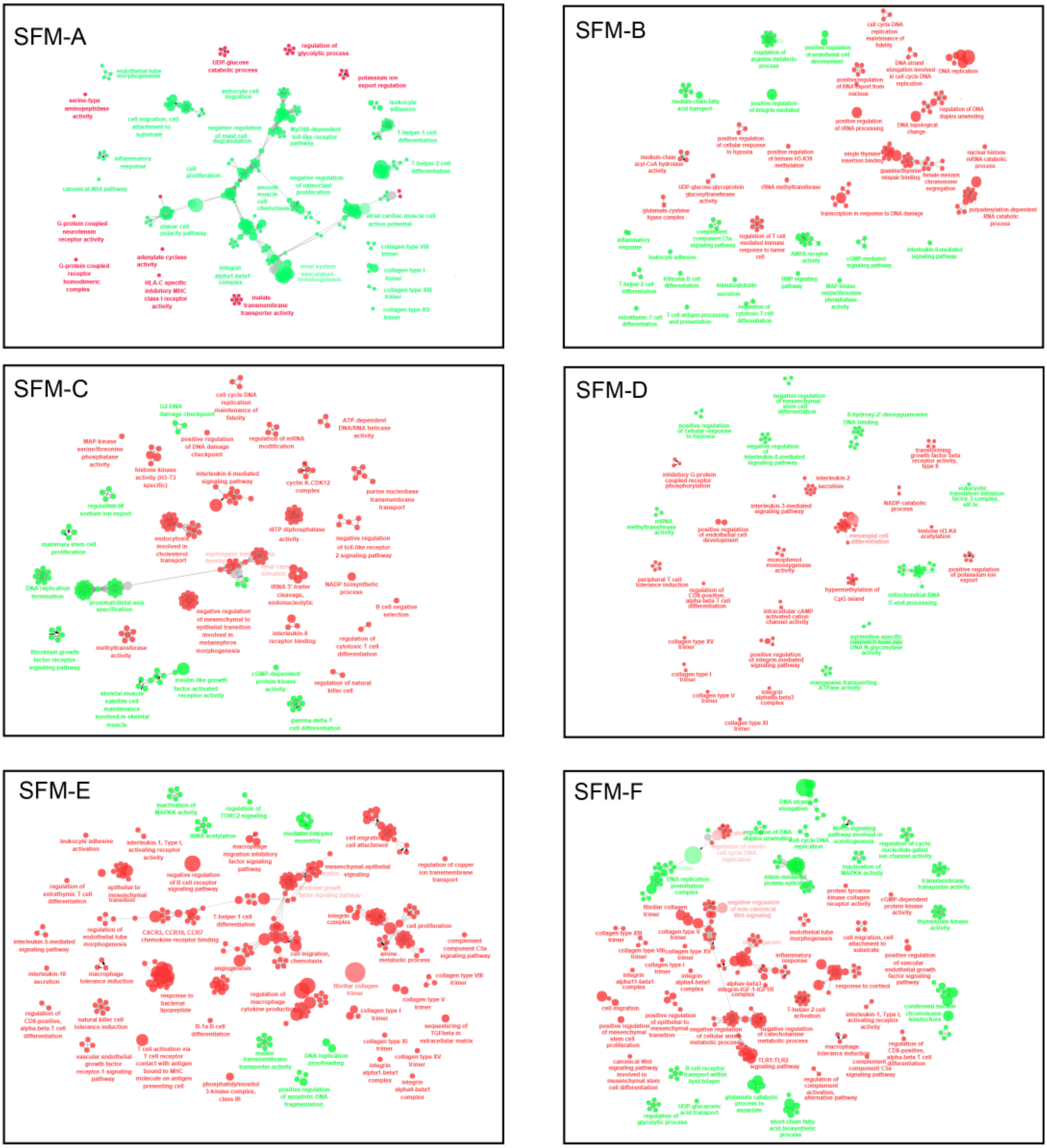

Supplementary Figure 1. Establishment of SFM subtypes and characteristics exploration. (a) Schematic workflow of SFM subtypes construction, validation and further analysis. (b) Heatmaps showing expression of SFM expression in GSE39395, GSE39396 and GSE62080, respectively. (c) Heatmaps showing expression of SFM expression in four scRNA-seq datasets. (d-g) Box plots showing SFM gene signature score based on GSVA among cell types in four scRNA-seq datasets, respectively. (h-l) K-means algorithm for molecular subtype classification in discovery and validation datasets (h, GSE39582; i, five GEO batch; j, TCGA; k, Renji cohort, l, CRC PDX). Gap statistics were plotted from K-means classification within the predetermined number of clusters (k) varying from 3 to 8 using SFM. Unsupervised hierarchical clustering displayed distinct molecular subtypes. Heatmaps showed SFM subtypes defined by SFM. (m) Sankey diagram showing how each SFM subtype contributes to previous CRC classifiers. (n) Distributions of key genomic and epigenomic markers (TP53, BRAF and KRAS, hypermutation, CIN, CIMP, MSI) and anatomical position in aggregated datasets. P value was calculated using Chi-squared test. (o) Box plots of the number of mutant CRC specific driver genes across SFM subtypes in TCGA data. (p) Landscape heatmap showing 53 out of 95 driver mutations that were significant among SFM subtypes. (q) Heatmap of critical pathological factors of SFM subtypes based on published gene signatures using the NTP algorithm. In these analyses, samples with FDR < 0.2 were regarded as significant. (r) Enriched functions and pathways of 2000 top differentially up and down-expressed genes associated with each SFM subtype, separately. The enrichment of KEGG, GO and immune system pathways were test in each subtype. The network of pathways were built using the ClueGO and CluePedia Cytoscappe apps. These pathways are functionally grouped and connected based on the kappa score. For d-g and o, box hinges represent 1st and 3rd quartiles, and middle represents the median. The short horizontal lines represent the standard deviations. The upper and lower whiskers extend from hinges up and down indicate the most extreme values that are within 1.5*IQR (interquartile range) of the respective hinge.

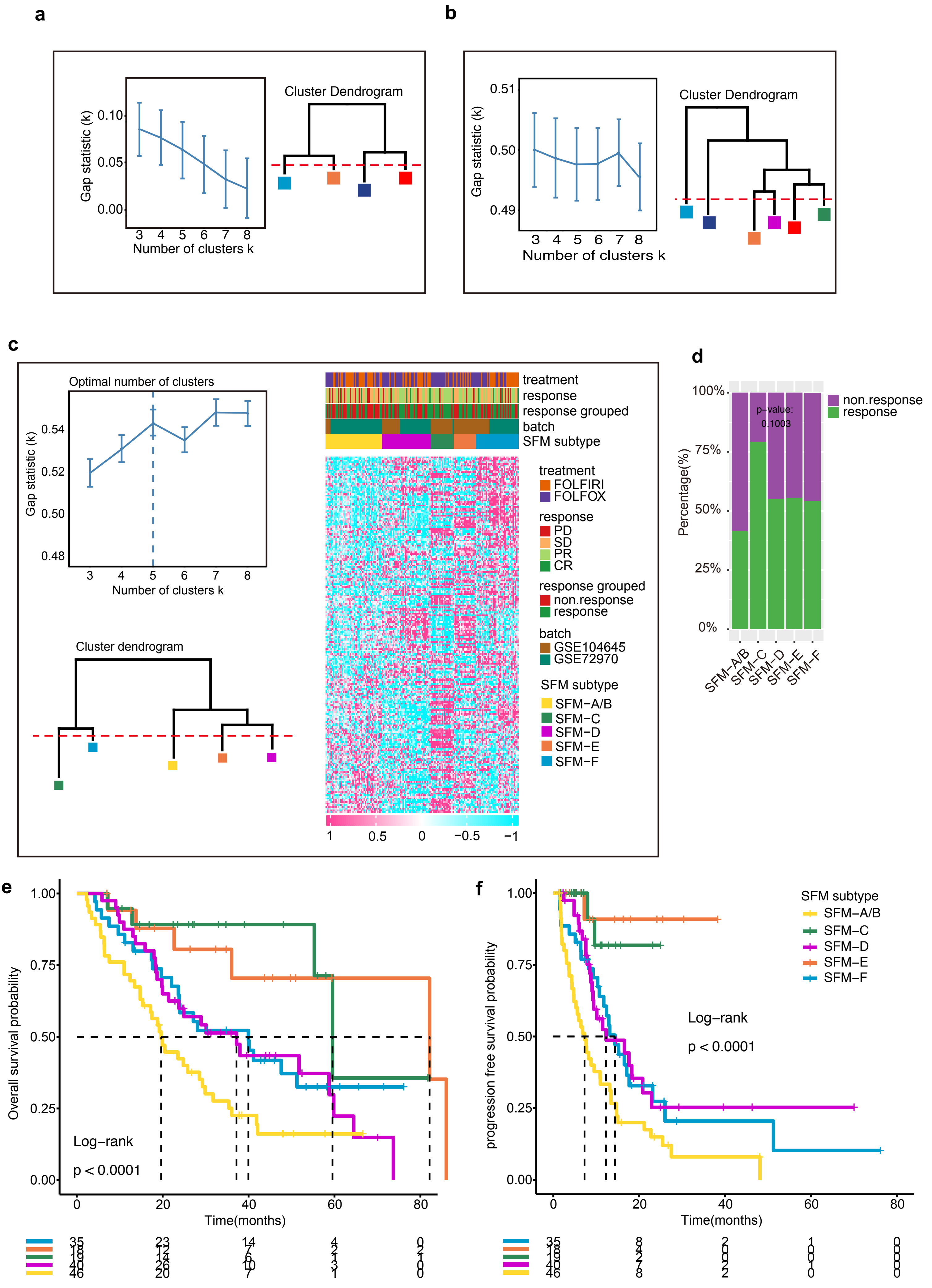
a
b
c
d
e
f
Gap statistic (k)
Number of clusters k
Cluster Dendrogram
Optimal number of clusters
Cluster dendrogram
treatment
response
response grouped
batch
SFM subtype
FOLFIRI
FOLFOX
PD
SD
PR
CR
non.response
response
GSE104645
GSE72970
SFM−A/B
SFM−C
SFM−D
SFM−E
SFM−F
p−value:
0.1003
Percentage(%)
100%
75%
50%
25%
0%
Overall survival probability
progression free survival probability
Time(months)
Log−rank
p < 0.0001
SFM subtype

g

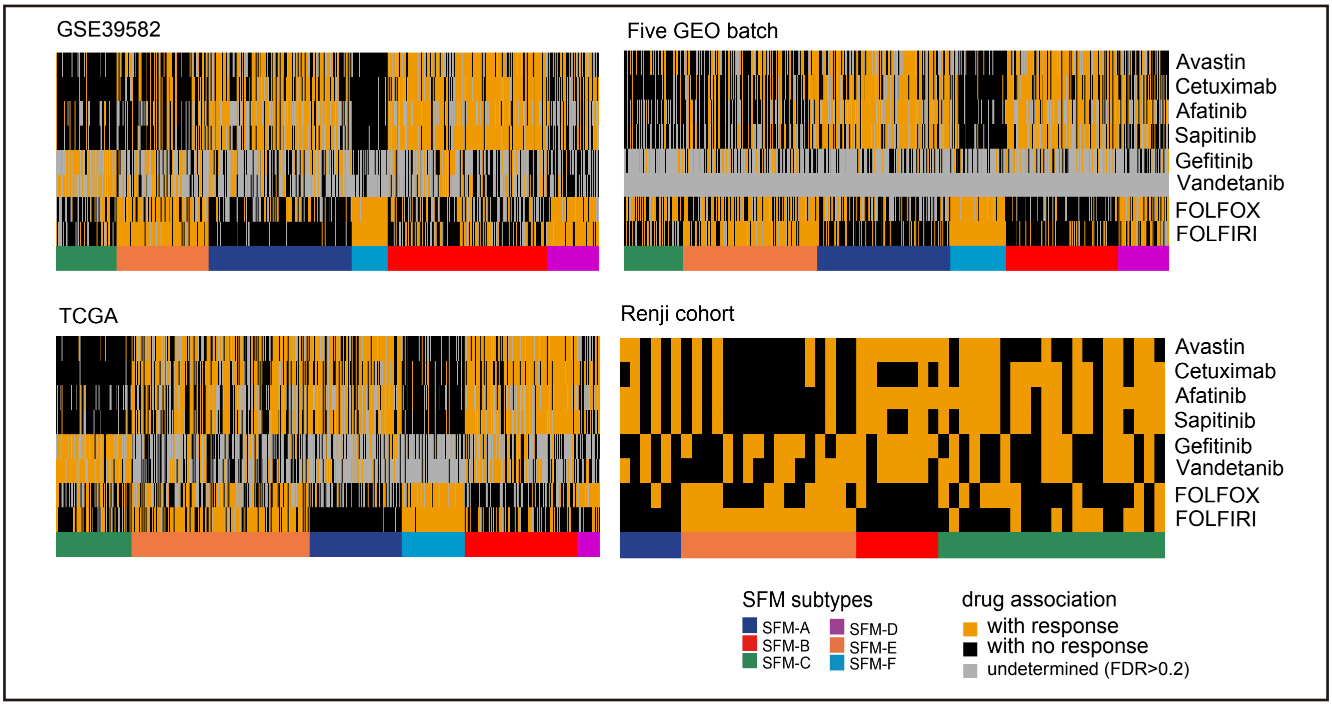
GSE39582
Five GEO batch
TCGA
Renji cohort
Avastin
Cetuximab
Afatinib
Sapitinib
Gefitinib
Vandetanib
FOLFOX
FOLFIRI
SFM subtypes
SFM-A
SFM-B
SFM-C
SFM-D
SFM-E
SFM-F
drug association
with response
with no response
undetermined (FDR>0.2)

h

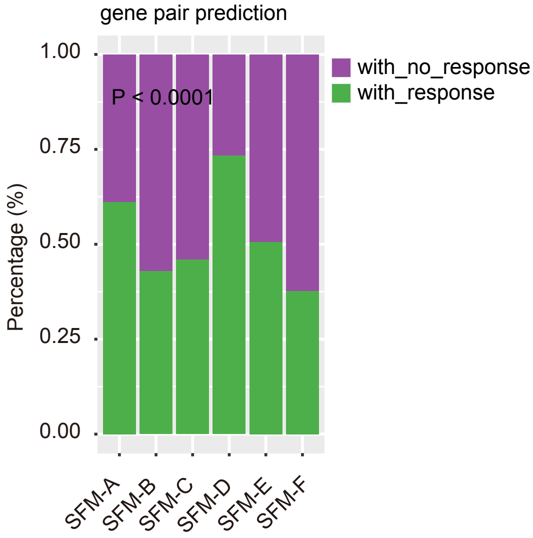
gene pair prediction
1.00
0.75
0.50
0.25
0.00
Percentage (%)
P < 0.0001
with_no_response
with_response
SFM-A
SFM-B
SFM-C
SFM-D
SFM-E
SFM-F

i

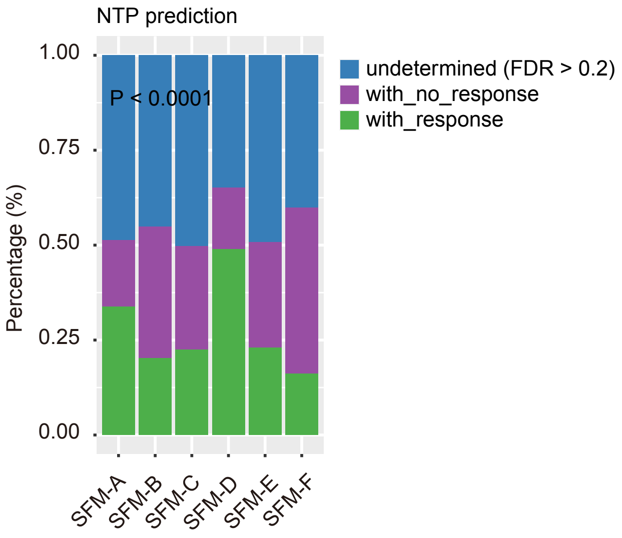
NTP prediction
1.00
0.75
0.50
0.25
0.00
Percentage (%)
P < 0.0001
undetermined (FDR > 0.2)
with_no_response
with_response
SFM-A
SFM-B
SFM-C
SFM-D
SFM-E
SFM-F

j

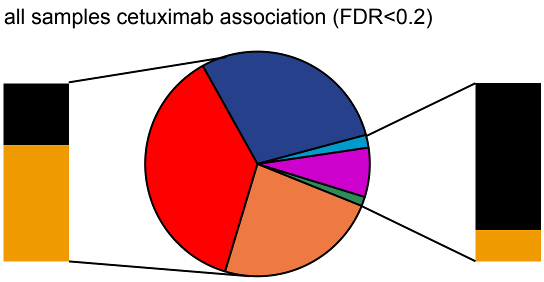
all samples cetuximab association (FDR<0.2)

k

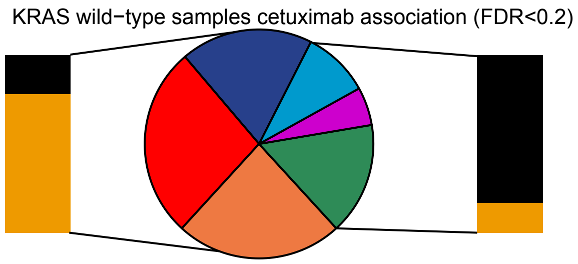
KRAS wild−type samples cetuximab association (FDR<0.2)

l

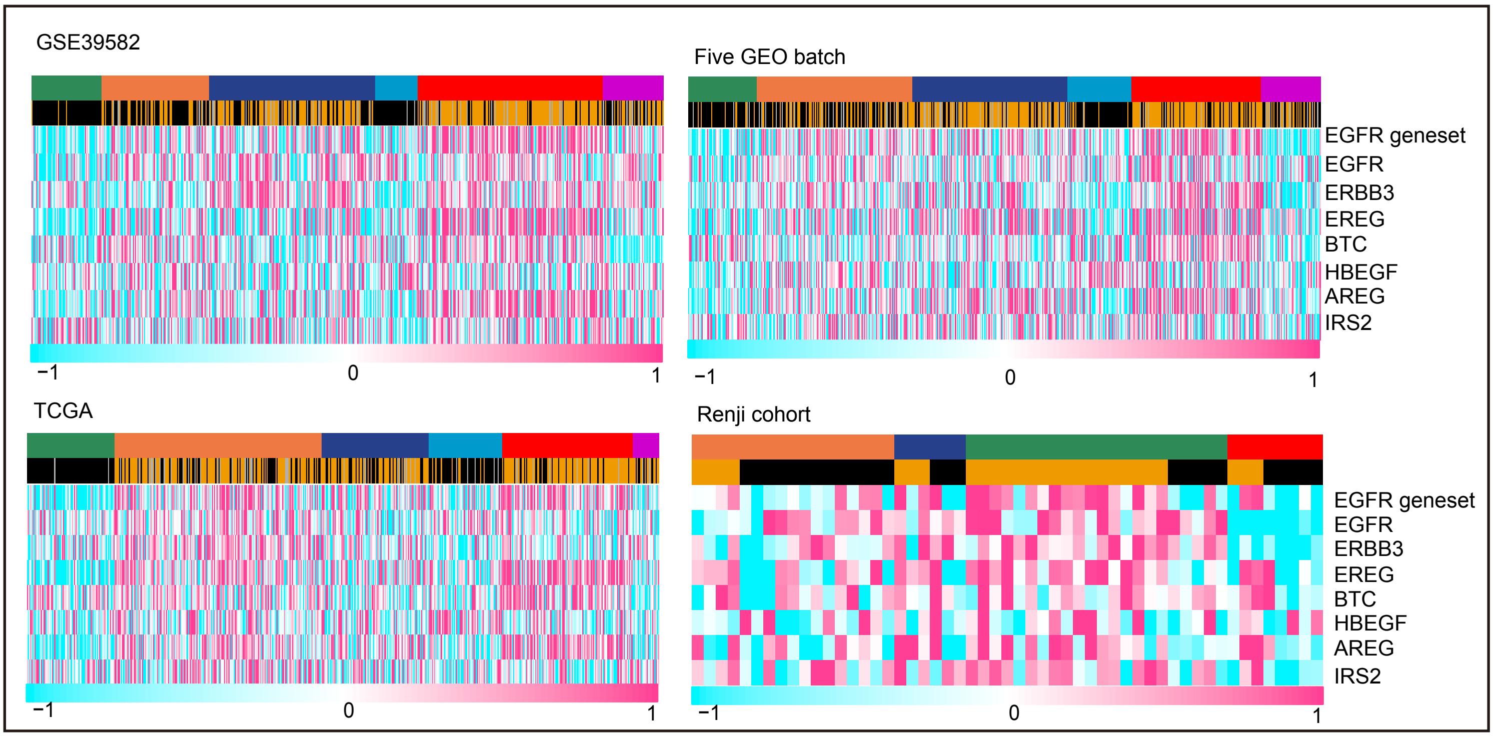
GSE39582
Five GEO batch
TCGA
Renji cohort
EGFR geneset
EGFR
ERBB3
EREG
BTC
HBEGF
AREG
IRS2
−1
0
1

m

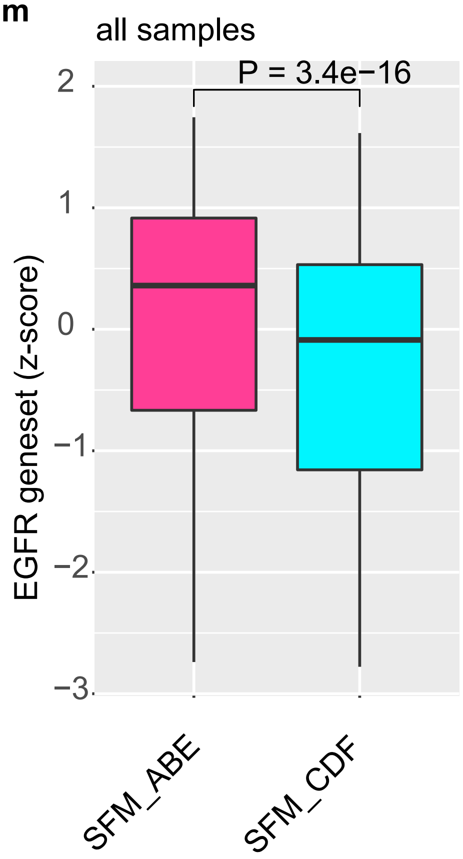
all samples
P = 3.4e−16
EGFR geneset (z-score)
2
1
0
−1
−2
−3
SFM_ABE
SFM_CDF

n

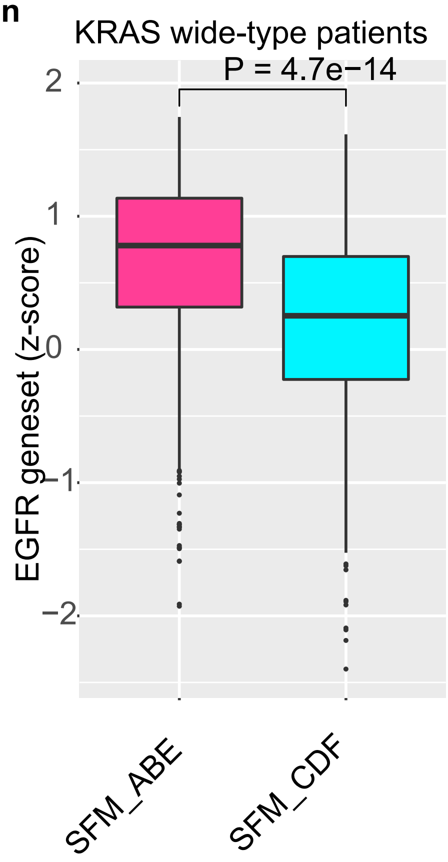
KRAS wide-type patients
P = 4.7e−14
EGFR geneset (z-score)
2
1
0
−1
−2
SFM_ABE
SFM_CDF

o

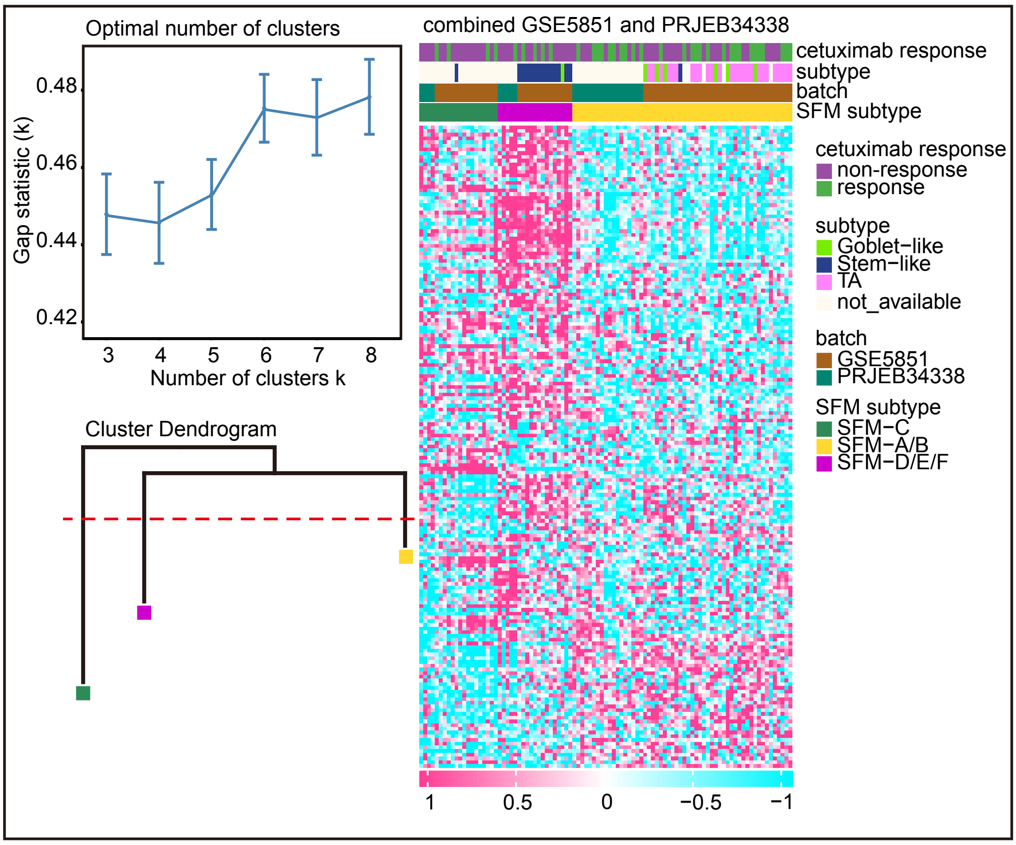
Optimal number of clusters
Gap statistic (k)
0.48
0.46
0.44
0.42
3 4 5 6 7 8
Number of clusters k
Cluster Dendrogram
combined GSE5851 and PRJEB34338
cetuximab response
subtype
batch
SFM subtype
cetuximab response
non−response
response
subtype
Goblet−like
Stem−like
TA
not_available
batch
GSE5851
PRJEB34338
SFM subtype
SFM−C
SFM−A/B
SFM−D/E/F
1 0.5 0 −0.5 −1

p

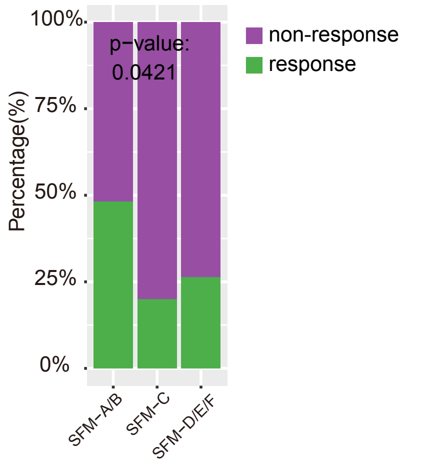
100%
75%
50%
25%
0%
Percentage(%)
p−value:
0.0421
non−response
response
SFM−A/B
SFM−C
SFM−D/E/F

q

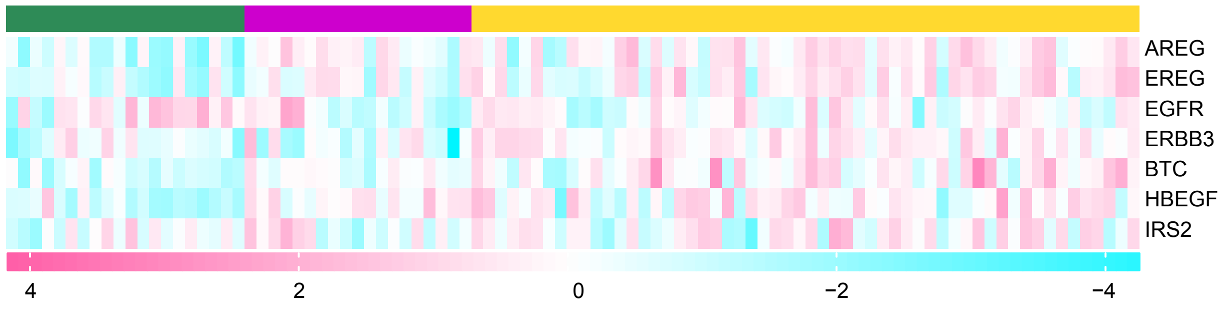
AREG
EREG
EGFR
ERBB3
BTC
HBEGF
IRS2
4 2 0 −2 −4

r

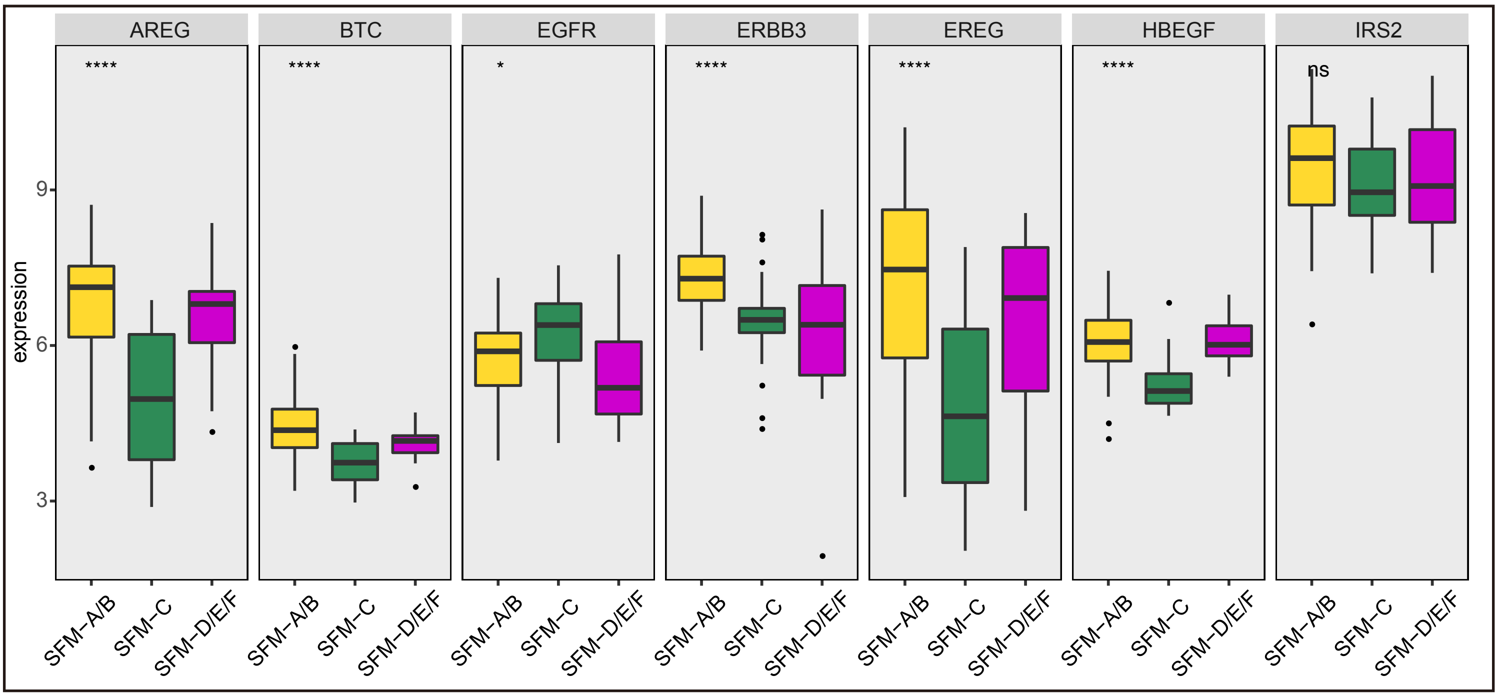
AREG
BTC
EGFR
ERBB3
EREG
HBEGF
IRS2
****
****
*
****
****
****
ns
expression
9
6
3
SFM−A/B
SFM−C
SFM−D/E/F

Supplementary Figure 2. Distinct sensitivities among SFM subtypes to FOLFIRI and FOLFOX chemotherapy regimens and EGFR inhibitors. (a-b) K-means algorithm for molecular subtype classification in GSE62080 (n = 21, a) and combined GSE19860, GSE28702 and GSE69657 (n = 142, b). Gap statistics were plotted from K-means classification within the predetermined number of clusters (k) varying from 3 to 8 using SFM. Unsupervised hierarchical clustering displayed distinct molecular subtypes. (c) K-means algorithm for molecular subtype classification in combined GSE104645 and GSE72970 (n = 158). (d) Stacked plot showing the proportion of responders and non-responders amongst SFM subtypes in in combined GSE104645 and GSE72970 dataset. P value was calculated using Chi-squared test. (e-f) Kaplan-Meier plots of SFM subtypes in combined GSE104645 and GSE72970 dataset for OS (e) and progression free survival (PFS, f). The tick marks on the Kaplan-Meier curves indicated the censored patients. The differences between the curves were determined by the log-rank test. (g) Heatmaps showing association of individual CRC patient's response to FOLFIRI, FOLFOX and EGFR inhibitors based on published gene signatures using NTP algorithm. In these analyses, samples with FDR < 0.2 were regarded as significant. (h-i) Stacked plots showing the distribution of responders and non-responders among SFM-subtypes based on two different approaches for FOLFOX response prediction, five gene pair prediction (h) and NTP (i). P value was calculated using Chi-squared test. (j-k) Cetuximab response association among SFM subtypes. Bar-of-pie charts showing the distribution of SFM subtypes in terms of cetuximab response after combining SFM A-B-E as one compared with SFM C-D-F in all CRC cases (j) and only in KRAS wild-type cases (k). (l) Heatmaps showing response to cetuximab, quantified expression of gene set of the EGFR pathway activity by applying GSVA and expression of individual genes of the EGFR gene set among SFM subtypes in training and validation datasets. (m-n) Box plots of the EGFR gene set across SFM subtypes after combining SFM A-B-E as one compared with SFM C-D-F in all CRC cases (m) and only in KRAS wild-type cases (n). (o) K-means algorithm for molecular subtype classification in combined GSE5851 and PRJEB34338 treated with cetuximab (n = 95). (d) Stacked plot showing the proportion of responders and non-responders amongst SFM subtypes in combined GSE5851 and PRJEB34338 dataset. P value was calculated using Chi-squared test. (q) Heatmaps showing the expression profiles of the EGFR associated genes among SFM subtypes in combined GSE5851 and PRJEB34338 dataset. (r) Box plots comparing the expression of EGFR associated genes among SFM subtypes in combined GSE5851 and PRJEB34338 dataset. The statistical difference was examined using the Kruskal-Wallis test. (ns, P > 0.05; *, P <= 0.05; **, P <= 0.01; ***, P <= 0.001; ****, P <= 0.0001). For m, n and r, box hinges represent 1st and 3rd quartiles, and middle represents the median. The short horizontal lines represent the standard deviations. The upper and lower whiskers extend from hinges up and down indicate the most extreme values that are within 1.5*IQR (interquartile range) of the respective hinge.

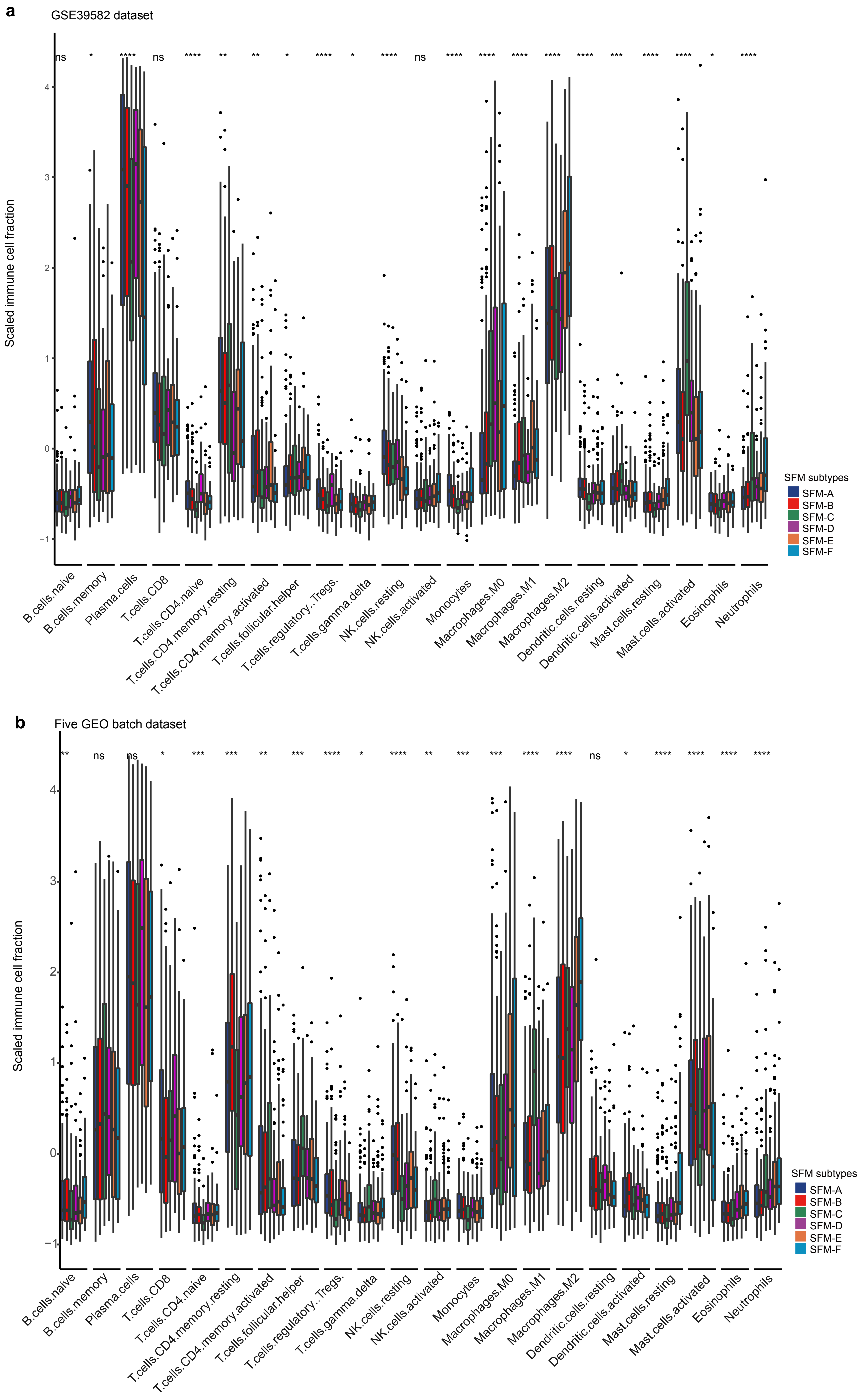
a
GSE39582 dataset
Scaled immune cell fraction
B.cells.naive
B.cells.memory
Plasma.cells
T.cells.CD8
T.cells.CD4.naive
T.cells.CD4.memory.resting
T.cells.CD4.memory.activated
T.cells.follicular.helper
T.cells.regulatory..Tregs.
T.cells.gamma.delta
NK.cells.resting
NK.cells.activated
Monocytes
Macrophages.M0
Macrophages.M1
Macrophages.M2
Dendritic.cells.resting
Dendritic.cells.activated
Mast.cells.resting
Mast.cells.activated
Eosinophils
Neutrophils
SFM subtypes
SFM-A
SFM-B
SFM-C
SFM-D
SFM-E
SFM-F
b
Five GEO batch dataset
Scaled immune cell fraction
B.cells.naive
B.cells.memory
Plasma.cells
T.cells.CD8
T.cells.CD4.naive
T.cells.CD4.memory.resting
T.cells.CD4.memory.activated
T.cells.follicular.helper
T.cells.regulatory..Tregs.
T.cells.gamma.delta
NK.cells.resting
NK.cells.activated
Monocytes
Macrophages.M0
Macrophages.M1
Macrophages.M2
Dendritic.cells.resting
Dendritic.cells.activated
Mast.cells.resting
Mast.cells.activated
Eosinophils
Neutrophils
SFM subtypes
SFM-A
SFM-B
SFM-C
SFM-D
SFM-E
SFM-F

c

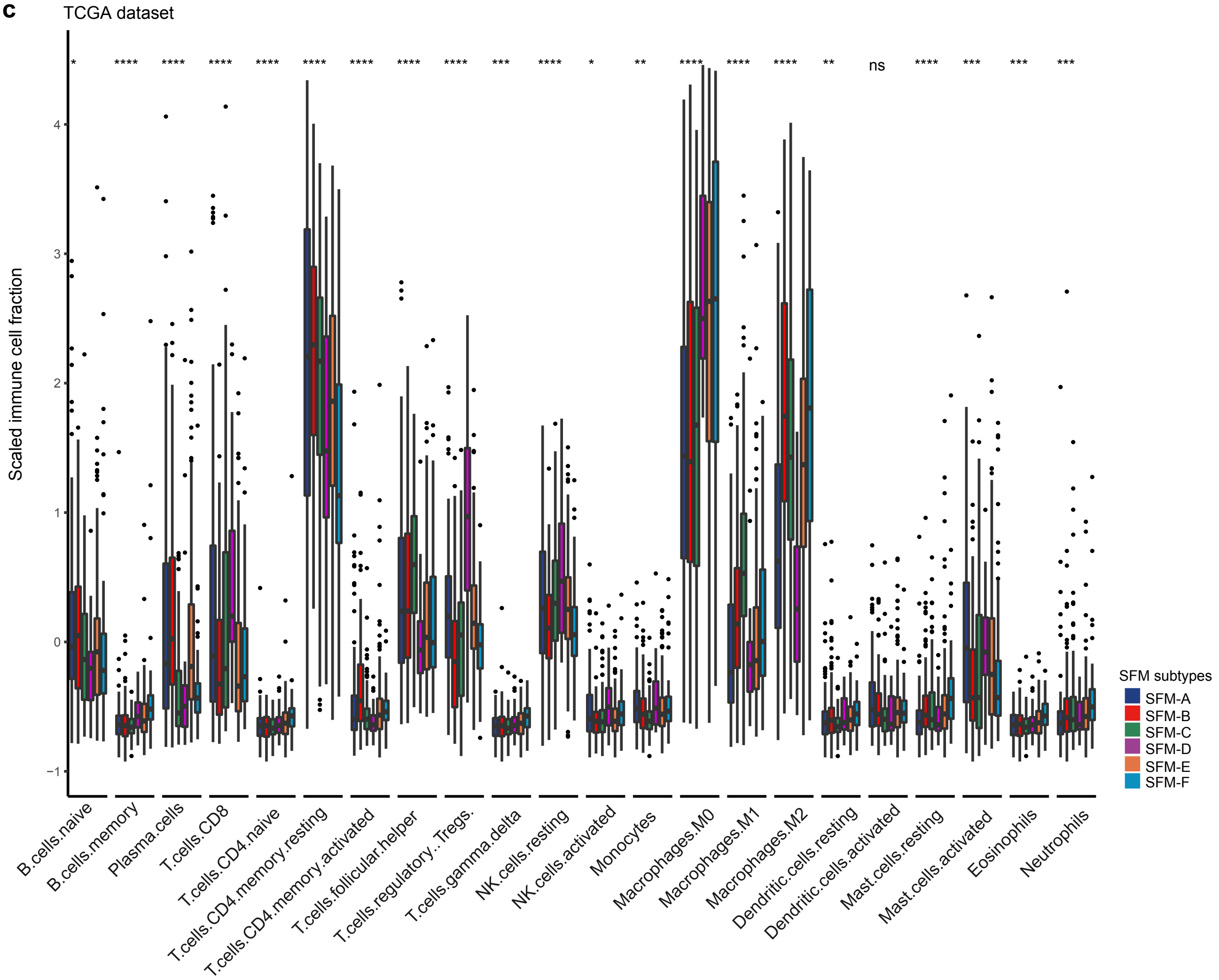
TCGA dataset
Scaled immune cell fraction
SFM subtypes
SFM-A
SFM-B
SFM-C
SFM-D
SFM-E
SFM-F
B.cells.naive
B.cells.memory
Plasma.cells
T.cells.CD8
T.cells.CD4.naive
T.cells.CD4.memory.resting
T.cells.CD4.memory.activated
T.cells.follicular.helper
T.cells.regulatory..Tregs.
T.cells.gamma.delta
NK.cells.resting
NK.cells.activated
Monocytes
Macrophages.M0
Macrophages.M1
Macrophages.M2
Dendritic.cells.resting
Dendritic.cells.activated
Mast.cells.resting
Mast.cells.activated
Eosinophils
Neutrophils
ns

Supplementary Figure 3. Estimate immune cells filtration using CIBERSORT. The fraction of immune cells were plotted using box plot across SFM subtypes (a, GSE39582; b, Five GEO batch; c, TCGA). The statistical difference was examined using the Kruskal-Wallis test. (ns, P > 0.05; *, P <= 0.05; **, P <= 0.01; ***, P <= 0.001; ****, P <= 0.0001). Box hinges represent 1st and 3rd quartiles, and middle represents the median. The short horizontal lines represent the standard deviations. The upper and lower whiskers extend from hinges up and down indicate the most extreme values that are within 1.5*IQR (interquartile range) of the respective hinge.

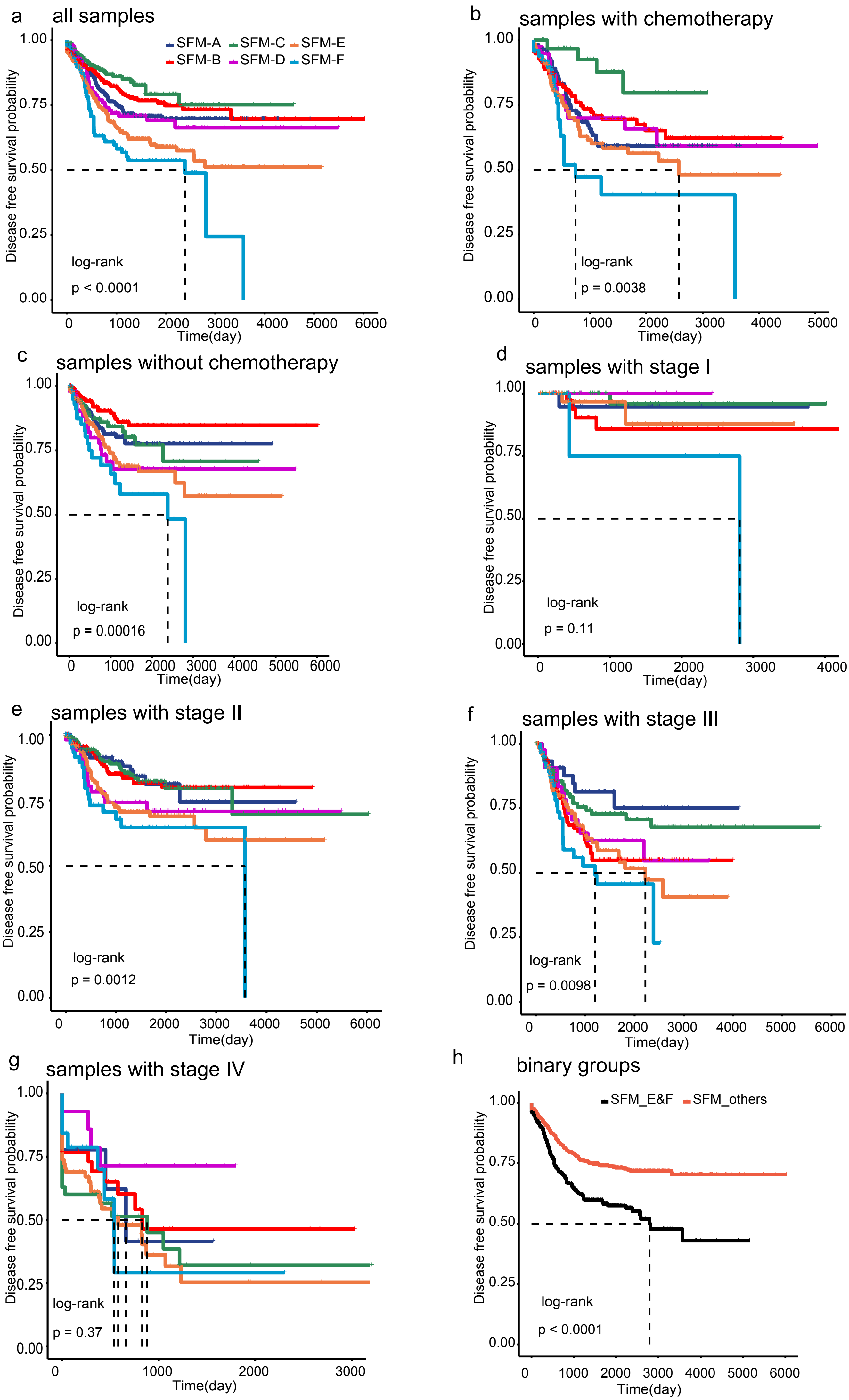
a
all samples
SFM-A
SFM-B
SFM-C
SFM-D
SFM-E
SFM-F
log-rank
p < 0.0001
b
samples with chemotherapy
log-rank
p = 0.0038
c
samples without chemotherapy
log-rank
p = 0.00016
d
samples with stage I
log-rank
p = 0.11
e
samples with stage II
log-rank
p = 0.0012
f
samples with stage III
log-rank
p = 0.0098
g
samples with stage IV
log-rank
p = 0.37
h
binary groups
SFM_E&F
SFM_others
log-rank
p < 0.0001
Disease free survival probability
Time(day)

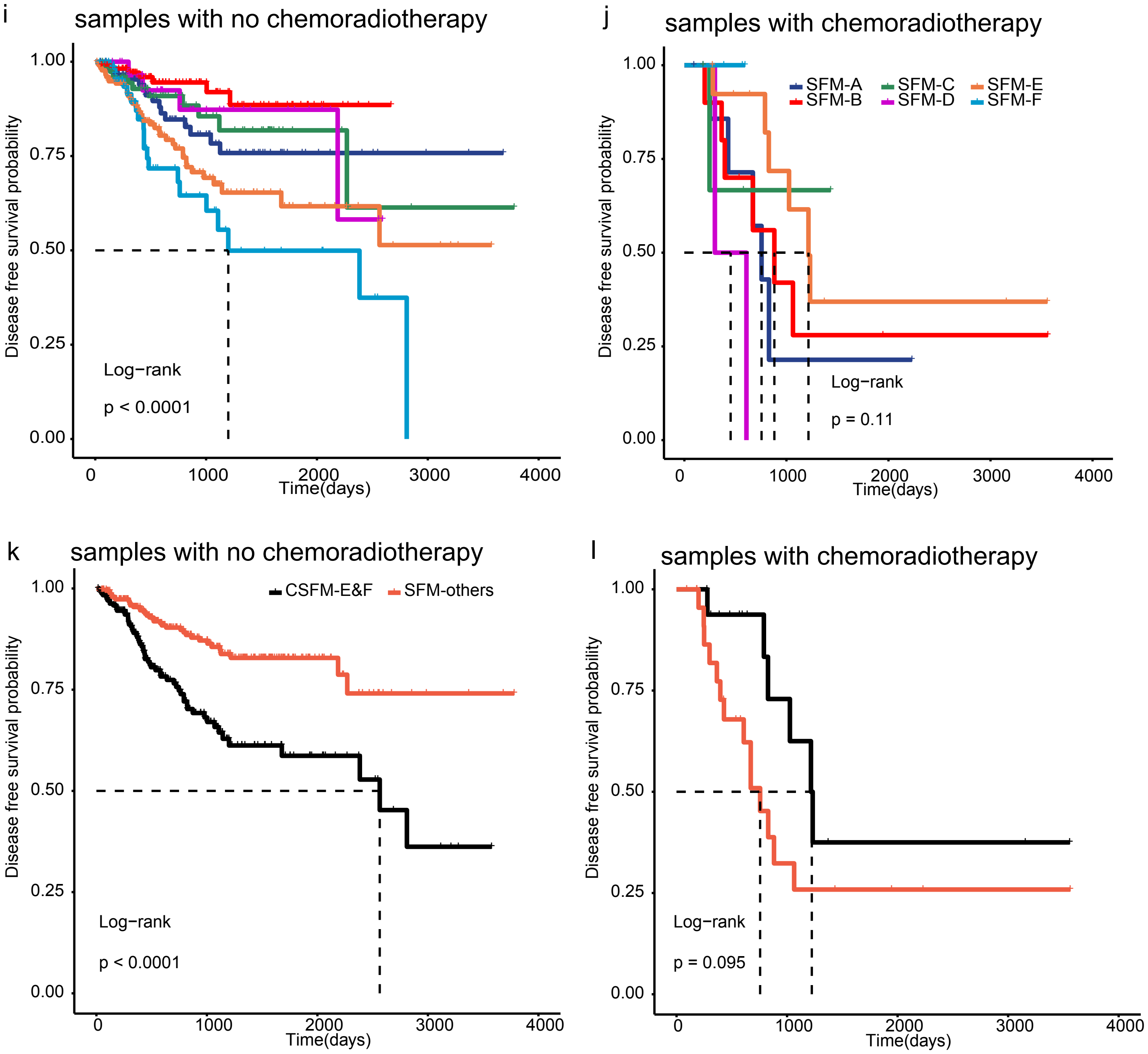
i
samples with no chemoradiotherapy
Disease free survival probability
1.00
0.75
0.50
0.25
0.00
Log−rank
p < 0.0001
0
1000
2000
3000
4000
Time(days)
j
samples with chemoradiotherapy
SFM-A
SFM-B
SFM-C
SFM-D
SFM-E
SFM-F
Disease free survival probability
1.00
0.75
0.50
0.25
0.00
Log−rank
p = 0.11
0
1000
2000
3000
4000
Time(days)
k
samples with no chemoradiotherapy
CSFM-E&F
SFM-others
Disease free survival probability
1.00
0.75
0.50
0.25
0.00
Log−rank
p < 0.0001
0
1000
2000
3000
4000
Time(days)
l
samples with chemoradiotherapy
Disease free survival probability
1.00
0.75
0.50
0.25
0.00
Log−rank
p = 0.095
0
1000
2000
3000
4000
Time(days)

m

Univariate risk factors	HR	95%CI	P value
age	0.99	(0.99 − 1.00)	0.0966
gender	1.16	(0.95 − 1.42)	0.1395
stage.I (ref)	1.00		
stage.II	2.97	(1.57 − 5.65)	0.0009
stage.III	5.67	(2.99 − 10.73)	<0.00001
stage.IV	13.92	(7.21 − 26.87)	<0.00001
SFM_C (ref)	1.00		
SFM_A	1.55	(1.01 − 2.39)	0.0455
SFM_B	1.28	(0.83 − 2.00)	0.2670
SFM_D	1.74	(1.05 − 2.88)	0.0319
SFM_E	2.39	(1.57 − 3.62)	<0.00001
SFM_F	3.10	(1.94 − 4.93)	<0.00001
C3.subtype.Marisa.et.al (ref)	1.00		
C1.subtype.Marisa.et.al	1.24	(0.71 − 2.16)	0.4453
C2.subtype.Marisa.et.al	0.65	(0.34 − 1.22)	0.1801
C4.subtype.Marisa.et.al	2.40	(1.35 − 4.29)	0.0030
C5.subtype.Marisa.et.al	1.14	(0.67 − 1.95)	0.6220
C6.subtype.Marisa.et.al	1.74	(0.96 − 3.14)	0.0677
CMS1.subtype.Guinney.et.al (ref)	1.00		
CMS2.subtype.Guinney.et.al	1.04	(0.72 − 1.49)	0.8453
CMS3.subtype.Guinney.et.al	0.99	(0.63 − 1.56)	0.9785
CMS4.subtype.Guinney.et.al	2.00	(1.40 − 2.87)	0.0002
MSI.CIMP.TCGA (ref)	1.00		
CIN.TCGA	1.04	(0.35 − 3.06)	0.9478
Invasive.TCGA	1.00	(0.35 − 3.54)	0.8625
Enterocyte.Goblet−like.subtype.Sadanandam.et.al (ref)	1.11		
Inflammatory.subtype.Sadanandam.et.al	1.08	(0.52 − 2.24)	0.8295
Stem−like.subtype.Sadanandam.et.al	1.74	(0.94 − 3.22)	0.0778
TA.subtype.Sadanandam.et.al	0.91	(0.47 − 1.76)	0.7880
CCS1.subtype.De.Sousa.et.al (ref)	1.00		
CCS2.subtype.De.Sousa.et.al	2.13	(0.43 − 10.54)	0.3555
CCS3.subtype.De.Sousa.et.al	11.05	(3.09 − 39.51)	0.0002
CRISA.subtype.Isella.et.al (ref)	1.00		
CRISB.subtype.Isella.et.al	1.54	(1.10 − 2.14)	0.0119
CRISC.subtype.Isella.et.al	0.82	(0.59 − 1.15)	0.2487
CRISD.subtype.Isella.et.al	1.04	(0.72 − 1.50)	0.8479
CRISE.subtype.Isella.et.al	1.16	(0.81 − 1.66)	0.4143
low.oncotype.DX (ref)	1.00		
high.oncotype.DX	1.96	(1.51 − 2.54)	<0.00001
intermediate.oncotype.DX	1.59	(1.16 − 2.19)	0.0043

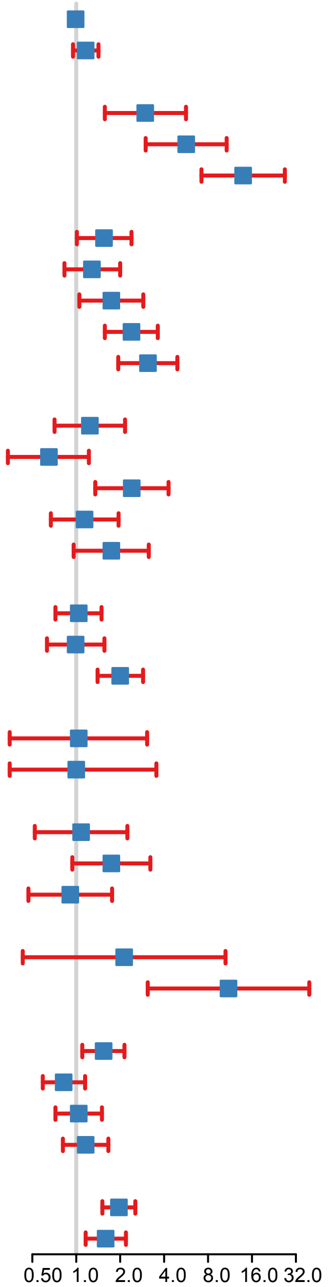

n

Multivariate risk factors (adjusted by age, gender and AJCC stage)	HR	95%CI	P value
SFM_C (ref)	1.00		
FSM_A	1.40	0.91 − 2.16	0.1277
SFM_B	1.15	0.74 − 1.79	0.5392
SFM_D	1.34	0.80 − 2.22	0.2649
SFM_E	1.89	1.24 − 2.89	0.0031
SFM_F	2.48	1.55 − 3.96	0.0001
C3.subtype.Marisa.et.al (ref)	1.00		
C1.subtype.Marisa.et.al	1.29	0.74 − 2.25	0.3731
C2.subtype.Marisa.et.al	0.72	0.38 − 1.37	0.3183
C4.subtype.Marisa.et.al	1.81	1.01 − 3.25	0.0462
C5.subtype.Marisa.et.al	1.09	0.64 − 1.87	0.7399
C6.subtype.Marisa.et.al	1.90	1.04 − 3.46	0.0364
CMS1.subtype.Guinney.et.al (ref)	1.00		
CMS2.subtype.Guinney.et.al	0.95	0.66 − 1.37	0.7771
CMS3.subtype.Guinney.et.al	1.02	0.64 − 1.60	0.9473
CMS4.subtype.Guinney.et.al	1.66	1.15 − 2.39	0.0063
MSI.CIMP.TCGA (ref)	1.00		
CIN.TCGA	0.91	0.3 − 2.8	0.8737
Invasive.TCGA	1.38	0.41 − 4.67	0.6077
Enterocyte/Goblet−like.subtype.Sadanandam.et.al (ref)	1.00		
Inflammatory.subtype.Sadanandam.et.al	1.11	0.53 − 2.34	0.7737
Stem−like.subtype.Sadanandam.et.al	1.43	0.76 − 2.70	0.2667
TA.subtype.Sadanandam.et.al	0.84	0.43 − 1.64	0.6092
CCS1.subtype.De.Sousa.et.al (ref)	1.00		
CCS2.subtype.De.Sousa.et.al	2.29	0.44 − 11.83	0.3214
CCS3.subtype.De.Sousa.et.al	11.32	3.14 − 40.84	0.0002
CRISA.subtype.Isella.et.al (ref)	1.00		
CRISB.subtype.Isella.et.al	1.26	0.90 − 1.77	0.1784
CRISC.subtype.Isella.et.al	0.72	0.52 − 1.01	0.0556
CRISD.subtype.Isella.et.al	0.97	0.67 − 1.41	0.8738
CRISE.subtype.Isella.et.al	1.03	0.72 − 1.48	0.8577
low.oncotype.DX (ref)	1.00		
high.oncotype.DX	1.93	1.49 − 2.51	<0.00001
intermediate.oncotype.DX	1.33	0.97 − 1.84	0.0776

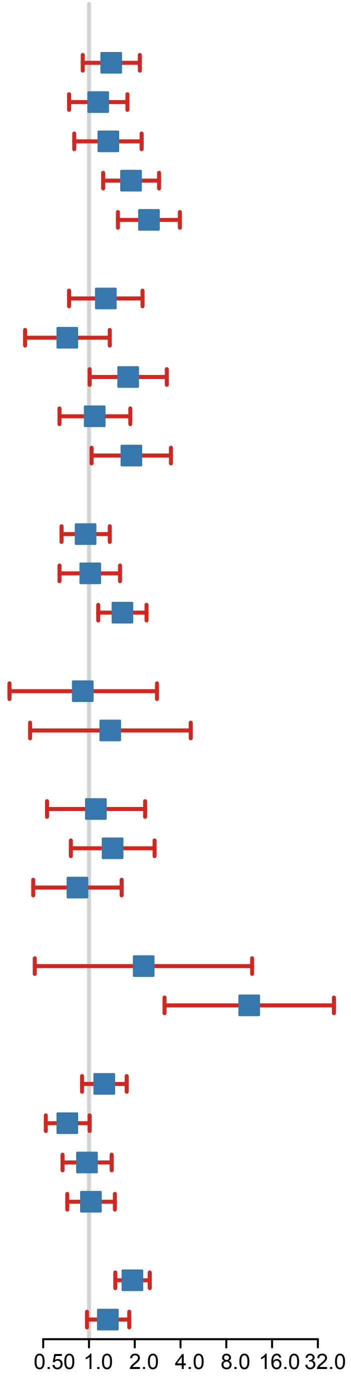

Supplementary Figure 4. Survival analysis across SFM subtypes using aggregated dataset. (a) Kaplan-Meier plot of DFS of all patients. (b) Kaplan-Meier plot of DFS in CRC patients with chemotherapy. (b) Kaplan-Meier plot of DFS in CRC patients without chemotherapy. (d-g) Kaplan-Meier plot of DFS in individual stage patients. (h) Kaplan-Meier plot of DFS after combining SFM E and F as one. (i-j) Kaplan-Meier plot of DFS in CRC patients with or without chemoradiotherapy. (k-l) Kaplan-Meier plot of DFS in CRC patients with or without chemoradiotherapy after combining SFM E and F as one. (l) Univariate Cox regression analysis for age, gender, AJCC stage, SFM subtypes, Oncotype DX and previous CRC classifiers. (m) Multivariate Cox regression analysis for SFM subtypes, Oncotype DX and previous CRC classifiers. These included factors were individually adjusted by age, gender and AJCC stage.

Supplementary Table 1. Data sets and variables used for correlative analyses

Dataset	Download Source	Platform	Tissue	Study design	Total	Age	Gender	stage(early/late)	Site(L/R)	location(distal)	AJCC Stage	Adjctx	Adjxrt	KRAS mut	BRAF mut	TP53 mut	hypermutation	MSI status	CIMP status	CIN status	RFS/DFS	OS
GSE39395	public	Affymetrix Human Genome U133 Plus 2.0 Array	fresh	Prospective series	24																	
GSE39396	public	Affymetrix HT HG-U133+ PM Array Plate	fresh	Prospective series	24																	
GSE14333	public	Affymetrix Human Genome U133 Plus 2.0 Array	fresh	Prospective series	157	*	*	*	*		*	*	*								*	
GSE39582	public	Affymetrix Human Genome U133 Plus 2.0 Array	fresh	Prospective series	566	*	*	*	*	*	*	*		*	*	*		*	*	*	*	
GSE33113	public	Affymetrix Human Genome U133 Plus 2.0 Array	fresh	Prospective series	90	*	*				*			*	*			*	*		*	
GSE17536	public	Affymetrix Human Genome U133 Plus 2.0 Array	fresh	Prospective series	177	*	*	*			*										*	*
GSE17537	public	Affymetrix Human Genome U133 Plus 2.0 Array	fresh	Prospective series	55	*	*	*			*										*	*
GSE62080(Rio)	public	Affymetrix Human Genome U133 Plus 2.0 Array	fresh	Prospective series	21																	
GSE37892	public	Affymetrix Human Genome U133 Plus 2.0 Array	fresh	Prospective series	130	*	*		*		*										*	
GSE28702	public	Affymetrix Human Genome U133 Plus 2.0 Array	fresh	Prospective series	83	*			*	*		*										
GSE19860	public	Affymetrix Human Genome U133 Plus 2.0 Array	fresh	Prospective series	29																	
GSE69657	public	Affymetrix Human Genome U133 Plus 2.0 Array	fresh	Prospective series	30	*	*				*											
TCGA	public	RNA sequencing	fresh	Prospective series	577	*	*			*	*	*	*	*	*		*	*	*		*	*
Renji cohort	renji hospital	RNA sequencing	fresh	Prospective series	53	*	*		*	*	*											
GSE5851	public	Affymetrix Human Genome U133 Plus 2.0 Array	fresh	Prospective series	68																	
PRJEB34338	public	RNA sequencing	fresh	Prospective series	27																	
GSE72970	public	Affymetrix Human Genome U133 Plus 2.0 Array	fresh	Prospective series	92																	
GSE104645	public	Agilent-014850 Whole Human Genome Microarray 4x4	fresh	Prospective series	66																	
GSE81861	public	single cell RNA seq	fresh	Prospective series	363 cells																	
GSE103322	public	single cell RNA seq	fresh	Prospective series	5502 cells																	
GSE72056	public	single cell RNA seq	fresh	Prospective series	4054 cells																	
GSE75688	public	single cell RNA seq	fresh	Prospective series	515 cells																	

Supplementary Table 2. gene lists of Signature associated with FOFIRI resistant and Microenvironment (SFM)

probe_id	Gene Symbol
209534_x_at	NA
221718_s_at	NA
222024_s_at	NA
213099_at	NA
213001_at	NA
213004_at	NA
209286_at	NA
214758_at	NA
209588_at	NA
209589_s_at	NA
210651_s_at	NA
221945_at	NA
218084_x_at	NA
207966_s_at	NA
1555997_s_at	NA
1553678_a_at	NA
1554306_at	NA
212086_x_at	NA
1556763_at	NA
1559949_at	NA
1561896_at	NA
1562306_at	NA
1562834_at	NA
1569672_at	NA
1570089_at	NA
213183_s_at	NA
215557_at	NA
215831_at	NA
215962_at	NA
216040_x_at	NA
217051_s_at	NA
217083_at	NA
217193_x_at	NA
217409_at	NA
224082_at	NA
224084_at	NA
229629_at	NA
234382_x_at	NA
235674_at	NA
236515_at	NA
237269_at	NA
237643_at	NA
239039_at	NA
239255_at	NA
239610_at	NA
240327_at	NA
241052_at	NA
241780_at	NA
242264_at	NA
242588_at	NA
242664_at	NA
242993_at	NA
243876_at	NA
244476_at	NA
244579_at	NA
244838_at	NA
1559400_s_at	NA
224940_s_at	NA
224941_at	NA
224942_at	NA
203370_s_at	NA
206138_s_at	NA
204517_at	NA
1554997_a_at	NA
212012_at	NA
202963_at	NA
212415_at	42984
208561_at	ABCC9
1567622_at	ABLIM2
209749_s_at	ACE
205180_s_at	ADAM8
206629_at	ADAMTSL2
234799_at	ADARB1
224884_at	AKAP13
224229_s_at	AKT3
36865_at	ANGEL1
219514_at	ANGPTL2
210143_at	ANXA10
213454_at	APITD1
39248_at	AQP3
232567_at	ARHGAP8
219636_s_at	ARMC9
221483_s_at	ARPP19
230275_at	ARSI
235619_at	ASB4
203454_s_at	ATOX1
214255_at	ATP10A
201172_x_at	ATP6V0E1
234955_at	AZGP1P1
222915_s_at	BANK1
1559220_at	BC039681
1562997_a_at	BC041484
228311_at	BCL6B
1570383_at	BMP1
230722_at	BNC2
226280_at	BNIP2
231859_at	C14orf132
228373_at	C16orf72
219476_at	C1orf116
1556721_at	C20orf203
235434_at	CCDC134
1568796_at	CCDC157
227043_at	CCDC159
1554175_at	CD300LB
35150_at	CD40
225685_at	CDC42EP3
219534_x_at	CDKN1C
227290_at	CDYL2
1560255_at	CELF2-AS1
1554488_at	CEP70
228007_at	CEP85L
237367_x_at	CFLAR
208807_s_at	CHD3
1568704_a_at	CHERP
204591_at	CHL1
239717_at	CHRNA10
206703_at	CHRNB1
205101_at	CIITA
230223_at	CINP
1569421_at	CLDN4
204482_at	CLDN5
1567079_at	CLN6
238527_at	CLUHP3
229604_at	CMAHP
239989_at	CNTLN
204345_at	COL16A1
205509_at	CPB1
201116_s_at	CPE
216314_at	CRISP1
218648_at	CRTC3
200621_at	CSRP1
204309_at	CYP11A1
219149_x_at	DBR1
226938_at	DCAF4
242605_at	DCN
238524_at	DENND2D
228184_at	DISP1
215341_at	DNAH6
241709_s_at	DOCK1
235721_at	DTX3
209365_s_at	ECM1
214445_at	ELL2
229011_at	EMP1
211165_x_at	EPHB2
234552_at	EPPK1
224030_s_at	FAM115A
223019_at	FAM129B
1553749_at	FAM76B
222013_x_at	FAM86A
223545_at	FANCD2
232968_at	FANK1
236405_at	FBXO41
231093_at	FCRL3
206412_at	FER
1565752_at	FGD2
229652_s_at	FOXN3
220819_at	FRMD1
226072_at	FUK
224252_s_at	FXYD5
208868_s_at	GABARAPL1
201816_s_at	GBAS
211767_at	GINS4
221407_at	GJD2
212045_at	GLG1
229435_at	GLIS3
35436_at	GOLGA2
208798_x_at	GOLGA8A
219388_at	GRHL2
220190_s_at	GTF2A1L
213835_x_at	GTPBP3
215071_s_at	HIST1H2AC
1559891_at	HMGA2
214851_at	HNF4A
204110_at	HNMT
39763_at	HPX
226944_at	HTRA3
1564573_at	IFITM10
203426_s_at	IGFBP5
212657_s_at	IL1RN
205291_at	IL2RB
207538_at	IL4
227774_s_at	IMPAD1
203331_s_at	INPP5D
238584_at	IQCA1
204562_at	IRF4
211945_s_at	ITGB1
203723_at	ITPKB
209760_at	KIAA0922
216077_s_at	L3MBTL1
200770_s_at	LAMC1
202267_at	LAMC2
1559224_at	LCE1E
1561327_at	LINC00242
1569090_x_at	LINC00957
212089_at	LMNA
231232_at	LOC100506125
209360_s_at	RUNX1
1556888_at	LOC101927121
240382_at	LOC101928076
244609_at	LOC102724487
212707_s_at	RASA4
1569739_at	LOC221946
244649_at	LOC646484
202822_at	LPP
200785_s_at	LRP1
202904_s_at	LSM5
202729_s_at	LTBP1
1559388_a_at	LYNX1
1559256_at	MAGI1
235421_at	MAP3K8
214969_at	MAP3K9
206296_x_at	MAP4K1
211499_s_at	MAPK11
244662_at	MBD5
228632_at	MEG9
1552432_at	MFSD6L
232568_at	MGC24103
212593_s_at	PDCD4
201875_s_at	MPZL1
209928_s_at	MSC
225080_at	MYO1C
241966_at	MYO5A
212803_at	NAB2
228608_at	NALCN
226707_at	NAPRT
217465_at	NCKAP1
1554082_a_at	NOL9
204310_s_at	NPR2
208709_s_at	NRD1
222075_s_at	OAZ3
234371_at	OR2AD1P
223464_at	OSBPL5
228703_at	P4HA3
228128_x_at	PAPPA
205717_x_at	PCDHGA1
215836_s_at	NA
221957_at	PDK3
214121_x_at	PDLIM7
218634_at	PHLDA3
1554822_at	PHTF2
210417_s_at	PI4KB
203035_s_at	PIAS3
229245_at	PLEKHA6
204518_s_at	PPIC
229375_at	PPIE
238522_at	PPP3CB-AS1
1566544_at	PPP5D1
240015_at	PRKCQ-AS1
204748_at	PTGS2
217177_s_at	PTPRB
1552480_s_at	PTPRC
219812_at	PVRIG
203149_at	PVRL2
212013_at	PXDN
241938_at	QKI
238853_at	RAB3IP
228548_at	RAP1A
213280_at	RAP1GAP2
224068_x_at	RBM22
239169_at	RDM1
202964_s_at	RFX5
203823_at	RGS3
242985_x_at	RNF180
1555529_at	RNH1
227943_at	RP11-196G18.24
217336_at	RPS10P2
213437_at	RUFY3
1560665_at	SCPEP1
230232_at	SEL1L3
209722_s_at	SERPINB9
214853_s_at	SHC1
1561880_a_at	SIGLEC16
206519_x_at	SIGLEC6
206052_s_at	SLBP
215274_at	SLC12A3
1568718_at	SLC22A23
225212_at	SLC25A25
212907_at	SLC30A1
219885_at	SLFN12
225014_at	SMIM20
215617_at	SPATS2L
205861_at	SPIB
204955_at	SRPX
233141_s_at	ST7L
1555088_x_at	STAT5B
214954_at	SUSD5
202565_s_at	SVIL
44702_at	SYDE1
202796_at	SYNPO
232387_at	SYNRG
1563360_at	T-18
234402_at	TCRVA15
226778_at	TDRP
201174_s_at	TERF2IP
1567704_at	TFE3
235086_at	THBS1
201666_at	TIMP1
202011_at	TJP1
216005_at	TNC
227345_at	TNFRSF10D
227812_at	TNFRSF19
205641_s_at	TRADD
202734_at	TRIP10
218502_s_at	TRPS1
239123_at	TSC22D1
220309_at	TTC22
219633_at	TTPAL
221964_at	TULP3
227141_at	TYW3
1570145_at	UCHL5
208998_at	UCP2
229812_at	USP48
1552415_a_at	WFDC9
228949_at	WLS
208318_s_at	XYLB
233031_at	ZEB2
213876_x_at	ZRSR2

Supplementary Table 3. Previously published gene signatures used to identify CRC molecuar subtypes

CRC subtype	Reference
De_Sousa_E_Melo_2013	De Sousa E Melo et al. Nature Medicine. 2013
De_Sousa_E_Melo_2011	De Sousa E Melo et al. Cell Stem Cell. 2011
Sadanandam_2013	Sadanandam et al. Nature Medicine. 2013
Marisa_2013	Marisa et al. Plos Medicine. 2013
Isella_2017	Claudio Isella et al. Nature communications. 2017
Roepman_2013	Roepman et al. International Journal of Cancer. 2013
Budinska_2013	Budinska et all. Journal of Pathology. 2013
Popovici_2012	Popovici et al. Journal of Clinical Oncology. 2012

Gene lists

De_Sousa_E_Melo_	De_Sousa_E_Melo_	Sadanandam_2013	Marisa_2013	Isella_2017	Roepman_2013	Budinska_2013	Popovici_2012
SRSF6	ACSL6	SFRP2	RGS1	REG4	HSPA4L	CEACAM7	ACOX1
HNRNPL	ADH6	MGP	CD69	MUC2	SLC7A11	FAM55A	AIFM3
ZIC2	AGPAT9	COL10A1	LILRB2	FCGBP	NUDT6	FAM55D	AMACR
CYP1B1	AKR1B10	MSRB3	GZMB	ZG16	ME1	MUC12	APCDD1
EIF5A	ANXA6	CYP1B1	LOC100287927	SPINK4	DLG7	SLC26A2	AQP5
TOB1	APCDD1	FNDC1	ST8SIA4	ST6GALNAC1	KNTC2	SLC26A3	ARID3A
KIAA0226L	APOBEC1	SFRP4	FCGR2B	FAM3D	PRC1	AK3L1	C10orf99
ITCH	AREGB	GAS1	OAS2	CLCA1	ECHS1	ARID3A	C11orf9
QPRT	ARL14	CCDC80	TLR1	ITLN1	DEPDC1	EREG	C13orf18
RBM6	ASB4	SPOCK1	TFEC	TFF1	ACADSB	LDLRAD3	C20orf111
PSIP1	ATXN1	THBS2	MGC29506	CDC42EP5	EIF4A2	ZBTB10	CBFA2T2
HTR2B	B3GNTL1	MFAP5	IGKV1-5	TCN1	MREG	HEPH	CD55
C10orf99	BZRAP1	ASPN	GZMA	CA2	NIPA1	KRT20	CDX2
PPP1R14C	C15orf48	TNS1	GPR34	BCL2L15	TIAL1	LOC644844	CELP
GAS1	C1orf105	TAGLN	CCL8	PIGR	URM1	MUC20	CFTR
NUTF2	C2	COMP	IFI44	CTSE	ZNF167	NGEF	CRIP1
SPP1	C8orf84	NTM	MRC1	IL1R2	RARA	ASCL2	CTSE
AXIN2	CAB39L	HOPX	IGLV1-36	LRRC26	SNX21	BCL11A	CTTNBP2
PLAGL2	CACHD1	AEBP1	ITM2A	DHRS9	NRXN2	CDCA7	DDC
SEMA5A	CADM1	FRMD6	CXCL9	TPSG1	ARFGAP1	DDC	DUSP4
HOXC6	CALCA	PLN	MSR1	AGR3	PAPLN	GPR160	FLJ32063
ECM2	CAMK2N2	FBN1	IFIT1	AGR2	SMARCC2	PHF16	FSCN1
VAV3	CAPN6	COL11A1	CCRL1	REP15	AS3MT	PRLR	GGH
KCNE4	CAPN8	ANTXR1	IGL@	MLPH	DKFZp547K054	VAV3	HOXD3
RGS4	CCL24	MIR100HG	CCL18	SPDEF	RGN	AIFM3	HSF5
MDM4	CD5	PCDH7	HLA-DQB1	KLK12	CTSF	C13orf18	IRX3
PDGFRL	CD55	DDR2	IGLJ3	HEPACAM2	SORBS1	CDX2	KIAA0802
RNF43	CDC20B	MYL9	IGLV1-40	WFDC2	FCGRT	IHH	KLK10
CFH	CEACAM5	FERMT2	NCF2	HYAL1	LARP6	NR1I2	LOC388199
FITM2	CEACAM6	VCAN	SAMD9L	LGALS9	FHOD3	PCDH21	MIR142
ASCL2	CEL	CDH11	MIR155HG	SLC9A2	NINL	ACE2	MLPH
TRIM7	CELP	SYNPO2	CXCL11	PFKP	SRPX2	C10orf99	OSBP2
CLEC5A	CGNL1	SULF1	ANKRD36BP2	TRIM7	BG114486	LRRC19	PHYH
STON1	CHP2	FAP	FCER1G	TMEM173	THC2669157	PPP1R14D	PIK3AP1
ATP10B	CHST13	COL3A1	C16orf54	RAB27B	QPRT	RPS6KA3	PIWIL1
PRPF39	CORO2A	CTHRC1	FCGR3A	SCNN1A	PLA2G12B	SPINK1	PLCB4
NAP1L3	CRNDE	ADAM12	RSAD2	PALLD	VAV3	CTSL2	PLK2
KHDRBS3	CSRNP3	COL1A2	IGHM	ARHGDIB	PTPRO	FHDC1	PLLP
CCDC80	CST1	TIMP2	SAMSN1	CA9	RNF43	GGH	PPP1R14C
AMFR	CXCR4	PRRX1	CLEC7A	MRAP2	DDC	PITX2	PPP1R14D
ISM1	CYP2S1	BGN	TNFRSF17	L1TD1	AXIN2	PLAGL2	PPPDE2
CDHR1	CYP4X1	GLT8D2	SLAMF7	MMP7	C13orf18	SPAG1	PTPRD
DDC	DACT1	DCN	TLR8	MGST1	TSPAN6	TSPAN6	PTPRO
PLN	DEFA5	FABP4	IFI44L	ATOH1	GGH	ANO9	RASSF6
AMACR	DEFA6	FBLN1	KYNU	IQGAP2	PLAGL2	C20orf112	RBBP8
PRDX5	DEPDC7	EFEMP1	CP	FOXA1	ACSL6	CHMP4B	RBM8A
PCDH7	DKK4	VGLL3	PRKCB	C12orf36	RBP2	CLDN3	REG4
MSRB3	DLX1	SPARC	MS4A4A	OASL	SLC6A4	PPP1R3D	RNF43
CASP2	DMBT1	ITGBL1	HLA-DPA1	GSDMB	CTSL2	PRSS8	RSBN1L
CXCL12	DPEP1	AKAP12	CXCL13	CKAP4	AMACR	RBM39	S100A16
TMEM45A	EDAR	INHBA	IGJ	FHL2	POFUT1	SPATA2	SATB2
PPP1R14D	EMB	COL5A2	POU2AF1	PLA2G3	CEBPA	TH1L	SERPINB5
TREM1	EML1	RAB31	TAGAP	KLF2	PARD6B	YTHDF1	SLC25A37
ZFPM2	EMP1	ISLR	ERAP2	KLF9	PRDX5	ANLN	SOX8
PRR15	EPAS1	STON1	DOCK8	BCAS1	SEPHS2	ASPM	SPINK1
TNFAIP6	ETV1	NOX4	FAM26F	PLA2G2A	C20orf142	BUB1	TM4SF4
VGLL3	F13A1	LOX	IDO1	CAPN9	GPSM2	BUB1B	TNNC2
ZNF827	FABP1	POSTN	IGHG1	F3	SLC5A6	C12orf48	TPK1
MEIS2	FLJ90757	ECM2	AIM2	NGFRAP1	TP53RK	CCNA2	TRNP1
PMEPA1	FLNA	LHFP	CCL5	XK	NCOA6	CCNB2	TSPAN6
PTGIS	FMOD	SERPINF1	MS4A7	MT1E	C20orf111	CDC2	VAV3
FSCN1	FOSL1	NNMT	GPNMB	ST3GAL4	C20orf43	CDT1	ZIC2
COLEC12	FRAS1	PTGIS	IGKV4-1	KLK1	HNF4A	CENPA	ZNF141
HSD17B6	FZD2	MYLK	IGKC	ANGPTL4	PSMA7	CENPE	ZSWIM1
DUSP4	GAD1	MAP1B	SAMD9	SOCS2	VAPB	CEP55	
TSPAN6	GALNT5	CALD1	GNLY	KIT	HNRNPA1L2	DEPDC1B	
PRLR	GATS	GREM1	42795	DLL1	KIF3B	DHFR	
PDGFC	GCNT3	COL5A1	FCGR1B	CAPN8	ARFGEF2	DSCC1	
IHH	GLT25D2	CNN1	LY96	CYB5R2	PIWIL2	ECT2	
HOPX	GPA33	TIMP3	CXCL10	KLF4	FANCF	FEN1	
ULBP2	GPR155	COL6A2	HLA-DRB1	FAM107B	THC2644861	FIGNL1	
PRRX1	GPR161	ZEB1	MNDA	C8orf4	MOCS3	GMNN	
LOC157860	HAS2	PPAPDC1A	IGHA1	FGFBP1	PIGU	HJURP	
AIFM3	HEPH	OLFML2B	KLRC1	FAM46A	CEP250	HOOK1	
CYR61	HMGCS2	HTRA1	TRDV3	RIMS3	IFT52	IQGAP3	
ETV5	HMOX1	CXCL12	PCDHGA4	RARRES3	CXorf56	KIF11	
FAP	HSD17B2	DPYSL3	CDKN2B	LEFTY1	COBLL1	KIF23	
DOK5	HUNK	PDGFC	FGF13	KCNN4	EPOR	KIF4A	
ZNF529	IER5L	COL6A3	C6orf168	QSOX1	MAPRE2	KPNA2	
NR1I2	IFI27	COL1A1	LYVE1	SGPP2	SLC41A1	LMNB1	
SLC5A6	IFI6	MYH11	FHL1	WBP5	KCTD1	LMNB2	
BHLHE41	IGFBP3	AOC3	CCL19	FAM46C	TRIB2	LSM5	
F13A1	IL18	SPARCL1	SOX2	SERPINA1	PLK2	MAD2L1	
FERMT2	IQGAP2	COL12A1	NOV	HES4	RAMP1	MCM2	
NAA15	IRX3	GPNMB	PENK	PHLDA1	LOC388610	MCM3	
GPR126	IRX5	BCAT1	DES	FAM174B	TPM2	MELK	
DACT1	ISM2	PHLDB2	GNAL	CLIC4	CD248	MLF1IP	
AVPR1A	ITPR2	SERPING1	CSRNP3	MT2A	LGALS1	NCAPD2	
DPYD	KCNA2	TPM2	MAMDC2	VSIG2	CRYAB	NEK2	
SYK	KCNJ2	TGFB1I1	TMEM35	MUC4	CXCL12	NUSAP1	
TRNP1	KIAA1199	MITF	LOC100127983	SLC16A9	CLDN5	POLQ	
SPHK1	KLHL5	GPC6	GPM6A	RNF144A	LOC387763	PRC1	
ARL4C	KRT20	NEXN	GREM2	LMO4	BNC2	PSAT1	
SSPN	LAMA2	MMP2	GPM6B	SLC28A2	OBSL1	PTTG1	
ENO2	LEF1	FAM129A	UST	PNMA2	EVL	RFC4	
INHBA	LEMD1	ADAMTS2	TYRP1	SERPINB6	COL6A3	RRM1	
C5orf46	LGR5	FIBIN	RBM24	EIF4E3	THBS2	RRM2	
VCAN	LHX2	TMEM47	COL4A3	ALDH3A1	SPOCK1	SKA2	
NNMT	LMCD1	IGFBP5	NBEA	CA12	COL5A2	SMC4	
VMP1	LOC100128079	TNFAIP6	LMO3	MT1X	FBLN1	TMEM48	
SET	LOC283859	MXRA5	LDB3	SIPA1L2	MGP	TOP2A	
GGH	LOC572558	ARL4C	KCND3	CD163L1	MXRA8	UHRF1	
PIK3AP1	LRP4	EPYC	KIT	NT5E	DCN	ZWINT	
SEC22B	MAOB	COL15A1	BCHE	BIK	AEBP1	HOXA10	
HPSE	MDFIC	LMOD1	FRAS1	LIPG	BASP1	HOXA11	
FRMD6	MGC16703	FN1	NKX2-3	ADM	COL6A1	HOXA13	
DSE	MIR155HG	DPT	C2orf40	WISP3	COL1A2	HOXA5	
ALYREF	MLLT11	GNB4	OMD	NFIC	HTRA1	HOXA7	
FCGR2A	MME	TWIST1	ZC3H12C	LXN	LOXL1	HOXA9	
SYNC	MMP28	SDC2	RGS13	SCGB2A1	COL5A1	CLCA1	
SLC11A1	MOBKL2B	FLRT2	GNAI1	NOXO1	FSTL1	KIAA1324	
PEG3	MPP1	LOXL1	PCDHB16	UGT2B11	RARRES2	MUC2	
TXNDC9	MTMR11	FHL1	GLIS3	EPHB3	MSN	RETNLB	
EPM2AIP1	MTSS1	MAB21L2	ZNF880	TLE1	SPARC	SPINK4	
KIAA1967	MUC17	SSPN	MYOCD	SH3BGRL	PDGFRB	ST6GALNAC1	
C1orf174	MUC2	CTSK	SCD5	BMP2	TUBB6	AGR2	
PRKD1	MX1	WWTR1	CPM	TSPAN13	SERPINF1	AGR3	
EPB41L4B	MYH7B	CYBRD1	SERPINA3	RAMP1	EFHA2	ASRGL1	
ANO9	NEURL1B	CLIP4	FILIP1	UGT2B7	GGTLA1	STYK1	
LMCD1	NFE2	ZEB2	PCOLCE2	AHNAK	LAMC1	TSPAN13	
PDLIM3	NKD1	SYNM	SYNM	C3orf70	ROBO4	CTSE	
CAB39L	NOD2	SNAI2	TMEM163	ALCAM	IGFBP5	IL1R2	
SLC2A3	NOTUM	DES	ANGPTL1	RNASE4	FAM20C	RAB27B	
PLXDC2	NRXN3	IGF1	NRN1	LIMCH1	TSPYL5	REG4	
POSTN	PGM5	TNC	CPNE8	VLDLR	VAMP5	TFAP2A	
CBX5	PHACTR1	GUCY1A3	SYNPO2	ALDH1L1	FBXO17	MT1E	
FHDC1	PHLDB2	GULP1	ZNF304	SAMD5	CLEC11A	MT1F	
TYMS	PLAGL1	TMEM45A	FKBP1B	ZIC2	PDLIM4	MT1G	
LOC339260	PLEKHM3	C3	SCN7A	ENO2	GPSM1	MT1M	
DUSP18	PLK1S1	VCAM1	KLF9	IGFBP2	VWF	MT1X	
ITGAM	PRICKLE2	AHNAK2	TCEAL2	CCNO	WISP1	CXCL5	
KANSL1L	PROX1	ACTG2	LEPR	SCG5	SLIT3	IL6	
ACSL5	PRSS23	KAL1	ABI3BP	TOX2	MC1R	MMP1	
TRIB2	PTCH1	FLNA	DTNA	TMEM171	LAMB2	MMP3	
ANGPTL1	PTK7	CYR61	PCDHB10	RNF186	PCOLCE	PTGS2	
MAP1B	RBMS1	NR3C1	SLC16A14	ANKRD37	GPX7	C5orf13	
RPL22L1	RBP1	DSE	SCG2	PTGER4	COX7A1	CCPG1	
LOC100130275	REEP1	PMP22	PRKAA2	FOXD1	FGFR1	FRY	
ZNF573	REG3A	RBMS1	FABP4	UGT1A10	AK021531	MN1	
UBE2QL1	REG4	SMARCA1	CPA3	TCEAL3	CALD1	ZBTB4	
SRGAP2C	RGS10	MAFB	C4orf7	TMEM211	JAK3	AEBP1	
CYBRD1	RHOU	MAF	PDZRN4	C18orf56	TRO	C1S	
LINC00094	ROR1	QKI	EPHA7	UCP2	TGFB3	COL1A1	
UQCRC2	RUNX2	MMP11	ROR1	PCSK1	C1QTNF6	COL6A3	
POFUT1	SDR16C5	CD109	PTX3	PROM2	DTX3	LAMA4	
C20orf112	SERP2	SRPX	ZNF83	SOSTDC1	NID2	PLEKHO1	
	SERPINE2	EDNRA	BMPR1B	C4BPA	COL18A1	VIM	
	SERPINI1	THBS1	VLDLR	TCEAL8	SLC27A1	ATP10D	
	SH3RF3	SLC2A3	CMAH	GCNT1	JAM2	BASP1	
	SI	CHRDL1	VIP	SYT13	SNRPC	CHST11	
	SLC1A1	APOD	SMPX	ADORA2B	SYNCRIP	CXCL12	
	SLC2A12	RUNX2	CR2	MYEOV	NDUFAB1	EPB41L3	
	SLC7A7	COL14A1	C7	ASB9	RABL3	FZD7	
	SMOC2	GPX3	FGF2	MYADM	XRCC2	GPNMB	
	SOBP	UBE2E2	HSPB6	GDPD3	NDUFA10	ITGB2	
	SOCS2	GEM	EML1	GPC3	PA2G4	KLHL5	
	SORBS2	LY96	IGF1	TFF3	RFC4	LRRC8C	
	SOX2	FAM126A	GATM	C2orf82	ZNF695	LYST	
	SP5	ANK2	AGTR1	PTPRR	PPARA	MPEG1	
	SPINK1	CTGF	ADIPOQ	37681	FBXO5	NRP1	
	SPINK4	SORBS1	CFL2	ENTPD8	LOC338328	RASSF8	
	SRGAP3	RGS2	ATP1A2	CHST4	ANKRD35	SH2B3	
	ST6GALNAC1	C1S	SFRP1	NCALD	KIAA1442	SLCO2B1	
	STBD1	CD36	SYT1	KCNS3	THY1	SRGN	
	SULF1	NRP1	GAP43	CAPN12	FES	TBC1D9	
	SULT1C2	KLHL5	CDH19	GSTA1	PGF	UBE2E2	
	TBC1D9	CFH	SDPR	GLRX	MAP3K3	ALOX5AP	
	TFCP2L1	SPP1	CHRDL2	SORBS2	GPSM3	AQP9	
	TGFBR3	RDX	PLN	CYTH2	NPC2	BCL2A1	
	TMEM150C	ADH1B	OGN	SLAMF6	C14orf139	CCL2	
	TMEM154	CCL2	MMRN1	RHOB	THC2532155	HSD11B1	
	TMEM200A	BASP1	NEFL	PTMS	C1orf198	PAPPA	
	TRIM29	ID4	PEG10	PTER	FLT4	PI15	
	TSPAN5	MDFIC	ABCA8	CXCL16	SNRP70	CCDC88A	
	TTC9	RASSF8	MYH11	MSLN	KIAA1602	ICAM1	
	TUBB2B	C11orf96	SCG5	KRT17	ELMO1	LCP2	
	UBE2L6	TSPAN2	SPON1	AHNAK2	RNF207	NCF2	
	VSNL1	MEIS2	ZNF415	GCNT3	POLE	TRIB2	
	WNT5B	AMIGO2	SORBS2	SERPINE2	CPSF6	DOK3	
	WWTR1	SHISA2	CHGB	KLK6	BCL2L14	FCGR2A	
	ZBTB20	APOE	PRKAR2B	S100A3	TOM1L1	FCGR2B	
	ZBTB7C	C5AR1	PDK4	SERPINB5	ASPM	LAPTM5	
	ZC3H12C	ZCCHC24	RERG	COL17A1	ORC6L	MSR1	
	ZNF362	MS4A7	DCLK1	TM4SF1	ZNF367	SLC11A1	
	ZNF503	DPYD	CD36	KRT6A	NIPSNAP1	SLC43A3	
	ZNF559	PLXNC1	STMN2	MUC17	SPBC25	SPP1	
		CFL2	CNN1	MX1	DIAPH3	APOC1	
		ITGAM	CPE	CLIC3		C1QA	
		SERPINE1	MS4A1	SLPI		C1QB	
		SFRP1	GHR	KLK10		C3AR1	
		DACT1	TUBB2B	KRT80		CD40	
		CLEC2B	MAB21L2	S100A16		CYBB	
		PAPPA	SRPX	ADAM19		FCER1G	
		APOC1	ERAP1	ITGA3		HAVCR2	
		RORA	FOXP2	UCA1		HCK	
		CAV2	PGM5	AFAP1L2		HLA-DMB	
		HDGFRP3	ANK2	TM4SF4		IFI30	
		CCL18	EPB41L3	BMP4		IGSF6	
		ADAMTS1	CLIC6	IGF2BP3		PTAFR	
		TBC1D9	ADH1B	C6orf15		SLAMF8	
		KCNMA1	GPR27	PLA2G16		CCL8	
		SPON1	BEX5	SFTA2		CD53	
		MS4A4A	LOC401093	LEMD1		FAM26F	
		PDZRN3	MT1M	CXCL1		GBP2	
		DMD	JAM2	CFD		GIMAP4	
		ABI3BP	PCDHB14	IFI6		HLA-DPA1	
		CD163	NEGR1	MALL		HLA-DRA	
		ABCA8	CHRDL1	CDR2L		PTPRC	
		TYROBP	CLU	IRF9		TNFSF13B	
		FCGR1B	BEX4	EPSTI1		APOL3	
		NCF2	TNFSF4	LACTB2		C1orf162	
		FCER1G	EFEMP1	S100A4		CD74	
		CXCR4	NTM	WNT11		IL10RA	
		FPR3	NLGN4X	SLC7A7		IL7R	
		LAPTM5	NOX4	SULT2B1		MYO5A	
		PLA1A	MGC24103	TNFRSF6B		PLCG2	
		ANXA1	PDGFRL	DCBLD2		RASSF4	
		STC1	ST6GALNAC5	CALB2		SASH3	
		BEX4	GUCY1A3	LGR6		TMC8	
		WASF3	EDNRA	S100A9		APBB1IP	
		SCRN1	THBS2	IFIT3		CECR1	
		CHI3L1	PDGFC	PRAP1		CXCR4	
		PMEPA1	SCUBE3	TRIM29		CYTIP	
		CPE	BICC1	IRF7		LCP1	
		SOCS3	ETV1	TFAP2C		LSP1	
		BHLHE41	FN1	ETS1		NLRC3	
		EVI2A	ID4	DEFB1		RAC2	
		ALOX5AP	LOC399959	ST3GAL1		APOL1	
		CD14	B3GALNT1	MX2		APOL6	
		TREM1	F13A1	ANXA2P1		BTN3A3	
		ETV1	FNDC1	FOXC1		CXCL10	
		TNFSF13B	CXCL12	HIST1H2AC		CXCL11	
		ITGB2	COLEC12	PMEPA1		CXCL9	
		SLAMF8	RGS4	NRCAM		GBP1	
		CLEC7A	CACNA2D1	HIST1H2BD		HLA-F	
		KLF9	CDO1	TUBB3		IDO1	
		ENPP2	COL8A1	ID3		IFI16	
		NRXN3	SYNC	EPAS1		IRF1	
		RGS1	TSPAN2	GSTO2		NLRC5	
		KRT80	GXYLT2	TRNP1		PARP14	
		TPSAB1	PDE10A	FLNC		PARP9	
		SERPINE2	CYP1B1	HSD17B2		RARRES3	
		KCTD12	RASSF8	LMTK3		SECTM1	
		S100A8	CYBRD1	PODXL		STAT1	
		CDKN2B	LOC401097	ABHD12B		TAP1	
		FCGR3B	STEAP4	NAPRT1		TAP2	
		MS4A6A	ASPN	PPP1R1B		TRIM22	
		CPA3	TFPI	NBL1		CMPK2	
		C1QC	FBN1	CEACAM5		DDX58	
		TPSB2	SPOCK1	PDZK1IP1		IFI44	
		GXYLT2	MN1	CEACAM6		IFI6	
		SRPX2	DIO2	PYGB		IFIH1	
		HSPA6	MEIS1	GGT6		IFIT3	
		ANO1	CDH11	ATP10B		MX1	
		EPDR1	DPYSL3	NCOA7		MX2	
		HCLS1	POSTN	KRT20		OAS2	
		APOLD1	DKK2	LY6G6D		OAS3	
		BCL2A1	RORA	MTMR11		SAMD9	
		SRGN	C5orf46	PYCARD		SAMD9L	
		LY6G6D	DCBLD2	CDH17		XAF1	
		EREG	HMCN1	CES2		BIRC3	
		CEL	PPP1R3C	C2CD4B		DAPP1	
		KRT23	TCEAL7	AUTS2		MAP3K8	
		ACSL6	FAP	MECOM		NFKBIZ	
		QPRT	STON1	SLC26A3		PIK3AP1	
		AXIN2	CCDC80	KRT23		RGS1	
		ABAT	FBLN1	SLC39A8		SP100	
		FARP1	MAP9	HSD11B2		TNFAIP3	
		CELP	RTN1	PSG1		DUSP5	
		C13orf18	TNFAIP6	MUC12		ERRFI1	
		HUNK	PRR16	SYTL2		KLF6	
		PLCB4	HTR2B	IFITM1		MXD1	
		APCDD1	TMEM45A	TGM2		PLAUR	
		RNF43	EPYC	CYP2S1		ATF3	
		ASCL2	DPYD	FLJ32063		C8orf4	
		CHN2	TWIST1	GZMB		FOS	
		AREG	RUNX1T1	NUPR1		JUNB	
		PAH	MEOX2	MAP7D2		NR4A1	
		NR1I2	DPT	PLTP		SIK1	
		FREM2	VIM	QPRT		ZFP36	
		CTTNBP2	SFRP2	FTH1		CCL11	
		GNG4	SLIT2	CDHR1		CH25H	
		PRR15	CD109	C10orf99		EDNRB	
		LOC100288092	CORIN	ADH1A		F2RL2	
		CFTR	KIAA1462	NOX1		FOXF1	
		BCL11A	BAG2	REPS2		FOXF2	
		ERP27	VGLL3	CEACAM7		PCDH18	
		PLA2G12B	PRICKLE1	TXNIP		WNT5A	
		DACH1	MGP	IRF8		ABI3BP	
		SPIN3	NPR3	MT1F		AKAP12	
		GGH	SOX7	ADH1C		ALDH1A3	
		ACE2	PCDH7	SLC30A2		ASPN	
		PTPRO	PLA1A	TNNC2		BNC2	
		DPEP1	ZEB2	MUC20		C10orf10	
		PROX1	IBSP	FOXD2		C1R	
		ZNRF3	CRISPLD1	GDPD5		C20orf194	
		CAB39L	BCAT1	ASCL2		C3	
		LRRC2	SGCE	PSG4		C7	
		REEP1	EPHA3	PAG1		CNRIP1	
		CYP2B6	GREM1	LGALS3		COLEC12	
		LAMP2	COMP	LDHB		DPYSL3	
		PPP1R14C	KAL1	SLC35F2		FBLN1	
		CBX5	PTGIS	CKMT2		FNDC1	
		NOX1	FAM19A5	PID1		GNB5	
		SLC22A3	RHOJ	CTSA		IGFBP5	
		TCFL5	ROBO2	IRS2		MOXD1	
		SATB2	ZEB1	RPRML		PRELP	
		AREGB	BNC2	CMBL		SERPINF1	
		AZGP1	CFH	EDN1		SERPING1	
		TMEM150C	COL10A1	LOC25845		SLIT3	
		LOC647979	MFAP5	EPDR1		SRPX	
		LOC100128822	OSMR	RICH2		SSBP2	
		CES1	FGF7	FXYD3		THBS2	
		[illegible]	[illegible]	[illegible]		[illegible]	

Supplemantary Table 4. Classification of three datasets according to the SFM using K-means algorithm

sample_id	cohort.id	DFS	DFS_status	SFM subtypes
GSM971957	GSE39582	0	1	SFM-C
GSM971979	GSE39582	1590	0	SFM-C
GSM971980	GSE39582	1770	0	SFM-C
GSM971983	GSE39582	2610	0	SFM-C
GSM971988	GSE39582	450	1	SFM-C
GSM971997	GSE39582	2670	0	SFM-C
GSM972002	GSE39582	750	1	SFM-C
GSM972015	GSE39582	2730	0	SFM-C
GSM972016	GSE39582	420	1	SFM-C
GSM972056	GSE39582	2760	0	SFM-C
GSM972058	GSE39582	1800	0	SFM-C
GSM972076	GSE39582	4590	0	SFM-C
GSM972080	GSE39582	1590	1	SFM-C
GSM972114	GSE39582	1380	0	SFM-C
GSM972129	GSE39582	330	0	SFM-C
GSM972168	GSE39582	120	1	SFM-C
GSM972196	GSE39582	2190	0	SFM-C
GSM972214	GSE39582	0	1	SFM-C
GSM972221	GSE39582	1950	0	SFM-C
GSM972222	GSE39582	1710	0	SFM-C
GSM972223	GSE39582	990	0	SFM-C
GSM972226	GSE39582	600	0	SFM-C
GSM972229	GSE39582	0	0	SFM-C
GSM972261	GSE39582	210	0	SFM-C
GSM972264	GSE39582	2190	0	SFM-C
GSM972265	GSE39582	1800	0	SFM-C
GSM972270	GSE39582	1950	0	SFM-C
GSM972276	GSE39582	1290	0	SFM-C
GSM972277	GSE39582	1530	0	SFM-C
GSM972278	GSE39582	1350	0	SFM-C
GSM972280	GSE39582	1380	0	SFM-C
GSM972282	GSE39582	1530	0	SFM-C
GSM972283	GSE39582	1530	0	SFM-C
GSM972285	GSE39582	60	0	SFM-C
GSM972286	GSE39582	1110	0	SFM-C
GSM972292	GSE39582	750	0	SFM-C
GSM972294	GSE39582	750	0	SFM-C
GSM972298	GSE39582	270	0	SFM-C
GSM972305	GSE39582	930	0	SFM-C
GSM972316	GSE39582	2490	0	SFM-C
GSM972317	GSE39582	150	1	SFM-C
GSM972325	GSE39582	1590	1	SFM-C
GSM972332	GSE39582	2610	0	SFM-C
GSM972346	GSE39582	2160	0	SFM-C
GSM972364	GSE39582	3090	0	SFM-C
GSM972391	GSE39582	1290	1	SFM-C
GSM972403	GSE39582	1680	0	SFM-C
GSM972406	GSE39582	3180	0	SFM-C
GSM972408	GSE39582	960	0	SFM-C
GSM972430	GSE39582	1590	0	SFM-C
GSM972445	GSE39582	1050	0	SFM-C
GSM972446	GSE39582	NA	NA	SFM-C
GSM972447	GSE39582	930	0	SFM-C
GSM972448	GSE39582	NA	NA	SFM-C
GSM972452	GSE39582	2970	0	SFM-C
GSM972458	GSE39582	510	0	SFM-C
GSM972465	GSE39582	2520	0	SFM-C
GSM972471	GSE39582	NA	NA	SFM-C
GSM972472	GSE39582	2400	0	SFM-C
GSM972473	GSE39582	1350	1	SFM-C
GSM972488	GSE39582	1470	0	SFM-C
GSM972499	GSE39582	2160	0	SFM-C
GSM972507	GSE39582	1260	0	SFM-C
GSM971958	GSE39582	0	1	SFM-E
GSM971961	GSE39582	30	1	SFM-E
GSM971963	GSE39582	510	1	SFM-E
GSM971966	GSE39582	1890	0	SFM-E
GSM971968	GSE39582	2790	1	SFM-E
GSM971977	GSE39582	1470	0	SFM-E
GSM971981	GSE39582	1440	0	SFM-E
GSM971982	GSE39582	1260	0	SFM-E
GSM971987	GSE39582	2040	0	SFM-E
GSM971999	GSE39582	30	0	SFM-E
GSM972006	GSE39582	0	1	SFM-E
GSM972010	GSE39582	300	1	SFM-E
GSM972019	GSE39582	630	1	SFM-E
GSM972026	GSE39582	960	1	SFM-E
GSM972046	GSE39582	630	1	SFM-E
GSM972047	GSE39582	990	0	SFM-E
GSM972063	GSE39582	2100	0	SFM-E
GSM972064	GSE39582	0	1	SFM-E
GSM972072	GSE39582	5160	0	SFM-E
GSM972073	GSE39582	4890	0	SFM-E
GSM972084	GSE39582	3870	0	SFM-E
GSM972088	GSE39582	2370	0	SFM-E
GSM972092	GSE39582	1410	0	SFM-E
GSM972093	GSE39582	2220	0	SFM-E
GSM972094	GSE39582	1830	0	SFM-E
GSM972095	GSE39582	2010	0	SFM-E
GSM972102	GSE39582	1380	0	SFM-E
GSM972112	GSE39582	960	0	SFM-E
GSM972116	GSE39582	60	1	SFM-E
GSM972117	GSE39582	330	1	SFM-E
GSM972125	GSE39582	420	1	SFM-E
GSM972128	GSE39582	2790	0	SFM-E
GSM972133	GSE39582	810	1	SFM-E
GSM972134	GSE39582	210	1	SFM-E
GSM972140	GSE39582	1680	1	SFM-E
GSM972141	GSE39582	450	1	SFM-E
GSM972146	GSE39582	1980	0	SFM-E
GSM972158	GSE39582	3600	0	SFM-E
GSM972162	GSE39582	2310	0	SFM-E
GSM972164	GSE39582	90	1	SFM-E
GSM972165	GSE39582	1080	1	SFM-E
GSM972170	GSE39582	2190	0	SFM-E
GSM972171	GSE39582	3900	0	SFM-E
GSM972187	GSE39582	270	1	SFM-E
GSM972201	GSE39582	2010	0	SFM-E
GSM972204	GSE39582	120	1	SFM-E
GSM972206	GSE39582	0	1	SFM-E
GSM972208	GSE39582	0	1	SFM-E
GSM972209	GSE39582	0	1	SFM-E
GSM972213	GSE39582	0	1	SFM-E
GSM972220	GSE39582	30	0	SFM-E
GSM972224	GSE39582	600	1	SFM-E
GSM972231	GSE39582	420	0	SFM-E
GSM972232	GSE39582	630	0	SFM-E
GSM972235	GSE39582	210	0	SFM-E
GSM972242	GSE39582	510	0	SFM-E
GSM972251	GSE39582	210	0	SFM-E
GSM972255	GSE39582	150	0	SFM-E
GSM972263	GSE39582	2280	0	SFM-E
GSM972274	GSE39582	1710	0	SFM-E
GSM972279	GSE39582	1560	0	SFM-E
GSM972290	GSE39582	1170	0	SFM-E
GSM972291	GSE39582	300	1	SFM-E
GSM972293	GSE39582	1500	0	SFM-E
GSM972297	GSE39582	1380	0	SFM-E
GSM972301	GSE39582	3150	0	SFM-E
GSM972327	GSE39582	2490	0	SFM-E
GSM972329	GSE39582	2340	0	SFM-E
GSM972330	GSE39582	2580	0	SFM-E
GSM972334	GSE39582	2310	0	SFM-E
GSM972337	GSE39582	1230	0	SFM-E
GSM972352	GSE39582	2220	1	SFM-E
GSM972353	GSE39582	3990	0	SFM-E
GSM972355	GSE39582	510	1	SFM-E
GSM972380	GSE39582	180	1	SFM-E
GSM972400	GSE39582	240	1	SFM-E
GSM972409	GSE39582	3120	0	SFM-E
GSM972412	GSE39582	150	1	SFM-E
GSM972421	GSE39582	660	1	SFM-E
GSM972431	GSE39582	1620	0	SFM-E
GSM972437	GSE39582	0	1	SFM-E
GSM972439	GSE39582	0	1	SFM-E
GSM972440	GSE39582	1560	0	SFM-E
GSM972441	GSE39582	0	1	SFM-E
GSM972459	GSE39582	360	0	SFM-E
GSM972464	GSE39582	2580	0	SFM-E
GSM972470	GSE39582	2550	0	SFM-E
GSM972474	GSE39582	270	1	SFM-E
GSM972482	GSE39582	1260	0	SFM-E
GSM972484	GSE39582	2280	0	SFM-E
GSM972493	GSE39582	1770	0	SFM-E
GSM972494	GSE39582	1710	0	SFM-E
GSM972503	GSE39582	4380	0	SFM-E
GSM972517	GSE39582	960	1	SFM-E
GSM972521	GSE39582	2280	0	SFM-E
GSM972522	GSE39582	2970	0	SFM-E
GSM971959	GSE39582	120	1	SFM-A
GSM971960	GSE39582	2220	0	SFM-A
GSM971967	GSE39582	930	NA	SFM-A
GSM971969	GSE39582	1890	0	SFM-A
GSM971970	GSE39582	2580	0	SFM-A
GSM971976	GSE39582	1080	0	SFM-A
GSM971985	GSE39582	210	0	SFM-A
GSM971989	GSE39582	480	1	SFM-A
GSM971992	GSE39582	600	0	SFM-A
GSM972000	GSE39582	270	1	SFM-A
GSM972001	GSE39582	2190	0	SFM-A
GSM972003	GSE39582	0	1	SFM-A
GSM972004	GSE39582	2670	0	SFM-A
GSM972005	GSE39582	0	1	SFM-A
GSM972012	GSE39582	3030	0	SFM-A
GSM972017	GSE39582	1290	0	SFM-A
GSM972024	GSE39582	1110	1	SFM-A
GSM972027	GSE39582	2820	0	SFM-A
GSM972028	GSE39582	2340	0	SFM-A
GSM972033	GSE39582	1500	0	SFM-A
GSM972034	GSE39582	1500	0	SFM-A
GSM972035	GSE39582	1170	0	SFM-A
GSM972036	GSE39582	1500	0	SFM-A
GSM972037	GSE39582	990	0	SFM-A
GSM972042	GSE39582	1530	0	SFM-A
GSM972044	GSE39582	1260	0	SFM-A
GSM972045	GSE39582	0	1	SFM-A
GSM972049	GSE39582	780	0	SFM-A
GSM972050	GSE39582	570	0	SFM-A
GSM972057	GSE39582	780	0	SFM-A
GSM972061	GSE39582	1290	0	SFM-A
GSM972090	GSE39582	2070	0	SFM-A
GSM972091	GSE39582	1350	1	SFM-A
GSM972096	GSE39582	90	1	SFM-A
GSM972098	GSE39582	450	0	SFM-A
GSM972099	GSE39582	450	0	SFM-A
GSM972100	GSE39582	750	0	SFM-A
GSM972105	GSE39582	960	0	SFM-A
GSM972108	GSE39582	3090	0	SFM-A
GSM972109	GSE39582	1080	0	SFM-A
GSM972110	GSE39582	120	1	SFM-A
GSM972111	GSE39582	390	1	SFM-A
GSM972115	GSE39582	330	1	SFM-A
GSM972118	GSE39582	570	1	SFM-A
GSM972121	GSE39582	1050	0	SFM-A
GSM972122	GSE39582	570	1	SFM-A
GSM972123	GSE39582	2130	0	SFM-A
GSM972126	GSE39582	1890	0	SFM-A
GSM972130	GSE39582	1650	0	SFM-A
GSM972135	GSE39582	120	1	SFM-A
GSM972137	GSE39582	270	1	SFM-A
GSM972138	GSE39582	1350	1	SFM-A
GSM972139	GSE39582	1140	1	SFM-A
GSM972144	GSE39582	3630	0	SFM-A
GSM972149	GSE39582	3960	0	SFM-A
GSM972151	GSE39582	2310	0	SFM-A
GSM972152	GSE39582	1680	0	SFM-A
GSM972169	GSE39582	4920	0	SFM-A
GSM972172	GSE39582	120	1	SFM-A
GSM972173	GSE39582	450	1	SFM-A
GSM972174	GSE39582	2910	0	SFM-A
GSM972175	GSE39582	840	1	SFM-A
GSM972177	GSE39582	600	1	SFM-A
GSM972178	GSE39582	630	1	SFM-A
GSM972180	GSE39582	570	1	SFM-A
GSM972182	GSE39582	690	1	SFM-A
GSM972183	GSE39582	330	1	SFM-A
GSM972185	GSE39582	660	1	SFM-A
GSM972188	GSE39582	330	1	SFM-A
GSM972190	GSE39582	3240	0	SFM-A
GSM972191	GSE39582	3660	0	SFM-A
GSM972193	GSE39582	2190	0	SFM-A
GSM972194	GSE39582	2490	0	SFM-A
GSM972195	GSE39582	3600	0	SFM-A
GSM972197	GSE39582	1500	0	SFM-A
GSM972198	GSE39582	1710	0	SFM-A
GSM972199	GSE39582	1980	0	SFM-A
GSM972212	GSE39582	0	1	SFM-A
GSM972218	GSE39582	0	1	SFM-A
GSM972227	GSE39582	600	0	SFM-A
GSM972228	GSE39582	30	0	SFM-A
GSM972244	GSE39582	420	0	SFM-A
GSM972245	GSE39582	0	0	SFM-A
GSM972247	GSE39582	180	0	SFM-A
GSM972248	GSE39582	420	0	SFM-A
GSM972257	GSE39582	270	1	SFM-A
GSM972258	GSE39582	0	0	SFM-A
GSM972259	GSE39582	870	0	SFM-A
GSM972266	GSE39582	600	0	SFM-A
GSM972268	GSE39582	1440	0	SFM-A
GSM972269	GSE39582	2370	0	SFM-A
GSM972271	GSE39582	390	1	SFM-A
GSM972272	GSE39582	450	0	SFM-A
GSM972273	GSE39582	480	0	SFM-A
GSM972284	GSE39582	810	0	SFM-A
GSM972288	GSE39582	1050	0	SFM-A
GSM972295	GSE39582	1830	0	SFM-A
GSM972300	GSE39582	1230	0	SFM-A
GSM972302	GSE39582	810	0	SFM-A
GSM972303	GSE39582	570	0	SFM-A
GSM972304	GSE39582	900	0	SFM-A
GSM972306	GSE39582	1680	0	SFM-A
GSM972307	GSE39582	2460	0	SFM-A
GSM972309	GSE39582	2850	0	SFM-A
GSM972311	GSE39582	3180	0	SFM-A
GSM972312	GSE39582	2910	0	SFM-A
GSM972314	GSE39582	1380	0	SFM-A
GSM972315	GSE39582	2520	0	SFM-A
GSM972319	GSE39582	2340	0	SFM-A
GSM972320	GSE39582	300	1	SFM-A
GSM972324	GSE39582	2460	0	SFM-A
GSM972326	GSE39582	2550	0	SFM-A
GSM972338	GSE39582	2220	0	SFM-A
GSM972343	GSE39582	1560	0	SFM-A
GSM972350	GSE39582	270	NA	SFM-A
GSM972357	GSE39582	1260	0	SFM-A
GSM972368	GSE39582	2760	0	SFM-A
GSM972370	GSE39582	960	1	SFM-A
GSM972372	GSE39582	990	1	SFM-A
GSM972373	GSE39582	1110	1	SFM-A
GSM972379	GSE39582	2850	0	SFM-A
GSM972381	GSE39582	810	1	SFM-A
GSM972383	GSE39582	2760	0	SFM-A
GSM972386	GSE39582	2700	0	SFM-A
GSM972387	GSE39582	720	1	SFM-A
GSM972388	GSE39582	2490	0	SFM-A
GSM972392	GSE39582	630	1	SFM-A
GSM972396	GSE39582	3210	0	SFM-A
GSM972397	GSE39582	3150	0	SFM-A
GSM972415	GSE39582	240	1	SFM-A
GSM972424	GSE39582	3120	0	SFM-A
GSM972429	GSE39582	1860	0	SFM-A
GSM972436	GSE39582	60	1	SFM-A
GSM972443	GSE39582	1440	0	SFM-A
GSM972450	GSE39582	570	1	SFM-A
GSM972451	GSE39582	2910	0	SFM-A
GSM972453	GSE39582	3000	0	SFM-A
GSM972457	GSE39582	1020	1	SFM-A
GSM972463	GSE39582	NA	NA	SFM-A
GSM972475	GSE39582	2610	0	SFM-A
GSM972477	GSE39582	2430	0	SFM-A
GSM972480	GSE39582	2070	0	SFM-A
GSM972487	GSE39582	2010	0	SFM-A
GSM972495	GSE39582	540	1	SFM-A
GSM972496	GSE39582	4260	0	SFM-A
GSM972502	GSE39582	3930	0	SFM-A
GSM972509	GSE39582	420	0	SFM-A
GSM972510	GSE39582	2580	0	SFM-A
GSM972516	GSE39582	1980	0	SFM-A
GSM971962	GSE39582	2190	0	SFM-F
GSM971996	GSE39582	2280	0	SFM-F
GSM972007	GSE39582	60	0	SFM-F
GSM972008	GSE39582	60	1	SFM-F
GSM972011	GSE39582	540	1	SFM-F
GSM972039	GSE39582	0	1	SFM-F
GSM972051	GSE39582	0	1	SFM-F
GSM972054	GSE39582	780	0	SFM-F
GSM972068	GSE39582	90	1	SFM-F
GSM972082	GSE39582	3570	1	SFM-F
GSM972089	GSE39582	2610	0	SFM-F
GSM972097	GSE39582	360	1	SFM-F
GSM972104	GSE39582	1230	0	SFM-F
GSM972113	GSE39582	540	1	SFM-F
GSM972131	GSE39582	2580	0	SFM-F
GSM972181	GSE39582	390	1	SFM-F
GSM972184	GSE39582	120	1	SFM-F
GSM972186	GSE39582	420	1	SFM-F
GSM972234	GSE39582	300	0	SFM-F
GSM972240	GSE39582	420	0	SFM-F
GSM972262	GSE39582	150	0	SFM-F
GSM972275	GSE39582	90	1	SFM-F
GSM972287	GSE39582	1290	0	SFM-F
GSM972345	GSE39582	1230	1	SFM-F
GSM972349	GSE39582	150	0	SFM-F
GSM972359	GSE39582	360	1	SFM-F
GSM972361	GSE39582	540	1	SFM-F
GSM972365	GSE39582	1410	0	SFM-F
GSM972369	GSE39582	540	1	SFM-F
GSM972401	GSE39582	0	0	SFM-F
GSM972413	GSE39582	480	1	SFM-F
GSM972433	GSE39582	90	1	SFM-F
GSM972438	GSE39582	0	1	SFM-F
GSM972444	GSE39582	150	1	SFM-F
GSM972455	GSE39582	2220	0	SFM-F
GSM972467	GSE39582	2160	0	SFM-F
GSM972476	GSE39582	1920	0	SFM-F
GSM972479	GSE39582	90	0	SFM-F
GSM971964	GSE39582	1050	0	SFM-B
GSM971971	GSE39582	1950	0	SFM-B
GSM971972	GSE39582	2250	0	SFM-B
GSM971973	GSE39582	1380	0	SFM-B
GSM971975	GSE39582	1380	0	SFM-B
GSM971978	GSE39582	3270	0	SFM-B
GSM971984	GSE39582	150	1	SFM-B
GSM971990	GSE39582	360	0	SFM-B
GSM971991	GSE39582	1650	0	SFM-B
GSM971993	GSE39582	1680	0	SFM-B
GSM971994	GSE39582	1290	0	SFM-B
GSM971995	GSE39582	750	0	SFM-B
GSM971998	GSE39582	540	1	SFM-B
GSM972013	GSE39582	1290	0	SFM-B
GSM972014	GSE39582	0	1	SFM-B
GSM972018	GSE39582	3510	0	SFM-B
GSM972020	GSE39582	270	1	SFM-B
GSM972021	GSE39582	150	1	SFM-B
GSM972022	GSE39582	2460	0	SFM-B
GSM972023	GSE39582	180	1	SFM-B
GSM972025	GSE39582	1590	0	SFM-B
GSM972031	GSE39582	1650	0	SFM-B
GSM972032	GSE39582	1800	0	SFM-B
GSM972038	GSE39582	30	0	SFM-B
GSM972040	GSE39582	1890	0	SFM-B
GSM972041	GSE39582	1650	0	SFM-B
GSM972043	GSE39582	0	1	SFM-B
GSM972048	GSE39582	0	1	SFM-B
GSM972052	GSE39582	450	0	SFM-B
GSM972053	GSE39582	870	0	SFM-B
GSM972060	GSE39582	2460	0	SFM-B
GSM972062	GSE39582	0	1	SFM-B
GSM972065	GSE39582	1380	0	SFM-B
GSM972066	GSE39582	0	1	SFM-B
GSM972069	GSE39582	6030	0	SFM-B
GSM972070	GSE39582	2040	0	SFM-B
GSM972071	GSE39582	5490	0	SFM-B
GSM972074	GSE39582	30	1	SFM-B
GSM972075	GSE39582	1320	0	SFM-B
GSM972081	GSE39582	780	0	SFM-B
GSM972083	GSE39582	3810	0	SFM-B
GSM972085	GSE39582	2910	0	SFM-B
GSM972086	GSE39582	1290	0	SFM-B
GSM972087	GSE39582	240	1	SFM-B
GSM972101	GSE39582	630	1	SFM-B
GSM972103	GSE39582	840	1	SFM-B
GSM972106	GSE39582	270	1	SFM-B
GSM972119	GSE39582	750	1	SFM-B
GSM972120	GSE39582	1080	0	SFM-B
GSM972124	GSE39582	2250	0	SFM-B
GSM972132	GSE39582	1830	0	SFM-B
GSM972136	GSE39582	1830	1	SFM-B
GSM972142	GSE39582	60	1	SFM-B
GSM972143	GSE39582	4620	0	SFM-B
GSM972145	GSE39582	4050	0	SFM-B
GSM972147	GSE39582	4200	0	SFM-B
GSM972148	GSE39582	3810	0	SFM-B
GSM972150	GSE39582	3270	0	SFM-B
GSM972153	GSE39582	1980	0	SFM-B
GSM972154	GSE39582	90	1	SFM-B
GSM972155	GSE39582	180	1	SFM-B
GSM972156	GSE39582	3360	0	SFM-B
GSM972157	GSE39582	3540	0	SFM-B
GSM972159	GSE39582	2790	0	SFM-B
GSM972160	GSE39582	1170	1	SFM-B
GSM972161	GSE39582	270	1	SFM-B
GSM972163	GSE39582	1230	0	SFM-B
GSM972166	GSE39582	90	1	SFM-B
GSM972167	GSE39582	1080	1	SFM-B
GSM972189	GSE39582	5760	0	SFM-B
GSM972200	GSE39582	1620	0	SFM-B
GSM972202	GSE39582	1590	0	SFM-B
GSM972203	GSE39582	1620	0	SFM-B
GSM972205	GSE39582	210	1	SFM-B
GSM972207	GSE39582	1020	1	SFM-B
GSM972210	GSE39582	0	1	SFM-B
GSM972211	GSE39582	1710	0	SFM-B
GSM972215	GSE39582	1410	0	SFM-B
GSM972216	GSE39582	0	1	SFM-B
GSM972217	GSE39582	0	1	SFM-B
GSM972219	GSE39582	0	1	SFM-B
GSM972225	GSE39582	1110	0	SFM-B
GSM972230	GSE39582	810	0	SFM-B
GSM972233	GSE39582	450	0	SFM-B
GSM972236	GSE39582	360	0	SFM-B
GSM972237	GSE39582	750	0	SFM-B
GSM972238	GSE39582	570	0	SFM-B
GSM972239	GSE39582	420	0	SFM-B
GSM972241	GSE39582	510	0	SFM-B
GSM972243	GSE39582	60	0	SFM-B
GSM972246	GSE39582	0	0	SFM-B
GSM972249	GSE39582	420	0	SFM-B
GSM972250	GSE39582	360	0	SFM-B
GSM972252	GSE39582	480	0	SFM-B
GSM972253	GSE39582	480	0	SFM-B
GSM972267	GSE39582	2010	0	SFM-B
GSM972281	GSE39582	1680	0	SFM-B
GSM972289	GSE39582	510	0	SFM-B
GSM972296	GSE39582	660	0	SFM-B
GSM972299	GSE39582	1230	0	SFM-B
GSM972308	GSE39582	2760	0	SFM-B
GSM972310	GSE39582	750	0	SFM-B
GSM972313	GSE39582	2730	0	SFM-B
GSM972318	GSE39582	1080	0	SFM-B
GSM972321	GSE39582	270	0	SFM-B
GSM972322	GSE39582	1980	1	SFM-B
GSM972323	GSE39582	2400	0	SFM-B
GSM972328	GSE39582	2340	1	SFM-B
GSM972331	GSE39582	2250	0	SFM-B
GSM972333	GSE39582	2400	0	SFM-B
GSM972335	GSE39582	1380	0	SFM-B
GSM972336	GSE39582	1500	0	SFM-B
GSM972339	GSE39582	2280	0	SFM-B
GSM972340	GSE39582	1440	1	SFM-B
GSM972341	GSE39582	2070	0	SFM-B
GSM972342	GSE39582	840	0	SFM-B
GSM972344	GSE39582	2250	0	SFM-B
GSM972347	GSE39582	210	0	SFM-B
GSM972348	GSE39582	1860	0	SFM-B
GSM972351	GSE39582	720	0	SFM-B
GSM972354	GSE39582	690	1	SFM-B
GSM972356	GSE39582	1590	NA	SFM-B
GSM972358	GSE39582	2430	0	SFM-B
GSM972360	GSE39582	4230	0	SFM-B
GSM972362	GSE39582	2940	0	SFM-B
GSM972363	GSE39582	4200	0	SFM-B
GSM972371	GSE39582	2430	0	SFM-B
GSM972374	GSE39582	2700	0	SFM-B
GSM972382	GSE39582	1110	1	SFM-B
GSM972384	GSE39582	3180	0	SFM-B
GSM972389	GSE39582	2790	0	SFM-B
GSM972395	GSE39582	4410	0	SFM-B
GSM972398	GSE39582	1920	0	SFM-B
GSM972399	GSE39582	2700	0	SFM-B
GSM972404	GSE39582	1440	0	SFM-B
GSM972405	GSE39582	2370	0	SFM-B
GSM972411	GSE39582	3750	0	SFM-B
GSM972414	GSE39582	1590	0	SFM-B
GSM972418	GSE39582	2100	0	SFM-B
GSM972434	GSE39582	570	1	SFM-B
GSM972435	GSE39582	420	1	SFM-B
GSM972442	GSE39582	0	1	SFM-B
GSM972449	GSE39582	570	1	SFM-B
GSM972454	GSE39582	2250	0	SFM-B
GSM972456	GSE39582	0	0	SFM-B
GSM972460	GSE39582	2880	0	SFM-B
GSM972461	GSE39582	2880	0	SFM-B
GSM972462	GSE39582	2640	0	SFM-B
GSM972466	GSE39582	2250	0	SFM-B
GSM972468	GSE39582	720	0	SFM-B
GSM972469	GSE39582	2580	0	SFM-B
GSM972478	GSE39582	2070	0	SFM-B
GSM972483	GSE39582	690	1	SFM-B
GSM972489	GSE39582	2220	0	SFM-B
GSM972490	GSE39582	2220	0	SFM-B
GSM972491	GSE39582	2100	0	SFM-B
GSM972492	GSE39582	2040	0	SFM-B
GSM972497	GSE39582	30	0	SFM-B
GSM972498	GSE39582	570	1	SFM-B
GSM972508	GSE39582	4020	0	SFM-B
GSM972511	GSE39582	1410	0	SFM-B
GSM972512	GSE39582	120	1	SFM-B
GSM972513	GSE39582	390	1	SFM-B
GSM972514	GSE39582	NA	NA	SFM-B
GSM972515	GSE39582	3540	0	SFM-B
GSM972520	GSE39582	1860	0	SFM-B
GSM971965	GSE39582	990	0	SFM-D
GSM971974	GSE39582	1680	0	SFM-D
GSM971986	GSE39582	2040	0	SFM-D
GSM972009	GSE39582	0	1	SFM-D
GSM972029	GSE39582	1080	0	SFM-D
GSM972030	GSE39582	1560	0	SFM-D
GSM972055	GSE39582	2790	0	SFM-D
GSM972059	GSE39582	1770	0	SFM-D
GSM972067	GSE39582	5490	0	SFM-D
GSM972077	GSE39582	780	1	SFM-D
GSM972078	GSE39582	1620	1	SFM-D
GSM972079	GSE39582	5040	0	SFM-D
GSM972107	GSE39582	1800	0	SFM-D
GSM972127	GSE39582	2550	0	SFM-D
GSM972176	GSE39582	1050	1	SFM-D
GSM972179	GSE39582	510	1	SFM-D
GSM972192	GSE39582	4740	0	SFM-D
GSM972254	GSE39582	270	1	SFM-D
GSM972256	GSE39582	390	1	SFM-D
GSM972260	GSE39582	390	0	SFM-D
GSM972366	GSE39582	420	1	SFM-D
GSM972367	GSE39582	2850	0	SFM-D
GSM972375	GSE39582	3510	0	SFM-D
GSM972376	GSE39582	2610	0	SFM-D
GSM972377	GSE39582	2460	0	SFM-D
GSM972378	GSE39582	2580	0	SFM-D
GSM972385	GSE39582	1890	0	SFM-D
GSM972390	GSE39582	180	1	SFM-D
GSM972393	GSE39582	150	1	SFM-D
GSM972394	GSE39582	180	1	SFM-D
GSM972402	GSE39582	0	1	SFM-D
GSM972407	GSE39582	3960	0	SFM-D
GSM972410	GSE39582	2730	0	SFM-D
GSM972416	GSE39582	150	1	SFM-D
GSM972417	GSE39582	4380	0	SFM-D
GSM972419	GSE39582	900	1	SFM-D
GSM972420	GSE39582	180	1	SFM-D
GSM972422	GSE39582	390	1	SFM-D
GSM972423	GSE39582	390	1	SFM-D
GSM972425	GSE39582	1770	0	SFM-D
GSM972426	GSE39582	2430	0	SFM-D
GSM972427	GSE39582	1590	0	SFM-D
GSM972428	GSE39582	1650	0	SFM-D
GSM972432	GSE39582	570	1	SFM-D
GSM972481	GSE39582	1950	0	SFM-D
GSM972485	GSE39582	2250	0	SFM-D
GSM972486	GSE39582	990	0	SFM-D
GSM972500	GSE39582	NA	NA	SFM-D
GSM972501	GSE39582	3180	0	SFM-D
GSM972504	GSE39582	720	1	SFM-D
GSM972505	GSE39582	4320	0	SFM-D
GSM972506	GSE39582	4230	0	SFM-D
GSM972518	GSE39582	450	1	SFM-D
GSM972519	GSE39582	330	1	SFM-D

sample_id	cohort.id	SFM subtypes	dfs_status	dfs_days
GSM358342	GSE14333	SFM-F	1	435.9
GSM358343	GSE14333	SFM-A	0	494.1
GSM358344	GSE14333	SFM-D	0	592.5
GSM358345	GSE14333	SFM-A	0	600.6
GSM358354	GSE14333	SFM-B	0	1923
GSM358355	GSE14333	SFM-D	0	1931.1
GSM358357	GSE14333	SFM-A	0	2015.7
GSM358359	GSE14333	SFM-F	0	2055.3
GSM358362	GSE14333	SFM-A	0	2508
GSM358363	GSE14333	SFM-A	0	2511.9
GSM358364	GSE14333	SFM-F	0	2523.9
GSM358369	GSE14333	SFM-A	NA	NA
GSM358370	GSE14333	SFM-A	0	684
GSM358371	GSE14333	SFM-B	0	432
GSM358372	GSE14333	SFM-B	NA	NA
GSM358373	GSE14333	SFM-B	0	1632
GSM358374	GSE14333	SFM-E	0	1935
GSM358375	GSE14333	SFM-B	0	54
GSM358376	GSE14333	SFM-C	0	1260
GSM358377	GSE14333	SFM-C	0	1695
GSM358378	GSE14333	SFM-B	0	1089
GSM358379	GSE14333	SFM-B	NA	NA
GSM358380	GSE14333	SFM-B	0	1326
GSM358381	GSE14333	SFM-C	0	1563
GSM358382	GSE14333	SFM-E	0	900
GSM358383	GSE14333	SFM-E	0	1317
GSM358384	GSE14333	SFM-D	0	2424
GSM358386	GSE14333	SFM-F	1	180.3
GSM358387	GSE14333	SFM-D	1	298.8
GSM358389	GSE14333	SFM-D	1	427.8
GSM358390	GSE14333	SFM-A	0	451.5
GSM358394	GSE14333	SFM-E	1	580.8
GSM358395	GSE14333	SFM-C	0	660.6
GSM358399	GSE14333	SFM-A	1	756.3
GSM358402	GSE14333	SFM-A	0	795.9
GSM358403	GSE14333	SFM-A	0	799.8
GSM358406	GSE14333	SFM-A	1	829.2
GSM358407	GSE14333	SFM-D	0	858.9
GSM358409	GSE14333	SFM-D	0	876.6
GSM358413	GSE14333	SFM-E	0	1077
GSM358416	GSE14333	SFM-E	0	1119.3
GSM358421	GSE14333	SFM-D	0	1495.2
GSM358422	GSE14333	SFM-C	0	1564.2
GSM358426	GSE14333	SFM-D	0	1740.6
GSM358428	GSE14333	SFM-D	0	1798.8
GSM358439	GSE14333	SFM-E	0	3208.2
GSM358440	GSE14333	SFM-E	0	3323.7
GSM358442	GSE14333	SFM-C	0	363
GSM358443	GSE14333	SFM-D	0	888
GSM358444	GSE14333	SFM-E	0	1110
GSM358445	GSE14333	SFM-B	0	855
GSM358446	GSE14333	SFM-A	0	1689
GSM358447	GSE14333	SFM-C	0	1422
GSM358448	GSE14333	SFM-A	0	405
GSM358449	GSE14333	SFM-A	0	1704
GSM358450	GSE14333	SFM-E	0	1584
GSM358451	GSE14333	SFM-A	0	1359
GSM358452	GSE14333	SFM-E	0	1671
GSM358453	GSE14333	SFM-B	0	1200
GSM358454	GSE14333	SFM-E	1	426
GSM358455	GSE14333	SFM-B	0	123
GSM358456	GSE14333	SFM-B	0	1785
GSM358457	GSE14333	SFM-B	0	1287
GSM358458	GSE14333	SFM-E	1	978
GSM358459	GSE14333	SFM-B	0	939
GSM358460	GSE14333	SFM-B	0	1017
GSM358461	GSE14333	SFM-B	0	1431
GSM358462	GSE14333	SFM-F	0	1146
GSM358463	GSE14333	SFM-C	0	1332
GSM358464	GSE14333	SFM-E	0	1344
GSM358465	GSE14333	SFM-E	0	1710
GSM358466	GSE14333	SFM-A	0	1212
GSM358467	GSE14333	SFM-D	0	156
GSM358468	GSE14333	SFM-E	0	348
GSM358469	GSE14333	SFM-E	0	1752
GSM358470	GSE14333	SFM-E	0	1086
GSM358471	GSE14333	SFM-A	0	1362
GSM358472	GSE14333	SFM-F	0	1527
GSM358473	GSE14333	SFM-A	0	1677
GSM358474	GSE14333	SFM-A	1	429
GSM358475	GSE14333	SFM-F	0	1014
GSM358476	GSE14333	SFM-E	0	1140
GSM358477	GSE14333	SFM-C	0	2226
GSM358478	GSE14333	SFM-F	0	2547
GSM358480	GSE14333	SFM-E	1	47.1
GSM358486	GSE14333	SFM-A	1	249.3
GSM358490	GSE14333	SFM-A	1	441.6
GSM358492	GSE14333	SFM-C	1	567.9
GSM358502	GSE14333	SFM-C	0	787.8
GSM358504	GSE14333	SFM-E	1	827.4
GSM358512	GSE14333	SFM-A	0	1142.1
GSM358513	GSE14333	SFM-D	0	1161.6
GSM358514	GSE14333	SFM-E	1	1231.8
GSM358526	GSE14333	SFM-E	0	1929.9
GSM358527	GSE14333	SFM-E	0	1947.9
GSM358530	GSE14333	SFM-A	0	2230.8
GSM358532	GSE14333	SFM-F	1	2383.8
GSM358533	GSE14333	SFM-A	0	2568.3
GSM358535	GSE14333	SFM-E	0	2688.6
GSM358540	GSE14333	SFM-A	0	927
GSM358541	GSE14333	SFM-F	0	720
GSM358542	GSE14333	SFM-A	0	948
GSM358543	GSE14333	SFM-A	0	1488
GSM358544	GSE14333	SFM-A	1	456
GSM358545	GSE14333	SFM-B	0	726
GSM358546	GSE14333	SFM-E	0	2073
GSM358547	GSE14333	SFM-B	0	2043
GSM358548	GSE14333	SFM-E	1	309
GSM358549	GSE14333	SFM-B	0	1776
GSM358550	GSE14333	SFM-B	0	1842
GSM358551	GSE14333	SFM-E	0	450
GSM358552	GSE14333	SFM-B	0	1734
GSM358553	GSE14333	SFM-F	0	564
GSM358554	GSE14333	SFM-C	1	135
GSM358555	GSE14333	SFM-E	1	1143
GSM358556	GSE14333	SFM-B	0	1212
GSM358557	GSE14333	SFM-A	1	171
GSM358558	GSE14333	SFM-D	0	306
GSM358559	GSE14333	SFM-C	0	2541
GSM358560	GSE14333	SFM-B	0	507
GSM358561	GSE14333	SFM-E	1	285
GSM358562	GSE14333	SFM-B	0	2061
GSM358563	GSE14333	SFM-E	0	1785
GSM358564	GSE14333	SFM-D	0	561
GSM358565	GSE14333	SFM-D	0	2124
GSM358566	GSE14333	SFM-B	1	133.2
GSM358567	GSE14333	SFM-F	1	1200
GSM358568	GSE14333	SFM-D	1	2187
GSM358569	GSE14333	SFM-A	1	540
GSM358570	GSE14333	SFM-F	NA	NA
GSM358573	GSE14333	SFM-E	NA	NA
GSM358575	GSE14333	SFM-E	NA	NA
GSM358582	GSE14333	SFM-E	NA	NA
GSM358591	GSE14333	SFM-F	NA	NA
GSM358599	GSE14333	SFM-A	NA	NA
GSM358600	GSE14333	SFM-A	NA	NA
GSM358608	GSE14333	SFM-C	NA	NA
GSM358611	GSE14333	SFM-E	NA	NA
GSM358612	GSE14333	SFM-B	NA	NA
GSM358613	GSE14333	SFM-B	NA	NA
GSM358614	GSE14333	SFM-D	NA	NA
GSM358615	GSE14333	SFM-A	NA	NA
GSM358616	GSE14333	SFM-E	NA	NA
GSM358617	GSE14333	SFM-E	NA	NA
GSM358618	GSE14333	SFM-B	NA	NA
GSM358619	GSE14333	SFM-B	NA	NA
GSM358620	GSE14333	SFM-B	NA	NA
GSM358621	GSE14333	SFM-F	NA	NA
GSM358622	GSE14333	SFM-A	NA	NA
GSM358623	GSE14333	SFM-B	NA	NA
GSM358624	GSE14333	SFM-A	NA	NA
GSM358625	GSE14333	SFM-F	NA	NA
GSM358626	GSE14333	SFM-B	NA	NA
GSM358627	GSE14333	SFM-E	NA	NA
GSM358628	GSE14333	SFM-C	NA	NA
GSM358629	GSE14333	SFM-A	NA	NA
GSM358630	GSE14333	SFM-F	NA	NA
GSM437093	GSE17536	SFM-A	0	4276.5
GSM437094	GSE17536	SFM-A	0	3681.6
GSM437095	GSE17536	SFM-A	0	868.8
GSM437096	GSE17536	SFM-E	0	3576.3
GSM437097	GSE17536	SFM-B	0	1785.9
GSM437098	GSE17536	SFM-A	1	810.6
GSM437099	GSE17536	SFM-B	0	2468.7
GSM437100	GSE17536	SFM-E	0	3276.3
GSM437101	GSE17536	SFM-B	0	1976.4
GSM437102	GSE17536	SFM-F	0	2523.9
GSM437103	GSE17536	SFM-E	0	494.1
GSM437104	GSE17536	SFM-A	0	2508
GSM437105	GSE17536	SFM-C	0	2282.1
GSM437106	GSE17536	SFM-B	0	2034.6
GSM437107	GSE17536	SFM-B	0	1567.2
GSM437108	GSE17536	SFM-B	0	1923
GSM437109	GSE17536	SFM-A	0	1653.9
GSM437110	GSE17536	SFM-A	0	1733.7
GSM437111	GSE17536	SFM-D	0	1503
GSM437112	GSE17536	SFM-A	0	1313.7
GSM437113	GSE17536	SFM-D	0	592.5
GSM437114	GSE17536	SFM-B	0	109.2
GSM437115	GSE17536	SFM-A	0	718.8
GSM437116	GSE17536	SFM-D	0	600.6
GSM437117	GSE17536	SFM-E	0	3557.4
GSM437118	GSE17536	SFM-C	0	1659.9
GSM437119	GSE17536	SFM-F	1	2809.8
GSM437120	GSE17536	SFM-E	0	3323.7
GSM437121	GSE17536	SFM-A	1	829.2
GSM437122	GSE17536	SFM-B	0	3208.2
GSM437123	GSE17536	SFM-A	NA	0
GSM437124	GSE17536	SFM-E	1	580.8
GSM437125	GSE17536	SFM-E	0	804.6
GSM437126	GSE17536	SFM-C	0	1575
GSM437127	GSE17536	SFM-A	1	271.5
GSM437128	GSE17536	SFM-B	0	2669.7
GSM437129	GSE17536	SFM-B	0	1945.8
GSM437130	GSE17536	SFM-E	1	423.9
GSM437131	GSE17536	SFM-D	0	2558.4
GSM437132	GSE17536	SFM-B	0	795.9
GSM437133	GSE17536	SFM-C	1	1119.3
GSM437134	GSE17536	SFM-D	0	2592.9
GSM437135	GSE17536	SFM-F	0	1677.6
GSM437136	GSE17536	SFM-A	0	2119.5
GSM437137	GSE17536	SFM-B	0	2281.2
GSM437138	GSE17536	SFM-E	0	2373.9
GSM437139	GSE17536	SFM-B	1	671.4
GSM437140	GSE17536	SFM-A	0	2407.5
GSM437141	GSE17536	SFM-B	0	865.8
GSM437142	GSE17536	SFM-A	1	756.3
GSM437143	GSE17536	SFM-C	1	931.8
GSM437144	GSE17536	SFM-E	0	1119.3
GSM437145	GSE17536	SFM-C	1	660.6
GSM437146	GSE17536	SFM-A	0	1897.5
GSM437147	GSE17536	SFM-C	0	725.7
GSM437148	GSE17536	SFM-C	0	1564.2
GSM437149	GSE17536	SFM-E	0	1788
GSM437150	GSE17536	SFM-D	0	1798.8
GSM437151	GSE17536	SFM-A	0	1435.8
GSM437152	GSE17536	SFM-D	0	1401.3
GSM437153	GSE17536	SFM-D	0	1379.7
GSM437154	GSE17536	SFM-D	0	1495.2
GSM437155	GSE17536	SFM-C	0	538.5
GSM437156	GSE17536	SFM-C	0	471.3
GSM437157	GSE17536	SFM-F	0	1177.5
GSM437158	GSE17536	SFM-E	0	1102.5
GSM437159	GSE17536	SFM-E	0	757.2
GSM437160	GSE17536	SFM-C	0	1349.1
GSM437161	GSE17536	SFM-E	0	1077
GSM437162	GSE17536	SFM-A	0	799.8
GSM437163	GSE17536	SFM-D	0	876.6
GSM437164	GSE17536	SFM-D	0	858.9
GSM437165	GSE17536	SFM-A	NA	0
GSM437166	GSE17536	SFM-D	0	996
GSM437167	GSE17536	SFM-B	0	1010.7
GSM437168	GSE17536	SFM-A	0	768.3
GSM437169	GSE17536	SFM-A	1	671.4
GSM437170	GSE17536	SFM-E	0	451.5
GSM437171	GSE17536	SFM-E	0	67.8
GSM437172	GSE17536	SFM-B	0	545.4
GSM437173	GSE17536	SFM-E	0	814.5
GSM437174	GSE17536	SFM-E	1	357
GSM437175	GSE17536	SFM-E	0	3155.1
GSM437176	GSE17536	SFM-C	1	246.3
GSM437177	GSE17536	SFM-B	1	881.7
GSM437178	GSE17536	SFM-C	0	2852.1
GSM437179	GSE17536	SFM-A	0	3369.9
GSM437180	GSE17536	SFM-E	1	638.1
GSM437181	GSE17536	SFM-F	1	482.1
GSM437182	GSE17536	SFM-E	1	832.2
GSM437183	GSE17536	SFM-A	1	441.6
GSM437184	GSE17536	SFM-C	1	160.5
GSM437185	GSE17536	SFM-A	0	2985.3
GSM437186	GSE17536	SFM-B	0	613.2
GSM437187	GSE17536	SFM-E	1	827.4
GSM437188	GSE17536	SFM-E	1	286.8
GSM437189	GSE17536	SFM-E	1	700.2
GSM437190	GSE17536	SFM-E	0	2688.6
GSM437191	GSE17536	SFM-A	0	2568.3
GSM437192	GSE17536	SFM-E	0	1929.9
GSM437193	GSE17536	SFM-B	0	667.5
GSM437194	GSE17536	SFM-B	0	2235.9
GSM437195	GSE17536	SFM-E	0	1772.1
GSM437196	GSE17536	SFM-C	0	1434
GSM437197	GSE17536	SFM-E	1	99.6
GSM437198	GSE17536	SFM-E	0	2162.7
GSM437199	GSE17536	SFM-F	1	745.5
GSM437200	GSE17536	SFM-E	1	1231.8
GSM437201	GSE17536	SFM-F	1	1107.6
GSM437202	GSE17536	SFM-E	0	1947.9
GSM437203	GSE17536	SFM-C	0	1517.7
GSM437204	GSE17536	SFM-F	NA	0
GSM437205	GSE17536	SFM-D	0	1161.6
GSM437206	GSE17536	SFM-B	0	1647
GSM437207	GSE17536	SFM-B	0	1966.5
GSM437208	GSE17536	SFM-E	0	1753.5
GSM437209	GSE17536	SFM-C	0	1780.2
GSM437210	GSE17536	SFM-E	0	807.6
GSM437211	GSE17536	SFM-E	1	257.4
GSM437212	GSE17536	SFM-C	0	787.8
GSM437213	GSE17536	SFM-C	0	1585.8
GSM437214	GSE17536	SFM-A	1	631.2
GSM437215	GSE17536	SFM-D	0	1340.1
GSM437216	GSE17536	SFM-B	0	1107.6
GSM437217	GSE17536	SFM-A	0	1512.9
GSM437218	GSE17536	SFM-E	0	127.2
GSM437219	GSE17536	SFM-A	0	1473.3
GSM437220	GSE17536	SFM-A	0	1142.1
GSM437221	GSE17536	SFM-B	0	1342.2
GSM437222	GSE17536	SFM-E	1	1025.7
GSM437223	GSE17536	SFM-E	1	723.9
GSM437224	GSE17536	SFM-E	0	27.6
GSM437225	GSE17536	SFM-C	0	940.8
GSM437226	GSE17536	SFM-F	0	568.8
GSM437227	GSE17536	SFM-E	1	2575.2
GSM437228	GSE17536	SFM-B	0	957.6
GSM437229	GSE17536	SFM-D	0	599.4
GSM437230	GSE17536	SFM-A	0	156.6
GSM437231	GSE17536	SFM-E	NA	0
GSM437232	GSE17536	SFM-D	0	3519
GSM437233	GSE17536	SFM-E	NA	0
GSM437234	GSE17536	SFM-E	NA	0
GSM437235	GSE17536	SFM-E	NA	0
GSM437236	GSE17536	SFM-A	NA	0
GSM437237	GSE17536	SFM-A	NA	0
GSM437238	GSE17536	SFM-A	0	2478.3
GSM437239	GSE17536	SFM-E	NA	0
GSM437240	GSE17536	SFM-A	NA	0
GSM437241	GSE17536	SFM-A	NA	0
GSM437242	GSE17536	SFM-E	NA	0
GSM437243	GSE17536	SFM-E	1	318.3
GSM437244	GSE17536	SFM-B	NA	0
GSM437245	GSE17536	SFM-E	NA	0
GSM437246	GSE17536	SFM-B	NA	0
GSM437247	GSE17536	SFM-B	NA	0
GSM437248	GSE17536	SFM-B	1	746.4
GSM437249	GSE17536	SFM-B	0	1515.9
GSM437250	GSE17536	SFM-F	NA	0
GSM437251	GSE17536	SFM-F	NA	0
GSM437252	GSE17536	SFM-D	NA	0
GSM437253	GSE17536	SFM-B	NA	0
GSM437254	GSE17536	SFM-C	NA	0
GSM437255	GSE17536	SFM-A	0	1655.7
GSM437256	GSE17536	SFM-D	NA	0
GSM437257	GSE17536	SFM-F	NA	0
GSM437258	GSE17536	SFM-A	NA	0
GSM437259	GSE17536	SFM-A	NA	0
GSM437260	GSE17536	SFM-A	NA	0
GSM437261	GSE17536	SFM-A	1	392.4
GSM437262	GSE17536	SFM-D	NA	0
GSM437263	GSE17536	SFM-E	NA	0
GSM437264	GSE17536	SFM-B	0	298.8
GSM437265	GSE17536	SFM-E	0	268.2
GSM437266	GSE17536	SFM-D	1	519.6
GSM437267	GSE17536	SFM-C	NA	0
GSM437268	GSE17536	SFM-D	NA	0
GSM437269	GSE17536	SFM-B	NA	0
GSM437270	GSE17537	SFM-E	0	1621.479452
GSM437271	GSE17537	SFM-E	0	1917.369863
GSM437272	GSE17537	SFM-D	0	1388.712329
GSM437273	GSE17537	SFM-C	0	1800.986301
GSM437274	GSE17537	SFM-E	1	1247.671233
GSM437275	GSE17537	SFM-A	0	1800.986301
GSM437276	GSE17537	SFM-E	1	1806.90411
GSM437277	GSE17537	SFM-B	0	1712.219178
GSM437278	GSE17537	SFM-A	0	1675.726027
GSM437279	GSE17537	SFM-A	0	1945.972603
GSM437280	GSE17537	SFM-E	0	1642.191781
GSM437281	GSE17537	SFM-F	0	1738.849315
GSM437282	GSE17537	SFM-A	0	1717.150685
GSM437283	GSE17537	SFM-D	0	1793.09589
GSM437284	GSE17537	SFM-E	0	1872
GSM437285	GSE17537	SFM-D	0	1635.287671
GSM437286	GSE17537	SFM-B	1	0
GSM437287	GSE17537	SFM-B	0	54.24657534
GSM437288	GSE17537	SFM-C	0	88.76712329
GSM437289	GSE17537	SFM-B	0	14.79452055
GSM437290	GSE17537	SFM-B	1	881.7534247
GSM437291	GSE17537	SFM-A	0	1628.383562
GSM437292	GSE17537	SFM-B	1	1048.438356
GSM437293	GSE17537	SFM-E	1	0
GSM437294	GSE17537	SFM-C	0	1382.794521
GSM437295	GSE17537	SFM-A	1	0
GSM437296	GSE17537	SFM-A	0	1363.068493
GSM437297	GSE17537	SFM-C	0	234.739726
GSM437298	GSE17537	SFM-A	0	1918.356164
GSM437299	GSE17537	SFM-B	0	2041.643836
GSM437300	GSE17537	SFM-B	0	1504.109589
GSM437301	GSE17537	SFM-F	1	541.4794521
GSM437302	GSE17537	SFM-A	1	0
GSM437303	GSE17537	SFM-A	0	1393.643836
GSM437304	GSE17537	SFM-F	0	2303.013699
GSM437305	GSE17537	SFM-E	1	0
GSM437306	GSE17537	SFM-F	0	648.9863014
GSM437307	GSE17537	SFM-C	0	993.2054795
GSM437308	GSE17537	SFM-E	1	0
GSM437309	GSE17537	SFM-C	0	1409.424658
GSM437310	GSE17537	SFM-A	0	159.7808219
GSM437311	GSE17537	SFM-D	0	1493.260274
GSM437312	GSE17537	SFM-B	0	1220.054795
GSM437313	GSE17537	SFM-C	0	1222.027397
GSM437314	GSE17537	SFM-D	1	116.3835616
GSM437315	GSE17537	SFM-F	0	2113.643836
GSM437316	GSE17537	SFM-B	1	0
GSM437317	GSE17537	SFM-E	1	0
GSM437318	GSE17537	SFM-E	1	0
GSM437319	GSE17537	SFM-E	1	0
GSM437320	GSE17537	SFM-E	1	0
GSM437321	GSE17537	SFM-B	1	0
GSM437322	GSE17537	SFM-B	1	0
GSM437323	GSE17537	SFM-B	0	12.82191781
GSM437324	GSE17537	SFM-A	0	1242.739726
GSM820048	GSE33113	SFM-E	0	2000
GSM820049	GSE33113	SFM-B	0	140
GSM820050	GSE33113	SFM-B	0	62
GSM820051	GSE33113	SFM-E	1	832
GSM820052	GSE33113	SFM-B	0	1175
GSM820053	GSE33113	SFM-B	0	1541
GSM820054	GSE33113	SFM-B	0	1613
GSM820055	GSE33113	SFM-E	0	748
GSM820056	GSE33113	SFM-E	0	1545
GSM820057	GSE33113	SFM-E	0	1175
GSM820058	GSE33113	SFM-B	1	657
GSM820059	GSE33113	SFM-F	0	1137
GSM820060	GSE33113	SFM-E	0	499
GSM820061	GSE33113	SFM-D	0	1470
GSM820062	GSE33113	SFM-B	0	1433
GSM820063	GSE33113	SFM-B	0	2810
GSM820064	GSE33113	SFM-A	0	2868
GSM820065	GSE33113	SFM-E	0	1774
GSM820066	GSE33113	SFM-A	0	1928
GSM820067	GSE33113	SFM-E	1	1060
GSM820068	GSE33113	SFM-C	0	929
GSM820069	GSE33113	SFM-A	0	3550
GSM820070	GSE33113	SFM-A	0	1329
GSM820071	GSE33113	SFM-C	1	204
GSM820072	GSE33113	SFM-F	1	357
GSM820073	GSE33113	SFM-A	0	994
GSM820074	GSE33113	SFM-C	0	1413
GSM820075	GSE33113	SFM-C	0	2157
GSM820076	GSE33113	SFM-F	0	1977
GSM820077	GSE33113	SFM-F	0	1807
GSM820078	GSE33113	SFM-A	0	446
GSM820079	GSE33113	SFM-A	0	1859
GSM820080	GSE33113	SFM-C	0	2273
GSM820081	GSE33113	SFM-E	0	1959
GSM820082	GSE33113	SFM-B	1	1278
GSM820083	GSE33113	SFM-F	1	269
GSM820084	GSE33113	SFM-A	0	3555
GSM820085	GSE33113	SFM-E	0	3599
GSM820086	GSE33113	SFM-A	0	974
GSM820087	GSE33113	SFM-E	0	1473
GSM820088	GSE33113	SFM-E	1	446
GSM820089	GSE33113	SFM-A	0	236
GSM820090	GSE33113	SFM-F	1	332
GSM820091	GSE33113	SFM-E	1	437
GSM820092	GSE33113	SFM-D	1	438
GSM820093	GSE33113	SFM-A	0	1006
GSM820094	GSE33113	SFM-D	0	803
GSM820095	GSE33113	SFM-A	0	2414
GSM820096	GSE33113	SFM-C	0	1174
GSM820097	GSE33113	SFM-E	1	391
GSM820098	GSE33113	SFM-C	0	3065
GSM820099	GSE33113	SFM-F	0	474
GSM820100	GSE33113	SFM-E	0	457
GSM820101	GSE33113	SFM-B	0	1260
GSM820102	GSE33113	SFM-F	0	54
GSM820103	GSE33113	SFM-A	0	1983
GSM820104	GSE33113	SFM-F	0	1667
GSM820105	GSE33113	SFM-C	0	2141
GSM820106	GSE33113	SFM-A	0	1281
GSM820107	GSE33113	SFM-E	1	409
GSM820108	GSE33113	SFM-A	0	56
GSM820109	GSE33113	SFM-F	0	1640
GSM820110	GSE33113	SFM-E	0	971
GSM820111	GSE33113	SFM-A	0	1189
GSM820112	GSE33113	SFM-F	0	1184
GSM820113	GSE33113	SFM-A	0	1785
GSM820114	GSE33113	SFM-C	0	1837
GSM820115	GSE33113	SFM-A	0	1844
GSM820116	GSE33113	SFM-B	1	273
GSM820117	GSE33113	SFM-E	1	538
GSM820118	GSE33113	SFM-C	0	1608
GSM820119	GSE33113	SFM-E	1	634
GSM820120	GSE33113	SFM-A	0	736
GSM820121	GSE33113	SFM-C	0	1378
GSM820122	GSE33113	SFM-C	0	1582
GSM820123	GSE33113	SFM-D	1	447
GSM820124	GSE33113	SFM-E	0	1296
GSM820125	GSE33113	SFM-A	0	893
GSM820126	GSE33113	SFM-C	0	1219
GSM820127	GSE33113	SFM-E	1	200
GSM820128	GSE33113	SFM-E	0	1319
GSM820129	GSE33113	SFM-A	0	344
GSM820130	GSE33113	SFM-A	0	1230
GSM820131	GSE33113	SFM-E	0	1147
GSM820132	GSE33113	SFM-A	0	1042
GSM820133	GSE33113	SFM-E	0	1094
GSM820134	GSE33113	SFM-A	0	1061
GSM820135	GSE33113	SFM-C	NA	50
GSM820136	GSE33113	SFM-E	0	1431
GSM820137	GSE33113	SFM-B	0	1428
GSM929494	GSE37892	SFM-B	0	1681.643836
GSM929495	GSE37892	SFM-B	0	1194.410959
GSM929496	GSE37892	SFM-C	1	162.739726
GSM929497	GSE37892	SFM-A	1	206.1369863
GSM929498	GSE37892	SFM-B	1	1124.383562
GSM929499	GSE37892	SFM-E	1	951.7808219
GSM929500	GSE37892	SFM-E	1	1697.424658
GSM929501	GSE37892	SFM-F	0	1732.931507
GSM929502	GSE37892	SFM-E	1	196.2739726
GSM929503	GSE37892	SFM-F	1	197.260274
GSM929504	GSE37892	SFM-F	1	951.7808219
GSM929505	GSE37892	SFM-D	0	2735.013699
GSM929506	GSE37892	SFM-B	0	1241.753425
GSM929507	GSE37892	SFM-D	0	2564.383562
GSM929508	GSE37892	SFM-F	1	432.9863014
GSM929509	GSE37892	SFM-E	0	2276.383562
GSM929510	GSE37892	SFM-C	0	353.0958904
GSM929511	GSE37892	SFM-D	1	846.2465753
GSM929512	GSE37892	SFM-D	0	968.5479452
GSM929513	GSE37892	SFM-F	0	1241.753425
GSM929514	GSE37892	SFM-A	0	2138.30137
GSM929515	GSE37892	SFM-A	0	1914.410959
GSM929516	GSE37892	SFM-A	0	1671.780822
GSM929517	GSE37892	SFM-B	0	1379.835616
GSM929518	GSE37892	SFM-A	0	454.6849315
GSM929519	GSE37892	SFM-A	1	768.3287671
GSM929520	GSE37892	SFM-A	0	1091.835616
GSM929521	GSE37892	SFM-C	0	1084.931507
GSM929522	GSE37892	SFM-A	0	1068.164384
GSM929523	GSE37892	SFM-E	1	296.8767123
GSM929524	GSE37892	SFM-A	1	582.9041096
GSM929525	GSE37892	SFM-A	1	109.4794521
GSM929526	GSE37892	SFM-A	0	452.7123288
GSM929527	GSE37892	SFM-A	0	764.3835616
GSM929528	GSE37892	SFM-B	0	1598.794521
GSM929529	GSE37892	SFM-A	0	1080.986301
GSM929530	GSE37892	SFM-D	0	1223.013699
GSM929531	GSE37892	SFM-C	0	1336.438356
GSM929532	GSE37892	SFM-B	0	1329.534247
GSM929533	GSE37892	SFM-B	0	1350.246575
GSM929534	GSE37892	SFM-B	0	1370.958904
GSM929535	GSE37892	SFM-B	0	1307.835616
GSM929536	GSE37892	SFM-A	0	1712.219178
GSM929537	GSE37892	SFM-B	0	1330.520548
GSM929538	GSE37892	SFM-B	0	1267.39726
GSM929539	GSE37892	SFM-F	1	377.7534247
GSM929540	GSE37892	SFM-B	0	1921.315068
GSM929541	GSE37892	SFM-A	0	1676.712329
GSM929542	GSE37892	SFM-B	0	1531.726027
GSM929543	GSE37892	SFM-B	0	1264.438356
GSM929544	GSE37892	SFM-E	0	992.2191781
GSM929545	GSE37892	SFM-B	0	1304.876712
GSM929546	GSE37892	SFM-B	1	259.3972603
GSM929547	GSE37892	SFM-B	0	1436.054795
GSM929548	GSE37892	SFM-F	0	2395.726027
GSM929549	GSE37892	SFM-F	0	2617.643836
GSM929550	GSE37892	SFM-A	0	2094.90411
GSM929551	GSE37892	SFM-B	1	1368
GSM929552	GSE37892	SFM-E	0	1424.219178
GSM929553	GSE37892	SFM-E	0	2242.849315
GSM929554	GSE37892	SFM-E	0	1852.273973
GSM929555	GSE37892	SFM-E	0	2030.794521
GSM929556	GSE37892	SFM-B	0	462.5753425
GSM929557	GSE37892	SFM-A	0	886.6849315
GSM929558	GSE37892	SFM-B	0	1763.506849
GSM929559	GSE37892	SFM-F	0	16.76712329
GSM929560	GSE37892	SFM-A	0	1017.863014
GSM929561	GSE37892	SFM-B	0	38.46575342
GSM929562	GSE37892	SFM-F	0	792.9863014
GSM929563	GSE37892	SFM-C	0	804.8219178
GSM929564	GSE37892	SFM-B	0	581.9178082
GSM929565	GSE37892	SFM-A	0	1669.808219
GSM929566	GSE37892	SFM-B	0	1808.876712
GSM929567	GSE37892	SFM-A	0	1501.150685
GSM929568	GSE37892	SFM-A	0	1523.835616
GSM929569	GSE37892	SFM-C	0	2072.219178
GSM929570	GSE37892	SFM-B	0	1193.424658
GSM929571	GSE37892	SFM-A	0	1512.986301
GSM929572	GSE37892	SFM-F	1	360.9863014
GSM929573	GSE37892	SFM-D	0	1410.410959
GSM929574	GSE37892	SFM-D	1	636.1643836
GSM929575	GSE37892	SFM-F	1	542.4657534
GSM929576	GSE37892	SFM-A	1	135.1232877
GSM929577	GSE37892	SFM-D	1	420.1643836
GSM929578	GSE37892	SFM-F	0	985.3150685
GSM929579	GSE37892	SFM-A	0	1661.917808
GSM929580	GSE37892	SFM-B	0	1640.219178
GSM929581	GSE37892	SFM-B	0	1656.986301
GSM929582	GSE37892	SFM-E	0	1912.438356
GSM929583	GSE37892	SFM-B	0	1746.739726
GSM929584	GSE37892	SFM-A	0	1754.630137
GSM929585	GSE37892	SFM-A	1	829.4794521
GSM929586	GSE37892	SFM-B	1	740.7123288
GSM929587	GSE37892	SFM-A	1	1134.246575
GSM929588	GSE37892	SFM-A	0	2125.479452
GSM929589	GSE37892	SFM-B	0	1877.917808
GSM929590	GSE37892	SFM-E	1	543.4520548
GSM929591	GSE37892	SFM-E	1	511.890411
GSM929592	GSE37892	SFM-E	0	1412.383562
GSM929593	GSE37892	SFM-E	1	432
GSM929594	GSE37892	SFM-F	0	1795.068493
GSM929595	GSE37892	SFM-B	0	1779.287671
GSM929596	GSE37892	SFM-B	0	3094.027397
GSM929597	GSE37892	SFM-A	0	1762.520548
GSM929598	GSE37892	SFM-A	0	1645.150685
GSM929599	GSE37892	SFM-E	1	1001.09589
GSM929600	GSE37892	SFM-E	0	2133.369863
GSM929601	GSE37892	SFM-D	0	1827.616438
GSM929602	GSE37892	SFM-B	0	1080.986301
GSM929603	GSE37892	SFM-E	0	1475.506849
GSM929604	GSE37892	SFM-A	0	2779.39726
GSM929605	GSE37892	SFM-C	0	1387.726027
GSM929606	GSE37892	SFM-E	0	1083.945205
GSM929607	GSE37892	SFM-B	0	2824.767123
GSM929608	GSE37892	SFM-E	0	1133.260274
GSM929609	GSE37892	SFM-E	1	201.2054795
GSM929610	GSE37892	SFM-A	1	1140.164384
GSM929611	GSE37892	SFM-E	0	1308.821918
GSM929612	GSE37892	SFM-A	1	979.3972603
GSM929613	GSE37892	SFM-B	0	1396.60274
GSM929614	GSE37892	SFM-F	1	553.3150685
GSM929615	GSE37892	SFM-A	0	2105.753425
GSM929616	GSE37892	SFM-E	0	157.8082192
GSM929617	GSE37892	SFM-C	1	332.3835616
GSM929618	GSE37892	SFM-A	1	599.6712329
GSM929619	GSE37892	SFM-E	0	1861.150685
GSM929620	GSE37892	SFM-F	0	2588.054795
GSM929621	GSE37892	SFM-E	0	2820.821918
GSM929622	GSE37892	SFM-F	1	76.93150685
GSM929623	GSE37892	SFM-A	1	345.2054795

sample_id	cohort.id	os	os_status	dfs	dfs_status	SFM subtypes
TCGA.A6.267	TCGA	503	0	503	0	SFM-C
TCGA.A6.267	TCGA	447	0	447	0	SFM-C
TCGA.A6.380	TCGA	314	0	314	0	SFM-C
TCGA.A6.566	TCGA	306	0	306	0	SFM-C
TCGA.A6.566	TCGA	404	0	404	0	SFM-C
TCGA.A6.665	TCGA	282	0	282	0	SFM-C
TCGA.A6.678	TCGA	354	0	354	0	SFM-C
TCGA.AA.351	TCGA	396	1	NA	NA	SFM-C
TCGA.AA.351	TCGA	31	0	NA	NA	SFM-C
TCGA.AA.352	TCGA	245	0	245	0	SFM-C
TCGA.AA.354	TCGA	30	0	NA	NA	SFM-C
TCGA.AA.355	TCGA	911	0	91	1	SFM-C
TCGA.AA.366	TCGA	1643	0	NA	NA	SFM-C
TCGA.AA.368	TCGA	182	0	182	0	SFM-C
TCGA.AA.371	TCGA	821	0	821	0	SFM-C
TCGA.AA.371	TCGA	579	0	579	0	SFM-C
TCGA.AA.381	TCGA	306	1	NA	NA	SFM-C
TCGA.AA.381	TCGA	1005	0	1005	0	SFM-C
TCGA.AA.382	TCGA	31	0	NA	NA	SFM-C
TCGA.AA.384	TCGA	0	1	NA	NA	SFM-C
TCGA.AA.385	TCGA	1096	0	1096	0	SFM-C
TCGA.AA.386	TCGA	1612	0	1612	0	SFM-C
TCGA.AA.387	TCGA	943	0	943	0	SFM-C
TCGA.AA.394	TCGA	791	0	791	0	SFM-C
TCGA.AA.395	TCGA	730	0	730	0	SFM-C
TCGA.AA.398	TCGA	NA	NA	NA	NA	SFM-C
TCGA.AA.A00	TCGA	1157	0	NA	NA	SFM-C
TCGA.AA.A00	TCGA	913	0	913	0	SFM-C
TCGA.AA.A00	TCGA	549	0	549	0	SFM-C
TCGA.AA.A00	TCGA	30	0	NA	NA	SFM-C
TCGA.AA.A01	TCGA	1064	0	1064	0	SFM-C
TCGA.AA.A01	TCGA	31	0	NA	NA	SFM-C
TCGA.AA.A02	TCGA	NA	NA	NA	NA	SFM-C
TCGA.AA.A02	TCGA	670	1	NA	NA	SFM-C
TCGA.AD.590	TCGA	2	0	NA	NA	SFM-C
TCGA.AD.688	TCGA	1720	0	NA	NA	SFM-C
TCGA.AD.689	TCGA	22	0	NA	NA	SFM-C
TCGA.AD.A5E	TCGA	NA	NA	NA	NA	SFM-C
TCGA.AG.359	TCGA	61	1	NA	NA	SFM-C
TCGA.AG.359	TCGA	366	0	366	0	SFM-C
TCGA.AG.360	TCGA	184	0	184	0	SFM-C
TCGA.AG.A00	TCGA	638	0	638	0	SFM-C
TCGA.AG.A02	TCGA	1885	0	NA	NA	SFM-C
TCGA.AM.582	TCGA	28	0	NA	NA	SFM-C
TCGA.AU.600	TCGA	824	0	NA	NA	SFM-C
TCGA.AY.619	TCGA	423	0	423	0	SFM-C
TCGA.AZ.461	TCGA	1002	0	1002	0	SFM-C
TCGA.AZ.461	TCGA	156	1	156	0	SFM-C
TCGA.AZ.659	TCGA	1503	1	1503	0	SFM-C
TCGA.AZ.660	TCGA	3042	1	2270	1	SFM-C
TCGA.CA.525	TCGA	68	0	68	0	SFM-C
TCGA.CA.671	TCGA	3	0	NA	NA	SFM-C
TCGA.CI.662	TCGA	184	0	NA	NA	SFM-C
TCGA.CK.495	TCGA	2134	1	480	1	SFM-C
TCGA.CK.495	TCGA	475	0	475	0	SFM-C
TCGA.CK.591	TCGA	1561	0	1561	0	SFM-C
TCGA.CK.591	TCGA	643	1	292	1	SFM-C
TCGA.CK.674	TCGA	NA	NA	NA	NA	SFM-C
TCGA.CK.675	TCGA	3780	0	3780	0	SFM-C
TCGA.CM.474	TCGA	184	0	184	0	SFM-C
TCGA.CM.586	TCGA	335	0	335	1	SFM-C
TCGA.CM.617	TCGA	182	0	182	0	SFM-C
TCGA.CM.667	TCGA	150	0	150	0	SFM-C
TCGA.CM.667	TCGA	153	0	153	0	SFM-C
TCGA.D5.653	TCGA	621	0	621	0	SFM-C
TCGA.D5.654	TCGA	491	0	491	0	SFM-C
TCGA.D5.692	TCGA	287	0	287	0	SFM-C
TCGA.D5.693	TCGA	46	0	NA	NA	SFM-C
TCGA.D5.693	TCGA	365	0	365	0	SFM-C
TCGA.DM.A1H	TCGA	4126	0	4126	0	SFM-C
TCGA.EI.650	TCGA	607	0	607	0	SFM-C
TCGA.EI.688	TCGA	262	0	262	0	SFM-C
TCGA.EI.691	TCGA	531	0	531	0	SFM-C
TCGA.F4.685	TCGA	591	0	591	0	SFM-C
TCGA.F5.681	TCGA	16	0	NA	NA	SFM-C
TCGA.G4.630	TCGA	1574	0	1574	0	SFM-C
TCGA.G4.632	TCGA	792	0	789	1	SFM-C
TCGA.G4.658	TCGA	796	0	796	0	SFM-C
TCGA.G4.662	TCGA	1595	0	1595	0	SFM-C
TCGA.QG.A5Z	TCGA	952	0	952	0	SFM-C
TCGA.A6.267	TCGA	424	0	NA	NA	SFM-E
TCGA.A6.267	TCGA	648	0	535	1	SFM-E
TCGA.A6.267	TCGA	846	0	846	0	SFM-E
TCGA.A6.268	TCGA	522	0	522	0	SFM-E
TCGA.A6.268	TCGA	552	0	552	0	SFM-E
TCGA.A6.268	TCGA	424	1	381	1	SFM-E
TCGA.A6.268	TCGA	387	0	387	0	SFM-E
TCGA.A6.380	TCGA	333	0	333	0	SFM-E
TCGA.A6.410	TCGA	442	1	364	1	SFM-E
TCGA.A6.410	TCGA	231	0	231	0	SFM-E
TCGA.A6.566	TCGA	453	0	453	0	SFM-E
TCGA.A6.613	TCGA	348	0	348	0	SFM-E
TCGA.A6.613	TCGA	280	0	280	0	SFM-E
TCGA.A6.614	TCGA	255	0	255	0	SFM-E
TCGA.A6.614	TCGA	368	0	368	0	SFM-E
TCGA.A6.665	TCGA	456	0	456	0	SFM-E
TCGA.A6.678	TCGA	189	0	189	0	SFM-E
TCGA.A6.A56	TCGA	1881	1	1069	1	SFM-E
TCGA.A6.A56	TCGA	1711	1	1678	1	SFM-E
TCGA.A6.A5Z	TCGA	293	0	293	0	SFM-E
TCGA.AA.349	TCGA	31	0	NA	NA	SFM-E
TCGA.AA.350	TCGA	1765	0	NA	NA	SFM-E
TCGA.AA.351	TCGA	212	0	59	1	SFM-E
TCGA.AA.351	TCGA	1186	0	821	1	SFM-E
TCGA.AA.352	TCGA	731	0	NA	NA	SFM-E
TCGA.AA.352	TCGA	NA	NA	NA	NA	SFM-E
TCGA.AA.352	TCGA	0	1	NA	NA	SFM-E
TCGA.AA.353	TCGA	882	0	NA	NA	SFM-E
TCGA.AA.353	TCGA	882	0	882	0	SFM-E
TCGA.AA.354	TCGA	426	0	426	0	SFM-E
TCGA.AA.354	TCGA	1034	0	1034	0	SFM-E
TCGA.AA.354	TCGA	639	0	639	0	SFM-E
TCGA.AA.355	TCGA	396	1	122	1	SFM-E
TCGA.AA.366	TCGA	184	0	184	0	SFM-E
TCGA.AA.367	TCGA	1522	0	1522	0	SFM-E
TCGA.AA.367	TCGA	1430	0	1430	0	SFM-E
TCGA.AA.368	TCGA	335	1	335	0	SFM-E
TCGA.AA.369	TCGA	153	1	NA	NA	SFM-E
TCGA.AA.371	TCGA	NA	NA	NA	NA	SFM-E
TCGA.AA.381	TCGA	NA	NA	NA	NA	SFM-E
TCGA.AA.381	TCGA	761	0	761	0	SFM-E
TCGA.AA.383	TCGA	1186	0	1186	0	SFM-E
TCGA.AA.385	TCGA	0	1	NA	NA	SFM-E
TCGA.AA.385	TCGA	1006	0	NA	NA	SFM-E
TCGA.AA.385	TCGA	0	1	NA	NA	SFM-E
TCGA.AA.385	TCGA	975	0	NA	NA	SFM-E
TCGA.AA.385	TCGA	30	0	NA	NA	SFM-E
TCGA.AA.386	TCGA	945	0	945	0	SFM-E
TCGA.AA.386	TCGA	518	0	518	0	SFM-E
TCGA.AA.386	TCGA	731	0	394	1	SFM-E
TCGA.AA.386	TCGA	822	1	822	0	SFM-E
TCGA.AA.395	TCGA	61	1	NA	NA	SFM-E
TCGA.AA.395	TCGA	1035	0	NA	NA	SFM-E
TCGA.AA.396	TCGA	669	0	669	0	SFM-E
TCGA.AA.397	TCGA	489	0	489	0	SFM-E
TCGA.AA.397	TCGA	397	0	397	0	SFM-E
TCGA.AA.397	TCGA	1036	0	1036	0	SFM-E
TCGA.AA.398	TCGA	580	0	580	0	SFM-E
TCGA.AA.398	TCGA	242	1	NA	NA	SFM-E
TCGA.AA.399	TCGA	822	0	822	0	SFM-E
TCGA.AA.A00	TCGA	578	0	578	0	SFM-E
TCGA.AA.A00	TCGA	1035	0	1035	0	SFM-E
TCGA.AA.A00	TCGA	549	0	NA	NA	SFM-E
TCGA.AA.A00	TCGA	1278	0	1278	0	SFM-E
TCGA.AA.A01	TCGA	457	0	457	0	SFM-E
TCGA.AA.A01	TCGA	457	0	457	0	SFM-E
TCGA.AA.A01	TCGA	334	1	NA	NA	SFM-E
TCGA.AA.A01	TCGA	791	0	791	1	SFM-E
TCGA.AA.A02	TCGA	90	1	NA	NA	SFM-E
TCGA.AA.A02	TCGA	61	1	NA	NA	SFM-E
TCGA.AD.654	TCGA	650	0	650	0	SFM-E
TCGA.AF.269	TCGA	323	0	323	0	SFM-E
TCGA.AF.269	TCGA	412	0	327	1	SFM-E
TCGA.AF.269	TCGA	581	0	581	0	SFM-E
TCGA.AF.411	TCGA	441	0	441	0	SFM-E
TCGA.AF.665	TCGA	355	0	355	0	SFM-E
TCGA.AF.667	TCGA	316	0	279	1	SFM-E
TCGA.AG.357	TCGA	974	0	974	0	SFM-E
TCGA.AG.358	TCGA	215	0	215	0	SFM-E
TCGA.AG.358	TCGA	730	1	730	0	SFM-E
TCGA.AG.358	TCGA	1400	0	1216	1	SFM-E
TCGA.AG.359	TCGA	1035	0	1035	0	SFM-E
TCGA.AG.360	TCGA	608	0	608	0	SFM-E
TCGA.AG.361	TCGA	608	0	608	0	SFM-E
TCGA.AG.373	TCGA	1003	0	1003	0	SFM-E
TCGA.AG.389	TCGA	518	0	518	0	SFM-E
TCGA.AG.389	TCGA	1065	0	1065	0	SFM-E
TCGA.AG.389	TCGA	31	0	NA	NA	SFM-E
TCGA.AG.389	TCGA	1461	0	1461	0	SFM-E
TCGA.AG.390	TCGA	608	0	608	0	SFM-E
TCGA.AG.399	TCGA	853	0	791	1	SFM-E
TCGA.AG.400	TCGA	1096	0	1096	0	SFM-E
TCGA.AG.400	TCGA	427	0	427	0	SFM-E
TCGA.AG.400	TCGA	31	0	NA	NA	SFM-E
TCGA.AG.400	TCGA	518	0	518	0	SFM-E
TCGA.AG.402	TCGA	NA	NA	NA	NA	SFM-E
TCGA.AG.402	TCGA	30	0	NA	NA	SFM-E
TCGA.AG.A00	TCGA	790	0	790	1	SFM-E
TCGA.AG.A01	TCGA	1126	0	1126	0	SFM-E
TCGA.AG.A01	TCGA	943	0	943	0	SFM-E
TCGA.AG.A01	TCGA	1947	0	NA	NA	SFM-E
TCGA.AG.A02	TCGA	1581	1	822	1	SFM-E
TCGA.AG.A02	TCGA	1520	0	NA	NA	SFM-E
TCGA.AG.A02	TCGA	59	1	NA	NA	SFM-E
TCGA.AG.A03	TCGA	1157	0	NA	NA	SFM-E
TCGA.AH.654	TCGA	532	0	532	0	SFM-E
TCGA.AH.664	TCGA	21	0	NA	NA	SFM-E
TCGA.AH.664	TCGA	22	0	NA	NA	SFM-E
TCGA.AU.377	TCGA	NA	NA	NA	NA	SFM-E
TCGA.AY.407	TCGA	496	1	186	1	SFM-E
TCGA.AY.407	TCGA	29	1	29	0	SFM-E
TCGA.AZ.468	TCGA	1977	0	411	1	SFM-E
TCGA.AZ.660	TCGA	368	1	NA	NA	SFM-E
TCGA.AZ.660	TCGA	899	1	899	0	SFM-E
TCGA.BM.619	TCGA	310	0	310	0	SFM-E
TCGA.CA.671	TCGA	8	0	NA	NA	SFM-E
TCGA.CA.671	TCGA	41	0	NA	NA	SFM-E
TCGA.CI.661	TCGA	184	0	NA	NA	SFM-E
TCGA.CI.662	TCGA	NA	NA	NA	NA	SFM-E
TCGA.CI.662	TCGA	144	0	NA	NA	SFM-E
TCGA.CK.494	TCGA	534	0	21	1	SFM-E
TCGA.CK.494	TCGA	3116	0	3116	0	SFM-E
TCGA.CK.495	TCGA	1227	0	1227	0	SFM-E
TCGA.CK.674	TCGA	61	0	61	0	SFM-E
TCGA.CM.474	TCGA	244	0	244	0	SFM-E
TCGA.CM.474	TCGA	214	0	214	0	SFM-E
TCGA.CM.475	TCGA	607	0	607	0	SFM-E
TCGA.CM.534	TCGA	304	0	304	0	SFM-E
TCGA.CM.534	TCGA	335	0	335	0	SFM-E
TCGA.CM.586	TCGA	304	0	304	0	SFM-E
TCGA.CM.616	TCGA	427	0	427	0	SFM-E
TCGA.CM.616	TCGA	487	0	487	0	SFM-E
TCGA.CM.616	TCGA	212	0	212	0	SFM-E
TCGA.CM.617	TCGA	242	0	242	0	SFM-E
TCGA.CM.617	TCGA	212	0	212	0	SFM-E
TCGA.CM.667	TCGA	153	0	153	0	SFM-E
TCGA.CM.667	TCGA	122	0	122	0	SFM-E
TCGA.CM.667	TCGA	122	0	122	0	SFM-E
TCGA.CM.668	TCGA	91	0	91	0	SFM-E
TCGA.D5.553	TCGA	1381	1	722	1	SFM-E
TCGA.D5.553	TCGA	373	0	373	0	SFM-E
TCGA.D5.553	TCGA	596	0	579	1	SFM-E
TCGA.D5.653	TCGA	540	0	540	0	SFM-E
TCGA.D5.653	TCGA	460	0	460	0	SFM-E
TCGA.D5.653	TCGA	543	0	493	1	SFM-E
TCGA.D5.653	TCGA	380	0	380	0	SFM-E
TCGA.D5.654	TCGA	474	0	474	0	SFM-E
TCGA.D5.689	TCGA	229	0	229	0	SFM-E
TCGA.D5.692	TCGA	377	0	377	0	SFM-E
TCGA.D5.692	TCGA	308	0	308	0	SFM-E
TCGA.D5.692	TCGA	435	0	435	0	SFM-E
TCGA.D5.692	TCGA	275	0	275	0	SFM-E
TCGA.D5.692	TCGA	408	0	408	0	SFM-E
TCGA.D5.693	TCGA	346	0	346	0	SFM-E
TCGA.DC.615	TCGA	273	0	273	0	SFM-E
TCGA.DM.A28	TCGA	186	1	186	0	SFM-E
TCGA.DY.A1D	TCGA	734	1	NA	NA	SFM-E
TCGA.EI.650	TCGA	625	0	625	0	SFM-E
TCGA.EI.650	TCGA	517	0	90	1	SFM-E
TCGA.EI.651	TCGA	482	0	482	0	SFM-E
TCGA.EI.651	TCGA	496	0	496	0	SFM-E
TCGA.EI.688	TCGA	328	0	328	0	SFM-E
TCGA.EI.688	TCGA	415	0	295	1	SFM-E
TCGA.EI.700	TCGA	364	0	364	0	SFM-E
TCGA.F4.646	TCGA	407	0	313	1	SFM-E
TCGA.F4.646	TCGA	415	0	415	0	SFM-E
TCGA.F4.680	TCGA	481	0	336	1	SFM-E
TCGA.F4.685	TCGA	16	0	16	0	SFM-E
TCGA.F5.646	TCGA	1141	0	1141	0	SFM-E
TCGA.F5.657	TCGA	392	0	122	1	SFM-E
TCGA.F5.681	TCGA	12	0	NA	NA	SFM-E
TCGA.F5.681	TCGA	979	0	979	0	SFM-E
TCGA.F5.681	TCGA	21	0	NA	NA	SFM-E
TCGA.F5.686	TCGA	23	0	NA	NA	SFM-E
TCGA.F5.686	TCGA	14	0	NA	NA	SFM-E
TCGA.G4.629	TCGA	3275	0	3275	0	SFM-E
TCGA.G4.629	TCGA	715	1	291	1	SFM-E
TCGA.G4.629	TCGA	2268	0	2268	0	SFM-E
TCGA.G4.630	TCGA	2003	1	872	1	SFM-E
TCGA.G4.631	TCGA	1372	0	1372	0	SFM-E
TCGA.G4.631	TCGA	1199	0	1199	0	SFM-E
TCGA.G4.631	TCGA	1093	0	38	1	SFM-E
TCGA.G4.632	TCGA	419	0	419	0	SFM-E
TCGA.G4.662	TCGA	2792	0	2564	1	SFM-E
TCGA.G5.623	TCGA	556	1	92	1	SFM-E
TCGA.G5.657	TCGA	1432	1	1432	0	SFM-E
TCGA.NH.A50	TCGA	588	0	588	0	SFM-E
TCGA.QG.A5Y	TCGA	NA	NA	NA	NA	SFM-E
TCGA.QG.A5Z	TCGA	256	1	256	0	SFM-E
TCGA.A6.565	TCGA	462	0	462	0	SFM-A
TCGA.A6.614	TCGA	356	0	356	0	SFM-A
TCGA.A6.665	TCGA	278	0	278	0	SFM-A
TCGA.AA.348	TCGA	153	1	NA	NA	SFM-A
TCGA.AA.349	TCGA	92	1	92	0	SFM-A
TCGA.AA.350	TCGA	1065	0	NA	NA	SFM-A
TCGA.AA.353	TCGA	1035	0	1035	0	SFM-A
TCGA.AA.354	TCGA	395	0	395	0	SFM-A
TCGA.AA.366	TCGA	212	0	212	0	SFM-A
TCGA.AA.397	TCGA	730	0	730	0	SFM-A
TCGA.AA.A00	TCGA	518	0	NA	NA	SFM-A
TCGA.AA.A00	TCGA	456	0	456	0	SFM-A
TCGA.AA.A01	TCGA	974	0	NA	NA	SFM-A
TCGA.AA.A01	TCGA	31	0	NA	NA	SFM-A
TCGA.AA.A01	TCGA	1065	0	NA	NA	SFM-A
TCGA.AA.A01	TCGA	1126	0	1126	1	SFM-A
TCGA.AA.A02	TCGA	1188	1	1188	0	SFM-A
TCGA.AA.A02	TCGA	1581	0	1581	0	SFM-A
TCGA.AA.A02	TCGA	153	1	NA	NA	SFM-A
TCGA.AA.A02	TCGA	426	1	306	1	SFM-A
TCGA.AA.A02	TCGA	28	0	NA	NA	SFM-A
TCGA.AD.688	TCGA	128	0	NA	NA	SFM-A
TCGA.AD.689	TCGA	24	0	NA	NA	SFM-A
TCGA.AD.696	TCGA	834	0	834	0	SFM-A
TCGA.AF.565	TCGA	257	0	257	0	SFM-A
TCGA.AG.361	TCGA	NA	NA	NA	NA	SFM-A
TCGA.AG.372	TCGA	NA	NA	NA	NA	SFM-A
TCGA.AG.A00	TCGA	424	0	424	0	SFM-A
TCGA.AG.A00	TCGA	183	0	183	0	SFM-A
TCGA.AG.A00	TCGA	700	0	123	1	SFM-A
TCGA.AG.A01	TCGA	485	0	NA	NA	SFM-A
TCGA.AG.A01	TCGA	1096	0	518	1	SFM-A
TCGA.AG.A01	TCGA	276	0	276	0	SFM-A
TCGA.AG.A01	TCGA	31	0	NA	NA	SFM-A
TCGA.AG.A01	TCGA	365	0	NA	NA	SFM-A
TCGA.AG.A02	TCGA	334	0	NA	NA	SFM-A
TCGA.AG.A02	TCGA	1185	1	578	1	SFM-A
TCGA.AG.A02	TCGA	1247	0	NA	NA	SFM-A
TCGA.AH.654	TCGA	15	0	NA	NA	SFM-A
TCGA.AH.689	TCGA	804	0	804	0	SFM-A
TCGA.AY.554	TCGA	479	0	479	0	SFM-A
TCGA.AY.638	TCGA	360	0	360	0	SFM-A
TCGA.AY.A54	TCGA	525	0	396	1	SFM-A
TCGA.AZ.431	TCGA	1532	0	1532	0	SFM-A
TCGA.AZ.461	TCGA	172	1	172	0	SFM-A
TCGA.AZ.468	TCGA	2549	0	2549	0	SFM-A
TCGA.AZ.540	TCGA	1868	0	1868	0	SFM-A
TCGA.AZ.659	TCGA	206	1	206	0	SFM-A
TCGA.AZ.660	TCGA	357	1	357	0	SFM-A
TCGA.AZ.660	TCGA	49	1	49	0	SFM-A
TCGA.CA.525	TCGA	93	0	93	0	SFM-A
TCGA.CA.579	TCGA	99	0	99	0	SFM-A
TCGA.CA.671	TCGA	2	0	NA	NA	SFM-A
TCGA.CI.662	TCGA	212	0	NA	NA	SFM-A
TCGA.CK.591	TCGA	NA	NA	NA	NA	SFM-A
TCGA.CL.591	TCGA	218	0	218	0	SFM-A
TCGA.CM.474	TCGA	699	0	699	0	SFM-A
TCGA.CM.475	TCGA	244	0	244	0	SFM-A
TCGA.D5.653	TCGA	555	0	555	0	SFM-A
TCGA.D5.653	TCGA	146	1	123	1	SFM-A
TCGA.D5.653	TCGA	521	0	521	0	SFM-A
TCGA.DM.A0X	TCGA	3641	0	3641	0	SFM-A
TCGA.DM.A0X	TCGA	743	1	484	1	SFM-A
TCGA.DM.A1D	TCGA	3974	0	3974	0	SFM-A
TCGA.DM.A1D	TCGA	2821	1	1920	1	SFM-A
TCGA.DM.A1D	TCGA	1518	1	1518	0	SFM-A
TCGA.DM.A1D	TCGA	405	1	80	1	SFM-A
TCGA.DM.A1D	TCGA	383	1	383	0	SFM-A
TCGA.DM.A1D	TCGA	4270	0	4270	0	SFM-A
TCGA.DM.A1D	TCGA	228	1	228	0	SFM-A
TCGA.DM.A1D	TCGA	1348	1	1348	0	SFM-A
TCGA.DM.A1H	TCGA	4000	0	4000	0	SFM-A
TCGA.DM.A28	TCGA	236	1	236	0	SFM-A
TCGA.DM.A28	TCGA	4233	0	4233	0	SFM-A
TCGA.DM.A28	TCGA	427	1	320	1	SFM-A
TCGA.DM.A28	TCGA	1849	1	1849	0	SFM-A
TCGA.DM.A28	TCGA	3561	0	484	1	SFM-A
TCGA.DM.A28	TCGA	2895	0	2895	0	SFM-A
TCGA.DY.A1D	TCGA	1741	1	1741	0	SFM-A
TCGA.DY.A1H	TCGA	992	1	992	0	SFM-A
TCGA.EF.583	TCGA	15	0	NA	NA	SFM-A
TCGA.EI.651	TCGA	556	0	556	0	SFM-A
TCGA.EI.688	TCGA	350	0	350	0	SFM-A
TCGA.F4.680	TCGA	1024	0	1024	0	SFM-A
TCGA.F5.686	TCGA	34	0	NA	NA	SFM-A
TCGA.G4.630	TCGA	1631	0	859	1	SFM-A
TCGA.G4.630	TCGA	1359	0	1359	0	SFM-A
TCGA.G4.630	TCGA	1674	0	1674	0	SFM-A
TCGA.G4.631	TCGA	1117	0	1117	0	SFM-A
TCGA.G4.631	TCGA	1095	0	592	1	SFM-A
TCGA.G4.632	TCGA	804	0	804	0	SFM-A
TCGA.G4.632	TCGA	672	0	672	0	SFM-A
TCGA.G4.658	TCGA	1089	0	1089	0	SFM-A
TCGA.G4.662	TCGA	1422	1	1422	0	SFM-A
TCGA.G5.623	TCGA	1696	0	1042	1	SFM-A
TCGA.NH.A50	TCGA	553	0	553	0	SFM-A
TCGA.QG.A5Y	TCGA	1301	0	1301	0	SFM-A
TCGA.QG.A5Y	TCGA	1003	0	1003	0	SFM-A
TCGA.A6.267	TCGA	523	0	523	0	SFM-F
TCGA.A6.268	TCGA	464	0	464	0	SFM-F
TCGA.A6.380	TCGA	279	0	279	0	SFM-F
TCGA.A6.381	TCGA	247	0	247	0	SFM-F
TCGA.A6.566	TCGA	362	0	183	1	SFM-F
TCGA.A6.664	TCGA	328	0	328	0	SFM-F
TCGA.A6.665	TCGA	259	0	259	0	SFM-F
TCGA.A6.665	TCGA	399	0	399	0	SFM-F
TCGA.A6.678	TCGA	293	0	293	0	SFM-F
TCGA.A6.A56	TCGA	494	1	439	1	SFM-F
TCGA.A6.A56	TCGA	758	1	257	1	SFM-F
TCGA.AA.348	TCGA	214	1	NA	NA	SFM-F
TCGA.AA.355	TCGA	546	0	546	0	SFM-F
TCGA.AA.368	TCGA	NA	NA	NA	NA	SFM-F
TCGA.AA.369	TCGA	NA	NA	NA	NA	SFM-F
TCGA.AA.371	TCGA	579	1	153	1	SFM-F
TCGA.AA.383	TCGA	485	0	485	0	SFM-F
TCGA.AA.387	TCGA	912	0	912	0	SFM-F
TCGA.AA.387	TCGA	NA	NA	NA	NA	SFM-F
TCGA.AA.396	TCGA	61	0	NA	NA	SFM-F
TCGA.AA.A00	TCGA	122	1	NA	NA	SFM-F
TCGA.AA.A00	TCGA	822	0	822	0	SFM-F
TCGA.AA.A01	TCGA	943	0	943	0	SFM-F
TCGA.AA.A01	TCGA	1158	1	762	1	SFM-F
TCGA.AD.689	TCGA	176	1	176	0	SFM-F
TCGA.AD.690	TCGA	682	1	468	1	SFM-F
TCGA.AD.696	TCGA	331	1	325	1	SFM-F
TCGA.AF.268	TCGA	595	0	595	0	SFM-F
TCGA.AF.269	TCGA	358	0	358	0	SFM-F
TCGA.AF.340	TCGA	387	0	387	0	SFM-F
TCGA.AG.357	TCGA	365	0	365	0	SFM-F
TCGA.AG.390	TCGA	NA	NA	NA	NA	SFM-F
TCGA.AH.654	TCGA	44	0	NA	NA	SFM-F
TCGA.AY.619	TCGA	6	0	NA	NA	SFM-F
TCGA.AZ.432	TCGA	43	1	43	0	SFM-F
TCGA.AZ.660	TCGA	159	1	159	0	SFM-F
TCGA.AZ.660	TCGA	97	1	97	0	SFM-F
TCGA.CA.671	TCGA	10	0	NA	NA	SFM-F
TCGA.CM.534	TCGA	393	0	393	0	SFM-F
TCGA.CM.616	TCGA	212	0	212	0	SFM-F
TCGA.CM.616	TCGA	122	0	122	0	SFM-F
TCGA.CM.616	TCGA	181	0	181	0	SFM-F
TCGA.CM.616	TCGA	243	0	243	0	SFM-F
TCGA.D5.652	TCGA	614	0	386	1	SFM-F
TCGA.D5.653	TCGA	1316	0	1316	0	SFM-F
TCGA.D5.692	TCGA	354	0	354	0	SFM-F
TCGA.DC.615	TCGA	212	0	212	0	SFM-F
TCGA.DC.615	TCGA	216	0	216	0	SFM-F
TCGA.DT.526	TCGA	161	0	161	0	SFM-F
TCGA.EI.700	TCGA	257	0	257	0	SFM-F
TCGA.F4.645	TCGA	262	1	262	0	SFM-F
TCGA.F4.646	TCGA	338	1	288	1	SFM-F
TCGA.F4.656	TCGA	409	0	409	0	SFM-F
TCGA.F4.657	TCGA	188	1	188	0	SFM-F
TCGA.F4.670	TCGA	1091	0	1091	0	SFM-F
TCGA.F4.670	TCGA	47	0	NA	NA	SFM-F
TCGA.F4.680	TCGA	496	0	496	0	SFM-F
TCGA.F4.680	TCGA	494	0	494	0	SFM-F
TCGA.F4.680	TCGA	403	1	403	0	SFM-F
TCGA.F4.685	TCGA	1077	0	1077	0	SFM-F
TCGA.F4.685	TCGA	NA	NA	NA	NA	SFM-F
TCGA.F5.646	TCGA	303	1	303	0	SFM-F
TCGA.F5.670	TCGA	869	1	368	1	SFM-F
TCGA.F5.681	TCGA	20	0	NA	NA	SFM-F
TCGA.G4.629	TCGA	2506	0	439	1	SFM-F
TCGA.G4.630	TCGA	2047	1	2047	0	SFM-F
TCGA.G4.662	TCGA	1434	0	1003	1	SFM-F
TCGA.A6.267	TCGA	541	0	541	0	SFM-B
TCGA.A6.267	TCGA	437	0	437	0	SFM-B
TCGA.A6.565	TCGA	286	0	286	0	SFM-B
TCGA.A6.566	TCGA	342	0	342	0	SFM-B
TCGA.A6.566	TCGA	362	0	362	0	SFM-B
TCGA.A6.566	TCGA	320	0	320	0	SFM-B
TCGA.A6.664	TCGA	344	0	344	0	SFM-B
TCGA.AA.349	TCGA	31	0	NA	NA	SFM-B
TCGA.AA.349	TCGA	1127	0	1127	0	SFM-B
TCGA.AA.350	TCGA	1915	0	1915	0	SFM-B
TCGA.AA.351	TCGA	276	0	276	0	SFM-B
TCGA.AA.352	TCGA	1127	0	1127	0	SFM-B
TCGA.AA.352	TCGA	1096	0	1096	0	SFM-B
TCGA.AA.353	TCGA	580	0	580	0	SFM-B
TCGA.AA.353	TCGA	791	0	791	0	SFM-B
TCGA.AA.355	TCGA	730	0	730	0	SFM-B
TCGA.AA.355	TCGA	700	0	NA	NA	SFM-B
TCGA.AA.356	TCGA	608	0	608	0	SFM-B
TCGA.AA.356	TCGA	424	0	424	0	SFM-B
TCGA.AA.356	TCGA	608	0	NA	NA	SFM-B
TCGA.AA.365	TCGA	1856	0	1856	0	SFM-B
TCGA.AA.366	TCGA	2375	0	2375	0	SFM-B
TCGA.AA.366	TCGA	61	1	NA	NA	SFM-B
TCGA.AA.367	TCGA	457	0	457	0	SFM-B
TCGA.AA.368	TCGA	578	0	578	0	SFM-B
TCGA.AA.369	TCGA	1095	1	396	1	SFM-B
TCGA.AA.369	TCGA	NA	NA	NA	NA	SFM-B
TCGA.AA.369	TCGA	2587	0	NA	NA	SFM-B
TCGA.AA.381	TCGA	30	1	NA	NA	SFM-B
TCGA.AA.383	TCGA	547	0	547	0	SFM-B
TCGA.AA.384	TCGA	1126	0	396	1	SFM-B
TCGA.AA.384	TCGA	454	0	365	1	SFM-B
TCGA.AA.384	TCGA	518	0	NA	NA	SFM-B
TCGA.AA.385	TCGA	945	0	945	0	SFM-B
TCGA.AA.386	TCGA	914	0	914	0	SFM-B
TCGA.AA.386	TCGA	914	0	914	0	SFM-B
TCGA.AA.387	TCGA	549	0	NA	NA	SFM-B
TCGA.AA.393	TCGA	61	1	61	0	SFM-B
TCGA.AA.393	TCGA	395	0	NA	NA	SFM-B
TCGA.AA.394	TCGA	730	0	NA	NA	SFM-B
TCGA.AA.394	TCGA	1004	0	1004	0	SFM-B
TCGA.AA.395	TCGA	638	0	638	0	SFM-B
TCGA.AA.397	TCGA	1096	0	1096	0	SFM-B
TCGA.AA.397	TCGA	1551	0	1216	1	SFM-B
TCGA.AA.397	TCGA	791	0	791	0	SFM-B
TCGA.AA.397	TCGA	761	0	761	0	SFM-B
TCGA.AA.398	TCGA	822	0	822	0	SFM-B
TCGA.AA.398	TCGA	822	0	NA	NA	SFM-B
TCGA.AA.A00	TCGA	518	0	518	0	SFM-B
TCGA.AA.A00	TCGA	669	0	669	0	SFM-B
TCGA.AA.A01	TCGA	365	0	365	0	SFM-B
TCGA.AA.A01	TCGA	943	0	943	0	SFM-B
TCGA.AA.A02	TCGA	1216	0	1216	0	SFM-B
TCGA.AA.A02	TCGA	1247	0	1005	1	SFM-B
TCGA.AD.696	TCGA	6	0	NA	NA	SFM-B
TCGA.AD.A5E	TCGA	500	0	500	0	SFM-B
TCGA.AF.391	TCGA	537	0	537	0	SFM-B
TCGA.AF.391	TCGA	316	1	316	0	SFM-B
TCGA.AF.613	TCGA	468	0	199	1	SFM-B
TCGA.AG.357	TCGA	1096	1	NA	NA	SFM-B
TCGA.AG.358	TCGA	244	0	244	0	SFM-B
TCGA.AG.358	TCGA	1096	1	NA	NA	SFM-B
TCGA.AG.358	TCGA	610	1	NA	NA	SFM-B
TCGA.AG.358	TCGA	31	0	NA	NA	SFM-B
TCGA.AG.359	TCGA	NA	NA	NA	NA	SFM-B
TCGA.AG.359	TCGA	NA	NA	NA	NA	SFM-B
TCGA.AG.359	TCGA	1522	0	1522	0	SFM-B
TCGA.AG.360	TCGA	NA	NA	NA	NA	SFM-B
TCGA.AG.360	TCGA	NA	NA	NA	NA	SFM-B
TCGA.AG.360	TCGA	30	0	NA	NA	SFM-B
TCGA.AG.360	TCGA	485	0	485	0	SFM-B
TCGA.AG.374	TCGA	30	0	NA	NA	SFM-B
TCGA.AG.388	TCGA	1124	0	1064	1	SFM-B
TCGA.AG.389	TCGA	396	0	396	0	SFM-B
TCGA.AG.389	TCGA	426	0	426	0	SFM-B
TCGA.AG.401	TCGA	NA	NA	NA	NA	SFM-B
TCGA.AG.A01	TCGA	2130	0	NA	NA	SFM-B
TCGA.AG.A03	TCGA	3562	0	3562	0	SFM-B
TCGA.AH.690	TCGA	55	0	NA	NA	SFM-B
TCGA.AM.582	TCGA	14	0	NA	NA	SFM-B
TCGA.AZ.431	TCGA	1776	0	1776	0	SFM-B
TCGA.AZ.540	TCGA	1910	1	304	1	SFM-B
TCGA.CA.525	TCGA	65	0	65	0	SFM-B
TCGA.CA.579	TCGA	100	0	100	0	SFM-B
TCGA.CI.662	TCGA	285	0	NA	NA	SFM-B
TCGA.CK.591	TCGA	1493	1	1493	0	SFM-B
TCGA.CK.591	TCGA	304	0	NA	NA	SFM-B
TCGA.CK.674	TCGA	89	0	89	0	SFM-B
TCGA.CL.495	TCGA	377	0	377	0	SFM-B
TCGA.CL.591	TCGA	2376	0	2376	0	SFM-B
TCGA.CM.475	TCGA	123	0	123	0	SFM-B
TCGA.CM.534	TCGA	273	0	273	0	SFM-B
TCGA.CM.586	TCGA	304	0	304	0	SFM-B
TCGA.CM.586	TCGA	153	1	31	1	SFM-B
TCGA.CM.586	TCGA	61	0	61	0	SFM-B
TCGA.CM.586	TCGA	426	0	426	0	SFM-B
TCGA.CM.616	TCGA	212	0	212	0	SFM-B
TCGA.CM.616	TCGA	424	0	424	0	SFM-B
TCGA.CM.667	TCGA	153	0	153	0	SFM-B
TCGA.D5.554	TCGA	411	0	411	0	SFM-B
TCGA.D5.554	TCGA	350	0	350	0	SFM-B
TCGA.D5.653	TCGA	40	0	NA	NA	SFM-B
TCGA.DC.474	TCGA	335	0	335	0	SFM-B
TCGA.DC.533	TCGA	304	0	304	0	SFM-B
TCGA.DC.586	TCGA	153	0	153	0	SFM-B
TCGA.DC.615	TCGA	850	0	850	0	SFM-B
TCGA.DC.616	TCGA	608	0	608	0	SFM-B
TCGA.DC.668	TCGA	122	0	122	0	SFM-B
TCGA.DC.668	TCGA	153	0	153	0	SFM-B
TCGA.DM.A0X	TCGA	1162	1	1162	0	SFM-B
TCGA.DY.A0X	TCGA	3846	0	3846	0	SFM-B
TCGA.DY.A1D	TCGA	1258	1	630	1	SFM-B
TCGA.DY.A1D	TCGA	3932	0	3316	1	SFM-B
TCGA.EF.583	TCGA	73	0	NA	NA	SFM-B
TCGA.EI.650	TCGA	636	0	636	0	SFM-B
TCGA.EI.651	TCGA	538	0	538	0	SFM-B
TCGA.EI.651	TCGA	497	0	497	0	SFM-B
TCGA.G4.629	TCGA	858	1	517	1	SFM-B
TCGA.G4.629	TCGA	254	0	254	0	SFM-B
TCGA.A6.267	TCGA	595	0	595	0	SFM-D
TCGA.A6.268	TCGA	472	0	300	1	SFM-D
TCGA.AA.351	TCGA	31	0	NA	NA	SFM-D
TCGA.AA.352	TCGA	NA	NA	NA	NA	SFM-D
TCGA.AA.366	TCGA	426	0	426	0	SFM-D
TCGA.AA.367	TCGA	28	0	NA	NA	SFM-D
TCGA.AA.381	TCGA	1066	0	762	1	SFM-D
TCGA.AA.384	TCGA	1124	0	1124	0	SFM-D
TCGA.AA.384	TCGA	306	1	NA	NA	SFM-D
TCGA.AA.A00	TCGA	424	0	424	0	SFM-D
TCGA.AA.A01	TCGA	1065	0	608	1	SFM-D
TCGA.AA.A01	TCGA	1005	0	1005	0	SFM-D
TCGA.AA.A03	TCGA	549	1	NA	NA	SFM-D
TCGA.AA.A03	TCGA	1246	0	1246	0	SFM-D
TCGA.AG.372	TCGA	243	0	243	0	SFM-D
TCGA.AG.372	TCGA	30	0	NA	NA	SFM-D
TCGA.AG.372	TCGA	912	0	NA	NA	SFM-D
TCGA.AG.387	TCGA	30	0	NA	NA	SFM-D
TCGA.AG.388	TCGA	NA	NA	NA	NA	SFM-D
TCGA.AG.388	TCGA	608	0	608	0	SFM-D
TCGA.AG.388	TCGA	31	0	NA	NA	SFM-D
TCGA.AG.388	TCGA	NA	NA	NA	NA	SFM-D
TCGA.AZ.430	TCGA	2048	0	2048	0	SFM-D
TCGA.DC.615	TCGA	225	0	225	0	SFM-D

sample_id	cohort.id	SFM subtype	gender	age	n	T.stage	N.stage	M.stage	TNM.stage
RJ014C	Renji.cohort	SFM-A	male	69	rectum	T3	N0	M0	II
RJ030C	Renji.cohort	SFM-A	female	51	rectum	NA	NA	NA	NA
RJ043C	Renji.cohort	SFM-A	female	51	rectum	T4	aN	OMO	II
RJ062C	Renji.cohort	SFM-A	female	67	ascendens	T4	N0	M0	II
RJ063C	Renji.cohort	SFM-A	male	71	right hemico	T3	N1	M0	III
RJ08C	Renji.cohort	SFM-A	male	88	descendens	T3	N0	M0	II
RJ011C	Renji.cohort	SFM-B	male	77	rectum	T4	N0	M0	II
RJ013C	Renji.cohort	SFM-B	male	58	rectum	T4	N3	M0	III
RJ017C	Renji.cohort	SFM-B	male	65	rectum	T4	N0	M0	II
RJ026C	Renji.cohort	SFM-B	female	75	rectum	NA	NA	NA	NA
RJ02C	Renji.cohort	SFM-B	male	71	sigmoid	T2	N0	M0	I
RJ031C	Renji.cohort	SFM-B	male	79	sigmoid	T4	N1	M0	III
RJ033C	Renji.cohort	SFM-B	female	63	ascendens	T4	N1	M1	IV
RJ07C	Renji.cohort	SFM-B	male	59	sigmoid	T4	N0	M0	II
RJ015C	Renji.cohort	SFM-C	male	65	rectum	NA	NA	NA	NA
RJ016C	Renji.cohort	SFM-C	male	60	right hemico	NA	NA	NA	NA
RJ018C	Renji.cohort	SFM-C	female	75	ascendens	T4	N0	M1	IV
RJ019C	Renji.cohort	SFM-C	male	63	rectum	T2	N0	M0	I
RJ020C	Renji.cohort	SFM-C	male	82	ascendens	T3	N1	M0	III
RJ022C	Renji.cohort	SFM-C	male	62	ascendens	NA	NA	NA	NA
RJ023C	Renji.cohort	SFM-C	male	29	rectum	T4	N2	M1	IV
RJ025C	Renji.cohort	SFM-C	male	49	sigmoid	T4	N2	M0	III
RJ027C	Renji.cohort	SFM-C	female	69	sigmoid	T1	N0	M0	I
RJ032C	Renji.cohort	SFM-C	male	64	sigmoid	T4	N2	M0	III
RJ036C	Renji.cohort	SFM-C	male	31	sigmoid	T4	N0	M0	II
RJ040C	Renji.cohort	SFM-C	male	59	rectum	T2	N0	M0	I
RJ042C	Renji.cohort	SFM-C	male	87	ascendens	T4	N2	M0	III
RJ044C	Renji.cohort	SFM-C	female	56	right hemico	T3	N0	M0	II
RJ045C	Renji.cohort	SFM-C	male	62	sigmoid	NA	NA	NA	NA
RJ049C	Renji.cohort	SFM-C	male	53	right hemico	T4	N1	M1	IV
RJ051C	Renji.cohort	SFM-C	male	64	transverse	T4	N2	M0	III
RJ054C	Renji.cohort	SFM-C	male	54	sigmoid	T3	N0	M0	II
RJ056C	Renji.cohort	SFM-C	male	70	sigmoid	T4	N2	M1	IV
RJ058C	Renji.cohort	SFM-C	female	67	sigmoid	NA	NA	NA	NA
RJ059C	Renji.cohort	SFM-C	male	50	transverse	NA	NA	NA	NA
RJ060C	Renji.cohort	SFM-C	male	73	rectum	T4	N0	M0	II
RJ01C	Renji.cohort	SFM-E	male	59	sigmoid	T1	N1	M0	III
RJ021C	Renji.cohort	SFM-E	male	42	rectum	T4	N0	M0	II
RJ024C	Renji.cohort	SFM-E	male	46	ascendens	NA	NA	NA	NA
RJ029C	Renji.cohort	SFM-E	female	53	rectum	T4	N0	M0	II
RJ034C	Renji.cohort	SFM-E	female	65	sigmoid	T2	N0	M0	I
RJ035C	Renji.cohort	SFM-E	male	31	rectum	T4b	N2	M1	IV
RJ037C	Renji.cohort	SFM-E	male	66	rectum	T4	N2	M0	III
RJ038C	Renji.cohort	SFM-E	female	70	sigmoid	T3	N1	M0	III
RJ039C	Renji.cohort	SFM-E	male	73	rectum	T3	N0	M0	II
RJ03C	Renji.cohort	SFM-E	female	60	ileocecal ju	T4	N3	M0	III
RJ041C	Renji.cohort	SFM-E	male	69	sigmoid	T4	N2	M0	III
RJ046C	Renji.cohort	SFM-E	female	75	sigmoid	T3	N0	M0	II
RJ048C	Renji.cohort	SFM-E	male	68	sigmoid	T4	N1	M0	III
RJ053C	Renji.cohort	SFM-E	male	66	right hemico	T3	N0	M1a	IV
RJ05C	Renji.cohort	SFM-E	male	59	rectum	T4a	N2b	M0	III
RJ06C	Renji.cohort	SFM-E	male	56	left hemico	T4	N0	M0	II
RJ09C	Renji.cohort	SFM-E	male	82	right hemico	NA	NA	NA	NA

Supplementary Table 5. Details of gene signatures used for characteristizing SFM subtypes using NTP algorithm

signature_type	author name	paper name
EMT signature	Loboda et al	EMT is the dominant program in human colon cancer
colon crypt base vs top	Kosinski et al	Gene expression patterns of human colon tops and basal crypts and BMP antagonists as intestinal stem cell niche factors
stem-like signature	Merlos-Suarez et a	The Intestinal Stem Cell Signature Identifies Colorectal Cancer Stem Cells and Predicts Disease Relapse Cell Stem Cell
serreated CRC signature	Laiho et al	Serrated carcinomas form a subclass of colorectal cancer with distinctmolecular basis
FOLFOX response signatu	Tong et al	Identifying clinically relevant drug resistance genes indrug-induced resistant cancer cell lines and post-chemotherapytissues
FOLFIRI response signature	Graudens et al	Deciphering cellular states of innate tumor drug responses
EGFR inhitors signature	Schutte et al	Molecular dissection of colorectal cancerin pre-clinical models identifies biomarkerspredicting sensitivity to EGFR inhibitors

EMT_UP	EMT_DOWN_EPI	Crypt_bottom	Crypt_up	STEM	late_TA
ADAM23	ACPP	ACPL2	AAK1	4933406C1	1810022C2
ADAMTS1	AGR3	ACY1L2	ABCB1	5730471H1	2010106E1
AFF3	ALDH3B2	ADAMTS1	ABCG1	ARL4C	2210403K
AK5	ANK3	ADH1B	ABCG2	ASCL2	ACE
AKAP12	ANKRD22	ALDH1A1	ABHD5	ASRGL1	ACE2
ALPK2	ANXA9	ALDH1B1	ACE2	BCL2	ADH4
ANGPTL2	AP1M2	ALG8	ACHE	BEX1	AI317395
ANKRD1	AQP3	AMT	ACOX1	BEX4	AI481121
ANTXR1	ARHGAP8	ANAPC1	ADM	C13ORF15	AKP3
ANXA6	ARHGDIB	ANGPTL1	AIM1L	CBS	AKR1B7
AOX1	ATAD4	ANXA13	AKR1B10	CD44	ALDH1A1
AP1S2	ATP2C2	ARGBP2	ALDOB	CENPF	ANPEP
ARMCX1	B3GNT3	ARHGAP8	ALDOC	CHST11	APOA4
ATP8B2	BLNK	ARID5B	ALOX5	CYP11A1	APOC2
ATP8B3	BSPRY	ATBF1	ALPI	CYP2E1	APOC3
AXL	C11orf52	ATIC	AMACR	DCLK1	ASAH2
BDNF	C19orf21	ATOH8	AMFR	E330027M2	BST1
BICC1	C1orf106	ATP11A	AMID	EPHB3	C11ORF86
BNC2	C1orf116	AURKB	ANPEP	ESAM	C6ORF145
BVES	C1orf210	B4GALT2	APOBEC3B	FAM49A	CD36
C10orf38	C1orf34	BEX1	APXL2	FSTL1	CEACAM20
C10orf56	CCDC64B	BEX2	AQP7	FZD2	CHP2
C16orf45	CD24	BRIX	AREG	HEBP2	CIDEC
C1S	CDH1	BST1	ARHE	IGFBP4	CLEC2D
C9orf19	CDH3	BYSL	ARHGEF18	KIF26B	CLIC5
CAP2	CDS1	C18orf24	ARL4A	KLHL8	CML5
CCL2	CEACAM5	C4orf14	ARSA	LFNG	CNDP1
CDH11	CEACAM6	C5orf13	ASAHL	LGR5	CREB3L3
CDH2	CGN	CACNA2D2	ASS	LIMCH1	CUBN
CDH4	CKMT1B	CAP2	ATF3	MARVELD1	CYP2B6
CHN1	CLDN4	CCNA2	ATP1B3	MEX3A	CYP2C44
CLDN11	CLDN7	CCNB1	ATP6V0D1	MPP3	CYP2C68
CLIP3	CNKSR1	CCNB2	B2M	NA	CYP2D26
CMTM3	CNTNAP2	CDC2	BAPX1	NAV1	CYP2D6
COL12A1	CTAGE4	CDC20	BCAS1	PCDH8	CYP3A4
COL1A2	DAPP1	CDC25C	BCL10	PHGDH	CYP3A43
COL3A1	DENND2D	CDC6	BCLP	PRICKLE1	CYP3A7
COL4A1	DMKN	CDC7	BDKRB2	PSRC1	DNASE1
COL5A1	DSC2	CDCA1	BENE	PTHLH	DPEP1
COL5A2	DSP	CDCA3	BIRC3	PTPRO	EGLN3
COL6A1	EHF	CDCA5	BIRC4	PVT1	EMP1
CPA4	ELF3	CDCA7	BMP2	PXDN	ENPP3
CTGF	ELF5	CDK4	C18orf11	RASSF5	F3
CYBRD1	EPN3	CDK6	C21orf4	SCN2B	FAM3B
DAB2	EPPK1	CDKN3	C2orf31	SLC14A1	FMO5
DFNA5	ERBB3	CENPA	C4A	SLC1A2	G6PC
DIO2	ERP27	CENPE	C6orf115	SLCO3A1	GDA
DKK3	FA2H	CENPF	C6orf170	SOAT1	GDE1
DLC1	FAAH2	CFHL1	C6orf33	SORCS2	GDPD2
DOCK10	FAM110C	CGI-30	C9orf42	ST3GAL3	GGT1
DPYSL3	FAM83A	MECR	C9orf75	TACC1	HSD17B6
EDIL3	FAM84A	CHEK1	CA12	TEAD2	IFIT1
ELOVL2	FAM84B	CHRDL1	CA4	TGIF2	IFIT1L
EML1	FBP1	CIRH1A	CARD9	TUBB2B	IGSF9
EMP3	FGD2	CLU	CCL23	VAV3	IL18
EPB41L5	FGFBP1	CNN1	CCNG2		IL33
EPDR1	FUT1	CNTNAP2	CDA		KIAA1919
EVI2A	FUT3	COG4	CDH1		KRT20
F2R	FXYD3	COL12A1	CDH18		LCT
FAM101B	GALNT3	DDX20	CDKN1A		LEAP2
FAT4	GCNT3	DDX28	CEACAM1		LOC728588
FBN1	GJB6	DEPDC1	CGN		LRRC19
FGF2	GOLT1A	DEPDC6	CHMP1.5		MAF
FGF5	GPR110	DKC1	CHP		MAFB
FGFR1	GPR87	DKFZp762E1312	cig5		MALL
FHL1	GPX2	DKFZp762K222	CIT		MEP1B
FLRT2	GRAMD2	DLG7	CKB		MGAT4C
FSTL1	GRHL1	DMD	CLCN2		MOCOS
GFPT2	GRHL2	DNMT2	CLDN23		MOGAT2
GLIPR1	HOOK1	EBAF	CLDN3		NAT8
GLT25D2	HS3ST1	EBPL	CLDN4		NLRP9
GNB4	HS6ST2	EEF1A1	CLDN7		OAS3
GNG11	IL1RN	EEF1E1	CLIC5		OSGIN1
GPC6	ILDR1	EEF1G	CNGA1		OSTALPHA
GPR176	INPP4B	EPHB2	COBL		OSTBETA
GREM1	IRF6	EPHB3	COL17A1		OTOP3
HAS2	ITGB6	EPHB4	COPEB		PAPSS2
HEG1	KLK10	EXO1	CTDSP2		PCK1
HS3ST3A1	KLK5	EXOSC5	CTSE		PFKP
HTRA1	KLK6	FARSLB	CXADR		PLB1
IGFBP7	KLK8	FBN1	CYLN2		PLEKHG6
IL13RA2	KRT16	FEN1	CYP3A5		PRRX1
JAM3	KRT19	FHOD3	CYP3A7		PTK6
KIRREL	KRT5	FLJ10156	DAPK2		RAB30
LAMA4	KRTCAP3	FLJ10774	DDX58		RDH16
LEPREL1	LAMA3	FLJ13220	DEDD2		REG3A
LGALS1	LAMB3	FLJ14525	DEFB1		RHOC
LHFP	LAMC2	FLJ20294	DHRS9		SAA2
LIX1L	LCN2	FLJ22318	DIO3		SLC13A1
LOX	LCP1	FLJ23311	DIRC2		SLC13A2
MAP1B	LIPG	FLJ23441	DKFZp547A023		SLC15A1
MMP2	MAL2	FLJ40629	DKFZp564J157		SLC23A1
MRAS	MAP7	FLRT2	DKFZP5640123		SLC25A45
MSRB3	MAPK13	FMOD	DKFZp761D221		SLC26A3
NAP1L3	MARVELD2	FN1	DNAPTP6		SLC26A6
NAV3	MARVELD3	FOXM1	DOCK5		SLC2A2
NDN	MB	FRAS1	DPH2L1		SLC2A5
NEGR1	MBNL3	FYN	DPP3		SLC30A10
NEXN	MPP7	GAB1	DSC2		SLC34A2
NID1	MUC20	GCG	DSCR1		SLC47A1
NRG1	MYH14	GMNN	DST		SLC5A11
NUDT11	MYO5B	GNB2L1	DUSP5		SLC5A12
PAPPA	NPNT	GPC4	DYRK2		SLC5A4
PDE7B	OR2A4	GREM1	EDN2		SLC5A4B
PLAGL1	OVOL2	GREM2	EDN3		SLC6A19
PMP22	PAK6	GYG2	EFNA1		SLC6A20
PNMA2	PKP3	HH114	EGLN3		SLC9A3
POPDC3	PLA2G10	HPS3	ELF3		SLITRK6
POSTN	POU2F3	HSPC049	ELF4		SPNS2
PRKD1	PPL	HTLF	EMP1		SPRR2G
PRR16	PPP1R14C	HUNK	ENPP1		ST3GAL4
PTGIS	PROM2	IARS	EPHA2		SYNPO
PTRF	PRR15	IFRD2	EPS8		TEX12
PTX3	PRSS8	IL17RB	EPSTI1		TREH
RBM24	PTAFR	IMPDH2	F2RL1		TRIM15
RBMS3	PVRL4	IQGAP3	F3		UGT2A3
RBPMS2	RAB25	ITGB3BP	FAM46A		UGT2B17
RECK	RAPGEF5	KCNMB1	FBXO32		VWA1
RFTN1	RASEF	KCNN4	FGL1		
SERPINE1	RASGEF1B	KIAA0114	FHL2		
SIRPA	RBM35A	KIAA1155	FLJ10665		
SLC2A3	RBM35B	KIAA1324	FLJ10901		
SLC47A1	S100A14	KIAA1893	FLJ13456		
SPARC	S100A8	KIF14	FLJ14146		
SRGN	S100A9	KIF4A	FLJ14490		
SRPX	S100P	KIF5C	FLJ20152		
ST3GAL2	SCEL	LAMR1	FLJ20507		
SUSD5	SCNN1A	LBR	FLJ22761		
SYDE1	SERPINB5	LDHB	FLJ23129		
TBXA2R	SH2D3A	LEFTB	FLJ25084		
TCF4	SH3YL1	LOC151878	FLJ31842		
TGFB2	SLC6A14	LOC219347	FLJ33915		
TMEM158	SLPI	LOC283345	FLJ35954		
TMEM47	SORBS2	LOC283596	FLJ37131		
TMSL8	SPINK5	LOC285705	FLJ38564		
TNFRSF19	SPINT1	LOC339316	FLJ40432		
TRPA1	SPINT2	LOC388974	FLJ43855		
TTC28	SPRR1A	LOC91942	FMO5		
TTLL7	SPRR1B	LRIG1	FOSL2		
TUB	SPRR3	LSM8	G1P2		
TUBA1A	ST14	MAD2L1	GBAP		
UCHL1	STEAP4	MAGEF1	GBP3		
VIM	STX19	MAL	GCNT3		
WIPF1	SYK	MCM3	GDA		
WNT5B	SYT7	MCM6	GDPD2		
ZEB1	TMC4	MELK	STBD1		
ZEB2	TMC5	MGC13170	GGA2		
ZFPM2	TMEM125	MGC14560	GGT2		
ZNF788	TMEM30B	MGC24665	GGTL4		
	TMEM45B	MGC26690	GK		
	TMPRSS11E	MGC3036	GNA11		
	TMPRSS13	MGC57827	GNG12		
	TMPRSS4	MKI67	GPA33		
	TOX3	MLF1IP	GPR115		
	TRIM29	MLPH	GSDML		
	TSPAN1	MRPL1	GTF2H5		
	TTC22	MRPL24	GUCA2A		
	TTC9	MRPL3	GZMA		
	VTCN1	MRPS2	HDAC9		
	WDR72	MTB	HIG1		
	WFDC2	MTHFD1	HIST1H1C		
		MTL5	HIST1H2AC		
		MTR	HIST1H2BC		
		MUC5B	HIST1H2BD		
		MYBL2	HIST1H3H		
		MYC	HIST1H4H		
		MYL9	HK2		
		MYO9A	HMOX1		
		MYST3	HN1		
		NAV3	HNF4A		
		NEK2	HPGD		
		NEXN	HS6ST1		
		NGFRAP1	HSD17B2		
		NHS	IFNGR2		
		NME1	IGSF3		
		NOB1P	IGSF9		
		NOL5A	IL10RB		
		NOLA2	IL28RA		
		NR2C2	IL2RG		
		NS	ISG20		
		NUP35	ISGF3G		
		NUSAP1	IVNS1ABP		
		OGDH	JUNB		
		OGN	JUND		
		OLFM4	KATNA1		
		OXCT1	KCNK1		
		PAFAH1B3	KIAA0063		
		PAICS	KIAA0376		
		PCDH7	KIAA0830		
		PCOLN3	KIAA0931		
		PEMT	KIAA1145		
		Pfs2	KIAA1211		
		PGAP1	KIAA1363		
		PGM5	KIAA1539		
		PKMYT1	KIAA1613		
		PLCB4	KMO		
		PLK1	KRT19		
		POLE2	KRT8		
		POLR1C	LAMA1		
		POLS	LAMB3		
		PRELP	LAMC2		
		Prostein	LARGE		
		PRRX1	LBA1		
		PTPN21	LHFPL2		
		PTPRO	LIPH		
		PTTG1	LITAF		
		PYCR1	LMO7		
		RAD51	LOC129607		
		RAD54B	LOC134548		
		RAMP	LOC196264		
		RANBP5	LOC222171		
		RASGEF1C	LOC253981		
		RCN1	LOC255189		
		RERG	LOC255512		
		RFC3	LOC339321		
		RFC4	LOC51066		
		RGMA	LOC90133		
		RODH	LOC90355		
		RPL10A	LPIN2		
		RPL12	LRP4		
		RPL21	LRRC19		
		RPL31	LTK		
		RPL35	MAD		
		RPL5	MAP1B		
		RPL7A	MAP1LC3B		
		RPS14	MAP2K5		
		RPS19	MAPK3		
		RPS27	MAPK6		
		RPS3	MEN1		
		RPS5	MEP1A		
		RPS6	MFI2		
		RPS8	MGAT4A		
		RPS9	MGC11324		
		RRM2	MGC13057		
		RUVBL1	MGC14376		
		SCML2	MGC24039		
		SEC8L1	MGC50853		
		SERPINA3	MGC72075		
		SFRP1	MGLL		
		SFXN4	MICAL2		
		SH3BP4	MKRN1		
		SIAT7B	MME		
		SIM2	MMP14		
		SLC12A2	MSP		
		SLC38A5	MXI1		
		SLC39A8	MYLK		
		SMARCA4	NDFIP2		
		SMOC2	NEDD9		
		SMTN	NGFB		
		SPINK1	NK4		
		SPRY1	NPY		
		SSX2IP	NT5C3		
		STK33	OAS1		
		SULF1	OCLN		
		SYNCRIP	OKL38		
		SYNPO2	OR7E47P		
		SYTL2	OSTbeta		
		TAGLN	OSTM1		
		TARBP1	P2RY2		
		TCEAL4	P4HA2		
		TCF19	PADI2		
		TFCP2	PAG		
		TIGA1	PAPPA		
		TIMP3	PARD6B		
		TK1	PAX8		
		TLE2	PDE1A		
		TOP2A	PDE9A		
		TOPK	PDGFA		
		TP53	PDXP		
		TP53I11	PDZK2		
		TPX2	PEX26		
		TRAP1	PHC3		
		TRIP6	PI3		
		TTK	PKIB		
		TUFT1	PLA2G7		
		TYMS	PLAC8		
		UBA52	PLAUR		
		UBE2C	PLCD1		
		UGT1A6	PLEKHA7		
		VPS41	PLEKHF2		
		WBSCR14	PLOD2		
		WFDC2	PLXNA2		
		ZCWCC2	POLD4		
		ZNF10	PPGB		
		ZNF185	PPIE		
		ZNF367	PPM1A		
		ZNF84	PR1		
		ZWINT	PRAP1		
			PRDX6		
			PRKCD		
			PRSS12		
			PRSS23		
			PRSS3		
			PSG1		
			PSG11		
			PSG4		
			PTPN18		
			PTPRD		
			PTPRH		
			PTPRR		
			RABEP1		
			RAI3		
			RARRES3		
			RECQL5		
			REV1L		
			RFK		
			RGS13		
			RGS19IP1		
			RHOC		
			RHOF		
			RHOG		
			RHPN2		
			RIOK3		
			RNF103		
			RNF152		
			ROPN1L		
			SARG		
			SAT		
			SCNN1A		
			SDCBP2		
			CCDC68		
			SEC23A		
			SEMA6D		
			SERPINA12		
			SFN		
			SFTPB		
			SGCD		
			SGK		
			SGK2		
			SGPP2		
			SHD1		
			SIRT6		
			SLC17A4		
			SLC1A1		
			SLC20A2		
			SLC22A5		
			SLC25A23		
			SLC25A25		
			SLC26A2		
			SLC2A1		
			SLC30A10		
			SLC36A1		
			SLC3A1		
			SLC40A1		
			SLC6A19		
			SLC6A8		
			SLC9A1		
			SLC9A2		
			SLC9A3R1		
			SMAD3		
			SMPD1		
			SMPDL3A		
			SOX17		
			SP1		
			SPINT1		
			SPINT2		
			SPRED2		
			SRI		
			SRPRB		
			STX3A		
			SYTL5		
			TAX1BP3		
			TBC1D1		
			TCF7L2		
			TCN2		
			TDE2L		
			TESK2		
			TEX11		
			TFF1		
			THBS1		
			TINAG		
			TJP3		
			TLR3		
			TM4SF8		
			TMEM24		
			TMPIT		
			TMPRSS2		
			TNFAIP8		
			TNFRSF21		
			TNFSF10		
			TNKS2		
			TP53INP1		
			TP53INP2		
			TPK1		
			TRIF		
			TRIM31		
			TRIM5		
			TRPV3		
			TST		
			UACA		
			UGCG		
			UGP2		
			UPP1		
			USP2		
			VAMP8		
			VASP		
			VDR		
			VEGF		
			VIL2		
			VILL		
			VIPR1		
			XRN1		
			YPEL1		
			ZC3HDC1		
			ZNF219		

serrated_	non-serra	FOLFIRI_	FOLFIRI_	FOLFOX_	FOLFOX_
ANXA2	ERAL1	LOC25513C	FLJ41589	APOC1	NAT1
PLS1	FBXO46	MGC4083	MGC4189	BST2	ABP1
HIF1A	SH2B	BC008967	LGALS1	C1QA	ACTG2
ANXA2	SMCX	ARMCX1	MGC4504	CD38	ADORA2B
PAM	F8A1	BA108L7.2	GTF2H1	CDA	ANG
ATP1B1	HNRPA1	FLJ45342	ABCC1	AKR1C4	APOBEC1
PLEKHB2	PABPN1	ABL1	ALS2CR2	CHIT1	ASCL2
SDCBP	ATP7B	ACTR1A	ARL4A	ABCC2	KLF5
ATP2B1	NFATC2IP	AGRN	ASF1B	COL9A3	BUB1B
NOLA3	KATNB1	AHR	ASNS	COL17A1	CCNB1
MFAP1	MTA1	ANXA5	AURKB	DFNA5	CDC6
ARPP-19	NEK3	ARL7	BIRC5	DUSP5	CDH17
ADAM10	CRSP2	BGN	C10orf3	EGR2	CDKN3
		BRD9	C20orf55	FCGR2B	CHGA
		C5orf4	CDC20	HSPA6	CLCA1
		COL11A1	CENPJ	HSPB1	CPE
		COL2A1	CHEK1	ID1	CLDN3
		COL4A2	CHEK2	IL6	CLDN7
		COL6A1	CKB	LAMA5	DDC
		COL6A2	CKS1B	CXCL9	COCH
		CSPG2	CRIP1	MMP9	SLC26A2
		CSRP1	DDX11	MTTP	EPHB3
		DAB2	DIRAS2	CLDN11	F2RL1
		DDR2	DKFZp762E	SERPINE1	FOXF2
		DUSP4	DPYSL2	PLTP	FMO5
		ECM1	FASTK	PRNP	FRZB
		EFEMP2	FGFBP1	CCL2	NR5A2
		EGR1	FLJ10407	CCL5	FUT2
		EMILIN1	GLO1	CCL18	FUT3
		FER1L3	GOLGA4	CXCL12	FUT4
		FSTL1	HIS1	SLC2A3	GCG
		GLG1	HMGB2	SLC22A3	GMDS
		GTF2H1	HOXA1	TNNC1	CXCL3
		GUCY1A3	KIAA0040	TYROBP	GUCA2A
		HIST2H2BE	KIF13A	UCHL1	HLA-DPB1
		HRMT1L1	KIF2C	CYP4F2	HMGCS2
		IGFBP6	KIF4A	SLIT2	FOXA3
		IGFBP7	KLF13	GFPT2	HNF4G
		KIAA1627	KLHDC3	MSLN	HOXA11
		LAMB1	LDHB	GPNMB	HOXA13
		LMCD1	LMNB2	PROCR	HSPA2
		LMO2	LOC90355	TNFSF13B	IL7
		LOC92689	LSM4	C10orf116	IMPA2
		LOXL2	MAD2L1	PTP4A3	KIF11
		LTBP4	MCM6	VSIG4	LGALS2
		LUM	MKKS	TRIM29	LGALS4
		MAPK12	MPDU1	FAM169A	VWA5A
		MAPK9	MSH5	KCNMB4	MAOA
		MEIS2	NET1	STMN3	MCM2
		MGC5395	NUP88	TNFRSF12A	SCGB2A1
		MMP11	PAICS	PLA1A	NR3C2
		MMP3	PFN2	CECR1	MMP3
		MMP7	PHGDH	EPDR1	MT1M
		MYL9	PRAME	CYP4F11	MUC2
		NAV1	PRC1	FKBP10	MUC4
		NDRG4	PRKCD	COLEC12	MYB
		NHP2L1	PRKDC	PLA2G12B	MYO1A
		PCDHGC3	RAB3A	KLHL13	NEK2
		PCOLCE	RAB6IP1	UAP1L1	TNFRSF11B
		PDHB	RAD51	PRAP1	ORC1
		PEA15	RANBP1	MUC17	PCK1
		PHLDB1	RECQL4	IGFL2	PCSK1
		PIAS3	RPL3	CCDC80	ATP8B1
		PLAT	RRM2		PIGR
		PPP3CC	SESN2		PKP2
		PTGS1	SFMBT1		PLA2G2A
		PTTG1IP	SLC35F5		PLAGL1
		RAB31	SLC43A1		PLS1
		RERG	SPAG5		PPARG
		RRAGD	ST14		PRIM1
		SELE	STK6		PTPRD
		SLC43A3	TAP2		RARRES1
		SPARC	TGDS		RFC4
		SPHK1	TGIF2		RFC5
		SQSTM1	TK1		RNASE4
		SULF1	TP53		STIL
		TAGLN	TPX2		SLC9A2
		TEAD2	TRIP13		HLTF
		TERF2IP	TTK		SPAG1
		TGFB1I1	TYMS		SPINK1
		TGFBI	UNG		SRI
		THBD	VILL		TCEA3
		THBS3	WDR12		TFF3
		THY1	YARS		TLR3
		TIMP2			TOP2A
		TNC			TST
		ZNF331			TTK
					TYMS
					UGT8
					UNG
					USP1
					VRK1
					XK
					IL1R2
					CADPS
					B4GALT4
					TNFRSF11A
					FCGBP
					SEMA5A
					PRC1
					CCNE2
					EBAG9
					GCNT3
					KLF4
					APOBEC3B
					DLGAP5
					KIF14
					FGFBP1
					KCNE3
					GNE
					TSPAN1
					KIF20A
					PDZK1IP1
					ALYREF
					GPA33
					NDC80
					WFDC2
					SPON1
					HOXB13
					AGR2
					SLC35A1
					PAIP1
					SPAG5
					CAPN9
					MTMR11
					KIF2C
					SPINK5
					ZWINT
					GPD1L
					TRIM2
					SLC35A3
					VSIG2
					HIGD1A
					LRIG1
					APPL1
					EHF
					GPR160
					NOX1
					TSPAN13
					TINAG
					SULT1B1
					SPINK4
					STXBP6
					PSAT1
					VILL
					SLC35B3
					RAPGEFL1
					NUSAP1
					DTL
					TDP2
					ACSL5
					GALNT7
					KRT20
					RNF186
					L1TD1
					FAM55D
					BSPRY
					SYTL2
					ESRP1
					SIDT1
					MS4A12
					PPP1R14D
					RHBDL2
					EPN3
					FAIM
					GOLPH3L
					C4orf19
					HES6
					ITLN1
					DEPDC1
					FAR2
					ZNF83
					ST6GALNAC1
					MYO5C
					CCBL2
					LXN
					BBX
					KIF15
					RAB25
					CD177
					VANGL2
					SPC25
					PLEKHH1
					KIAA1211
					KIAA1324
					NCEH1
					ZSWIM5
					CHP2
					C19orf33
					CENPK
					C5orf28
					SUDS3
					LRRC19
					MLPH
					PRR15L
					MLF1IP
					NSUN7
					E2F8
					LRRC31
					CLMN
					ARL14
					ASRGL1
					CWH43
					SLC44A4
					APOLD1
					ITM2C
					MS4A8B
					SH3BGRL2
					MND1
					PPP1R1B
					TMEM246
					C15orf48
					LNX1
					HPDL
					PAQR8
					ZNF468
					ZNF502
					MRAP2
					HENMT1
					FBXO32
					RPL39L
					FAM55A
					GGT6
					CCDC58
					FAM3D
					RNF183
					SPIN4
					PPP1R36
					CDC42EP5
					SAMD13
					MANEAL
					AIFM3
					SGOL2
					SLC16A14
					LRRC34
					CMC1
					AGR3
					SPTSSA
					B3GNT6
					MUC20
					SLC16A9
					FAM171A1
					C6orf130
					PRR15
					HEPACAM2
					PCSK9
					POC1B
					RGMB
					FAM221A
					C9orf152
					ZG16
					CD24

avastin_S	avastin_F	cetuxima	cetuxima	afatinib_S	afatinib_I
LUC7L	ALOX5	TSPAN6	FYN	TAC1	PRSS22
HSF2	CPS1	CEACAM7	ALOX5	NLRP2	FYN
TOMM34	NLRP2	KIF26A	VSIG2	KIF26A	RAB27B
PHKA2	TYMP	CYP2W1	GRAMD1B	GAL	TNC
RCN1	ZIC2	DNMT3B	RAB27B	CXCL2	ZIC2
MCUR1	TMCC3	CDIP1	TNC	CDIP1	WNK1
LETMD1	PTPRU	SORBS1	ZIC2	COCH	SYT1
SPAG4	GPC1	TIMP3	GNA15	PCSK1N	MAOB
MYH7B	BCAS1	PLTP	DGKA	TBX18	GNB5
C1QTNF3	SBNO2	ABHD12	TRIB2	FN1	DHRS9
RRAGB	CLDN18	RAE1	FSCN1	CNTFR	ZNF532
PEBP1	PFKP	NELFCD	HSD17B2	ZNF337	FSCN1
CECR1	GNB5	PSMA7	PXN	WASF3	ERC1
DDT	FSTL3	POFUT1	LYZ	E2F5	LYZ
GZMB	DHRS9	PIGU	GLG1	GSTM1	NRCAM
ISM2	ANO8	ACOT8	FCGBP	MYC	DPYSL2
TRIB3	CIC	APMAP	CD200	CAMKV	ABLIM1
AHCY	SLC4A4	SCML2	NRP1	SLC39A2	NRP1
SMS	FCGBP	NOD1	APOL1	HDGFRP3	NTSR1
FGD1	HSD3B7	RARRES2	NTSR1	PPP1R14A	RBBP8
PIN4	STS	SLC6A4	SMARCA1	DMRT2	SRPX
PBDC1	CA2	KAT2A	C16orf62	BRSK2	SMARCA1
BEX4	DMPK	ACOT13	CA2	NUPR1	CALB1
AQP8	TNNT1	FN1	CALB1	BNIP3	DKK4
CALB1	TGFB1	TGIF2	CAV2	PRR15	SERPINE1
FZD10	ERF	COPS5	BLVRA	RPRML	DNAJC12
TMEM14C	SIPA1L3	FABP3	UNC5B	TOP1MT	ST3GAL4
TNNC1	NPTX2	MOCS3	ANXA10	KLRG2	IL23A
OLFML3	ADTRP	DDX27	SELPLG	ANKRD13B	CDK2AP1
NID1	DUSP1	BCAS4	ADTRP	ZDHHC11B	DBN1
MTIF3	CHST3	EREG	VNN1		GNAI2
GTF3A	PI3	GZF1	PDE4D		SLC4A3
EBPL	SEMG1	SNRPB2	HYAL1		GADD45A
PFDN5	AHNAK	PLAGL2	MLPH		CRYZ
ATP5E	ABCC4	SNRPN	KYNU		F3
WRNIP1	SCAF1	TAF4	EPHA4		NRP2
SNRPC	PRR12	RAMP2	F3		FKBP1B
RPP40	MLLT1	NXT1	RARRES1		DUSP4
GPCPD1	ULBP2	CTNNBL1	KLF12		CHST3
SNRPB2	BARX1	PPP1R3D	FKBP1B		SEMG1
PRDX5	KDM6B	WASF3	IFIT3		TRERF1
CTAG2	CA1	E2F5	IFIT2		PIWIL1
TNFRSF19	REG4	ECHDC3	DUSP4		HOXD1
ERMARD	RSAD2	SLC19A3	BHLHE41		GAD1
CKMT2	CMPK2	TBRG4	RAB38		HOXD9
MTL5	ITM2C	MYC	SEMG1		HOXD10
LRP4	GALNT5	FHDC1	TRERF1		HOXD11
RBMX2	DDX60	TTPA	CDKN1A		HOXD13
TIMM10	SLC28A2	NXPE4	NRN1		RAI2
SLC19A3	IFI44L	SLC5A6	HOXD1		RAMP1
PHF11	IFI44	TRIM54	HOXD9		SERPINF1
TMEM14B	MYOF	SERPINA1C	HOXD13		NMUR2
BPHL	OSBPL1A	RASL10B	BST2		TTC9
HMGA1	CTD-3222D	SYBU	ULBP2		DSG3
SLC5A6	EVI5L	SPATA25	ANO1		TCN1
LNX2	CDC42BPA	WIF1	RAMP1		CKAP4
RASL10B	SETD7	SUSD3	REG4		TUBB2B
RAB40B	TRIM7	GDPD5	TCN1		BCAR3
NBEAL1	TMEM140	SLC1A7	ANXA1		CALHM2
CXCL14	TMCO3	CAMKV	OASL		LEF1
TBC1D7	SEC24D	MED30	MDFIC		GABARAPL1
AIG1	NR3C2	HEATR2	KRT7		CLSTN3
ZNF711	AP1S3	SLC39A2	ITM2C		AMIGO2
C12orf45	PID1	HDGFRP3	IL1RN		PHLDA1
TSEN2	KCTD15	ENGASE	TUBB2A		ABHD2
GTF3C6	CD109	PPP1R14A	IFI44L		IL17RD
WIF1	STEAP2	CCDC88B	LEF1		PLK2
C14orf2	ABHD3	MPLKIP	PHLDA1		DCDC2
GRHL3	AHCYL2	SERPINA6	RHOF		PPP1R18
SLC13A3	FAM131B	NANP	WARS		SYTL5
ALDH4A1	NACC1	CHCHD7	MAP3K6		FAM83A
VMA21	DMKN	CEL	CTDSPL		GSN
AQP5	CXCL16	TP53RK	NFKBIZ		CD109
SLC1A7	SSTR5	PDE3A	PLK2		AQP5
AXDND1	MEGF6	FADS6	ZNF300		FGF19
GPR155	SGPP2	DMRT2	IGFBP3		SERPINI1
RETNLB	C1orf106	STAT5B	SYTL5		FAM198B
SERPINI1	PRSS12	PAAF1	DOCK5		RHOBTB3
FUNDC2	TLR3	TMEM70	FAM83A		GPX8
C2	HDGFRP3	NUPR1	CDKN2B		COL1A2
TPP1	SCG5	BNIP3	LCN2		SBSPON
STX3	ABCA3	PRR15	C12orf23		ALDH1A1
PDIA3	TRIM56	JUN	TNIK		MMP10
NOD2	NLGN2	FJX1	CD109		LY6D
ATP5L	CHD3	RPRML	CREB3L1		NXN
PPP1R14A	SEZ6L2	TMEM252	AHCYL2		KLK6
SMIM4	ASPHD1	C8orf33	TFF2		KLK11
CST2	ZG16	URAD	CCL5		IGFBP6
CST1	SAMD9L	MAP7D2	ZNF385A		TM4SF4
C20orf196	FAM20C	TOP1MT	AQP5		HOXD4
ALG14	PLEC	PTP4A3	SGCB		ABLIM3
TRMT112	CTXN1	MAGEA11	SPATA18		HOXB2
MRP63	TMEM64	FOXD2	ALPPL2		HOXD8
AHSA2	RELL1	SMTNL2	ANTXR2		WSB2
GOLT1A	B3GALT5	KLRG2	IFI16		ZBED2
UBE2C	IFIT1	ZNF34	PTPN13		ARSJ
RMI2	CCDC42B	FITM2	CDCP1		HEPHL1
ETV4	PLA2G2A	ENTPD6	RHOBTB3		SOX2
SOX12	HES4	CES1	GPX8		CHST15
ZCCHC3	HBA2	PCMTD2	SBSPON		CRIP2
ATP5EP2	ENTPD8	SNURF	KLK6		LCK
C11orf71	CTSE	YAE1D1	IGFBP6		CBX6
CHST13	STMN3	AMACR	HR		SLITRK6
PSMG4	B3GNT6		KLK7		KRT5
XKRX	MUC2		TM4SF4		THBS2
SFXN4	SAMD9		HOXD4		ARL4C
CHEK2	CRYBG3		KCNS3		GJB5
TMEM186	LEFTY1		SDR16C5		SERPINA3
KLHDC8B	AGAP2-AS1		ABLIM3		SPTSSB
POLR1D	CEA		CREG2		PLXNB3
SPIN2B	AC011530.4		HOXD8		CSAG1
S100A3	AC006486.9		PLA2G16		C6orf15
COMMD6	CTD-2207023.3		WSB2		KRT6A
ADAT2			ARSJ		KLRC2
APOD			SOX2		MAGEA12
SULT1A1			CHST15		MAGEA3
MME			LCK		ETV5
LAMA2			ARHGEF37		TRNP1
TOMM7			SLITRK6		
MAGEA6			TMEM173		
CES1			ZBTB7C		
CSAG1			IFIT1		
RNF5			KRT5		
CSNK2B			THBS2		
TMSB4X			AGRN		
MAGEA12			HBA2		
ARL2			GJB5		
TMX2			SERPINA3		
LYRM4			CTSE		
ALG3			CD55		
MAGEA3			SPTSSB		
L1TD1			FAM177B		
AMACR			FAM114A1		
EIF6			PLXNB3		
NACA2			MUC2		
RP11-691N7.6			SERPINB5		
			ARHGEF28		
			TRNP1		

AZD8931	AZD8931	gefitinib_	gefitinib_	vandetan	vandetanib_R
TAC1	PLXND1	FAS	COL9A3	CLCA1	SEMA3F
GGCT	FYN	RRM2B	RUNDC3A	CPS1	HSPB6
SLC13A2	SLC18A1	SI	GNAI2	FAS	CORO1A
TKTL1	TNC	JPH1	ERRFI1	SI	GNAI2
MRPS24	ZIC2	SPAG1	RGS4	CALB1	C3orf14
KIF26A	RIMBP2	BAG2	INS-IGF2	SPAG1	RGS4
GAL	MAOB	CCND2	NES	BAG2	TGM3
SLC4A4	TRIB2	SPINK4	CHRNA1	BTNL8	INS-IGF2
KIAA0020	ZNF532	PLAU	ART3	CCND2	COL5A1
COCH	FSCN1	SERPINB6	IGF2BP1	SPINK4	CHI3L1
GLA	LAT2	CD70	SBSPON	PLAU	LCP1
CBLN1	LYZ	TRIM22	TMPRSS5	BST2	RBP4
CCL20	FCGBP	EFR3A	IGF2	HRASLS2	CHRNA1
FN1	DPYSL2	GSTM1	BASP1	GSTM1	CXCL14
ACTL8	NRP1	CHST5	PGBD5	SLC28A2	PRAC1
NIP7	NTSR1	SLC28A2	FAM3B	PHLDB2	IGF2
WASF3	RBBP8	PTPN7	PCDHB16	PLEKHS1	PCCA
E2F5	CALB1	TDO2	DSCR8	ESAM	BASP1
GSTM1	NCALD	KCNJ3		TMEM200A	FAM3B
ECHDC3	PON3	TMEM200A		TPST1	KCNH8
LRP4	MEGF9	AQP3		KCTD12	C1orf110
SLC19A3	UNC5B	PDZD8		MUC1	PTCH1
TBRG4	DNAJC12	RAB3B		C2CD4A	PCDHB16
NOB1	COL1A1	KCTD12		SGMS1	TUBB3
RASL10B	PCDHB2	PRKX		MICB	GJC2
HSPD1	GNAI2	C2CD4A		TEX40	RGL3
BOD1	HYAL1	SGMS1			LAT
TYSND1	SLC4A3	MALRD1			
CELF3	EPHA4	IFI30			
SLC39A2	GADD45A	RP11-77K12.7			
HDGFRP3	RIMS3				
PPP1R14A	F3				
MPLKIP	FKBP1B				
SERPINA6	DUSP4				
PKIA	RNF11				
PDE3A	KCNJ2				
DMRT2	TRERF1				
RMI2	PIWIL1				
NUPR1	CTAG2				
BNIP3	GNAI1				
PRR15	HOXD1				
RPRML	GAD1				
RNF135	HOXD9				
XKRX	HOXD13				
MAP7D2	AKAP12				
TOP1MT	ANO1				
NKRF	RAMP1				
CLN3	REG4				
KLRG2	DSG3				
PCDH20	CALHM2				
SNURF	PDE5A				
	MTTP				
	PHLDA1				
	CELF6				
	ABHD2				
	PTPRF				
	SOX13				
	PLK2				
	SYTL5				
	FAM83A				
	CDKN2B				
	TFF2				
	GATS				
	AQP5				
	SERPINI1				
	DNALI1				
	COL1A2				
	TNFRSF11B				
	SBSPON				
	MYRFL				
	ANPEP				
	MS4A8				
	LY6D				
	KLK6				
	KLK11				
	TM4SF4				
	HOXD4				
	SIX2				
	GPR37				
	SDR16C5				
	ABLIM3				
	HOXB2				
	AKIRIN1				
	HOXD8				
	WSB2				
	TRIM72				
	CA8				
	PRF1				
	ARSJ				
	SOX2				
	HOXB4				
	CRIP2				
	CBX6				
	SRPK3				
	SLITRK6				
	ZBTB7C				
	MAPK11				
	KRT5				
	THBS2				
	BCAM				
	HEPACAM2				
	HBA2				
	SPTSSB				
	CACNA1H				
	MBP				
	AKR1C4				
	F5				
	PLXNB3				
	DUSP27				
	TOX				
	C6orf15				
	PRSS1				
	KRT6A				
	KLRC2				
	ETV5				
	TRNP1				

Supplementary Table 6. molecular, clinicopathological features and drug response across SFM subtypes based on NTP algorithm

sample_id	SFM subtypes	STEM	EMT	crypt_top	serratedCRC	FOLFIRI_response	Vandetanib_respor	Gefitinib_response	AZD8931_response	afatinib_response	cetuximab_respons	Avastin_response	FOLFOX_response
GSM971957	SFM-C	undetermined	emt	base	nsCRC	with_response	undetermined	undetermined	with_response	with_response	with_response	with_response	undetermined
GSM971979	SFM-C	stem	emt	top	sCRC	with_response	undetermined	undetermined	with_no_response	with_no_response	with_no_response	with_no_response	with_no_response
GSM971980	SFM-C	late.TA	epi	top	sCRC	undetermined	with_response	undetermined	with_no_response	with_no_response	with_no_response	with_no_response	with_no_response
GSM971983	SFM-C	stem	emt	base	sCRC	with_response	with_response	undetermined	with_no_response	with_no_response	with_no_response	with_no_response	undetermined
GSM971988	SFM-C	late.TA	epi	top	undetermined	undetermined	with_response	undetermined	with_no_response	with_no_response	with_no_response	with_no_response	undetermined
GSM971997	SFM-C	undetermined	emt	base	undetermined	undetermined	undetermined	with_response	with_no_response	with_no_response	with_no_response	with_no_response	with_response
GSM972002	SFM-C	stem	epi	top	nsCRC	with_no_response	undetermined	undetermined	with_no_response	with_no_response	with_no_response	undetermined	with_no_response
GSM972015	SFM-C	undetermined	epi	top	sCRC	with_response	with_response	undetermined	with_no_response	with_no_response	with_no_response	with_no_response	with_no_response
GSM972016	SFM-C	undetermined	emt	base	sCRC	with_no_response	with_response	undetermined	undetermined	with_response	with_no_response	with_no_response	with_no_response
GSM972056	SFM-C	late.TA	epi	top	undetermined	with_no_response	undetermined	undetermined	with_no_response	with_no_response	with_no_response	with_no_response	with_response
GSM972058	SFM-C	stem	undetermined	base	sCRC	undetermined	with_response	with_response	with_no_response	with_no_response	with_no_response	with_no_response	undetermined
GSM972076	SFM-C	stem	emt	base	sCRC	with_response	undetermined	with_response	with_no_response	with_no_response	with_no_response	with_no_response	with_no_response
GSM972080	SFM-C	undetermined	undetermined	undetermined	sCRC	with_no_response	with_response	with_response	with_no_response	undetermined	with_no_response	with_no_response	with_no_response
GSM972114	SFM-C	late.TA	epi	undetermined	sCRC	with_no_response	undetermined	undetermined	with_no_response	with_no_response	with_no_response	with_no_response	with_no_response
GSM972129	SFM-C	undetermined	epi	undetermined	sCRC	with_no_response	with_response	with_response	with_response	with_response	with_no_response	with_no_response	with_no_response
GSM972168	SFM-C	undetermined	epi	top	sCRC	with_no_response	with_response	with_response	with_no_response	with_no_response	with_no_response	with_no_response	undetermined
GSM972196	SFM-C	undetermined	epi	top	undetermined	with_no_response	undetermined	undetermined	with_no_response	with_no_response	with_no_response	undetermined	with_response
GSM972214	SFM-C	undetermined	epi	top	sCRC	with_no_response	with_response	with_response	with_no_response	with_no_response	with_no_response	with_no_response	with_no_response
GSM972221	SFM-C	stem	epi	base	undetermined	with_no_response	with_response	with_response	with_no_response	with_no_response	with_no_response	with_no_response	with_no_response
GSM972222	SFM-C	undetermined	epi	top	sCRC	with_no_response	undetermined	with_response	with_response	with_response	with_no_response	with_no_response	with_no_response
GSM972223	SFM-C	late.TA	emt	top	sCRC	undetermined	with_response	with_response	with_no_response	with_no_response	with_no_response	with_no_response	with_no_response
GSM972226	SFM-C	late.TA	epi	top	sCRC	with_no_response	with_response	with_response	with_no_response	with_no_response	with_no_response	with_no_response	with_no_response
GSM972229	SFM-C	stem	undetermined	base	undetermined	undetermined	undetermined	with_response	with_no_response	with_no_response	with_no_response	undetermined	undetermined
GSM972261	SFM-C	undetermined	epi	top	sCRC	undetermined	undetermined	with_response	with_no_response	with_no_response	with_no_response	with_no_response	undetermined
GSM972264	SFM-C	stem	epi	base	undetermined	with_no_response	with_response	with_response	with_response	with_response	with_response	undetermined	with_no_response
GSM972265	SFM-C	undetermined	undetermined	undetermined	nsCRC	with_response	undetermined	with_response	with_no_response	undetermined	with_no_response	with_no_response	with_no_response
GSM972270	SFM-C	late.TA	epi	undetermined	undetermined	with_no_response	undetermined	with_response	with_no_response	undetermined	with_no_response	with_no_response	with_no_response
GSM972276	SFM-C	late.TA	epi	top	sCRC	with_no_response	with_response	with_response	with_no_response	undetermined	with_no_response	with_no_response	undetermined
GSM972277	SFM-C	undetermined	epi	top	sCRC	with_no_response	undetermined	undetermined	with_no_response	with_no_response	with_no_response	with_no_response	with_no_response
GSM972278	SFM-C	stem	epi	undetermined	undetermined	with_response	undetermined	undetermined	with_no_response	with_no_response	with_no_response	undetermined	with_response
GSM972280	SFM-C	stem	emt	top	undetermined	with_no_response	undetermined	with_no_response	with_no_response	with_no_response	with_no_response	with_no_response	with_response
GSM972282	SFM-C	stem	epi	base	nsCRC	with_no_response	undetermined	with_no_response	with_response	with_response	with_response	with_response	with_no_response
GSM972283	SFM-C	undetermined	epi	top	sCRC	with_response	with_response	undetermined	with_no_response	with_no_response	with_no_response	with_no_response	with_response
GSM972285	SFM-C	undetermined	epi	top	sCRC	undetermined	with_response	with_response	undetermined	undetermined	with_no_response	with_no_response	with_no_response
GSM972286	SFM-C	undetermined	emt	base	sCRC	with_no_response	with_response	with_response	with_no_response	undetermined	with_no_response	with_no_response	with_no_response
GSM972292	SFM-C	late.TA	epi	top	undetermined	with_no_response	undetermined	undetermined	with_no_response	with_no_response	with_no_response	with_no_response	undetermined
GSM972294	SFM-C	undetermined	undetermined	top	sCRC	with_no_response	undetermined	undetermined	with_no_response	with_no_response	with_no_response	with_no_response	with_response
GSM972298	SFM-C	late.TA	epi	top	sCRC	with_no_response	with_response	with_response	with_no_response	with_no_response	with_no_response	with_no_response	with_no_response
GSM972305	SFM-C	late.TA	epi	top	sCRC	with_no_response	undetermined	undetermined	with_no_response	with_no_response	with_no_response	with_no_response	with_no_response
GSM972316	SFM-C	late.TA	epi	top	sCRC	with_no_response	with_response	with_response	with_no_response	with_no_response	with_no_response	with_no_response	with_no_response
GSM972317	SFM-C	undetermined	epi	top	sCRC	with_no_response	undetermined	undetermined	with_no_response	with_no_response	with_no_response	with_no_response	with_response
GSM972325	SFM-C	undetermined	undetermined	base	sCRC	with_no_response	with_response	with_response	with_no_response	with_no_response	with_no_response	with_no_response	with_no_response
GSM972332	SFM-C	undetermined	emt	top	sCRC	with_response	undetermined	with_response	with_no_response	with_no_response	with_no_response	with_no_response	undetermined
GSM972346	SFM-C	stem	epi	top	sCRC	with_no_response	undetermined	undetermined	with_no_response	with_no_response	with_no_response	with_no_response	undetermined
GSM972364	SFM-C	undetermined	epi	top	undetermined	with_no_response	with_response	with_response	with_no_response	with_no_response	with_no_response	with_no_response	with_no_response
GSM972391	SFM-C	undetermined	epi	top	undetermined	with_no_response	with_response	undetermined	with_no_response	undetermined	with_no_response	with_no_response	undetermined
GSM972403	SFM-C	late.TA	epi	top	sCRC	with_no_response	with_response	undetermined	undetermined	undetermined	with_no_response	undetermined	with_response
GSM972406	SFM-C	stem	undetermined	base	sCRC	with_response	with_response	with_response	undetermined	undetermined	with_no_response	with_no_response	undetermined
GSM972408	SFM-C	undetermined	epi	top	sCRC	with_response	undetermined	undetermined	with_no_response	with_no_response	with_no_response	with_no_response	with_response
GSM972430	SFM-C	undetermined	undetermined	top	sCRC	with_no_response	with_response	undetermined	with_no_response	with_no_response	with_no_response	with_no_response	with_response
GSM972445	SFM-C	stem	emt	base	undetermined	undetermined	with_response	with_response	with_response	with_response	with_no_response	with_no_response	with_response
GSM972446	SFM-C	undetermined	emt	undetermined	sCRC	undetermined	undetermined	undetermined	with_no_response	undetermined	with_no_response	with_response	with_no_response
GSM972447	SFM-C	late.TA	epi	undetermined	sCRC	with_response	with_response	with_response	with_no_response	with_no_response	with_no_response	with_no_response	with_no_response
GSM972448	SFM-C	late.TA	undetermined	top	sCRC	with_no_response	undetermined	undetermined	with_no_response	with_no_response	with_no_response	with_no_response	undetermined
GSM972452	SFM-C	stem	emt	base	sCRC	with_response	with_response	with_response	with_no_response	with_no_response	with_no_response	with_no_response	with_response
GSM972458	SFM-C	undetermined	emt	base	sCRC	undetermined	with_response	undetermined	with_response	with_response	with_no_response	with_no_response	with_response
GSM972465	SFM-C	stem	epi	base	undetermined	with_no_response	with_response	with_response	with_no_response	with_no_response	with_no_response	with_no_response	with_no_response
GSM972471	SFM-C	late.TA	epi	top	sCRC	with_no_response	with_response	with_response	with_no_response	with_no_response	with_no_response	with_no_response	with_no_response
GSM972472	SFM-C	stem	undetermined	base	sCRC	with_no_response	with_response	undetermined	with_no_response	with_no_response	with_no_response	with_no_response	with_no_response
GSM972473	SFM-C	late.TA	epi	undetermined	sCRC	undetermined	undetermined	with_response	with_no_response	with_no_response	with_no_response	with_no_response	with_no_response
GSM972488	SFM-C	undetermined	epi	top	sCRC	with_no_response	with_response	with_response	with_no_response	with_no_response	with_no_response	with_no_response	with_response
GSM972499	SFM-C	undetermined	epi	top	sCRC	with_no_response	with_response	undetermined	with_no_response	with_no_response	with_no_response	with_no_response	undetermined
GSM972507	SFM-C	late.TA	epi	undetermined	sCRC	with_no_response	with_response	with_response	with_no_response	with_no_response	with_no_response	with_no_response	with_no_response
GSM358376	SFM-C	undetermined	epi	top	undetermined	with_no_response	undetermined	undetermined	with_no_response	with_no_response	with_no_response	with_no_response	with_no_response
GSM358377	SFM-C	stem	epi	undetermined	sCRC	with_no_response	undetermined	with_response	with_no_response	with_no_response	with_no_response	with_no_response	with_no_response
GSM358381	SFM-C	late.TA	epi	undetermined	undetermined	with_no_response	undetermined	undetermined	with_response	with_response	with_no_response	with_no_response	with_no_response
GSM358395	SFM-C	undetermined	emt	base	sCRC	with_response	undetermined	with_no_response	with_no_response	with_no_response	with_no_response	with_no_response	with_response
GSM358422	SFM-C	late.TA	epi	top	undetermined	with_no_response	undetermined	with_no_response	with_response	with_response	undetermined	undetermined	undetermined
GSM358442	SFM-C	stem	emt	top	sCRC	with_no_response	undetermined	undetermined	with_no_response	with_no_response	with_no_response	with_no_response	with_no_response
GSM358447	SFM-C	undetermined	emt	top	undetermined	undetermined	undetermined	undetermined	with_no_response	with_no_response	with_no_response	with_no_response	with_response
GSM358463	SFM-C	undetermined	epi	base	undetermined	with_no_response	undetermined	undetermined	with_response	undetermined	with_no_response	with_response	undetermined
GSM358477	SFM-C	undetermined	emt	top	sCRC	with_no_response	undetermined	undetermined	with_no_response	with_no_response	with_no_response	with_no_response	with_response
GSM358492	SFM-C	undetermined	emt	base	sCRC	with_response	undetermined	undetermined	with_response	with_response	with_response	undetermined	with_response
GSM358502	SFM-C	undetermined	epi	top	sCRC	with_no_response	undetermined	undetermined	with_no_response	with_no_response	with_no_response	with_no_response	with_response
GSM358554	SFM-C	undetermined	epi	top	sCRC	with_no_response	undetermined	undetermined	with_no_response	with_no_response	with_no_response	with_no_response	with_response
GSM358559	SFM-C	undetermined	epi	top	nsCRC	with_no_response	undetermined	with_response	with_response	with_response	with_response	with_response	with_response
GSM358608	SFM-C	stem	emt	base	undetermined	with_response	undetermined	undetermined	with_no_response	with_no_response	with_no_response	with_no_response	with_no_response
GSM358628	SFM-C	stem	emt	base	undetermined	undetermined	undetermined	undetermined	undetermined	with_no_response	with_no_response	with_response	with_response
GSM437105	SFM-C	late.TA	epi	top	sCRC	with_no_response	undetermined	undetermined	with_no_response	undetermined	with_no_response	with_no_response	with_no_response
GSM437118	SFM-C	undetermined	emt	top	undetermined	with_no_response	undetermined	undetermined	with_no_response	with_no_response	with_no_response	with_no_response	with_response
GSM437126	SFM-C	late.TA	epi	top	undetermined	with_no_response	undetermined	with_response	undetermined	undetermined	with_no_response	with_no_response	with_no_response
GSM437133	SFM-C	undetermined	emt	top	sCRC	with_no_response	undetermined	undetermined	with_no_response	with_no_response	with_no_response	with_no_response	with_response
GSM437143	SFM-C	undetermined	epi	undetermined	sCRC	with_no_response	undetermined	with_response	undetermined	undetermined	with_no_response	undetermined	with_response
GSM437145	SFM-C	stem	emt	base	sCRC	with_response	undetermined	undetermined	with_no_response	with_no_response	with_no_response	with_no_response	with_response
GSM437147	SFM-C	undetermined	epi	top	sCRC	with_no_response	undetermined	undetermined	with_no_response	with_no_response	with_no_response	with_no_response	undetermined
GSM437148	SFM-C	late.TA	epi	top	sCRC	with_no_response	undetermined	with_no_response	with_response	with_response	with_no_response	with_response	with_no_response
GSM437155	SFM-C	undetermined	emt	base	undetermined	with_response	undetermined	undetermined	undetermined	undetermined	with_no_response	with_no_response	with_response
GSM437156	SFM-C	late.TA	epi	top	sCRC	with_no_response	undetermined	undetermined	with_no_response	with_no_response	with_no_response	with_no_response	with_no_response
GSM437160	SFM-C	undetermined	epi	top	nsCRC	with_no_response	undetermined	with_response	undetermined	with_response	with_no_response	with_no_response	with_no_response
GSM437176	SFM-C	late.TA	epi	top	undetermined	undetermined	undetermined	undetermined	with_no_response	with_no_response	with_no_response	with_no_response	with_no_response
GSM437178	SFM-C	undetermined	emt	base	sCRC	with_response	undetermined	undetermined	with_no_response	undetermined	with_no_response	with_no_response	with_no_response
GSM437184	SFM-C	undetermined	epi	top	sCRC	with_no_response	undetermined	with_response	with_no_response	with_no_response	with_no_response	with_no_response	with_response
GSM437196	SFM-C	late.TA	epi	base	sCRC	undetermined	undetermined	undetermined	undetermined	with_no_response	with_no_response	with_response	undetermined
GSM437203	SFM-C	undetermined	emt	top	sCRC	with_response	undetermined	undetermined	with_response	with_response	with_no_response	undetermined	with_response
GSM437209	SFM-C	undetermined	emt	top	sCRC	with_response	undetermined	undetermined	with_no_response	with_no_response	with_no_response	with_no_response	with_response
GSM437212	SFM-C	undetermined	epi	top	sCRC	with_no_response	undetermined	undetermined	with_no_response	with_no_response	with_no_response	with_no_response	with_no_response
GSM437213	SFM-C	undetermined	undetermined	base	undetermined	with_no_response	undetermined	undetermined	with_no_response	with_no_response	with_no_response	with_response	undetermined
GSM437225	SFM-C	stem	emt	base	sCRC	with_response	undetermined	undetermined	with_no_response	with_no_response	with_no_response	with_no_response	with_response
GSM437254	SFM-C	undetermined	emt	top	undetermined	with_no_response	undetermined	with_no_response	undetermined	undetermined	with_response	with_response	with_response
GSM437267	SFM-C	stem	emt	base	sCRC	undetermined	undetermined	undetermined	with_no_response	with_no_response	with_no_response	with_no_response	with_no_response
GSM437273	SFM-C	undetermined	epi	top	undetermined	with_no_response	undetermined	with_response	with_no_response	undetermined	with_no_response	with_no_response	with_no_response
GSM437288	SFM-C	undetermined	epi	top	sCRC	with_no_response	undetermined	undetermined	with_no_response	with_no_response	with_no_response	undetermined	with_no_response
GSM437294	SFM-C	undetermined	undetermined	undetermined	sCRC	undetermined	undetermined	with_response	undetermined	with_no_response	with_no_response	with_no_response	with_response
GSM437297	SFM-C	undetermined	emt	base	sCRC	with_response	undetermined	with_response	with_no_response	with_no_response	with_no_response	with_no_response	with_response
GSM437307	SFM-C	undetermined	emt	undetermined	sCRC	with_response	undetermined	undetermined	with_no_response	with_no_response	with_no_response	with_no_response	with_response
GSM437309	SFM-C	undetermined	epi	base	undetermined	with_no_response	undetermined	undetermined	with_response	with_response	with_no_response	undetermined	with_no_response
GSM437313	SFM-C	stem	undetermined	base	sCRC	with_response	undetermined	undetermined	undetermined	with_no_response	undetermined	with_response	undetermined
GSM820068	SFM-C	late.TA	epi	top	undetermined	with_no_response	undetermined	undetermined	with_no_response	with_no_response	with_no_response	with_no_response	with_no_response
GSM820071	SFM-C	stem	emt	base	undetermined	undetermined	undetermined	undetermined	with_response	with_response	with_response	with_response	with_response
GSM820074	SFM-C	undetermined	emt	base	undetermined	with_no_response	undetermined	with_no_response	with_response	with_response	with_response	with_response	with_response
GSM820075	SFM-C	late.TA	emt	undetermined	undetermined	with_no_response	undetermined	undetermined	with_no_response	undetermined	with_no_response	with_no_response	with_response
GSM820080	SFM-C	undetermined	epi	undetermined	sCRC	with_no_response	undetermined	undetermined	with_no_response	with_no_response	with_no_response	with_no_response	with_no_response
GSM820096	SFM-C	undetermined	emt	undetermined	sCRC	with_response	undetermined	undetermined	with_no_response	undetermined	with_no_response	with_no_response	undetermined
GSM820098	SFM-C	undetermined	epi	base	sCRC	with_response	undetermined	with_response	with_no_response	with_no_response	with_no_response	with_no_response	undetermined
GSM820105	SFM-C	undetermined	epi	top	nsCRC	with_no_response	undetermined	undetermined	undetermined	with_no_response	with_no_response	with_no_response	undetermined
GSM820114	SFM-C	late.TA	epi	top	sCRC	with_no_response	undetermined	undetermined	undetermined	undetermined	with_no_response	with_no_response	with_no_response
GSM820118	SFM-C	stem	emt	top	undetermined	with_no_response	undetermined	undetermined	with_no_response	with_no_response	with_no_response	with_no_response	with_no_response
GSM820121	SFM-C	stem	emt	undetermined	sCRC	undetermined	undetermined	undetermined	with_no_response	with_no_response	with_no_response	with_no_response	with_response
GSM820122	SFM-C	stem	emt	top	undetermined	with_response	undetermined	undetermined	with_response	with_response	with_response	with_response	with_response
GSM820126	SFM-C	stem	emt	undetermined	nsCRC	with_no_response	undetermined	undetermined	with_no_response	undetermined	undetermined	with_response	with_response
GSM820135	SFM-C	undetermined	undetermined	top	sCRC	with_response	undetermined	undetermined	with_no_response	with_no_response	with_no_response	with_no_response	with_response
GSM929496	SFM-C	stem	emt	base	undetermined	with_no_response	undetermined	undetermined	undetermined	undetermined	undetermined	with_response	with_response
GSM929510	SFM-C	undetermined	epi	undetermined	sCRC	with_no_response	undetermined	with_response	with_response	with_response	with_no_response	with_no_response	with_no_response
GSM929521	SFM-C	undetermined	emt	base	sCRC	with_response	undetermined	undetermined	undetermined	with_no_response	with_no_response	undetermined	with_response
GSM929531	SFM-C	late.TA	undetermined	top	sCRC	undetermined	undetermined	with_response	with_no_response	with_no_response	with_no_response	with_no_response	with_no_response

Supplementar Table 7. Differential expression analysis of each SFM subtype versus the rest subtypes

SFM-A								SFM-B								SFM-C								SFM-D								SFM-E								SFM-F							
	logFC	AveExpr	t	P.Value	adj.P.Val	B	FC		logFC	AveExpr	t	P.Value	adj.P.Val	B	FC		logFC	AveExpr	t	P.Value	adj.P.Val	B	FC		logFC	AveExpr	t	P.Value	adj.P.Val	B	FC		logFC	AveExpr	t	P.Value	adj.P.Val	B	FC		logFC	AveExpr	t	P.Value	adj.P.Val	B	FC
[illegible]	[illegible]	[illegible]	[illegible]	[illegible]	[illegible]	[illegible]	[illegible]	[illegible]	[illegible]	[illegible]	[illegible]	[illegible]	[illegible]	[illegible]	[illegible]	[illegible]	[illegible]	[illegible]	[illegible]	[illegible]	[illegible]	[illegible]	[illegible]	[illegible]	[illegible]	[illegible]	[illegible]	[illegible]	[illegible]	[illegible]	[illegible]	[illegible]	[illegible]	[illegible]	[illegible]	[illegible]	[illegible]	[illegible]	[illegible]	[illegible]	[illegible]	[illegible]	[illegible]	[illegible]	[illegible]	[illegible]	[illegible]

Supplementary Table 8. Classification of four drug-associated datasets according to the SFM using K-means algorithm

A

GSE62080 dataset (FOLFIRI)		
sample_id	Response	SFM subtype
GSM1519418	S	SFM-E
GSM1519430	S	SFM-E
GSM1519437	S	SFM-E
GSM1519419	R	SFM-B
GSM1519421	R	SFM-B
GSM1519422	R	SFM-B
GSM1519425	R	SFM-B
GSM1519426	R	SFM-B
GSM1519427	R	SFM-B
GSM1519431	S	SFM-B
GSM1519432	R	SFM-B
GSM1519433	R	SFM-B
GSM1519438	R	SFM-B
GSM1519420	S	SFM-F
GSM1519423	S	SFM-F
GSM1519424	S	SFM-F
GSM1519428	S	SFM-F
GSM1519429	S	SFM-F
GSM1519434	R	SFM-A
GSM1519435	R	SFM-A
GSM1519436	R	SFM-A

B

GSE19860, GSE28702 and GSE69657 datasets (FOLFOX)			
sample_id	FL_response	GEO_id	SFM subtype
GSM1705393	noresponder	GSE69657	SFM-C
GSM1705409	noresponder	GSE69657	SFM-C
GSM1705410	responder	GSE69657	SFM-C
GSM1705413	responder	GSE69657	SFM-C
GSM1705417	noresponder	GSE69657	SFM-C
GSM1705418	noresponder	GSE69657	SFM-C
GSM496017	noresponder	GSE19860	SFM-C
GSM496019	noresponder	GSE19860	SFM-C
GSM496021	responder	GSE19860	SFM-C
GSM496024	noresponder	GSE19860	SFM-C
GSM496031	noresponder	GSE19860	SFM-C
GSM496033	noresponder	GSE19860	SFM-C
GSM496035	noresponder	GSE19860	SFM-C
GSM496041	responder	GSE19860	SFM-C
GSM710819	responder	GSE28702	SFM-C
GSM710833	noresponder	GSE28702	SFM-C
GSM710836	noresponder	GSE28702	SFM-C
GSM710837	noresponder	GSE28702	SFM-C
GSM710853	noresponder	GSE28702	SFM-C
GSM710855	noresponder	GSE28702	SFM-C
GSM710869	noresponder	GSE28702	SFM-C
GSM710873	noresponder	GSE28702	SFM-C
GSM710877	responder	GSE28702	SFM-C
GSM710883	responder	GSE28702	SFM-C
GSM710885	responder	GSE28702	SFM-C
GSM710892	responder	GSE28702	SFM-C
GSM710911	noresponder	GSE28702	SFM-C
GSM710922	noresponder	GSE28702	SFM-C
GSM710924	noresponder	GSE28702	SFM-C
GSM1705392	responder	GSE69657	SFM-E
GSM1705407	responder	GSE69657	SFM-E
GSM1705416	responder	GSE69657	SFM-E
GSM496016	noresponder	GSE19860	SFM-E
GSM496023	noresponder	GSE19860	SFM-E
GSM496025	noresponder	GSE19860	SFM-E
GSM496036	responder	GSE19860	SFM-E
GSM496037	noresponder	GSE19860	SFM-E
GSM496038	noresponder	GSE19860	SFM-E
GSM496039	responder	GSE19860	SFM-E
GSM710805	responder	GSE28702	SFM-E
GSM710806	responder	GSE28702	SFM-E
GSM710820	responder	GSE28702	SFM-E
GSM710829	noresponder	GSE28702	SFM-E
GSM710835	noresponder	GSE28702	SFM-E
GSM710841	noresponder	GSE28702	SFM-E
GSM710846	noresponder	GSE28702	SFM-E
GSM710849	noresponder	GSE28702	SFM-E
GSM710858	noresponder	GSE28702	SFM-E
GSM710898	responder	GSE28702	SFM-E
GSM710902	responder	GSE28702	SFM-E
GSM710908	noresponder	GSE28702	SFM-E
GSM710913	noresponder	GSE28702	SFM-E
GSM710915	noresponder	GSE28702	SFM-E
GSM710916	noresponder	GSE28702	SFM-E
GSM710926	noresponder	GSE28702	SFM-E
GSM710928	noresponder	GSE28702	SFM-E
GSM710930	noresponder	GSE28702	SFM-E
GSM1705390	noresponder	GSE69657	SFM-A
GSM1705403	noresponder	GSE69657	SFM-A
GSM1705405	noresponder	GSE69657	SFM-A
GSM1705408	noresponder	GSE69657	SFM-A
GSM1705411	responder	GSE69657	SFM-A
GSM1705412	noresponder	GSE69657	SFM-A
GSM1705414	noresponder	GSE69657	SFM-A
GSM1705415	noresponder	GSE69657	SFM-A
GSM496020	responder	GSE19860	SFM-A
GSM496022	noresponder	GSE19860	SFM-A
GSM496027	responder	GSE19860	SFM-A
GSM496028	noresponder	GSE19860	SFM-A
GSM496029	noresponder	GSE19860	SFM-A
GSM496032	noresponder	GSE19860	SFM-A
GSM496040	responder	GSE19860	SFM-A
GSM496042	noresponder	GSE19860	SFM-A
GSM496043	responder	GSE19860	SFM-A
GSM710802	responder	GSE28702	SFM-A
GSM710803	responder	GSE28702	SFM-A
GSM710808	responder	GSE28702	SFM-A
GSM710810	responder	GSE28702	SFM-A
GSM710811	responder	GSE28702	SFM-A
GSM710812	responder	GSE28702	SFM-A
GSM710818	responder	GSE28702	SFM-A
GSM710825	responder	GSE28702	SFM-A
GSM710845	noresponder	GSE28702	SFM-A
GSM710860	noresponder	GSE28702	SFM-A
GSM710862	noresponder	GSE28702	SFM-A
GSM710865	noresponder	GSE28702	SFM-A
GSM710867	noresponder	GSE28702	SFM-A
GSM710871	noresponder	GSE28702	SFM-A
GSM710905	noresponder	GSE28702	SFM-A
GSM710906	noresponder	GSE28702	SFM-A
GSM1705397	responder	GSE69657	SFM-F
GSM1705399	responder	GSE69657	SFM-F
GSM1705406	responder	GSE69657	SFM-F
GSM496018	noresponder	GSE19860	SFM-F
GSM710804	responder	GSE28702	SFM-F
GSM710809	responder	GSE28702	SFM-F
GSM710817	responder	GSE28702	SFM-F
GSM710831	noresponder	GSE28702	SFM-F
GSM1705391	noresponder	GSE69657	SFM-B
GSM1705394	noresponder	GSE69657	SFM-B
GSM1705395	noresponder	GSE69657	SFM-B
GSM1705396	responder	GSE69657	SFM-B
GSM1705398	responder	GSE69657	SFM-B
GSM496015	noresponder	GSE19860	SFM-B
GSM496026	noresponder	GSE19860	SFM-B
GSM496030	responder	GSE19860	SFM-B
GSM496034	noresponder	GSE19860	SFM-B
GSM710807	responder	GSE28702	SFM-B
GSM710822	responder	GSE28702	SFM-B
GSM710823	responder	GSE28702	SFM-B
GSM710824	responder	GSE28702	SFM-B
GSM710826	responder	GSE28702	SFM-B
GSM710827	responder	GSE28702	SFM-B
GSM710863	noresponder	GSE28702	SFM-B
GSM710886	responder	GSE28702	SFM-B
GSM710888	responder	GSE28702	SFM-B
GSM710890	responder	GSE28702	SFM-B
GSM710894	responder	GSE28702	SFM-B
GSM710896	responder	GSE28702	SFM-B
GSM710920	noresponder	GSE28702	SFM-B
GSM1705389	noresponder	GSE69657	SFM-D
GSM1705400	responder	GSE69657	SFM-D
GSM1705401	responder	GSE69657	SFM-D
GSM1705402	noresponder	GSE69657	SFM-D
GSM1705404	noresponder	GSE69657	SFM-D
GSM710801	responder	GSE28702	SFM-D
GSM710813	responder	GSE28702	SFM-D
GSM710814	responder	GSE28702	SFM-D
GSM710815	responder	GSE28702	SFM-D
GSM710816	responder	GSE28702	SFM-D
GSM710821	responder	GSE28702	SFM-D
GSM710828	noresponder	GSE28702	SFM-D
GSM710830	noresponder	GSE28702	SFM-D
GSM710832	noresponder	GSE28702	SFM-D
GSM710834	noresponder	GSE28702	SFM-D
GSM710839	noresponder	GSE28702	SFM-D
GSM710843	noresponder	GSE28702	SFM-D
GSM710875	responder	GSE28702	SFM-D
GSM710879	responder	GSE28702	SFM-D
GSM710881	responder	GSE28702	SFM-D
GSM710900	responder	GSE28702	SFM-D
GSM710918	noresponder	GSE28702	SFM-D

C

GSE104645, GSE72970 datasets (FOLFOX or FOLFIRI)									
sample_id	treatment	response	GEO_id	response.agg	sfm	pfs.status	pfs	os.status	os
GSM2804851	FOLFOX	PR	GSE104645	response	SFM-A/B	0	15.83333	0	38.4
GSM2804866	FOLFOX	SD	GSE104645	non.response	SFM-A/B	1	6.8	1	29.9
GSM2804880	FOLFOX	PR	GSE104645	response	SFM-A/B	0	23.5	1	35.4
GSM2804930	FOLFOX	PD	GSE104645	non.response	SFM-A/B	0	1.466667	1	14.76667
GSM1875897	FOLFOX	PR	GSE72970	response	SFM-A/B	1	5.585216	1	5.585216
GSM1875899	FOLFOX	PD	GSE72970	non.response	SFM-A/B	1	1.87269	1	2.49692
GSM1875905	FOLFIRI	SD	GSE72970	non.response	SFM-A/B	1	3.055441	1	3.449692
GSM1875906	FOLFIRI	SD	GSE72970	non.response	SFM-A/B	1	14.65298	1	41.85626
GSM1875918	FOLFOX	PR	GSE72970	response	SFM-A/B	1	13.40452	0	47.83573
GSM1875919	FOLFOX	PR	GSE72970	response	SFM-A/B	1	4.336756	1	4.336756
GSM1875921	FOLFIRI	PD	GSE72970	non.response	SFM-A/B	1	1.64271	1	19.64682
GSM1875929	FOLFOX	PR	GSE72970	response	SFM-A/B	1	10.87474	1	17.1499
GSM1875930	FOLFIRI	PD	GSE72970	non.response	SFM-A/B	1	1.87269	1	25.79055
GSM1875933	FOLFIRI	PR	GSE72970	response	SFM-A/B	0	26.71047	0	26.71047
GSM1875937	FOLFOX	SD	GSE72970	non.response	SFM-A/B	1	4.731006	1	18.79261
GSM1875938	FOLFOX	PD	GSE72970	non.response	SFM-A/B	1	3.022587	1	11.03901
GSM1875939	FOLFIRI	SD	GSE72970	non.response	SFM-A/B	1	4.271047	1	15.34292
GSM1875940	FOLFIRI	PR	GSE72970	response	SFM-A/B	1	9.494866	0	15.77002
GSM1875941	FOLFIRI	PR	GSE72970	response	SFM-A/B	1	13.40452	1	42.08624
GSM1875943	FOLFIRI	PR	GSE72970	response	SFM-A/B	1	10.94045	1	24.80493
GSM1875944	FOLFIRI	PD	GSE72970	non.response	SFM-A/B	1	2.004107	1	12.38604
GSM1875946	FOLFIRI	PR	GSE72970	response	SFM-A/B	1	8.213552	0	19.12115
GSM1875951	FOLFIRI	SD	GSE72970	non.response	SFM-A/B	1	4.76386	1	6.373717
GSM1875952	FOLFOX	SD	GSE72970	non.response	SFM-A/B	1	7.26078	1	13.43737
GSM1875953	FOLFIRI	PD	GSE72970	non.response	SFM-A/B	1	1.708419	1	5.486653
GSM1875954	FOLFOX	PR	GSE72970	response	SFM-A/B	1	13.14168	0	37.78234
GSM1875956	FOLFOX	PR	GSE72970	response	SFM-A/B	1	7.687885	1	14.75154
GSM1875960	FOLFIRI	PD	GSE72970	non.response	SFM-A/B	1	1.905544	1	19.21971
GSM1875961	FOLFIRI	SD	GSE72970	non.response	SFM-A/B	1	3.646817	1	19.90965
GSM1875962	FOLFIRI	CR	GSE72970	response	SFM-A/B	1	48.09856	0	50.39836
GSM1875965	FOLFIRI	SD	GSE72970	non.response	SFM-A/B	1	3.679672	1	17.47844
GSM1875967	FOLFIRI	SD	GSE72970	non.response	SFM-A/B	1	5.256673	1	6.439425
GSM1875969	FOLFOX	PR	GSE72970	response	SFM-A/B	1	14.71869	1	20.50103
GSM1875972	FOLFOX	PR	GSE72970	response	SFM-A/B	1	15.01437	1	28.55031
GSM1875973	FOLFIRI	PD	GSE72970	non.response	SFM-A/B	1	2.464066	1	2.891171
GSM1875974	FOLFIRI	PD	GSE72970	non.response	SFM-A/B	1	1.741273	1	2.26694
GSM1875976	FOLFIRI	SD	GSE72970	non.response	SFM-A/B	1	6.406571	1	6.406571
GSM1875977	FOLFIRI	SD	GSE72970	non.response	SFM-A/B	1	21.15811	0	57.7577
GSM1875978	FOLFIRI	SD	GSE72970	non.response	SFM-A/B	1	7.622177	1	11.03901
GSM1875981	FOLFOX	PR	GSE72970	response	SFM-A/B	1	25.42916	0	66.59548
GSM1875983	FOLFIRI	SD	GSE72970	non.response	SFM-A/B	1	5.913758	1	23.55647
GSM1875989	FOLFOX	SD	GSE72970	non.response	SFM-A/B	0	48.03286	0	48.03286
GSM1876008	FOLFOX	SD	GSE72970	non.response	SFM-A/B	1	22.70226	1	36.07392
GSM1876009	FOLFOX	PD	GSE72970	non.response	SFM-A/B	1	1.577002	1	7.556468
GSM1876011	FOLFIRI	PR	GSE72970	response	SFM-A/B	1	8.772074	1	31.73717
GSM1876012	FOLFIRI	PR	GSE72970	response	SFM-A/B	1	27.46612	1	29.24025
GSM2804850	FOLFOX	SD	GSE104645	non.response	SFM-D	0	7.8	1	30.13333
GSM2804853	FOLFOX	PD	GSE104645	non.response	SFM-D	0	2.266667	1	9.933333
GSM2804857	FOLFOX	PR	GSE104645	response	SFM-D	0	8.333333	1	21.36667
GSM2804867	FOLFOX	SD	GSE104645	non.response	SFM-D	1	22.86667	1	51.76667
GSM2804868	FOLFOX	SD	GSE104645	non.response	SFM-D	0	18.66667	1	64.46667
GSM2804873	FOLFOX	PR	GSE104645	response	SFM-D	0	11.43333	0	44.8
GSM2804882	FOLFIRI	PR	GSE104645	response	SFM-D	0	7.466667	1	73.7
GSM2804889	FOLFOX	PD	GSE104645	non.response	SFM-D	0	2.7	0	30.53333
GSM2804891	FOLFOX	PR	GSE104645	response	SFM-D	0	10.06667	1	24.93333
GSM2804948	FOLFOX	PR	GSE104645	response	SFM-D	0	14.7	0	43.96667
GSM2804981	FOLFIRI	SD	GSE104645	non.response	SFM-D	0	6.166667	1	18.3
GSM2804987	FOLFOX	SD	GSE104645	non.response	SFM-D	0	14.7	0	22.83333
GSM2804992	FOLFOX	PR	GSE104645	response	SFM-D	0	29.23333	0	33.46667
GSM2805004	FOLFOX	SD	GSE104645	non.response	SFM-D	0	5.6	0	35.9
GSM2805016	FOLFOX	PR	GSE104645	response	SFM-D	0	10.73333	1	10.96667
GSM1875898	FOLFOX	PR	GSE72970	response	SFM-D	1	8.476386	1	13.66735
GSM1875900	FOLFOX	PR	GSE72970	response	SFM-D	0	70.01232	0	70.01232
GSM1875901	FOLFIRI	PR	GSE72970	response	SFM-D	1	7.556468	1	19.9425
GSM1875902	FOLFOX	PR	GSE72970	response	SFM-D	1	17.60986	1	58.74333
GSM1875903	FOLFIRI	SD	GSE72970	non.response	SFM-D	1	7.556468	1	9.560575
GSM1875904	FOLFIRI	CR	GSE72970	response	SFM-D	0	46.29158	0	46.29158
GSM1875907	FOLFOX	SD	GSE72970	non.response	SFM-D	1	6.767967	1	9.067761
GSM1875913	FOLFIRI	SD	GSE72970	non.response	SFM-D	1	4.829569	1	13.07598
GSM1875915	FOLFIRI	CR	GSE72970	response	SFM-D	1	9.232033	0	52.40247
GSM1875916	FOLFOX	PR	GSE72970	response	SFM-D	1	7.88501	1	15.93429
GSM1875920	FOLFOX	PR	GSE72970	response	SFM-D	0	39.55647	0	39.55647
GSM1875922	FOLFIRI	SD	GSE72970	non.response	SFM-D	1	8.73922	1	19.81109
GSM1875923	FOLFOX	PR	GSE72970	response	SFM-D	1	16.55852	1	37.97947
GSM1875924	FOLFOX	PR	GSE72970	response	SFM-D	1	12.28747	1	37.19096
GSM1875925	FOLFIRI	PD	GSE72970	non.response	SFM-D	1	2.26694	1	17.97125
GSM1875926	FOLFIRI	SD	GSE72970	non.response	SFM-D	1	5.848049	1	18.69405
GSM1875927	FOLFIRI	SD	GSE72970	non.response	SFM-D	1	4.829569	1	5.880904
GSM1875928	FOLFIRI	SD	GSE72970	non.response	SFM-D	1	9.396304	1	28.94456
GSM1875931	FOLFIRI	PR	GSE72970	response	SFM-D	1	10.1191	0	33.5113
GSM1875932	FOLFOX	PR	GSE72970	response	SFM-D	1	20.79671	1	23.78645
GSM1875934	FOLFIRI	SD	GSE72970	non.response	SFM-D	1	6.045175	0	24.67351
GSM1875935	FOLFOX	SD	GSE72970	non.response	SFM-D	0	24.90349	0	24.90349
GSM1875936	FOLFIRI	PR	GSE72970	response	SFM-D	1	11.46612	0	31.2115
GSM1875950	FOLFIRI	PR	GSE72970	response	SFM-D	1	9.034907	1	18.43121
GSM1875957	FOLFIRI	CR	GSE72970	response	SFM-D	1	18.26694	1	59.82751
GSM2804849	FOLFOX	PR	GSE104645	response	SFM-C	0	10.93333	0	22.43333
GSM2804854	FOLFOX	PR	GSE104645	response	SFM-C	0	5.333333	0	16.83333
GSM2804856	FOLFOX	SD	GSE104645	non.response	SFM-C	0	4.933333	1	59.53333
GSM2804858	FOLFOX	PR	GSE104645	response	SFM-C	0	15.63333	0	81.73333
GSM2804859	FOLFOX	PR	GSE104645	response	SFM-C	0	7.066667	0	26.03333
GSM2804864	FOLFOX	PR	GSE104645	response	SFM-C	0	11.3	0	57.96667
GSM2804878	FOLFOX	PR	GSE104645	response	SFM-C	0	14.46667	0	23.46667
GSM2804886	FOLFOX	PD	GSE104645	non.response	SFM-C	0	1.733333	1	7.133333
GSM2804890	FOLFOX	PR	GSE104645	response	SFM-C	0	23.1	0	32.96667
GSM2804897	FOLFOX	SD	GSE104645	non.response	SFM-C	0	5.266667	1	12.86667
GSM2804912	FOLFOX	PR	GSE104645	response	SFM-C	0	24.93333	1	55.3
GSM2804922	FOLFOX	PR	GSE104645	response	SFM-C	0	4.2	0	10.5
GSM2804944	FOLFIRI	PR	GSE104645	response	SFM-C	0	6.533333	0	26.96667
GSM2804972	FOLFOX	PR	GSE104645	response	SFM-C	0	11.2	0	27.3
GSM2804978	FOLFIRI	PR	GSE104645	response	SFM-C	0	12.83333	0	34.7
GSM2804999	FOLFOX	PR	GSE104645	response	SFM-C	0	9.7	0	14.6
GSM2805010	FOLFOX	PR	GSE104645	response	SFM-C	0	4.666667	0	39
GSM2805019	FOLFOX	SD	GSE104645	non.response	SFM-C	1	9.6	0	48.06667
GSM1875958	FOLFIRI	CR	GSE72970	response	SFM-C	1	7.950719	0	55.98357
GSM2804870	FOLFOX	SD	GSE104645	non.response	SFM-E	0	5.166667	1	82.13333
GSM2804872	FOLFOX	SD	GSE104645	non.response	SFM-E	0	30.33333	1	36.03333
GSM2804875	FOLFIRI	SD	GSE104645	non.response	SFM-E	0	5.3	1	13.8
GSM2804938	FOLFOX	SD	GSE104645	non.response	SFM-E	0	8.433333	0	32.33333
GSM2804939	FOLFOX	PR	GSE104645	response	SFM-E	0	24.1	0	45.56667
GSM2804957	FOLFIRI	PR	GSE104645	response	SFM-E	0	38.23333	0	50.93333
GSM2804958	FOLFOX	PR	GSE104645	response	SFM-E	0	25.43333	0	59.33333
GSM2804959	FOLFIRI	SD	GSE104645	non.response	SFM-E	0	3.766667	0	34.76667
GSM2804965	FOLFOX	PR	GSE104645	response	SFM-E	0	17.93333	1	22.66667
GSM2804979	FOLFIRI	PR	GSE104645	response	SFM-E	0	2.9	0	49.7
GSM2804983	FOLFOX	PD	GSE104645	non.response	SFM-E	0	1.333333	0	7
GSM2804995	FOLFIRI	PR	GSE104645	response	SFM-E	0	14.26667	0	18.03333
GSM2804997	FOLFOX	PR	GSE104645	response	SFM-E	0	6.433333	0	26.36667
GSM2804998	FOLFIRI	PR	GSE104645	response	SFM-E	0	9.133333	0	43.7
GSM2805003	FOLFOX	PR	GSE104645	response	SFM-E	1	7.2	1	7.3
GSM2805011	FOLFOX	SD	GSE104645	non.response	SFM-E	0	8.433333	0	12.56667
GSM2805032	FOLFOX	PD	GSE104645	non.response	SFM-E	0	1.633333	0	14.56667
GSM2805036	FOLFOX	PR	GSE104645	response	SFM-E	0	16.26667	1	86.03333
GSM2804847	FOLFOX	SD	GSE104645	non.response	SFM-F	0	18.2	0	55.96667
GSM2804860	FOLFOX	PR	GSE104645	response	SFM-F	0	21	0	65.6
GSM2804862	FOLFOX	CR	GSE104645	response	SFM-F	0	5.566667	0	30.33333
GSM2804879	FOLFOX	PR	GSE104645	response	SFM-F	0	28.66667	0	42.83333
GSM2804946	FOLFIRI	SD	GSE104645	non.response	SFM-F	0	15.46667	0	34.8
GSM2804954	FOLFOX	SD	GSE104645	non.response	SFM-F	0	25.76667	0	71.4
GSM2804991	FOLFOX	PR	GSE104645	response	SFM-F	0	23.1	0	43.63333
GSM2805012	FOLFOX	SD	GSE104645	non.response	SFM-F	0	7.866667	0	13.66667
GSM2805014	FOLFOX	PR	GSE104645	response	SFM-F	0	10.5	1	19.7
GSM2805015	FOLFIRI	SD	GSE104645	non.response	SFM-F	1	15.16667	1	41.13333
GSM2805022	FOLFOX	SD	GSE104645	non.response	SFM-F	0	7.2	0	11.23333
GSM1875908	FOLFIRI	SD	GSE72970	non.response	SFM-F	1	17.08419	0	58.15195
GSM1875909	FOLFIRI	PR	GSE72970	response	SFM-F	1	8.180698	1	22.04517
GSM1875910	FOLFIRI	SD	GSE72970	non.response	SFM-F	1	6.472279	1	9.659138
GSM1875911	FOLFIRI	CR	GSE72970	response	SFM-F	1	11.72895	1	39.95072
GSM1875912	FOLFIRI	PR	GSE72970	response	SFM-F	1	10.71047	1	47.60575
GSM1875914	FOLFOX	PR	GSE72970	response	SFM-F	1	23.12936	0	53.38809
GSM1875917	FOLFOX	SD	GSE72970	non.response	SFM-F	1	13.14168	1	13.14168
GSM1875942	FOLFIRI	PR	GSE72970	response	SFM-F	1	10.31622	1	23.65503
GSM1875945	FOLFIRI	PR	GSE72970	response	SFM-F	1	12.9117	1	17.37988
GSM1875947	FOLFOX	PD	GSE72970	non.response	SFM-F	1	1.445585	1	5.749486
GSM1875948	FOLFOX	CR	GSE72970	response	SFM-F	1	17.64271	1	17.64271
GSM1875949	FOLFIRI	SD	GSE72970	non.response	SFM-F	1	3.712526	1	7.523614
GSM1875955	FOLFOX	PR	GSE72970	response	SFM-F	0	76.12321	0	76.12321
GSM1875959	FOLFOX	PD	GSE72970	non.response	SFM-F	1	1.675565	1	4.238193
GSM1875963	FOLFIRI	PR	GSE72970	response	SFM-F	1	25.98768	0	45.99589
GSM1875964	FOLFIRI	PD	GSE72970	non.response	SFM-F	1	1.544148	1	23.85216
GSM1875966	FOLFIRI	PR	GSE72970	response	SFM-F	1	14.35729	1	23.98357
GSM1875968	FOLFIRI	PD	GSE72970	non.response	SFM-F	1	1.774127	1	4.599589
GSM1875971	FOLFIRI	PR	GSE72970	response	SFM-F	1	12.38604	1	40.08213
GSM1875980	FOLFIRI	PR	GSE72970	response	SFM-F	1	9.232033	1	28.09035
GSM1875991	FOLFIRI	PR	GSE72970	response	SFM-F	1	6.373717	1	11.13758
GSM1875993	FOLFIRI	SD	GSE72970	non.response	SFM-F	1	16.52567	1	27.13758
GSM1875998	FOLFIRI	SD	GSE72970	non.response	SFM-F	1	5.125257	0	61.20739
GSM1876013	FOLFIRI	CR	GSE72970	response	SFM-F	1	51.25257	1	51.25257

D

GSE5851, PRJEB34338 datasets (cetuximab)				
sample_id	cetuximab	mole_subtype	batch	sfm
X1_T	n	not.available	PRJEB34338	SFM-C
X2_T	n	not.available	PRJEB34338	SFM-C
X47_T	n	not.available	PRJEB34338	SFM-C
X53_T	n	not.available	PRJEB34338	SFM-C
GSM136584	y	not_available	GSE5851	SFM-C
GSM136590	n	not_available	GSE5851	SFM-C
GSM136594	n	not_available	GSE5851	SFM-C
GSM136603	y	not_available	GSE5851	SFM-C
GSM136605	n	not_available	GSE5851	SFM-C
GSM136614	n	Stem-like	GSE5851	SFM-C
GSM136615	n	not_available	GSE5851	SFM-C
GSM136618	n	not_available	GSE5851	SFM-C
GSM136625	n	not_available	GSE5851	SFM-C
GSM136627	n	not_available	GSE5851	SFM-C
GSM136636	n	not_available	GSE5851	SFM-C
GSM136638	n	not_available	GSE5851	SFM-C
GSM136642	n	not_available	GSE5851	SFM-C
GSM136643	y	not_available	GSE5851	SFM-C
GSM136649	n	not_available	GSE5851	SFM-C
GSM136652	y	not_available	GSE5851	SFM-C
X3080_T	n	not.available	PRJEB34338	SFM-D/E/F
X4612_AR_T	n	not.available	PRJEB34338	SFM-D/E/F
X14001378T	n	not.available	PRJEB34338	SFM-D/E/F
PYM.R	n	not.available	PRJEB34338	SFM-D/E/F
PYM.S	y	not.available	PRJEB34338	SFM-D/E/F
GSM136578	n	Stem-like	GSE5851	SFM-D/E/F
GSM136582	y	Stem-like	GSE5851	SFM-D/E/F
GSM136586	n	Stem-like	GSE5851	SFM-D/E/F
GSM136587	n	Stem-like	GSE5851	SFM-D/E/F
GSM136588	y	Stem-like	GSE5851	SFM-D/E/F
GSM136592	n	Stem-like	GSE5851	SFM-D/E/F
GSM136593	y	Stem-like	GSE5851	SFM-D/E/F
GSM136597	n	Stem-like	GSE5851	SFM-D/E/F
GSM136599	y	Stem-like	GSE5851	SFM-D/E/F
GSM136601	n	Stem-like	GSE5851	SFM-D/E/F
GSM136608	n	Stem-like	GSE5851	SFM-D/E/F
GSM136616	n	Goblet-like	GSE5851	SFM-D/E/F
GSM136630	n	Stem-like	GSE5851	SFM-D/E/F
GSM136645	n	Stem-like	GSE5851	SFM-D/E/F
X3772T	n	not.available	PRJEB34338	SFM-A/B
X12184612_S_T	y	not.available	PRJEB34338	SFM-A/B
X13004198T	n	not.available	PRJEB34338	SFM-A/B
X13004222T	n	not.available	PRJEB34338	SFM-A/B
X14000456T	n	not.available	PRJEB34338	SFM-A/B
X24_T	y	not.available	PRJEB34338	SFM-A/B
X32215804.T1.Lv	y	not.available	PRJEB34338	SFM-A/B
X32215804.T2.Co	y	not.available	PRJEB34338	SFM-A/B
X46_T	n	not.available	PRJEB34338	SFM-A/B
X54_T	y	not.available	PRJEB34338	SFM-A/B
X55_T	y	not.available	PRJEB34338	SFM-A/B
X65_T	n	not.available	PRJEB34338	SFM-A/B
X69_T	y	not.available	PRJEB34338	SFM-A/B
X72_T	y	not.available	PRJEB34338	SFM-A/B
X73_T	n	not.available	PRJEB34338	SFM-A/B
GJG.S.Tumor	y	not.available	PRJEB34338	SFM-A/B
JJ.R.Tumor	n	not.available	PRJEB34338	SFM-A/B
JJ.S.Tumor	y	not.available	PRJEB34338	SFM-A/B
GSM136579	n	Goblet-like	GSE5851	SFM-A/B
GSM136580	n	TA	GSE5851	SFM-A/B
GSM136581	n	TA	GSE5851	SFM-A/B
GSM136585	n	Goblet-like	GSE5851	SFM-A/B
GSM136589	y	TA	GSE5851	SFM-A/B
GSM136591	n	Goblet-like	GSE5851	SFM-A/B
GSM136595	n	TA	GSE5851	SFM-A/B
GSM136596	n	TA	GSE5851	SFM-A/B
GSM136598	n	TA	GSE5851	SFM-A/B
GSM136600	n	Stem-like	GSE5851	SFM-A/B
GSM136602	y	not_available	GSE5851	SFM-A/B
GSM136606	n	not_available	GSE5851	SFM-A/B
GSM136609	y	TA	GSE5851	SFM-A/B
GSM136610	y	TA	GSE5851	SFM-A/B
GSM136611	y	TA	GSE5851	SFM-A/B
GSM136612	n	not_available	GSE5851	SFM-A/B
GSM136617	y	TA	GSE5851	SFM-A/B
GSM136619	n	TA	GSE5851	SFM-A/B
GSM136620	n	Goblet-like	GSE5851	SFM-A/B
GSM136621	n	TA	GSE5851	SFM-A/B
GSM136622	y	not_available	GSE5851	SFM-A/B
GSM136623	n	Goblet-like	GSE5851	SFM-A/B
GSM136624	y	TA	GSE5851	SFM-A/B
GSM136626	y	TA	GSE5851	SFM-A/B
GSM136628	y	TA	GSE5851	SFM-A/B
GSM136629	n	TA	GSE5851	SFM-A/B
GSM136631	n	TA	GSE5851	SFM-A/B
GSM136632	y	TA	GSE5851	SFM-A/B
GSM136633	y	Goblet-like	GSE5851	SFM-A/B
GSM136634	y	TA	GSE5851	SFM-A/B
GSM136637	y	TA	GSE5851	SFM-A/B
GSM136641	n	TA	GSE5851	SFM-A/B
GSM136647	n	not_available	GSE5851	SFM-A/B
GSM136648	n	TA	GSE5851	SFM-A/B
GSM136650	n	TA	GSE5851	SFM-A/B
GSM136651	y	TA	GSE5851	SFM-A/B
GSM136653	y	TA	GSE5851	SFM-A/B
GSM136654	y	TA	GSE5851	SFM-A/B

Supplementary Table 9. Estimation of proportions of immune cell populations, gene signatures and immune checkpoint markers

immune cell fraction from CIBERSORT

	B.cells.naiv	B.cells.men	Plasma.cell	T.cells.CD8	T.cells.CD4	T.cells.CD4	T.cells.CD4	T.cells.follic	T.cells.regu	T.cells.gam	NK.cells.res	NK.cells.act	Monocytes	Macrophag	Macrophag	Macrophag	Dendritic.c	Dendritic.c
GSM971957	0	0.0507162	0.0959973	0.0820469	0	0.0250235	0.132896	0.0278886	0	0	0.0077104	0.0516353	0	0.1035528	0.0701162	0.1600469	0.0438761	0
GSM971958	0	0.0977094	0.1367345	0.0241512	0.0401835	0.0312525	0.0406362	0.0016659	0	0	0.0488319	0	0	0.2994603	0.0103522	0.1953669	0	0
GSM971959	0	0.0846397	0.2136025	0.057304	0	0.0090986	0	0.0157903	0.0263632	0	0.0034417	0.0015806	0	0	0.0082164	0.2053301	0.0005	0.0156839
GSM971960	0.0418613	0	0.4918877	0.0394799	0.0347803	0.0624137	0.045105	0.0103319	0	0	0.0415422	0	0.0137427	0.0115931	0.0038525	0.1402784	0	0
GSM971961	0	0.0319123	0.1746649	0.0321221	0	0	0.0128917	0.0133801	0.0198632	0	0.0307357	0	0.0013949	0.3984561	0.0057604	0.1953023	0	0
GSM971962	0.014071	0	0.2998496	0.0353509	0	0.0500447	0	0	0.0355898	0	0.0614043	0	0	0.1829746	0.0538604	0.1848016	0	0
GSM971963	0	0.0559773	0.1048123	0.1479566	0	0	0.1427314	0.0332647	0	0	0.0560141	0.0304675	0	0.0650634	0.110299	0.1836299	0.018817	0.0020132
GSM971964	0	0.0214883	0.4262117	0.0591663	0	0.1331027	0.0290567	0.0140284	0	0	0.0167902	0.0197417	0.0074186	0.0421207	0.001129	0.1079493	0.0477317	0.039765
GSM971965	0.0187445	0	0.2739167	0.057315	0.0006	0.0915928	0	0	0.0273625	0	0.0470157	0	0	0.1147716	0.0319453	0.2495027	0	0.0148675
GSM971966	0	0.1505047	0.1597733	0.0805429	0	0.0815084	0	0	0.0387985	0	0.0287292	0	0.0097596	0	0.0227821	0.247454	0.016068	0.0397651
GSM971967	0	0.0008	0.0469617	0.0457922	0	0.2450589	0.0039062	0.0262094	0	0	0.0449833	0.018613	0.0135331	0.0735846	0.0166242	0.1631041	0	0.0419356
GSM971968	0	0.103312	0.0870252	0.0457568	0	0.0618189	0	0.0138963	0.0173881	0	0	0.0606315	0.0591325	0.1439516	0.0211772	0.228382	0.05628	0.0213236
GSM971969	0	0.0839689	0.1938239	0.0807841	0	0.0747433	0.0496811	0.0400326	0.0135676	0.0646555	0	0	0	0.0884757	0.0840567	0.201697	0.0073609	0.0070476
GSM971970	0	0.0390235	0.0679303	0.1073047	0	0.1393749	0	0.1060683	0	0	0.1029734	0	0	0.0355409	0.0486309	0.2215343	0.0213747	0.0144453
GSM971971	0.0115906	0	0.217924	0.1715034	0	0.0316291	0.0616685	0.0451438	0	0	0.0094733	0.0218121	0	0.065997	0.1167871	0.1471426	0.0311037	0.0082988
GSM971972	0.0036173	0.0062161	0.2442624	0.173326	0	0.0459702	0.0528292	0.0508151	0	0	0.0218581	0.0299011	0.0255329	0.0199989	0.090227	0.1998216	0.018046	0
GSM971973	0	0.0629421	0.0306834	0.1364456	0	0	0	0.0566673	0.0346111	0	0.0168562	0.0122391	0	0.1286097	0.0411974	0.2770897	0.0200529	0.0108644
GSM971974	0	0.0315467	0.2985596	0.0609264	0.0171264	0.2038237	0	0.0098564	0.0174653	0	0.0500595	0	0	0.0360979	0.006468	0.1597288	0.0304147	0.0157918
GSM971975	0	0.1413386	0.1974679	0	0.0134766	0.1203928	0.0786555	0	0	0	0.1294875	0	0.0122366	0.0963423	0.0011687	0.0548657	0	0.0153258
GSM971976	0	0.1164031	0.227342	0.0926936	0.0508701	0.0906669	0.0843274	0.0039372	0	0	0.0491648	0	0.0034256	0	0.0625382	0.1352118	0.0132952	0
GSM971977	0	0.01022	0.2485965	0.0321598	0	0.0932601	0	0.0668443	0.0280847	0	0.0570251	0	0	0.0239626	0.090473	0.2044833	0.008723	0
GSM971978	0.0178581	0	0.24879	0.1420003	0	0.0021216	0.1027009	0.0667505	0	0	0.0279391	0.0251583	0	0.0501367	0.1022525	0.1673819	0.0136879	0
GSM971979	0	0.0253023	0.0192217	0.0053214	0	0.0712706	0	0.0017334	0	0	0.0275772	0	0	0.3055402	0.0138018	0.0902382	0	0.0063005
GSM971980	0.014327	0	0.308312	0.1394972	0	0.0999918	0.0176928	0.0113423	0	0	0.0164918	0.0062488	0	0.0001	0.1319564	0.1634652	0.0007	0.0452072
GSM971981	0	0.0432992	0.2278773	0.1314588	0	0	0.0313806	0.060102	0	0.0019176	0	0.0089109	0	0	0.1216667	0.2328596	0.0408404	0.0070661
GSM971982	0	0.1242248	0.0578918	0.0300556	0	0.052534	0.0743607	0.0403194	0	0	0.015611	0.0352304	0	0.0674288	0.1304361	0.1985195	0.0085467	0.0045049
GSM971983	0	0.0255304	0.1846393	0.0930093	0	0.0183637	0.0430379	0.0818968	0.0098444	0	0.0359189	0	0	0.1418431	0.0882683	0.1497917	0.0071377	0.0017055
GSM971984	0	0.1864602	0.1409126	0.0779633	0	0.0267933	0.088157	0.0422448	0	0	0.0274487	0.0246183	0.0203018	0.0926127	0.1051602	0.145416	0.0162417	0
GSM971985	0	0	0.4109818	0.0747788	0	0.2887785	0.0048774	0	0	0	0.023119	0	0	0.0029614	0.0129795	0.0776155	0.0412079	0.015086
GSM971986	0	0.0265746	0.2936382	0.082418	0	0.0977287	0.0238673	0.0365407	0.0108456	0	0.0500094	0	0	0.0710137	0.0532202	0.1775074	0	0
GSM971987	0	0.0466418	0.2464399	0.0955283	0.0334538	0.1547561	0	0.0042905	0	0.0004	0.005618	0	0	0	0.0293248	0.1587941	0.0982729	0.010823
GSM971988	0	0.0261303	0.1924341	0.1605739	0	0.1592846	0	0	0.0514137	0	0.0125572	0.0148692	0	0.0429365	0.038434	0.1484412	0.009049	0.070175
GSM971989	0	0.028782	0.4221168	0.0521205	0	0.2051306	0	0.0510406	0.0069422	0	0.0200238	0.0108633	0.0064224	0	0.0135383	0.0898993	0	0.0426993
GSM971990	0	0.0828121	0.2752081	0.073421	0	0.1059696	0.0327454	0.0305059	0	0	0.0229968	0.0142937	0	0.0715912	0.0647673	0.1532117	0.0077902	0.0128777
GSM971991	0	0.1141714	0.1627126	0.0394054	0	0.0678355	0.0717736	0.0194444	0	0	0.0578736	0	0	0.1987768	0.0495526	0.0900259	0	0.0092854
GSM971992	0	0.0810479	0.3651066	0.02227	0.0438872	0.0878228	0.045067	0.026473	0	0	0.0244014	0	0	0.0426579	0.0486169	0.1498785	0.007481	0
GSM971993	0	0.1400553	0.2303502	0.1098375	0	0.0579108	0.0076391	0.0572016	0	0	0.0235116	0	0.0147915	0	0.0727186	0.1437248	0.0167155	0.012363
GSM971994	0.0080713	0	0.3475538	0.0747099	0	0.1445453	0	0.0124766	0	0	0.0452024	0.0276797	0.048733	0.0031401	0.0118323	0.1775342	0.002927	0.0037262
GSM971995	0	0	0.3532813	0.1005445	0	0.0640535	0	0.0258796	0.0156625	0	0	0.0326978	0	0	0.0533791	0.1933522	0.0877441	0
GSM971996	0.0167057	0	0.1802071	0.073144	0	0.0466146	0.0168983	0.0327293	0	0	0	0.0361328	0	0.2363735	0.0684319	0.1922615	0	0.013797
GSM971997	0.0591317	0	0.1794893	0.1706419	0	0.0654883	0.0926648	0.0118574	0	0	0.0284084	0.0202548	0	0.0473897	0.1440128	0.1269909	0.0168454	0
GSM971998	0.01284	0	0.3964318	0.1038098	0.01114	0.084995	0.0110549	0	0	0	0.0315651	0	0	0	0.0570696	0.183477	0.0252094	0.0091082
GSM971999	0.0246728	0.0030502	0.2902087	0.0294843	0	0.1473667	0.0936922	0.049812	0	0	0.0354702	0	0.0117978	0.048968	0.0275429	0.1823412	0.0067631	0.0109026
GSM972000	0	0.1487567	0.0837073	0.162715	0.016966	0.0337712	0	0.0084662	0	0	0.029257	0	0.0269342	0.0769132	0.0433536	0.2902938	0.020308	0
GSM972001	0	0.0436033	0.4746093	0.1042312	0	0.0987741	0.0347629	0.008763	0.0069641	0	0.014953	0	0	0.0325236	0.0297832	0.0915093	0.0031184	0.0309913
GSM972002	0	0	0.1374036	0.0795335	0	0.0595369	0	0.0104062	0.011376	0	0.0370794	0	0	0.1173291	0.0400633	0.13261	0	0.021462
GSM972003	0	0.1421256	0.4160606	0.0607214	0.002323	0.0518337	0	0.0165915	0	0	0.0935022	0	0.0008	0	0.0175878	0.0098216	0.0105312	0.0114772
GSM972004	0.01607	0	0.3902414	0.0307016	0.0096686	0.1218424	0.0543495	0.0092748	0	0	0.0687865	0	0	0.0087577	0.0220518	0.1688158	0.0138712	0.0054571
GSM972005	0.0298163	0.039654	0.0187571	0.0062861	0	0.206447	0	0.0056798	0.0645732	0	0.098968	0	0.0565474	0	0.0078113	0	0.0404513	0
GSM972006	0.0199919	0	0.1824757	0.0794296	0	0.0836194	0.029771	0.0738189	0	0	0	0.0158245	0	0.0825858	0.0504475	0.2494105	0.0179327	0.005107
GSM972007	0.0068731	0	0.3668353	0.098408	0.0007	0.0390518	0.0038149	0.0081911	0.0213067	0	0.0085505	0.0072686	0.0475524	0.0225176	0.0146304	0.2728595	0.0158089	0.0143683
GSM972008	0	0.0497124	0.0915072	0.0333022	0	0.0744644	0.0162943	0.0171496	0	0	0.0213829	0	0.040042	0.1247278	0.0072501	0.206414	0	0.0399613
GSM972009	0	0	0.4731741	0.1164137	0	0.0706932	0	0.0072801	0.0028286	0	0.0096549	0.0078906	0	0	0.0070827	0.190033	0	0.0204669
GSM972010	0.0083125	0	0.1986083	0.0975895	0	0.044698	0.0662471	0.0044886	0	0	0.0082465	0.008916	0	0.1474304	0.0339566	0.1825843	0.006219	0.0269924
GSM972011	0.0309511	0	0.4333079	0.1050484	0.0111653	0	0	0.0393721	0.0076498	0	0	0.0354097	0.008945	0.0129732	0.1185553	0.1500106	0.0073924	0
GSM972012	0	0.1780017	0.2085866	0.0349729	0.0223906	0.1177192	0	0.0155294	0	0	0.1087181	0	0.0238342	0.0218267	0.0113217	0.0437696	0.0109241	0.0324108
GSM972013	0	0.0449376	0.1878485	0.0858758	0	0.0644311	0.0536151	0.0006	0.0007	0	0.0706815	0	0	0	0.0293908	0.2470871	0.0017304	0.0276093
GSM972014	0	0.0232284	0.1172515	0.063574	0	0.0666259	0	0.0250614	0.0277119	0	0	0.0037069	0	0.067064	0.0288527	0.1630421	0.0023018	0.0459279
GSM972015	0	0.0438993	0.0501047	0.1485326	0	0	0.0058388	0.0585461	0.0047176	0	0.0729246	0.018027	0	0.0958387	0.1193475	0.2308058	0	0.0258662
GSM972016	0.0148269	0	0.310857	0.0312386	0.0043546	0.0307562	0	0.0303692	0.0045159	0	0.0452698	0	0	0.1812481	0.0071192	0.1082758	0	0.0157797
GSM972017	0	0.0542015	0.2327247	0.0816271	0.0453302	0.0555811	0.0096833	0.0228233	0	0	0.0446246	0	0	0.1701745	0.0203304	0.1672653	0.0117404	0
GSM972018	0	0.1357241	0.1575666	0.0345784	0	0.0714563	0.0152181	0.0820798	0	0	0	0.0275481	0	0.0078256	0.1370101	0.1981464	0.0176236	0.0101717
GSM972019	0	0.1559456	0.1622943	0.1521667	0	0.0110742	0.0197202	0.1378072	0.0129579	0.0198164	0	0	0.0097407	0	0.0651705	0.1744469	0.0240925	0.0132167
GSM972020	0.0359882	0	0.4326979	0.0347994	0	0.1346076	0.009528	0.0218445	0.0095932	0	0.0496195	0	0	0.0100757	0.0264857	0.0826128	0.027248	0.0263166
GSM972021	0	0.1398842	0.0954603	0.0239219	0	0.1461437	0	0.0092813	0.0318995	0	0.0905218	0	0	0.1110966	0.0121102	0.1377152	0.0153321	0.0195597
GSM972022	0	0.0289257	0.3517276	0.1326248	0.0060954	0.0579989	0	0.0151189	0	0	0.020878	0	0.0160719	0.0487528	0.0883445	0.1352597	0.0181368	0
GSM972023	0	0.0527285	0.1878567	0.0380016	0.0542224	0.0849136	0	0	0	0	0.0396781	0	0	0.2392325	0.0225143	0.1035579	0	0
GSM972024	0	0.0546691	0.0907588	0.0223307	0.0135274	0.1610809	0.1010361	0.001445	0	0	0.1043708	0	0.0058574	0.0555908	0.0119284	0.1812647	0.0466917	0.0110452
GSM972025	0	0	0.4139592	0.1240287	0	0.1484165	0	0	0.0080327	0	0	0.0172897	0	0.0088602	0.0231598	0.1531631	0.0351946	0
GSM972026	0.0172841	0.0025034	0.1226697	0.1190013	0.0222941	0.0111379	0.1239975	0	0	0	0	0.0655306	0.0169123	0	0.0264641	0.3174699	0.0277956	0.0201381
GSM972027	0	0.1506712	0.1429095	0.0677079	0.0518959	0	0.0686436	0	0	0	0.0940858	0	0.0660535	0.1489088	0	0.1138078	0.0177362	0.0020334
GSM972028	0.0058294	0	0.3851207	0.0588314	0	0.1108988	0.0322405	0	0.0082012	0	0.0517696	0	0	0	0.0281416	0.1780242	0.0472001	0.0003
GSM972029	0.0168392	0	0.2729455	0.0989808	0.0133685	0.1311539	0	0.0082806	0	0	0.0687559	0	0	0.0839447	0.017232	0.1879309	0	0.0100077
GSM972030	0	0.184728	0.2058545	0.0792103	0.0343417	0.0979826	0	0	0	0	0.0679662	0	0.0383092	0	0.0233672	0.1366501	0.0024183	0.017098
GSM972031	0	0.1960047	0.2525087	0.0197192	0.0217524	0.1042996	0	0.0214952	0	0	0.0844174	0	0.0281702	0.0409045	0.025354	0.0790829	0.0172265	0.0038313
GSM972032	0	0.1725152	0.2197345	0.0072206	0.0432473	0.1127222	0.0892124	0.0318341	0	0	0.0787918	0	0.0295094	0.0369566	0.0089672	0.0740103	0.0078185	0.0218949
GSM972033	0	0.0952712	0.1776954	0.1484717	0	0.0799544	0.0091825	0.0370777	0.0340571	0	0.0385171	0.0015806	0	0	0.0009	0.233721	0.0154269	0.0275192
GSM972034	0	0.2788878	0.2237201	0.0159425	0.0547453	0.0714315	0.0037269	0	0	0	0.0494943	0	0.0193226	0	0.0110812	0.0743402	0.0162545	0.0415225
GSM972035	0	0.046641	0.1048073	0.0528448	0	0.1734602	0.052956	0.0192189	0.0157864	0	0.0513573	0	0	0	0.0168356	0.3034284	0.0622534	0.042708
GSM972036	0.0279324	0	0.3984235	0.030476	0	0.1637331	0.0412671	0	0	0	0.0730907	0	0	0.0302315	0.0386047	0.1278622	0.0228745	0
GSM972037	0	0.096408	0.2724783	0.0941022	0	0.1219478	0	0.0272184	0	0	0.0329234	0	0	0.0449305	0.0707065	0.1595601	0.0041205	0.0301289
GSM972038	0.0095235	0.046191	0.115816	0.1120819	0	0.0403475	0.0077553	0.0686612	0.039998	0	0.0096367	0.0281253	0.0043803	0.0383343	0.0367834	0.2511001	0.033624	0.0691497
GSM972039	0	0.1433868	0.4346162	0.0668412	0	0.0539716	0	0.0814887	0	0.0062627	0	0	0.0370786	0	0.0101356	0.0595843	0	0.0197408
GSM972040	0	0.1052765	0.1796309	0.0415295	0.0163661	0.1368155	0.0794473	0	0	0	0.0577815	0	0.0130628	0.0913401	0.0114777	0.1442898	0.0171583	0.055018
GSM972041	0	0.1430422	0.098129	0.0251163	0.0168978	0.1431904	0.0428827	0.0209684	0	0	0.0950386	0.0077284	0.0528454	0.09954	0.0225692	0.0743917	0.0095145	0.0322914
GSM972042	0	0.0150687	0.5297236	0.036512	0	0.1222528	0.0015674	0	0.0254133	0	0.0137833	0.0004	0	0.0449968	0.0606611	0.0545755	0.0421775	0.013241
GSM972043	0	0.1258205	0.1072233	0.0621261	0	0.131238	0.1022272	0.019452	0	0	0.0928981	0	0.0216602	0.0673758	0.0335539	0.1647134	0.0150478	0.02571
GSM972044	0.003338	0	0.1232448	0.0394686	0	0.1489172	0	0.01985	0.0765726	0	0.019804	0	0	0.0697389	0.0022889	0.1756013	0.0078003	0.0296729
GSM972045	0.0033276	0.0808526	0.1194979	0.1158616	0	0.0298687	0	0.1292389	0.0544773	0	0.0663113	0	0	0.0258562	0.0115372	0.1753142	0	0.0330485
GSM972046	0	0.0725958	0.1279356	0.0676738	0	0.1710371	0.1410401	0.0127808	0	0	0.0128019	0.012332	0.0111385	0	0.0421474	0.2809712	0.0091905	0
GSM972047	0	0.1051973	0.1356095	0.0286061	0.0282368	0.1728115	0.0313971	0	0	0	0.0432143	0.0049989	0.0033162	0.1080207	0.0154511	0.222906	0.0070514	0
GSM972048	0	0.0349227	0.3365038	0.0586304	0.0209814	0.0798424	0	0	0	0	0.0799682	0	0.0095812	0.065091	0.0081972	0.1998042	0.002366	0.0256346
GSM972049	0	0.0452106	0.0792665	0.0864129	0	0.1301749	0	0.0310043	0.0334354	0	0.0679424	0	0	0.0894473	0.0074103	0.2546809	0	0.023694
GSM972050	0	0.0030537	0.5766909	0.0778029	0	0.180407	0	0	0	0	0.0147747	0	0.0071205	0	0.0023345	0.0800792	0.012844	0.0235101
GSM972051	0	0.0112427	0.0989195	0.0709067	0	0.0748726	0.0561948	0.0196907	0	0	0.0361475	0	0.0034671	0.0855968	0.0430678	0.3439295	0	0.0249844
GSM972052	0	0.1732206	0.3455589	0	0.0265279	0.121116	0.0646496	0	0	0	0.0429429	0	0	0.0293587	0.0259553	0.1045085	0	0.023514
GSM972053	0	0.2440532	0.1166314	0.0365591	0.0099231	0.1248796	0.1054077	0.028881	0	0	0.0897625	0	0.049155	0.0371654	0.0732823	0.0272318	0.0344352	0.0054821
GSM972054	0.0240598	0.0025648	0.141612	0.0771545	0	0.0217263	0.0137036	0.066335	0	0	0.0402276	0	0	0.2406279	0.057368	0.2060074	0	0.0014835
GSM972055	0	0.0456981	0.2542064	0.0600573	0.0109943	0.0567727	0.0620661	0.0269181	0	0	0.0633591	0.0297085	0	0.0642207	0.0595866	0.2122498	0.0162066	0
GSM972056	0	0.0942053	0.1644136	0.0424314	0.0303861		0	0.0693941	0.033206	0.0512164	0.0801292	0		0.0491864	0.037548	0.0840348	0.0061545	0.0360233
GSM972057	0	0.0861949	0.1222301	0.1698339	0	0.0452779	0.0601838	0	0	0	0.0598969	0	0.0301432	0	0.0233467	0.2230407	0.0256627	0
GSM972058	0.0262842	0	0.1203413	0.0361429	0	0.1355145	0	0.056541	0	0	0.0101526	0.0333322	0	0.0952924	0.0430722	0.1710629	0	0.0230644
GSM972059	0	0.0884841	0.2228326	0.1399368	0.017716	0	0.0502157	0.0254417	0	0	0.0786582	0	0.0070708	0.0394021	0.0483423	0.1364609	0.0157161	0
GSM972060	0	0.0551416	0.0948962	0.0834161	0	0.0803089	0.0473976	0.0666366	0.0224092	0	0	0.0509002	0	0.0552994	0.0848125	0.238338	0.0451487	0.0149786
GSM972061	0	0.0236771	0.4007275	0.096405	0.0321172	0.0172393	0.0584772	0.0048817	0	0	0.0172412	0	0	0.0781644	0.0251346	0.2027104	0	0
GSM972062	0.0111515	0	0.3434283	0.0718153	0	0.1260287	0.0127349	0.0068447	0	0	0.0091544	0.0035271	0	0.0041346	0.0082989	0.1817607	0.0784454	0.012716
GSM972063	0.018787	0	0.2956457	0.0584788	0.0064904	0.0980729	0.0784589	0	0	0	0.0288321	0	0	0.0765864	0.0599669	0.2025947	0.0096046	0
GSM972064	0	0.0445698	0.248399	0.0503428	0	0.0842416	0	0	0.0075711	0	0	0.012393	0	0.0995996	0.0295945	0.1748453	0.0074553	0
GSM972065	0.0152347	0	0.3823958	0.0263834	0.0012572	0.1033795	0.047104	0.0126101	0	0	0.0456958	0	0.0028512	0.0315661	0.0336415	0.1956863	0.0011439	0.004005
GSM972066	0.0155653	0	0.4109127	0.0338129	0	0.1019777	0.0550475	0	0	0	0.0114158	0	0	0.1252783	0.0272634	0.1252019	0	0.004604
GSM972067	0	0.036415	0.4087558	0.0908929	0.0014727	0.0233564	0	0.0220431	0.0191547	0	0	0.0406222	0	0.0322487	0.0614979	0.2170695	0.0193787	0
GSM972068	0	0.0539356	0.1322671	0.0655413	0	0.0318488	0	0.0057982	0.0336006	0	0.0090007	0.0011884	0	0.2021997	0.0299783	0.3119083	0.012885	0
GSM972069	0	0.1110963	0.3364138	0.0637466	0.030841	0.0134648	0.0579655	0.0205098	0	0	0.073958	0	0	0.0263605	0.013861	0.1577714	0.008143	0
GSM972070	0.0073122	0	0.4923006	0.0468762	0	0.088092	0	0	0.0188805	0	0	0.0125929	0	0.0119497	0.0120406	0.2040197	0	0.0031852
GSM972071	0	0.0452337	0.1205592	0.0208019	0.0131011	0.0855334	0	0.0005	0.0385524	0	0.0760501	0	0.0028654	0.224676	0.0103561	0.1833492	0.0184976	0.0080618
GSM972072	0	0.1024385	0.134284	0.0742081	0	0.0815899	0.0213511	0.0577496	0	0.019696	0	0.0951319	0	0.06434	0.1028614	0.1680742	0.0128997	0.006464
GSM972073	0	0.0035939	0.2616311	0.0324187	0	0.1049469	0.0817014	0.0250698	0	0	0.0271183	0.0044065	0	0.1448764	0.0737045	0.1741499	0.0047963	0.0038619
GSM972074	0.0185009	0	0.3528698	0.0441685	0.0155255	0.0960213	0.0532229	0	0	0	0.0497962	0	0.0100639	0.0388007	0.0397999	0.1513325	0.0172281	0
GSM972075	0	0.0489118	0.1888879	0.150325	0	0.0414735	0.0128936	0.0173831	0	0	0	0.0372517	0	0.0886245	0.081066	0.252297	0.0165132	0
GSM972076	0	0.015647	0.16802	0.1084837	0	0.0654873	0.0767365	0.0240494	0	0	0.0382411	0.0502804	0	0.1399468	0.1075402	0.1471775	0.003786	0
GSM972077	0	0.0174229	0.3705555	0.1341959	0	0.0131794	0.0071318	0.0465108	0.0410516	0	0.0306475	0	0	[illegible]	0.0099249	0.2097796	0.0446579	0
GSM972078	0.0171694	0.0029761	0.1201789	0.0287075	0	0.1428121	0.0463746	0.0197772	0	0	0.0645271	0	0.0210318	0.1507195	0.0531837	0.1766298	0	0
GSM972079	0.0290637	0.0015186	0.2286935	0.0823708	0	0.0058875	0.0446778	0.0283235	0.0351523	0	0.0918084	0	0	0.0750123	0.0318201	0.2145172	0.0085417	0

[illegible]

Supplementary Table 10. Top 15 abundance microbes in each subtype

SFM-A		SFM-B		SFM-C		SFM-D		SFM-E		SFM-F	
**Genus**	**%**	**Genus**	**%**	**Genus**	**%**	**Genus**	**%**	**Genus**	**%**	**Genus**	**%**
escherichia.coli	28	escherichia.coli	57.7	escherichia.coli	37.7	escherichia.coli	35	escherichia.coli	41.7	escherichia.coli	30.4
propionibacterium.acnes	10.9	propionibacterium.acnes	6.4	propionibacterium.acnes	6.1	propionibacterium.acnes	7.3	propionibacterium.acnes	7.4	propionibacterium.acnes	8.4
pseudomonas.fluorescens	2.1	acinetobacter.baumannii	1.4	fusobacterium.nucleatum	4.5	bacteroides.fragilis	2.3	pseudomonas.fluorescens	2	pseudomonas.fluorescens	2.8
acinetobacter.baumannii	2.1	fusobacterium.nucleatum	1.1	bacteroides.fragilis	3.4	fusobacterium.nucleatum	2.3	acinetobacter.baumannii	1.4	fusobacterium.nucleatum	2.5
fusobacterium.nucleatum	1.7	pseudomonas.fluorescens	1	pseudomonas.fluorescens	1.9	bacteroides.vulgatus	1.7	ralstonia.pickettii	1.3	bacteroides.fragilis	2.1
staphylococcus.epidermidis	1.5	bacteroides.vulgatus	1	acinetobacter.baumannii	1.5	rhodopseudomonas.palustris	1.7	fusobacterium.nucleatum	1.1	ralstonia.pickettii	1.7
ralstonia.pickettii	1.4	staphylococcus.epidermidis	0.9	bacteroides.thetaiotaomicron	1.2	[clostridium].saccharolyticum	1.3	staphylococcus.epidermidis	0.9	acinetobacter.baumannii	1.6
bacteroides.fragilis	1.3	ralstonia.pickettii	0.7	ralstonia.pickettii	1.1	pseudomonas.fluorescens	1.1	bacteroides.fragilis	0.9	bacteroides.vulgatus	1.3
pseudomonas.mendocina	1.1	pseudomonas.putida	0.6	bacteroides.vulgatus	1.1	oscillibacter.valericigenes	1.1	pseudomonas.putida	0.9	pseudomonas.putida	1.2
staphylococcus.aureus	0.8	streptococcus.pneumoniae	0.5	[clostridium].saccharolyticum	1	pseudomonas.putida	0.9	bacteroides.vulgatus	0.8	[clostridium].saccharolyticum	1
pseudomonas.putida	0.8	staphylococcus.aureus	0.5	pseudomonas.putida	0.8	roseburia.hominis	0.7	staphylococcus.aureus	0.6	staphylococcus.epidermidis	0.9
agrobacterium.sp..h13-3	0.7	bacteroides.fragilis	0.5	roseburia.hominis	0.7	acinetobacter.baumannii	0.6	[clostridium].saccharolyticu m	0.6	roseburia.hominis	0.8
micrococcus.luteus	0.7	pseudomonas.mendocina	0.4	staphylococcus.epidermidis	0.6	microbacterium.testaceum	0.6	pseudomonas.mendocina	0.5	pseudomonas.mendocina	0.7
pseudomonas.aeruginosa	0.6	pseudomonas.aeruginosa	0.3	pseudomonas.stutzeri	0.6	paracoccus.denitrificans	0.6	pseudomonas.aeruginosa	0.5	corynebacterium.aurimucosum	0.7
streptococcus.pneumoniae	0.6	shigella.sonnei	0.3	pseudomonas.aeruginosa	0.5	[eubacterium].rectale	0.6	roseburia.hominis	0.5	pseudomonas.aeruginosa	0.7

data for drawwing Fig. 5

	CRSS_A	CRSS_B	CRSS_(	CRSS_D	CRSS_E	CRSS_F	p.value
Pseudomonas.mendocina	1.062	0.368777577	0.486	0.169491673	0.541	0.706040387	######
escherichia.coli	27.97	57.70114047	37.71	34.98348652	41.65	30.38986482	######
Rhodopseudomonas.palustri	0.362	0.142685299	0.156	1.703969399	0.24	0.187046759	######
Agrobacterium.sp..h13-3	0.742	0.312669231	0.373	0.044327268	0.394	0.547589297	2E-07
Fusobacterium.nucleatum	1.716	1.084502	4.464	2.284216035	1.135	2.460814015	2E-05
Oscillibacter.valericigenes	0.154	0.138129817	0.375	1.05589719	0.32	0.430715524	7E-05
[Clostridium].saccharolyticun	0.273	0.327511563	0.982	1.349365442	0.627	0.974617501	0.0001
Bacteroides.fragilis	1.31	0.469511487	3.393	2.319138398	0.94	2.123527165	0.0002
[Eubacterium].rectale	0.198	0.164780589	0.492	0.574876564	0.358	0.593671562	0.0002
Roseburia.hominis	0.238	0.262849471	0.734	0.653166828	0.452	0.783240718	0.0005
Microbacterium.testaceum	0.188	0.105133173	0.115	0.602063757	0.208	0.149447129	0.0005
Ralstonia.pickettii	1.401	0.737701994	1.122	0.124468586	1.265	1.709520718	0.0007
Pseudomonas.fluorescens	2.106	0.997462089	1.858	1.106171809	1.978	2.756970225	0.0018
Micrococcus.luteus	0.728	0.313277249	0.241	0.228828176	0.316	0.327378875	0.0045
Staphylococcus.epidermidis	1.527	0.862064343	0.644	0.278019346	0.94	0.897266037	0.0046
Pseudomonas.aeruginosa	0.607	0.332291198	0.538	0.356011459	0.49	0.67332042	0.0195
Staphylococcus.aureus	0.827	0.47610077	0.414	0.385998938	0.65	0.487106471	0.0262
Propionibacterium.acnes	10.95	6.371077329	6.063	7.262427897	7.447	8.381980958	0.0386
Shigella.sonnei	0.2	0.329653293	0.263	0.372844823	0.266	0.187294847	0.0408
Bacteroides.thetaiotaomicroı	0.518	0.318176177	1.175	0.541609097	0.423	0.630584825	0.0506
Corynebacterium.aurimucosu	0.459	0.242939	0.319	0.371499447	0.285	0.703531095	0.0567
Streptococcus.pneumoniae	0.581	0.515711745	0.28	0.307148679	0.299	0.288951658	0.0579
Pseudomonas.putida	0.82	0.562078273	0.792	0.889922791	0.859	1.181991154	0.0905
Acinetobacter.baumannii	2.079	1.404405282	1.514	0.619177667	1.372	1.561749602	0.0935
Paracoccus.denitrificans	0.309	0.04575912	0.097	0.588394346	0.281	0.349542164	0.128
Pseudomonas.stutzeri	0.488	0.298898171	0.585	0.319854662	0.431	0.533309562	0.2232
Bacteroides.vulgatus	0.503	0.97520664	1.061	1.709571719	0.761	1.294990309	0.3019
